# Visible light-induced ruthenium(ii)-catalyzed hydroarylation of unactivated olefins†

**DOI:** 10.1039/d4sc06005a

**Published:** 2024-10-23

**Authors:** Sven Trienes, Stéphane Golling, Matthew H. Gieuw, Marco Di Matteo, Lutz Ackermann

**Affiliations:** a Wöhler Research Institute for Sustainable Chemistry (WISCh), Georg-August-Universität Tammannstraße 2 37077 Göttingen Germany Lutz.Ackermann@chemie.uni-goettingen.de; b DZHK (German Centre for Cardiovascular Research) Potsdamer Straße 58 10875 Berlin Germany

## Abstract

Hydroarylation reactions have emerged as a valuable tool for the direct functionalization of C–H bonds with ideal atom economy. However, common catalytic variants for these transformations largely require harsh reaction conditions, which often translate into reduced selectivites. In contrast, we herein report on a photo-induced hydroarylation of unactivated olefins at room temperature employing a readily available ruthenium(ii) catalyst. Our findings include high position- and regio-selectivity and remarkable tolerance of a wide range of functional groups, which further enabled the late-stage diversification.

## Abstract 2

Ruthenium-catalyzed photo-induced hydroarylation at room temperature shows high functional group tolerance allowing to incorporate biorelevant motifs. Mechanistic studies provided in-depth details on the light's role to generate the active catalyst.

## Graphical abstract

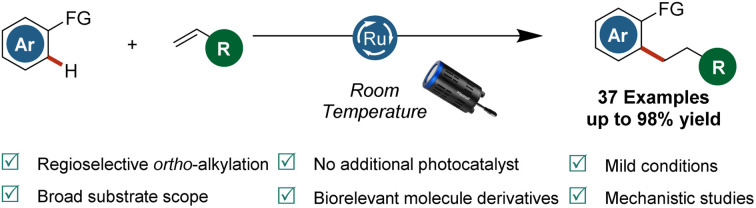

## Introduction

**Scheme 1:**
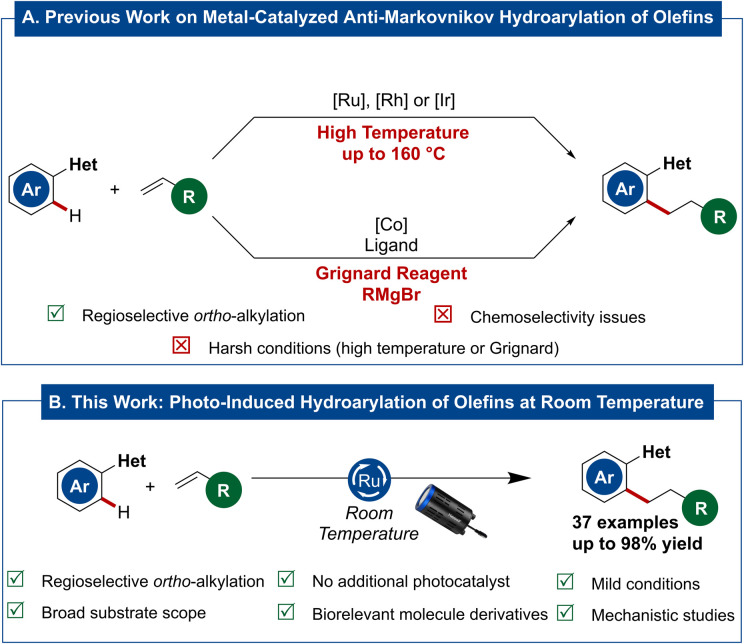
(A) Previous work: metal-catalyzed anti-Markovnikov hydroarylation of olefins; (B) this work: photo-induced ruthenium-catalyzed hydroarylation of non-activated olefins at room temperature.

During the last decades, C–H activation has surfaced as a powerful and sustainable tool in modern organic synthesis.^1–6^ In this context, ruthenium has emerged as a privileged metal for this type of transformation.^7,8^ Particularly, the direct aryl addition to C–C double or triple bonds *via* hydroarylation turned out to be an attractive and highly atom-economic approach for the direct functionalization of aromatic C(sp^2^)–H bonds. Different approaches were developed to realize the transition metal-catalyzed anti-Markovnikov hydroarylation of unactivated olefins. Prominent transition metal-catalysts for this transformation^9^ are primarily based on ruthenium^10–15^ – with pioneering contributions by Lewis^16^ and Murai^17^ – rhodium^18–23^ or iridium.^24–29^ However, these processes are of limited utility for late-stage functionalization, as they require elevated reaction temperatures. For the same reason, the degree of C–H bond functionalization can be difficult to control, as overfunctionalization is observed in some cases. To overcome this challenge, Yoshikai^30–32^ reported on the cobalt-catalyzed hydroarylation of olefins at room temperature. Although those reactions were performed at reduced temperature and therefore prevented the formation of the bis-alkylated product, they required a substoichiometric amount of Grignard reagents which translates into reduced functional group tolerance, and hence potentially limits viable late-stage transformations of complex molecules (Scheme 1A).

To circumvent the harsh reaction conditions represented either by high reaction temperatures or Grignard reagents, we envisioned that a photochemical approach could realize the desired transformation under significantly milder conditions. Additionally, visible light irradiation appears as sustainable and easily tunable driver for chemical reactions, comparable to electrochemistry,^33–36^ that has proven to be highly efficient in organic chemistry.^37–43^ Among a variety of different photochemical approaches, dual-catalytic systems with a transition metal-catalyst and an exogeneous photosensitizer, often based on ruthenium or iridium, showed remarkable efficiency. Alternatively, in some cases the transition metal-catalyst itself can show interesting photophysical properties, thereby superseding the addition of a separate photocatalyst. In the context of photochemical C–H functionalization, both with and without an exogenous photocatalyst, transformations utilizing transition metals,^44,45^ such as palladium,^46–51^ rhodium,^52,53^ copper^54–56^ or iron^57^ have been exploited. Another metal with remarkable synergistic effects of merging photochemistry and C–H activation is ruthenium.^58^ Within our program on photoinduced, ruthenium-catalyzed C–H functionalizations,^59–63^ we wondered whether a photo-induced ruthenium-catalyzed hydroarylation of non-activated olefins would indeed be viable at room temperature (Scheme 1B).

## Results and discussion

We commenced our studies by probing reaction conditions for the envisioned photo-enabled, room-temperature ruthenium-catalyzed hydroarylation of olefins with ketimine 1a as model substrate. [Ru(OAc)_2_(*p*-cymene)] turned out to be the catalyst of choice for this transformation, affording the corresponding alkylated ketone 2a in 85% yield after hydrolysis (Table 1, entry 1). Other ruthenium(ii) catalysts, such as [RuCl_2_(*p*-cymene)]_2_ or [Ru(O_2_CMes)_2_(*p*-cymene)], allowed the formation of the desired product 2a, albeit with lower efficacy (Table 1, entries 2 and 3). Several other arene-containing ruthenium-complexes were also employed giving comparable efficacy.^64^ The biscationic ruthenium-aqua complex^65^ [Ru(H_2_O)(*t-*BuCN)_5_][BF_4_]_2_ gave low yields, also in the presence of KOAc (Table 1, entry 4). Likewise, the arene-free, biscationic complex^66^ [Ru(MeCN)_6_][BF_4_]_2_ failed to furnish the desired product 2a in the absence of light, highlighting the unique features of the metallaphotocatalysis (Table 1, entry 5). RuCl_3_·3H_2_O and Ru_3_(CO)_12_ failed to give the desired product (Table 1, entries 6, 7). Additional carboxylate sources^67^ did not improve the catalytic performance (Table 1, entries 8, 9). Finally, a similar result was obtained when the *p*-methoxyphenyl (PMP) moiety was substituted by a 3,4,5-trimethoxyphenyl group (TMP) (Table 1, entry 10). Control experiments were conducted, leading to a complete inhibition of reactivity in the dark or without the ruthenium catalyst, mirroring their essential role in the catalysis (Table 1, entry 11).

**Table 1:** Optimized conditions and deviation effects^a^

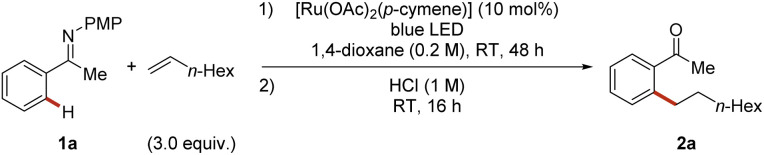		
Entry	Deviation from standard conditions	Yield 2a^b^ (%)
1	No deviation	85
2	[RuCl_2_(*p*-cymene)]_2_	32
3	[Ru(O_2_CMes)_2_(*p*-cymene)]	69
4	[Ru(H_2_O)(*t*-BuCN)_5_][BF_4_]_2_ + KOAc (20 mol%)	(6)
5^c^	[Ru(MeCN)_6_][BF_4_]_2_ + KOAc (20 mol%)	0
6	RuCl_3_·3H_2_O	0
7	Ru_3_(CO)_12_	0
8^d^	1-Cyclohexenecarboxylic acid as additive	85
9^d^	Boc-Leu-OH as additive	(30)
10	3,4,5-Trimethoxyphenyl instead of *p*-methoxyphenyl	68
11	Without light or without [Ru]	0

a Reaction conditions: 1a (0.20 mmol), 1-octene (0.60 mmol), [Ru(OAc)_2_(*p*-cymene)] (10 mol%), 1,4-dioxane (1.0 mL), room temperature (30–35 °C). Subsequent hydrolysis with 1 M HCl (3.0 mL). Detailed experimental procedures are shown in the ESI.

b Yields of isolated product. Yields in parentheses determined by ^1^H NMR using an internal standard.

c 24 h, 30 °C, in the dark.

d Using [RuCl_2_(*p*-cymene)]_2_ as catalyst. PMP = *p*-methoxyphenyl.

### Substrate scope evaluation

**Scheme 2:**
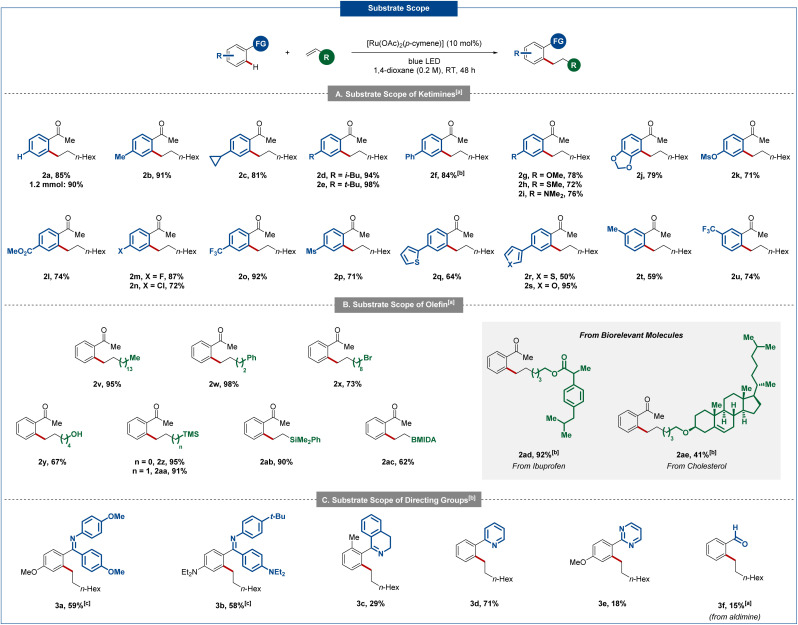
Substrate scope for various ketimines 1 with 1-octene. Reaction conditions: 1 (0.20 mmol), 1-octene (0.60 mmol), [Ru(OAc)_2_(*p*-cymene)] (10 mol%), 1,4-dioxane (1.0 mL), room temperature (30–35 °C). Detailed experimental procedures are shown in the ESI.† (a) Subsequent hydrolysis with 1 M HCl (3.0 mL). (b) 96 h reaction time. (c) 1.6 equiv. of 1-octene.

With the optimized conditions in hand, we explored the versatility of the direct hydroarylation of 1-octene with various ketimines 1 (Scheme 2A). Initially, the synthetic utility was successfully demonstrated by upscaling our standard reaction on 1.2 mmol scale yielding the alkylated compound 2a in 90% yield. Subsequently, a wide range of substituted ketimines 1 were subjected to our reaction conditions to evaluate the functional group compatibility. Noteworthy, numerous functionalities were well tolerated, and the mono-alkylated product was selectively obtained in all cases. Different alkyl substituents in the *para*-position (2b–2e) or a phenyl-group (2f) afforded the desired compounds in excellent yields. Additionally, the use of various electron donating groups such as methoxy (2g), thioether (2h), dimethylamine (2i), 1,3-dioxole (2j) or mesylate (2k) led to the targeted products in high yields. It is noteworthy that only one isomer was observed in the case of 1,3-dioxole derivative (2j). Other interesting functionalities, such as methyl ester (2l), *p*-fluoro (2m), *p*-chloro (2n), *p*-trifluoromethyl (2o) or even *p*-mesyl (2p) were well tolerated. Subsequently, the use of ketimines bearing a heterocycle in *para*-position were also smoothly transformed into the corresponding hydroarylation products with yields up to 95%, demonstrating the excellent functional group tolerance of this approach (2q–2s). Finally, substituents in the *meta*-position of the aromatic ring were also tolerated, and only one constitutional isomer was obtained (2t, 2u) in 59% and 74% yield, respectively.

Then, the effect of substituents on the alkene was explored (Scheme 2B). A longer alkyl chain did not impact the reactivity as ketone 2v was obtained in 95% yield. The use of an alkene bearing a phenyl ring led to an excellent yield of 98% (2w). A sensitive bromide derivative (2x) and a free alcohol (2y) were also compatible and afforded the corresponding compounds in good yields. Different silyl groups (2z-ab) proved to be suitable as well. Similarly, a boronic acid masking group (MIDA) was well tolerated (2ac). Finally, terminal alkenes incorporated in important biorelevant compounds were successfully transformed into the desired hydroarylation products. Therefore, the reaction was performed in the presence of ibuprofen (2ad) and cholesterol (2ae) derivatives giving the targeted products in acceptable to excellent yields.

Additionally, the ruthenium-catalyzed hydroarylation proved viable for different directing groups (Scheme 2C). The use of a bis(*p*-methoxyphenyl)imine led to the corresponding mono-alkylated product 3a in a satisfactory yield of 59%. A good yield of 58% was observed for bis(diethylamino phenyl)imine 3b. Moreover, a cyclic imine was employed to furnish the corresponding dihydroisoquinoline 3c in moderate yield. Furthermore, heteroarenes also enabled the photo-induced hydroarylation. Therefore, 2-phenylpyridine gave access to the alkylated product 3d in 80% yield. The mild conditions of the photo-induced C–H activation translated into improved mono-selectivities as compared to thermal mode of action.^10,64^ A reduced efficacy was observed for a pyrimidyl coordinating group (3e) and an aldimine (3f).

### Mechanistic investigations

**Scheme 3:**
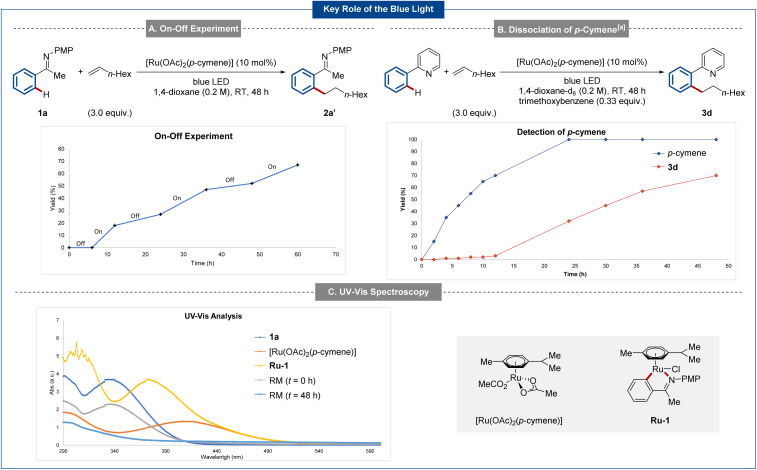
Key mechanistic findings on the role of the blue light. Detailed experimental procedures are shown in ESI.† (a) Relative yields referring to the initial amount of phenylpyridine (0.2 mmol) or [Ru(OAc)_2_(*p*-cymene)] (0.02 mmol). RM = reaction mixture.

To gain insights into the working mode of the metallaphotocatalysis, mechanistic studies were conducted. Initially, the role of the blue light irradiation was investigated in detail with an on-off experiment (Scheme 3A). Here, the essential role of the light was confirmed. Next, the reaction was monitored *via*^1^H NMR spectroscopy, with free *p*-cymene being observed.^59^ The addition of a substoichiometric amount of *p*-cymene to the reaction mixture inhibited catalysis.^64^ Additionally, a quantum yield^64^ of 1.2% was in good agreement. Finally, UV-spectroscopy allowed us to identify that both the ruthenium complex and ruthenacycle Ru-1 show a strong absorbance of light in the range between 380–460 nm. Analysis of the reaction mixture before and after irradiation with blue light also show the formation of new species (Scheme 3C).^64^

**Scheme 4:**
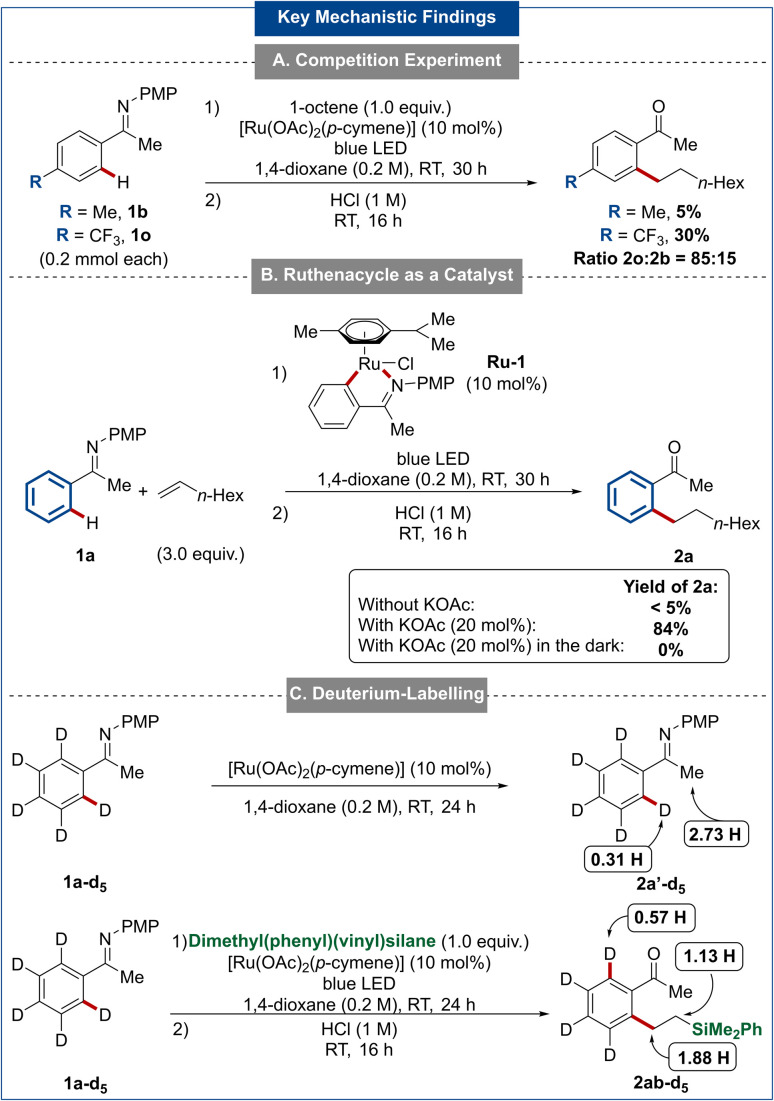
Key mechanistic findings.

To gain further insights into the potential mode of action, an intermolecular competition experiment was performed between electron-rich ketimine 1b and electron-deficient ketimine 1o (Scheme 4A). After 24 h, the trifluoromethyl-substituted compound 2o was obtained in 30% yield against 5% for the methyl-substituted product 2b. Consequently, the C–H activation may occur preferentially on electron-poor arenes with weaker C–H bonds indicating a carboxylate-assisted cycloruthenation. This tendency was also observed during the evaluation of the substrate scope of the ketimine 1, as slightly higher yields were obtained in the case of electron-poor ketimines (Scheme 2). To pursue our studies, the independently synthesized ruthenacycle Ru-1 was employed as catalyst under our standard conditions. Notably, this complex was only effective in the presence of a catalytic amount of potassium acetate (20 mol%), further substantiating that a carboxylate-assisted ruthenation is involved in the catalytic cycle (Scheme 4B). Interestingly, product formation was not observed in the dark with complex Ru-1 as catalyst, being suggestive of light-independent cyclometallation, along with a light-induced activation – arguably through decoordination of the *p*-cymene ligand.^64^ Next, deuterium-labelling experiments were carried out. When ketimine 1a-d_5_ was treated with [Ru(OAc)_2_(*p*-cymene)] under blue light irradiation, a significant H/D exchange was observed (Scheme 4C). This result points to the reversibility of the cleavage of the *ortho* C–H bond. The hydroarylation reaction between 1a-d_5_ and dimethyl(phenyl)(vinyl)silane was also examined. Deuterium incorporation was only observed at the β-position of the hydroarylation product 2ac-d_5_ while no deuterium was introduced at the α-position (Scheme 4C). This observation is in contrast to rhodium(i)-catalyzed alkylations of aromatic amides reported by Chatani whose mechanistic proposal was considered to undergo a carbene mechanism which was confirmed by deuterium labelling experiments and DFT calculations.^68^ The carbene mechanism was therefore considered unlikely for the photo-induced hydroarylation.

**Scheme 5:**
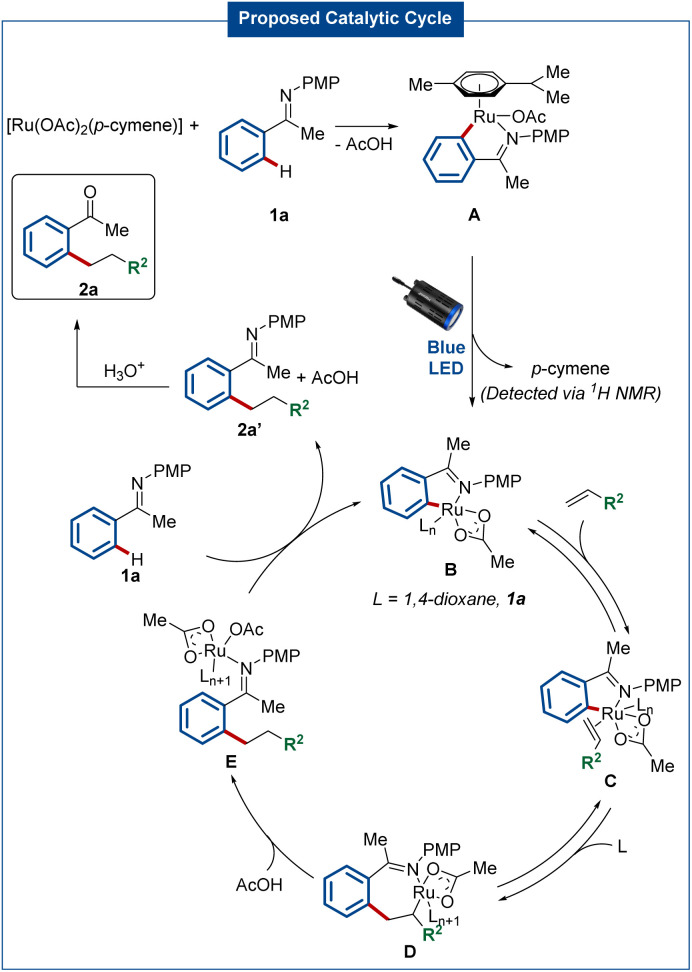
Proposed catalytic cycle.

Based on our experiments and literature precedents,^59–61^ a plausible mechanism for the photo-enabled hydroarylation of olefins involves an initial C–H ruthenation of ketimine 1 to generate ruthenacycle A (Scheme 5). After light-induced *p*-cymene dissociation, the catalytic active species B is formed. Subsequently, the coordination of the olefin *via* intermediate C, along with migratory insertion into the Ru–C bond forms intermediate D. Then, acetic acid enables proto-demetallation to generate the species E. Finally, ligand exchange with another equivalent of the starting material releases the desired product 2a′, while regenerating catalyst B through cyclometalation.

## Conclusions

In summary, we reported on versatile C–H alkylations enabled by photo-induced ruthenium(ii)-catalyzed hydroarylation of non-activated alkenes. In sharp contrast to established catalytic systems, the carboxylate-assisted ruthenium(ii) catalysis proved efficient under exceedingly mild reaction conditions, namely at ambient temperature, enabled by visible light irradiation. The ruthenaphotocatalysis was easily conducted on gram-scale and its robustness was mirrored by a broad functional group tolerance.

## Data availability

The data supporting this article have been uploaded as part of the ESI.†

## Author contributions

Conceptualization, L. A.; funding acquisition, L. A.; investigation S. T., S. G., M. H. G., M. D. M., methodology, S. T., S. G.; resources, L. A., supervision, L. A.; writing – original draft, S. T., S. G., L. A., writing – reviewing & editing, all authors.

## Conflicts of interest

There are no conflicts to declare.

## Supplementary Material

SC-OLF-D4SC06005A-s001
