# Supplementary material for: Visible light-induced ruthenium(ii)-catalyzed hydroarylation of unactivated olefins
Source: Chem Sci. 2024 Oct 23;15(45):19037–43. doi: 10.1039/d4sc06005a (PMC11514381; doi:10.1039/d4sc06005a)

Supplementary Information (SI) for Chemical Science.
This journal is © The Royal Society of Chemistry 2024

Supporting Information

Visible Light-Induced Ruthenium(II)-Catalyzed Hydroarylation of Unactivated Olefins

Sven Trienes,[a,b,‡] Stéphane Golling,[a,‡] Matthew H. Gieuw,[a] Marco Di Matteo,[a] and Lutz Ackermann[a,b]*

[a] Wöhler Research Institute for Sustainable Chemistry (WISCh), Georg-August-Universität, Tammannstraße 2, 37077 Göttingen (Germany)

[b] DZHK (German Centre for Cardiovascular Research), Potsdamer Straße 58, 10875 Berlin (Germany)

E-mail: Lutz.Ackermann@chemie.uni-goettingen.de

https://www.ackermann.chemie.uni-goettingen.de/

Contents

Supporting Information 1
1 General Remarks 3
2 Synthesis of Starting Material 4
2.1 Imine Synthesis 4
2.2 Alkene Synthesis 9
3 Optimization Studies 12
4 Visible Light-Induced Ruthenium-Catalyzed Hydroarylation of Olefins 16
5 Mechanistic Studies 33
5.1 Detection of Free *p*-Cymene 33
5.2 Inhibition Experiment with *p*-Cymene 39
5.3 Sequential Experiments 40
5.4 On-Off Experiment 42
5.5 UV-Vis Absorption Measurements 43
5.6 Intermolecular Competition Experiment 45
5.7 Ruthenacycle Ru-1 as Catalyst 46
5.8 Deuterium-Labelling Experiment 47
5.9 Determination of Quantum Yield 49
6 References 51
7 NMR Spectra 52

1 General Remarks

General Conditions: Catalytic reactions were performed under a N_2 atmosphere using pre-dried glassware. 1,4-Dioxane was dried over sodium, distilled under N_2, degassed by several cycles of freeze-pump-thaw-method, and was stored in a N_2-filled glovebox over 4 Å molecular sieves. Solvents for column chromatography (EtOAc, hexane, CH_2Cl_2) were distilled prior to use. The reaction temperature for photochemical reactions was measured by a digital thermometer PCE-T 390, which was determined to be in the range of 30–35 °C. The substrates were either purchased directly from commercial suppliers or prepared according to previously reported procedures, if not noted otherwise. All other reagents and solvents used in this study were purchased from commercial sources and used as received.

Chromatographic Methods: Routine TLC was performed on Merck TLC Silica Gel 60 F_{254} with detection under UV light at 254 nm. Chromatographic separations were carried out on Merck Geduran SI-60 (40–63 μm, 230–400 mesh ASTM).

IR Spectroscopy: IR spectra were recorded on a Bruker FT-IR *alpha-P* device.

Mass Spectrometry: ESI-MS was recorded on Bruker Daltonik *micrOTOF* and *maXis*. The ratios of mass to charge (m/z) are reported and the intensity relative to the base peak (I = 100) is given in parentheses.

Melting Point: Melting points (m.p.) were measured on Stuart® melting point apparatus *SMP3*, values are uncorrected.

Spectroscopic Methods: Nuclear magnetic resonance (NMR) spectroscopy was performed at 400 MHz, 300 MHz (^{1}H NMR), 126 MHz, 101 MHz (^{13}C NMR, APT), and 282 MHz (^{19}F NMR) on Bruker *Avance Neo 300*, *Avance III HD 300,* and *Avance Neo 400* instruments. Chemical shifts (δ) are provided in ppm and spectra referred to non-deuterated solvent signal.

2 Synthesis of Starting Material

2.1 Imine Synthesis

The following imines were synthesized according to literature protocols which are described in more detail in the following. Compound **S3** was synthesized following the procedure reported by Zhang et al. and matches the reported spectroscopic data.[1]

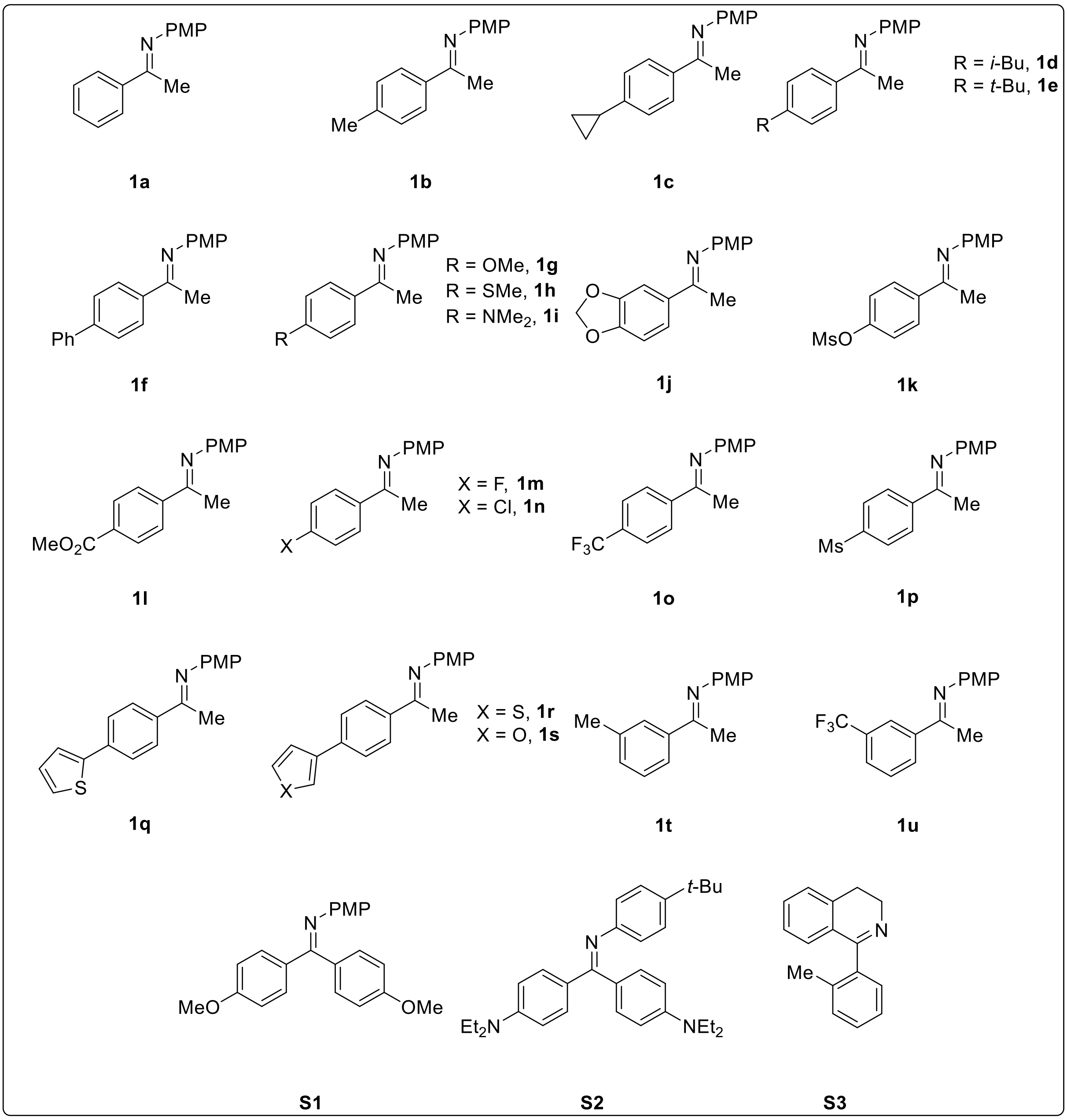

Reaction Schemes for Imine-Synthesis:

General Procedure A:

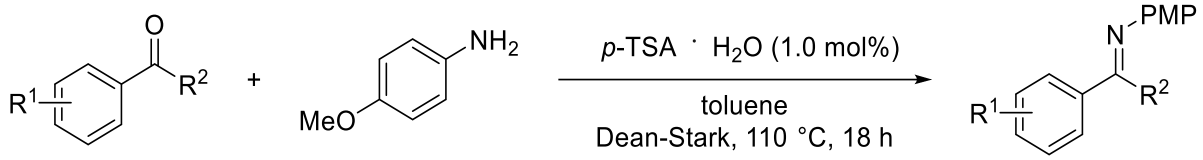

This procedure follows a slightly modified method reported by Martin et al.[2]

A round-bottom was charged with ketone (10 mmol, 1.0 equiv.), *p*-anisidine (10 mmol, 1.0 equiv.), *p*-toluene sulfonic acid monohydrate (0.1 mmol, 1 mol%) and toluene (40 mL). The resulting reaction mixture was refluxed at 110 °C under Dean-Stark conditions for 18 h. After cooling down to ambient temperature, the volatiles were removed under reduced pressure. The crude product was subsequently purified by recrystallization.

General Procedure B:

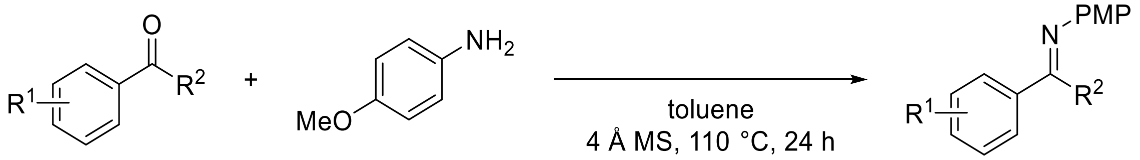

This procedure follows a slightly modified method reported Xiao et al.[3]

A round-bottom Schlenk flask equipped with reflux condenser was charged with the ketone (10 mmol, 1.0 equiv.), *p*-anisidine (10 mmol, 1.0 equiv.), 4 Å molecular sieves (3.75 g) and toluene (40 mL). The resulting reaction mixture was stirred for 24 h at 110 °C. After cooling down to ambient temperature, the reaction was filtered through a short pad of celite which was washed with EtOAc afterwards. Volatiles were removed under reduced pressure and the residue was purified either by recrystallization or column chromatography on silica gel.

The following compounds are known and were synthesized according to the described procedures. Analytical data match with those reported in the literature.

(*E*)-*N*-(4-Methoxyphenyl)-1-phenylethan-1-imine (1a): General procedure A.[4]

(*E*)-*N*-(4-Methoxyphenyl)-1-(*p*-tolyl)ethan-1-imine (1b): General procedure A.[4]

(*E*)-1-(4-Isobutylphenyl)-*N*-(4-methoxyphenyl)ethan-1-imine (1d): General procedure B.[4]

(*E*)-1-(4-(*tert*-Butyl)phenyl)-*N*-(4-methoxyphenyl)ethan-1-imine (1e): General procedure B.[5]

(*E*)-1-([1,1'-Biphenyl]-4-yl)-*N*-(4-methoxyphenyl)ethan-1-imine (1f): General procedure B.[4]

(*E*)-*N*,1-Bis(4-methoxyphenyl)ethan-1-imine (1g): General procedure A.[4]

(*E*)-1-(Benzo[*d*][1,3]dioxol-5-yl)-*N*-(4-methoxyphenyl)ethan-1-imine (1j): General procedure B.[4]

Methyl (*E*)-4-(1-((4-methoxyphenyl)imino)ethyl)benzoate (1l): General procedure A.[4]

(*E*)-1-(4-Fluorophenyl)-*N*-(4-methoxyphenyl)ethan-1-imine (1m): General procedure B.[4]

(*E*)-1-(4-Chlorophenyl)-*N*-(4-methoxyphenyl)ethan-1-imine (1n): General procedure A.[4]

(*E*)-*N*-(4-Methoxyphenyl)-1-(4-(trifluoromethyl)phenyl)ethan-1-imine (1o): General procedure A.[4]

(*E*)-*N*-(4-Methoxyphenyl)-1-(*m*-tolyl)ethan-1-imine (1t): General procedure B.[4]

(*E*)-*N*-(4-Methoxyphenyl)-1-(3-(trifluoromethyl)phenyl)ethan-1-imine (1u): General procedure B.[4]

The following compounds are not known and were synthesized through the described methods:

(*E*)-1-(4-Cyclopropylphenyl)-*N*-(4-methoxyphenyl)ethan-1-imine (1c)

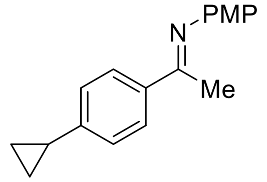

1c was prepared according to general procedure B.

^{1}H NMR (300 MHz, $CDCl_3$): δ 7.86 (d, *J* = 8.4 Hz, 2H), 7.12 (d, *J* = 8.3 Hz, 2H), 6.90 (d, *J* = 8.8 Hz, 2H), 6.74 (d, *J* = 8.8 Hz, 2H), 3.82 (s, 3H), 2.22 (s, 3H), 1.94 (tt, *J* = 8.4, 5.0 Hz, 1H), 1.08 – 0.97 (m, 2H), 0.80 – 0.72 (m, 2H) ppm.

^{13}C NMR (75 MHz, $CDCl_3$): δ 165.6 (C_q), 155.9 (C_q), 147.0 (C_q), 145.1 (C_q), 137.1 (C_q), 127.2 (CH), 125.5 (CH), 120.9 (CH), 114.3 (CH), 55.6 (CH_3), 17.3 (CH_3), 15.6 (CH), 10.0 (CH_2) ppm.

M.p.: 101 °C.

IR (ATR): $\tilde{\nu}$ = 3003, 1626, 1606, 1503, 1286, 1244, 1209, 1035, 848, 826 cm^{-1}.

MS (ESI) *m/z* (relative intensity): 553 (20) $[2M+Na]^+$, 288 (10) $[M+Na]^+$, 266 (100) $[M+H]^+$

HRMS (ESI): *m/z* calcd. for $C_{18}H_{20}NO^+$ $[M+H]^+$ 266.1539, found 266.1541.

(*E*)-*N*-(4-Methoxyphenyl)-1-(4-(methylthio)phenyl)ethan-1-imine (1h)

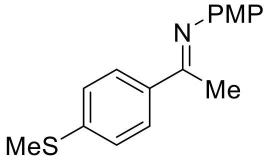

1h was prepared according to general procedure A.

^{1}H NMR (400 MHz, $CDCl_3$): δ 7.89 (d, *J* = 8.7 Hz, 2H), 7.28 (d, *J* = 8.6 Hz, 2H), 6.91 (d, *J* = 8.9 Hz, 2H), 6.74 (d, *J* = 8.9 Hz, 2H), 3.82 (s, 3H), 2.52 (s, 3H), 2.22 (s, 3H) ppm.

^{13}C NMR (101 MHz, $CDCl_3$): δ 165.0 (C_q), 156.1 (C_q), 144.9 (C_q), 141.8 (C_q), 136.4 (C_q), 127.6 (CH), 125.7 (CH), 121.0 (CH), 114.4 (CH), 55.6 (CH_3), 17.2 (CH_3), 15.4 (CH_3) ppm.

M.p.: 131 °C.

IR (ATR): $\tilde{\nu}$ = 2834, 1621, 1503, 1282, 1241, 1209, 1102, 1032, 845, 820 cm^{-1}.

MS (ESI) *m/z* (relative intensity): 565 (15) $[2M+Na]^+$, 294 (15) $[M+Na]^+$, 272 (100) $[M+H]^+$.

HRMS (ESI): *m/z* calcd. for $C_{16}H_{18}NOS^+$ $[M+H]^+$ 272.1104, found 272.1107.

(*E*)-4-(1-((4-Methoxyphenyl)imino)ethyl)-*N*,*N*-dimethylaniline (1i)

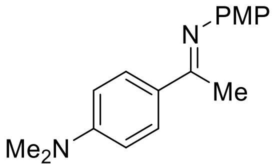

1i was prepared according to general procedure B.

^{1}H NMR (400 MHz, $CDCl_3$): δ 7.89 (d, *J* = 9.0 Hz, 2H), 6.89 (d, *J* = 8.8 Hz, 2H), 6.73 (dd, *J* = 9.0, 7.8 Hz, 4H), 3.81 (s, 3H), 3.03 (s, 6H), 2.19 (s, 3H) ppm.

^{13}C NMR (101 MHz, $CDCl_3$): δ 165.1 (C_q), 155.7 (C_q), 152.0 (C_q), 145.7 (C_q), 128.6 (CH), 127.7 (C_q), 121.2 (CH), 114.3 (CH), 111.5 (CH), 55.6 (CH_3), 40.4 (CH_3), 17.0 (CH_3) ppm.

M.p.: 163 °C.

IR (ATR): $\tilde{\nu}$ = 1610, 1525, 1503, 1372, 1243, 1035, 908, 839, 817, 731 cm^{-1}.

MS (ESI) *m*/*z* (relative intensity): 559 (6) $[2M+Na]^+$, 291 (8) $[M+Na]^+$, 269 (100) $[M+H]^+$.

HRMS (ESI): *m*/*z* calcd. for $C_{17}H_{21}N_2O$ $[M+H]^+$ 269.1648, found 269.1649.

(*E*)-4-(1-((4-Methoxyphenyl)imino)ethyl)phenyl methanesulfonate (1k)

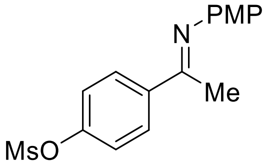

1k was prepared according to general procedure B.

^{1}H NMR (300 MHz, $CDCl_3$): δ 8.03 (d, *J* = 8.8 Hz, 2H), 7.35 (d, *J* = 8.8 Hz, 2H), 6.92 (d, *J* = 8.8 Hz, 2H), 6.74 (d, *J* = 8.9 Hz, 2H), 3.82 (s, 3H), 3.17 (s, 3H), 2.25 (s, 3H) ppm.

^{13}C NMR (75 MHz, $CDCl_3$): δ 164.3 (C_q), 156.3 (C_q), 150.7 (C_q), 144.4 (C_q), 139.0 (C_q), 129.1 (CH), 121.9 (CH), 120.9 (CH), 114.4 (CH), 55.6 (CH_3), 37.7 (CH_3), 17.4 (CH_3) ppm.

M.p.: 127 °C.

IR (ATR): $\tilde{\nu}$ = 1632, 1604, 1503, 1367, 1241, 1175, 1150, 869, 845 cm^{-1}.

MS (ESI) *m*/*z* (relative intensity): 661 (13) $[2M+Na]^+$, 639 (3) $[2M+H]^+$, 342 (16) $[M+Na]^+$, 320 (100) $[M+H]^+$.

HRMS (ESI): *m*/*z* calcd. for $C_{16}H_{18}NO_4S^+$ $[M+H]^+$ 320.0951, found 320.0948.

(*E*)-*N*-(4-Methoxyphenyl)-1-(4-(methylsulfonyl)phenyl)ethan-1-imine (1p)

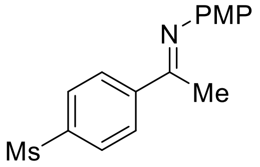

1p was prepared according to general procedure B.

^{1}H NMR (300 MHz, $CDCl_3$): δ 8.15 (d, *J* = 8.7 Hz, 2H), 8.01 (d, *J* = 8.7 Hz, 2H), 6.93 (d, *J* = 8.9 Hz, 2H), 6.77 (d, *J* = 8.8 Hz, 2H), 3.83 (s, 3H), 3.08 (s, 3H), 2.31 (s, 3H) ppm.

^{13}C NMR (75 MHz, $CDCl_3$): δ 164.1 (C_q), 156.6 (C_q), 144.8 (C_q), 144.0 (C_q), 141.7 (C_q), 128.2 (CH), 127.6 (CH), 120.9 (CH), 114.5 (CH), 55.6 (CH_3), 44.7 (CH_3), 17.6 (CH_3) ppm.

M.p.: 156 °C.

IR (ATR): $\tilde{\nu}$ = 1624, 1502, 1312, 1296, 1240, 1150, 1034, 850, 785, 760 cm^{-1}.

MS (ESI) *m*/*z* (relative intensity): 629 (67) $[2M+Na]^+$, 607 (10) $[2M+H]^+$, 326 (45) $[M+Na]^+$, 304 (100) $[M+H]^+$.

HRMS (ESI): *m/z* calcd. for $C_{16}H_{18}NO_3S^+$ $[M+H]^+$ 304.1002, found 304.1004.

(*E*)-*N*-(4-Methoxyphenyl)-1-(4-(thiophen-2-yl)phenyl)ethan-1-imine (1q)

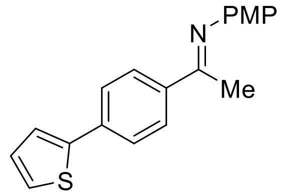

1q was prepared according to general procedure B.

^{1}H NMR (300 MHz, $CDCl_3$): δ 7.98 (d, *J* = 8.8 Hz, 2H), 7.68 (d, *J* = 8.8 Hz, 2H), 7.41 (dd, *J* = 3.6, 1.2 Hz, 1H), 7.33 (dd, *J* = 5.1, 1.1 Hz, 1H), 7.11 (dd, *J* = 5.1, 3.6 Hz, 1H), 6.92 (d, *J* = 8.8 Hz, 2H), 6.77 (d, *J* = 8.8 Hz, 2H), 3.83 (s, 3H), 2.27 (s, 3H) ppm.

^{13}C NMR (75 MHz, $CDCl_3$): δ 165.1 (C_q), 156.1 (C_q), 144.9 (C_q), 143.8 (C_q), 138.7 (C_q), 136.3 (C_q), 128.3 (CH), 127.9 (CH), 125.8 (CH), 125.7 (CH), 123.9 (CH), 121.0 (CH), 114.4 (CH), 55.6 (CH_3), 17.4 (CH_3) ppm.

M.p.: 179 °C.

IR (ATR): $\tilde{\nu}$ = 3004, 2947, 2832, 1622, 1605, 1505, 1287, 1245, 850, 705 cm^{-1}.

MS (ESI) *m/z* (relative intensity): 637 (8) $[2M+Na]^+$, 330 (5) $[M+Na]^+$, 308 (100) $[M+Na]^+$.

HRMS (ESI): *m/z* calcd. for $C_{19}H_{18}NOS^+$ $[M+H]^+$ 308.1104, found 308.1106.

(*E*)-*N*-(4-Methoxyphenyl)-1-(4-(thiophen-3-yl)phenyl)ethan-1-imine (1r)

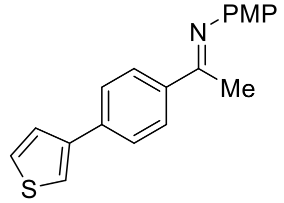

1s was prepared according to general procedure B.

^{1}H NMR (300 MHz, $CDCl_3$): δ 8.01 (d, *J* = 8.7 Hz, 2H), 7.67 (d, *J* = 8.7 Hz, 2H), 7.55 (dd, *J* = 2.8, 1.5 Hz, 1H), 7.49 – 7.39 (m, 2H), 6.92 (d, *J* = 8.8 Hz, 2H), 6.77 (d, *J* = 8.8 Hz, 2H), 3.83 (s, 3H), 2.27 (s, 3H) ppm.

^{13}C NMR (75 MHz, $CDCl_3$): δ 165.3 (C_q), 156.1 (C_q), 144.9 (C_q), 141.8 (C_q), 138.5 (C_q), 137.7 (C_q), 127.8 (CH), 126.6 (CH), 126.4 (CH), 126.4 (CH), 121.2 (CH), 121.0 (CH), 114.4 (CH), 55.6 (CH_3), 17.4 (CH_3) ppm.

IR (ATR): $\tilde{\nu}$ = 2833, 1620, 1606, 1507, 1285, 1248, 1033, 841, 782, 730 cm^{-1}.

MS (ESI) *m/z* (relative intensity): 637 (6) $[2M+Na]^+$, 330 (3) $[M+Na]^+$, 308 (100) $[M+H]^+$.

HRMS (ESI): *m/z* calcd. for $C_{19}H_{18}NOS^+$ $[M+H]^+$ 308.1104, found 308.1105.

(*E*)-1-(4-(Furan-3-yl)phenyl)-*N*-(4-methoxyphenyl)ethan-1-imine (1s)

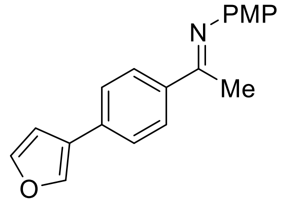

1r was prepared according to general procedure B.

^{1}H NMR (400 MHz, $CDCl_3$): δ 7.99 (d, *J* = 8.5 Hz, 2H), 7.81 (dd, *J* = 1.5, 0.9 Hz, 1H), 7.56 (d, *J* = 8.6 Hz, 2H), 7.51 (t, *J* = 1.7 Hz, 1H), 6.93 (d, *J* = 8.8 Hz, 2H), 6.82 – 6.74 (m, 3H), 3.83 (s, 3H), 2.26 (s, 3H) ppm.

^{13}C NMR (101 MHz, $CDCl_3$): δ 165.2 (C_q), 156.1 (C_q), 144.9 (C_q), 144.0 (CH), 139.2 (CH), 138.4 (C_q), 134.5 (C_q), 127.8 (CH), 126.1 (CH), 125.7 (CH), 120.9 (CH), 114.4 (CH), 108.9 (CH), 55.6 (CH_3), 17.3 (CH_3) ppm.

M.p.: 144 °C.

IR (ATR): $\tilde{\nu}$ = 2834, 1680, 1607, 1510, 1364, 1237, 1163, 1034, 788, 735 cm^{-1}.

MS (ESI) *m/z* (relative intensity): 605 (15) $[2M+Na]^+$, 314 (15) $[M+Na]^+$, 292 (100) $[M+H]^+$.

HRMS (ESI): *m/z* calcd. for $C_{19}H_{18}NO_2^+$ $[M+H]^+$ 292.1332, found 292.1334.

***N*,1,1-Tris(4-methoxyphenyl)methanimine (S1)**

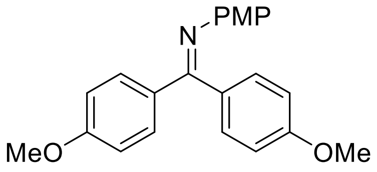

3a was prepared according to general procedure A.

^{1}H NMR (400 MHz, $CDCl_3$): 7.67 (d, J = 8.9 Hz, 2H), 7.04 (d, J = 8.8 Hz, 2H), 6.90 (d, J = 8.9 Hz, 2H), 6.78 (d, J = 8.8 Hz, 2H), 6.73 – 6.63 (m, 4H), 3.85 (s, 3H), 3.79 (s, 3H), 3.74 (s, 3H) ppm.

^{13}C NMR (101 MHz, $CDCl_3$): δ 167.2 (C_q), 161.7 (C_q), 159.6 (C_q), 155.7 (C_q), 145.1 (C_q), 133.4 (C_q), 131.5 (CH), 131.1 (CH), 129.0 (C_q), 122.8 (CH), 113.9 (CH), 113.6 (CH), 113.5 (CH), 55.6 (CH_3), 55.5 (CH_3), 55.3 (CH_3) ppm.

M.p.: 118 °C.

IR (ATR): $\tilde{\nu}$ = 2835, 1606, 1571, 1501, 1464, 1247, 1169, 1033, 839, 751 cm^{-1}.

MS (ESI) *m/z* (relative intensity): 1064 (6) $[3M+Na]^+$, 717 (10) $[2M+Na]^+$, 370 (8) $[M+Na]^+$, 348 (100) $[M+H]^+$.

HRMS (ESI): *m/z* calcd. for $C_{22}H_{22}NO_3^+$ $[M+H]^+$ 348.1594, found 348.1594.

4,4'-(((4-(*tert*-Butyl)phenyl)imino)methylene)bis(*N*,*N*-diethylaniline) (S2)

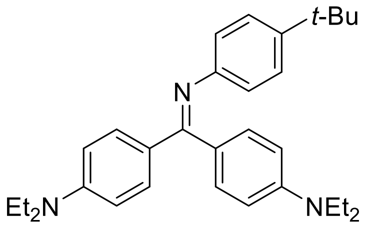

3b was prepared according to general procedure A.

^{1}H NMR (300 MHz, $CDCl_3$): δ 7.64 (d, J = 9.0 Hz, 2H), 7.15 (d, J = 8.5 Hz, 2H), 7.01 (d, J = 8.8 Hz, 2H), 6.70 (d, J = 8.5 Hz, 2H), 6.64 (d, J = 9.0 Hz, 2H), 6.47 (d, J = 8.9 Hz, 2H), 3.40 (q, J = 7.4 Hz, 4H), 3.33 (q, J = 7.2 Hz, 4H), 1.26 (s, 9H), 1.21 – 1.12 (m, 12H) ppm.

M.p.: 133 °C.

^{13}C NMR (75 MHz, $CDCl_3$): δ 167.8 (C_q), 150.2 (C_q), 149.3 (C_q), 147.7 (C_q), 144.6 (C_q), 132.2 (CH), 131.5 (CH), 128.1 (C_q), 125.3 (CH), 123.3 (C_q), 121.4 (CH), 110.6 (CH), 110.3 (CH), 44.6 (CH_2), 44.3 (CH_2), 34.3 (C_q), 31.6 (CH_3), 12.8 (CH_3), 12.7 (CH_3) ppm.

IR (ATR): $\tilde{\nu}$ = 2965, 2900, 1604, 1574, 1518, 1264, 1188, 1139, 822, 731 cm^{-1}.

MS (ESI) *m/z* (relative intensity): 911 (7) $[2M+H]^+$, 456 (100) $[M+H]^+$.

HRMS (ESI): *m/z* calcd. for $C_{31}H_{42}N_3^+$ $[M+H]^+$ 456.3373, found 456.3376.

2.2 Alkene Synthesis

The following terminal alkenes were synthesized according to literature protocols which are described in more detail in the following. The Ibuprofen-derived terminal alkene **S4** was synthesized following the procedure reported by Wu et al. and matches the reported spectroscopic data.[6]

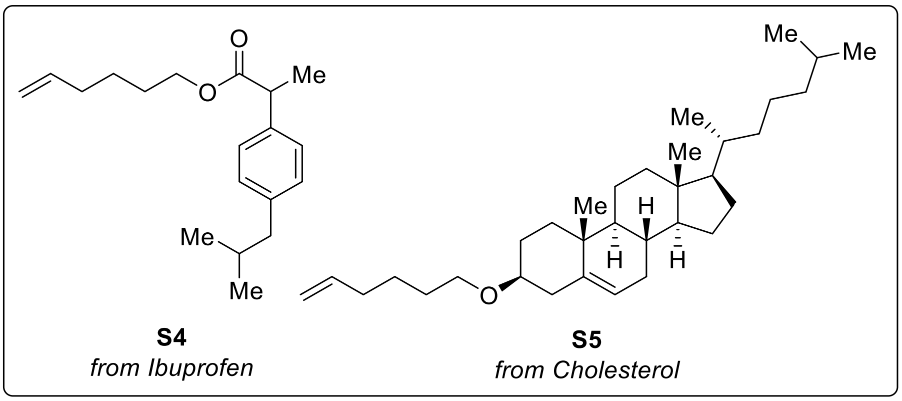

S4
from Ibuprofen

S5
from Cholesterol

General Procedure C:

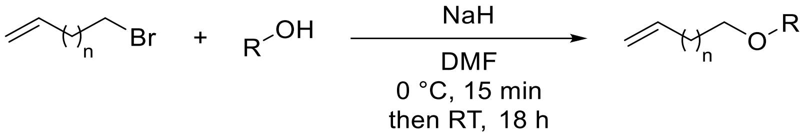

An oven-dried 50 mL flask containing a stirring bar was charged with the corresponding alcohol (1.0 equiv.) and dry DMF (0.2 M). At 0 °C, NaH (1.1 equiv., 60% in mineral oil) was added slowly. The reaction mixture has been stirred at ambient temperature for 15 min until H_2-evolution ceased. Then, the corresponding bromoalkene (1.1 – 1.3 equiv.) was added dropwise and the reaction was stirred at room temperature for 18 h. The reaction mixture was quenched by the addition of saturated NH_4Cl at 0 °C. After extraction with CH_2Cl_2, combined organic phases were dried over $MgSO_4$ and the solvent was removed under reduced pressure. Column chromatography of the residue afforded the corresponding product.

The following compounds are new and were synthesized through the described method:

(3*S*,8*S*,9*S*,10*R*,13*R*,14*S*,17*R*)-3-(Hex-5-en-1-yloxy)-10,13-dimethyl-17-((*R*)-6-methylheptan-2-yl)-2,3,4,7,8,9,10,11,12,13,14,15,16,17-tetradecahydro-1*H*-cyclopenta[*a*]phenanthrene (S5)

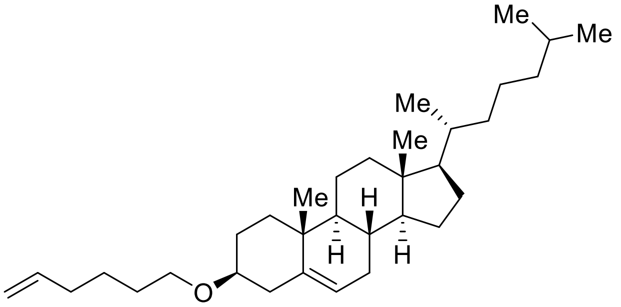

The title compound was prepared according to the general procedure C.

^{1}H NMR (400 MHz, $CDCl_3$): δ 5.81 (ddt, *J* = 16.9, 10.2, 6.7 Hz, 2H), 5.34 (dd, *J* = 5.1, 2.4 Hz, 1H), 5.08 – 4.90 (m, 4H), 3.50 – 3.37 (m, 4H), 3.12 (tt, *J* = 11.4, 4.4 Hz, 1H), 2.35 (ddd, *J* = 13.2, 4.8, 2.3 Hz, 1H), 2.24 – 2.11 (m, 1H), 2.11 – 2.03 (m, 4H), 2.01 – 1.78 (m, 3H), 1.64 – 1.51 (m, 6H), 1.46 (dddd, *J* = 14.5, 6.8, 4.1, 2.3 Hz, 6H), 1.39 – 1.23 (m, 4H), 1.18 – 0.99 (m, 4H), 1.00 (s, 3H), 0.91 (d, *J* = 6.6 Hz, 3H), 0.87 (d, *J* = 1.8 Hz, 3H), 0.85 (d, *J* = 1.8 Hz, 3H), 0.67 (s, 3H) ppm.

^{13}C NMR (101 MHz, $CDCl_3$): δ 141.3 (C_q), 139.0 (CH), 121.6 (CH), 114.6 (CH_2), 79.1 (CH) 68.0 (CH_2), 57.0 (CH), 56.3 (CH), 50.4 (CH), 42.5 (C_q), 40.0 (CH), 39.7 (CH_2), 39.4 (CH_2), 37.5 (CH_2), 37.1 (C_q), 36.3 (CH_2), 35.9 (CH), 33.8 (CH_2), 32.1 (CH_2), 32.1 (CH), 29.8 (CH_2), 28.6 (CH_2), 28.4 (CH_2), 28.2 (CH_2), 25.7 (CH_2), 24.5 (CH_2), 24.0 (CH_3), 23.0 (CH_3), 22.7 (CH_2), 21.2 (CH_2), 19.5 (CH_3), 18.9 (CH_3), 12.0 (CH_3).

IR (ATR): $\tilde{\nu}$ = 2932, 2867, 1767, 1640, 1465, 1376, 1343, 1107, 908 cm^{-1}.

MS (ESI) *m*/*z* (relative intensity): 491 (100) $[M+Na]^+$.

HRMS (ESI): *m*/*z* calcd. for $C_{33}H_{56}ONa^+$ $[M+Na]^+$ 491.4211, found 491.4223.

3 Optimization Studies

Table S1. Optimization of ruthenium-catalyzed hydroarylation probing different Ru catalysts.[a]

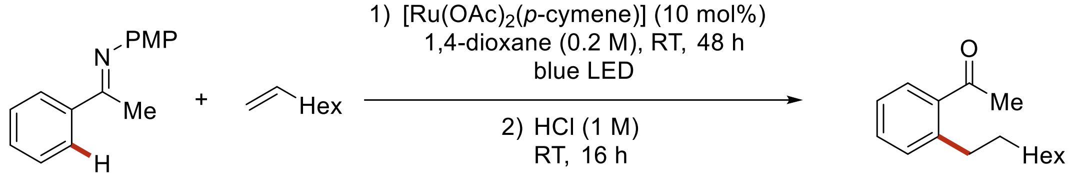

1a **2a**

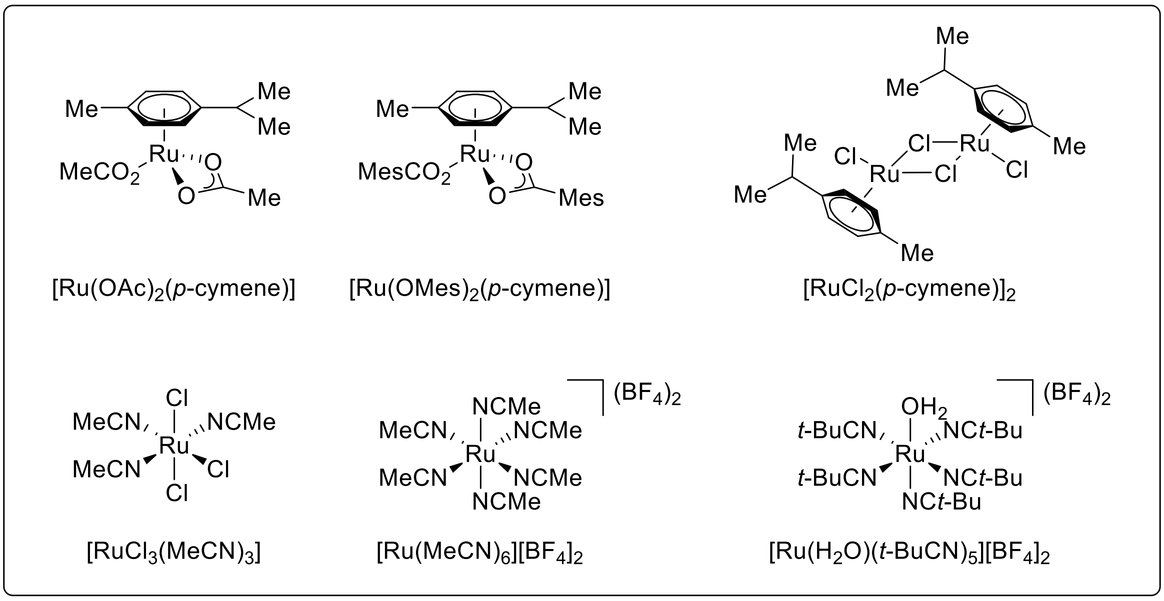

Entry	Deviations from the conditions above	Yield (%)[a]
1	None	85
2	$RuCl_3{\cdot}3H_2O$ as catalyst	0
3	$Ru_3(CO)_{12}$ as catalyst	0
4	$[Ru(O_2CMes)_2(p\text{-cymene})]$ as catalyst	69
5	$[RuCl_2(p\text{-cymene})]_2$ as catalyst	32
6	$[RuCl_2(p\text{-cymene})]_2$ + PPh_3 (10 mol%) as catalyst	43
7[b,c]	$[RuCl_3(MeCN)_3]$ as catalyst + KOAc (20 mol%)	0
8[b,c]	$[RuCl_3(MeCN)_3]$ as catalyst + KOAc (20 mol%) in MeCN	0
9[b,c]	$[RuCl_3(MeCN)_3]$ as catalyst + KOAc (20 mol%) in PEG400	0
10[b,c]	$[Ru(MeCN)_6][BF_4]_2$ as catalyst + KOAc (20 mol%)	0
11[b,c]	$[Ru(MeCN)_6][BF_4]_2$ as catalyst + KOAc (20 mol%) in MeCN	0
12[b,c]	$[Ru(MeCN)_6][BF_4]_2$ as catalyst + KOAc (20 mol%) in PEG400	0
13	$[Ru(H_2O)(t\text{-BuCN})_5]$ as catalyst + KOAc (20 mol%)	(6)
14[c]	$[Ru(H_2O)(t\text{-BuCN})_5]$ as catalyst + KOAc (20 mol%)	0
15[d]	$[Ru(H_2O)(t\text{-BuCN})_5]$ as catalyst + KOAc (20 mol%)	0
16[e]	$[Ru(H_2O)(t\text{-BuCN})_5]$ as catalyst + KOAc (20 mol%)	(18)
17[e]	$[Ru(H_2O)(t\text{-BuCN})_5]$ as catalyst + K_2CO_3 (2.0 equiv.)	0

[a] Reaction conditions: **1a** (0.2 mmol), 1-octene (0.6 mmol**)**, [Ru(OAc)$_2$(*p*-cymene)] (10 mol%), 1,4-dioxane (1.0 mL), 30–35°C, 48 h, under N_2, blue LEDs. Yields of isolated product **2a**, in parentheses yields determined by ^{1}H-NMR using 1,3,5-trimethoxybenzene as the internal standard. [b] 24 h. [c] 30 °C, in the dark. [d] 40 °C, in the dark. [e] 50 °C, in the dark.

Table S2. Optimization of ruthenium-catalyzed hydroarylation probing different Ru catalysts.[a]

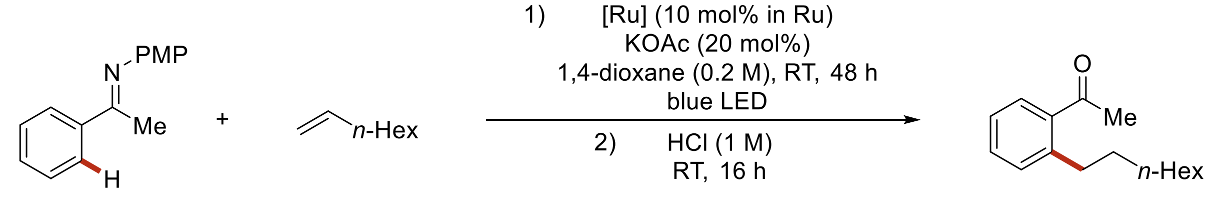

1a **2a**

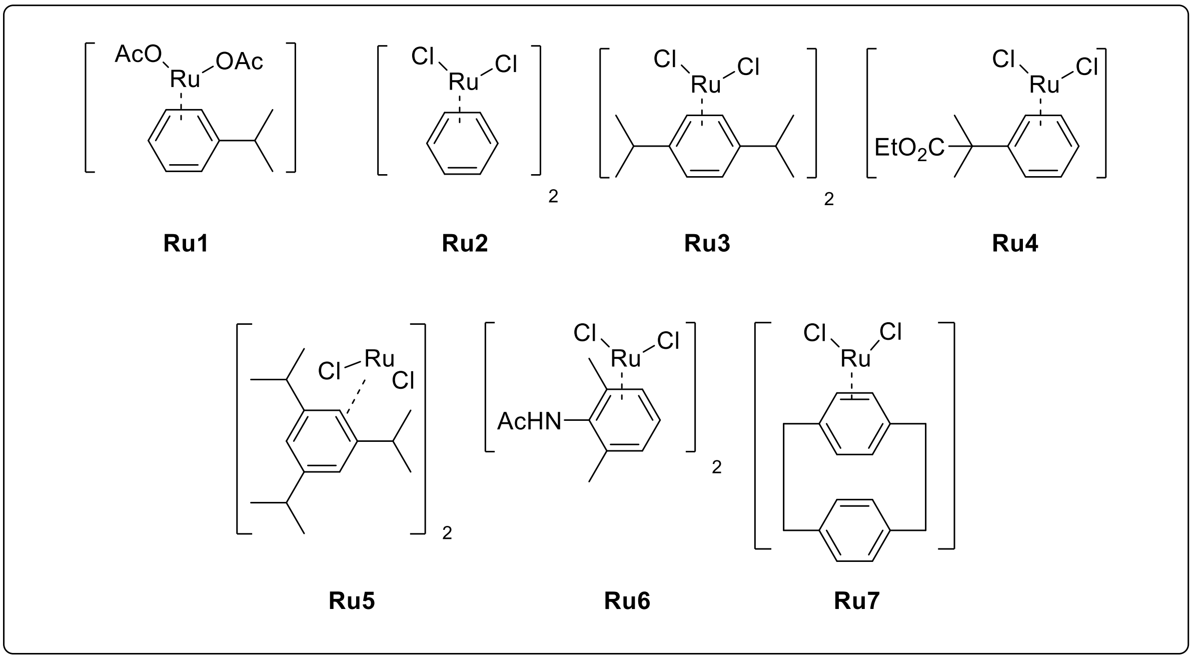

Entry	[Ru] Complex	Yield (%)[a]	Comments
**1**	Ru1	85%[b]	No KOAc added
**2**	Ru2	(68%)	-
**3**	Ru3	(85%)	-
**4**	Ru4	(85%)	-
**5**	Ru5	(63%)	-
**6**	Ru6	(83%)	-
**7**	Ru7	(68%)	-

[a] Reaction conditions: **1a** (0.2 mmol), 1-octene (0.6 mmol**)**, [Ru] (5 or 10 mol%), KOAc (20 mol%), 1,4-dioxane (1.0 mL), 30–35 °C, 48 h, under N_2, blue LEDs. Yields of isolated product **2a**, in parentheses yields determined by ^{1}H-NMR using 1,3,5-trimethoxybenzene as the internal standard.

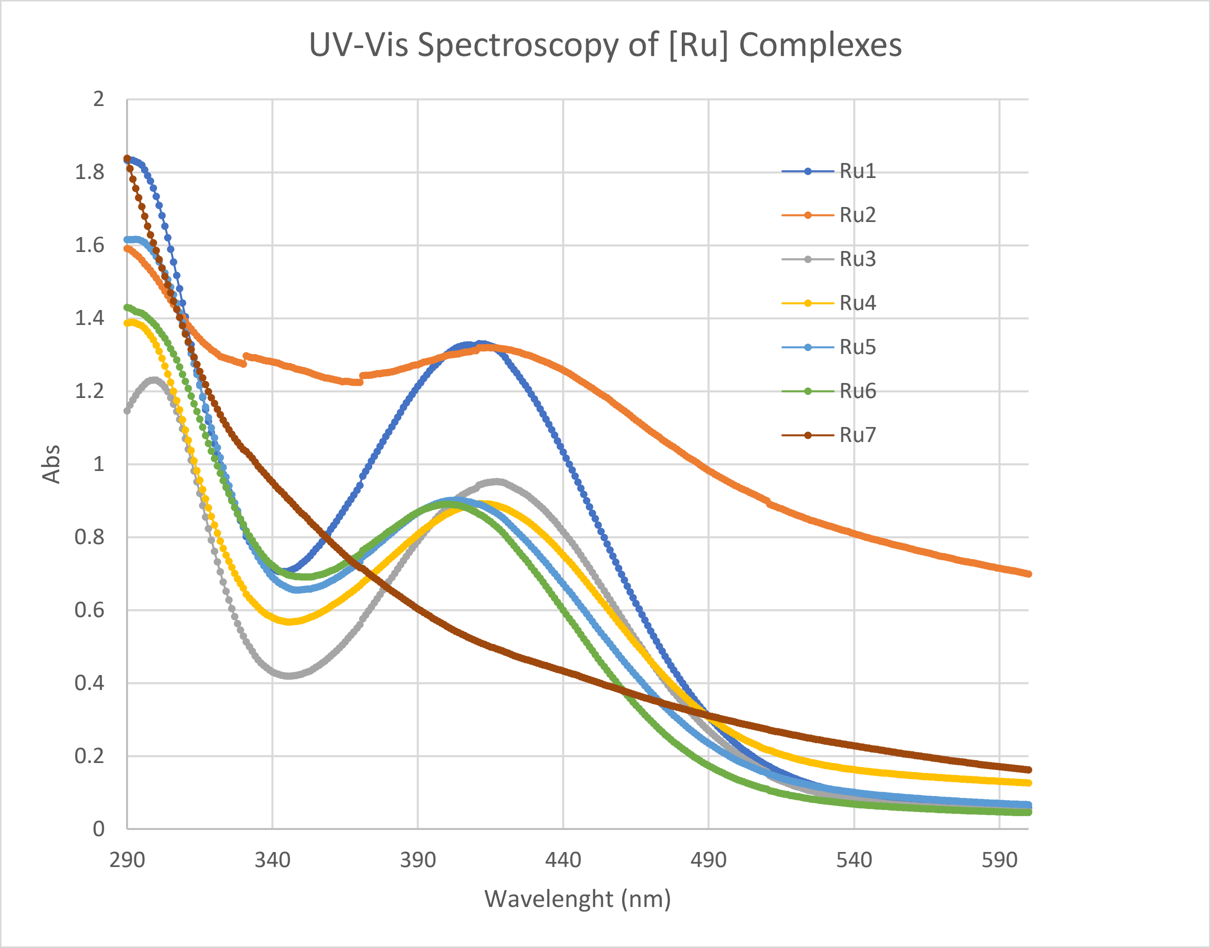

The solutions were prepared in a N_2 filed glovebox by dissolving the [Ru] complex (0.02 mmol) and KOAc (0.04 mmol) in 1 mL of 1,4-dioxane. The solutions were stirred in the dark overnight. Then, 50 µL of the solutions were taken, added to a 10 mL volumetric flask and the solution was diluted to a total volume of 10 mL. Then, 3 mL of the solutions were taken from the volumetric flask and filled in a quartz cuvette for UV-Vis analysis.

Table S3. Control experiments and optimization of additives.[a]

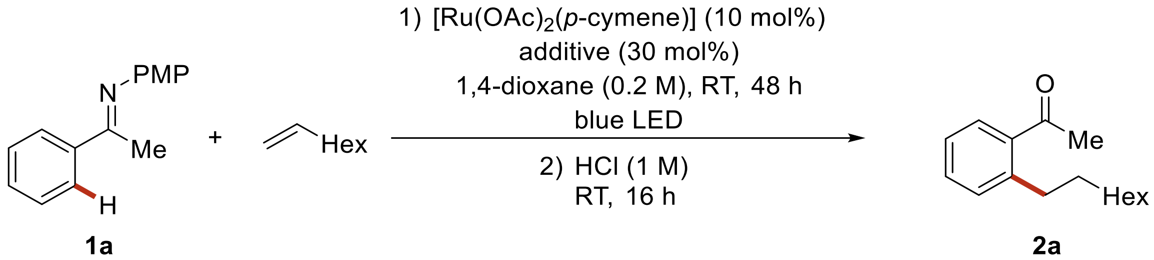

Entry	Deviations from the standard conditions[a]	Yield (%)
1	None	85
2	Without light	0
3	Without [Ru]	0
4[b]	(*n*-BuO)$_2$PO$_2$H as additive (30 mol%)	37
5[b]	(PhO)$_2$PO$_2$H as additive (30 mol%)	19
6[b]	Boc-Val-OH as additive (30 mol%)	(49)
7[b]	Boc-Leu-OH as additive (30 mol%)	(30)
8[b]	Cyclopentanecarboxylic acid as additive (30 mol%)	(28)
9[b]	1-Cyclohexanecarboxylic acid as additive (30 mol%)	43
10[b]	1-Cyclohexenecarboxylic acid as additive (30 mol%)	85
11[b]	1-Methylcyclohexanecarboxylic acid as additive (30 mol%)	41

[a] Reaction conditions: **1a** (0.2 mmol), 1-octene (0.6 mmol**)**, [Ru(OAc)$_2$(*p*-cymene)] (10 mol%), additive (30 mol%), 1,4-dioxane (1.0 mL), 30–35°C, 48 h, under N$_2$, blue LEDs. Yields of isolated product **2a**, in parentheses yields determined by ^{1}H-NMR using dibromomethane as the internal standard. [b] [RuCl$_2$(*p*-cymene)]$_2$ as catalyst.

4 Visible Light-Induced Ruthenium-Catalyzed Hydroarylation of Olefins

General Procedure D:

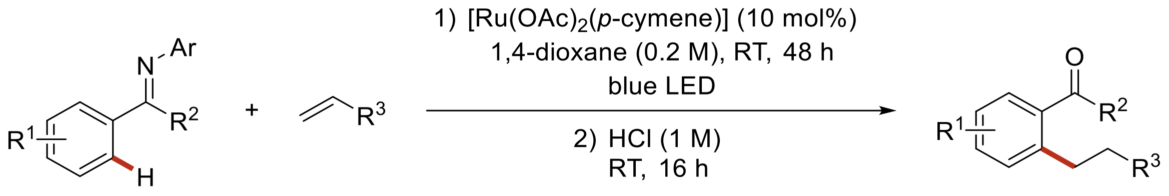

1 **2**

To an oven-dried 10 mL glass vial containing a teflon-coated magnetic stirring bar the ketimine (0.2 mmol, 1.0 equiv.) and [Ru(OAc)$_2$(*p*-cymene)] (7.1 mg, 20 µmol, 10 mol%) were added. The vial was transferred into a N_2-filled glovebox before the terminal alkene (0.6 mmol, 3.0 equiv.) and 1,4-dioxane (1.0 mL) were added. The vial was closed, sealed with parafilm, and transferred out of the glovebox. Subsequently, the reaction mixture was stirred for 48 h under blue light irradiation (two Kessil PR160L lamps, 456 nm, 5 cm distance from vial). Afterwards, the reaction was quenched by the addition of HCl (1 M, 3 mL). The resulting mixture was stirred vigorously for 16 h at ambient temperature. After extraction with CH_2Cl_2 (3 x 2 mL), drying over Na_2SO_4 and filtration, volatiles were removed under reduced pressure. Purification of the residue by column chromatography on silica gel (hexane/EtOAc) afforded the corresponding product.

1-(2-Octylphenyl)ethan-1-one (2a)

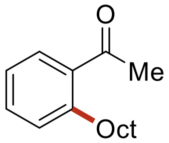

Prepared according to general procedure D using (*E*)-*N*-(4-methoxyphenyl)-1-phenylethan-1-imine (45.1 mg, 0.2 mmol) and 1-octene (95 µL, 0.6 mmol). Purification by column chromatography (hexane/EtOAc 20:1) afforded the title compound as colorless oil (39.5 mg, 85%).

^{1}H NMR (400 MHz, $CDCl_3$): δ 7.63 (dd, *J* = 8.1, 1.5 Hz, 1H), 7.42 – 7.37 (m, 1H), 7.29 – 7.23 (m, 2H), 2.89 – 2.80 (m, 2H), 2.59 (s, 3H), 1.63 – 1.51 (m, 2H), 1.42 – 1.25 (m, 10H), 0.90 (m, *J* = 7.3 Hz, 3H) ppm.

^{13}C NMR (101 MHz, $CDCl_3$): δ 202.5 (C_q), 143.0 (C_q), 138.3 (C_q), 131.4 (CH), 131.3 (CH), 129.0 (CH), 125.7 (CH), 34.1 (CH_2), 32.0 (CH_2), 32.0 (CH_2), 30.1 (CH_3), 29.9 (CH_2), 29.6 (CH_2), 29.4 (CH_2), 22.8 (CH_2), 14.3 (CH_3) ppm.

IR (ATR): $\tilde{\nu}$ = 2957, 2924, 2854, 1686, 1600, 1354, 1248, 955, 758, 597 cm^{-1}.

MS (ESI) *m*/*z* (relative intensity): 487 (35) [2M+Na]$^+$, 255 (100) [M+Na]$^+$, 233 [M+H]$^+$ (17).

HRMS (ESI): *m*/*z* calcd. for $C_{16}H_{24}ONa^+$ [M+Na]$^+$ 255.1719, found 255.1719.

1-(4-Methyl-2-octylphenyl)ethan-1-one (2b)

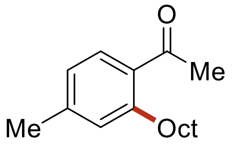

Prepared according to general procedure D using (*E*)-*N*-(4-methoxyphenyl)-1-(*p*-tolyl)ethan-1-imine (47.9 mg, 0.2 mmol) and 1-octene (95 µL, 0.6 mmol). Purification by column chromatography (hexane/EtOAc 30:1) afforded the title compound as colorless oil (44.8 mg, 91%).

^{1}H NMR (300 MHz, $CDCl_3$): δ 7.57 (d, *J* = 8.5 Hz, 1H), 7.06 – 7.04 (m, 2H), 2.86 – 2.80 (m, 2H), 2.55 (s, 3H), 2.36 (s, 3H), 1.57 – 1.52 (m, 2H), 1.30 – 1.27 (m, 10H), 0.87 (t, J = 7.3 Hz, 3H) ppm.

^{13}C NMR (75 MHz, $CDCl_3$): δ 201.7 (C_q), 143.7 (C_q), 142.0 (C_q), 135.1 (C_q), 132.2 (CH), 129.8 (CH), 126.4 (CH), 34.3 (CH_2), 32.0 (CH_2), 32.0 (CH_2), 30.0 (CH_2), 29.9 (CH_3), 29.6 (CH_2), 29.4 (CH_2), 22.8 (CH_2), 21.5 (CH_3), 14.3 (CH_3) ppm.

IR (ATR): $\tilde{\nu}$ = 2923, 2854, 1682, 1609, 1467, 1353, 1229, 966, 812, 676 cm^{-1}.

MS (ESI) *m*/*z* (relative intensity): 269 (100) [M+Na]$^+$, 247 (6) [M+H]$^+$.

HRMS (ESI): *m*/*z* calcd. for $C_{17}H_{26}ONa^+$ [M+Na]$^+$ 269.1876, found 269.1873.

1-(4-Cyclopropyl-2-octylphenyl)ethan-1-one (2c)

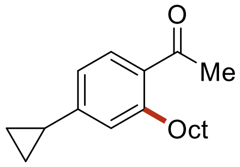

Prepared according to general procedure D using (*E*)-1-(4-cyclopropylphenyl)-*N*-(4-methoxyphenyl)ethan-1-imine (53.1 mg, 0.2 mmol) and 1-octene (95 µL, 0.6 mmol). Purification by column chromatography (hexane/EtOAc 25:1) afforded the title compound as colorless oil (44.1 mg, 81%).

^{1}H NMR (400 MHz, $CDCl_3$) δ 7.57 (d, *J* = 8.0 Hz, 1H), 6.94 (d, *J* = 1.9 Hz, 1H), 6.90 (dd, *J* = 8.0, 1.9 Hz, 1H), 2.90 – 2.78 (m, 2H), 2.54 (s, 3H), 1.89 (tt, *J* = 8.3, 5.0 Hz, 1H), 1.53 (qd, *J* = 7.4, 3.4 Hz, 2H), 1.39 – 1.24 (m, 10H), 1.07 – 0.97 (m, 2H), 0.92 – 0.86 (m, 3H), 0.80 – 0.71 (m, 2H) ppm.

^{13}C NMR (101 MHz, $CDCl_3$) δ 201.3 (C_q), 148.4 (C_q), 143.9 (C_q), 134.8 (C_q), 130.0 (CH), 128.7 (CH), 122.5 (CH), 34.5 (CH_2), 32.0 (CH_2), 32.0 (CH_2), 30.0 (CH_2), 29.8 (CH_3), 29.6 (CH_2), 29.5 (CH_2), 22.8 (CH_2), 15.5 (CH), 14.2 (CH_3), 10.0 (CH_2) ppm.

IR (ATR): $\tilde{\nu}$ = 2925, 2853, 1681, 1607, 1561, 1467, 1353, 1252, 964, 814 cm^{-1}.

MS (ESI) *m*/*z* (relative intensity): 295 (100) [M+Na]$^+$, 273 (17) [M+H]$^+$.

HRMS (ESI): *m*/*z* calcd. for $C_{19}H_{28}ONa^+$ [M+Na]$^+$ 295.2032, found 295.2028.

1-(2-Acetyl-5-isobutylphenyl)octan-1-one (2d)

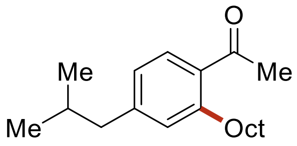

Prepared according to general procedure D using (*E*)-1-(4-isobutylphenyl)-*N*-(4-methoxyphenyl)ethan-1-imine (56.3 mg, 0.2 mmol) and 1-octene (95 µL, 0.6 mmol). Purification by column chromatography (hexane/EtOAc 10:1) afforded the title compound as yellow oil (54.1 mg, 94%).

^{1}H NMR (400 MHz, $CDCl_3$) δ 7.58 (d, *J* = 8.3 Hz, 1H), 7.02 (dt, *J* = 4.6, 2.3 Hz, 2H), 2.88 – 2.82 (m, 2H), 2.56 (s, 3H), 2.48 (d, *J* = 7.2 Hz, 2H), 1.89 (dt, *J* = 13.6, 6.8 Hz, 1H), 1.63 – 1.51 (m, 2H), 1.30 (m, 10H), 0.91 (d, *J* = 6.6 Hz, 6H), 0.91 – 0.82 (m, 3H) ppm.

^{13}C NMR (101 MHz, $CDCl_3$) δ 201.7 (C_q), 145.7 (C_q), 143.4 (C_q), 135.4 (C_q), 132.3 (CH), 129.6 (CH) 126.4 (CH), 45.4 (CH_2), 34.4 (CH_2), 32.0 (CH_2), 32.0 (CH_2), 30.2 (CH_3), 29.9 (CH_2), 29.8 (CH), 29.6 (CH_2), 29.5 (CH_2), 22.8 (CH_2), 22.5 (CH_3), 14.2 (CH_3) ppm.

IR (ATR): $\tilde{\nu}$ = 2955, 2923, 2853, 1682, 1608, 1467, 1353, 1251, 802, 590 cm^{-1}.

MS (ESI) *m*/*z* (relative intensity): 311 (100) $[M+Na]^+$, 289 (20) $[M+H]^+$.

HRMS (ESI): *m*/*z* calcd. for $C_{20}H_{32}ONa^+$ $[M+Na]^+$ 311.2345, found 311.2342.

1-(2-Acetyl-5-(*tert*-butyl)phenyl)octan-1-one (2e)

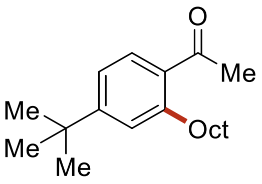

Prepared according to general procedure D using (*E*)-1-(4-(*tert*-butyl)phenyl)-*N*-(4-methoxyphenyl)ethan-1-imine (56.3 mg, 0.2 mmol) and 1-octene (95 µL, 0.6 mmol). Purification by column chromatography (hexane/EtOAc 30:1) afforded the title compound as colorless oil (56.3 mg, 98%).

^{1}H NMR (300 MHz, $CDCl_3$) δ 7.63 (d, *J* = 8.9 Hz, 1H), 7.30 – 7.24 (m, 2H), 2.94 – 2.82 (m, 2H), 2.57 (s, 3H), 1.62 – 1.52 (m, 2H), 1.34 (s, 19H), 0.94 – 0.80 (m, 3H) ppm.

^{13}C NMR (75 MHz, $CDCl_3$) δ 201.6 (C_q), 154.9 (C_q), 143.4 (C_q), 135.0 (C_q), 129.7 (CH), 128.5 (CH), 122.6 (CH), 34.9 (C_q), 34.7 (CH_2), 32.2 (CH_2), 32.0 (CH_2), 31.2 (CH_3), 30.0 (CH_2), 29.8 (CH_3), 29.6 (CH_2), 29.5 (CH_2), 22.8 (CH_2), 14.2 (CH_3) ppm.

IR (ATR): $\tilde{\nu}$ = 2956, 2924, 2855, 1685, 1605, 1465, 1353, 1252, 960, 823 cm^{-1}.

MS (ESI) *m*/*z* (relative intensity): 600 (95) $[2M+Na]^+$, 311 (100) $[M+Na]^+$, 289 (80) $[M+H]^+$.

HRMS (ESI): *m*/*z* calcd. for $C_{20}H_{32}ONa^+$ $[M+Na]^+$ 311.2345, found 311.2352.

1-(3-Octyl-[1,1'-biphenyl]-4-yl)ethan-1-one (2f)

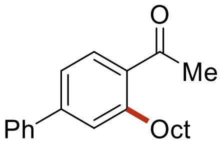

Prepared according to general procedure D using (*E*)-1-([1,1'-biphenyl]-4-yl)-*N*-(4-methoxyphenyl)ethan-1-imine (60.3 mg, 0.2 mmol) and 1-octene (95 µL, 0.6 mmol) with 96 h of reaction time. Purification by column chromatography (hexane/EtOAc 20:1) afforded the title compound as colorless oil (52.0 mg, 84%).

^{1}H NMR (400 MHz, $CDCl_3$): δ 7.73 (d, *J* = 8.6 Hz, 1H), 7.64 – 7.60 (m, 2H), 7.50 – 7.44 (m, 4H), 7.41 – 7.36 (m, 1H), 2.97 – 2.89 (m, 2H), 2.62 (s, 3H), 1.66 – 1.57 (m, 2H), 1.44 – 1.22 (m, 10H), 0.91 – 0.84 (m, 3H) ppm.

^{13}C NMR (101 MHz, $CDCl_3$): δ 201.8 (C_q), 144.2 (C_q), 144.0 (C_q), 140.3 (C_q), 136.6 (C_q), 130.1 (CH), 130.0 (CH), 129.0 (CH), 128.1 (CH), 127.4 (CH), 124.4 (CH), 34.5 (CH_2), 32.1 (CH_2), 32.0 (CH_2), 30.0 (CH_3), 30.0 (CH_2), 29.6 (CH_2), 29.5 (CH_2), 22.8 (CH_2), 14.3 (CH_3) ppm.

IR (ATR): $\tilde{\nu}$ = 2924, 2853, 1680, 1606, 1353, 1245, 956, 828, 764, 696 cm^{-1}.

MS (ESI) *m*/*z* (relative intensity): 331 (100) $[M+Na]^+$, 309 (7) $[M+H]^+$.

HRMS (ESI): *m*/*z* calcd. for $C_{22}H_{28}ONa^+$ $[M+Na]^+$ 331.2032, found 331.2032.

1-(4-Methoxy-2-octylphenyl)ethan-1-one (2g)

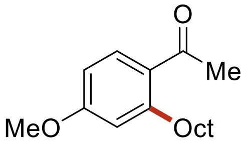

Prepared according to general procedure D using (*E*)-*N*,1-bis(4-methoxyphenyl)ethan-1-imine (51.1 mg, 0.2 mmol) and 1-octene (95 µL, 0.6 mmol). Purification by column chromatography (hexane/EtOAc 20:1) afforded the title compound as orange oil (40.9 mg, 78%).

^{1}H NMR (300 MHz, $CDCl_3$): δ 7.72 – 7.69 (m, 1H), 6.75 – 6.72 (m, 2H), 3.84 (s, 3H), 2.92 – 2.87 (m, 2H), 2.54 (s, 3H), 1.57 – 1.53 (m, 2H), 1.39 – 1.27 (m, 10H), 0.87 (t, *J* = 7.3 Hz, 3H) ppm.

^{13}C NMR (75 MHz, $CDCl_3$): δ 199.9 (C_q), 162.0 (C_q), 147.0 (C_q), 132.5 (CH), 130.1 (C_q), 116.8 (CH), 110.5 (CH), 55.4 (CH_3), 34.9 (CH_2), 32.0 (CH_2), 31.7 (CH_2), 29.9 (CH_2), 29.6 (CH_2), 29.5 (CH_3), 29.4 (CH_2), 22.8 (CH_2), 14.2 (CH_3) ppm.

IR (ATR): $\tilde{\nu}$ = 2925, 2854, 1675, 1602, 1565, 1354, 1248, 1139, 1068, 804 cm^{-1}.

MS (ESI) *m*/*z* (relative intensity): 285 (100) $[M+Na]^+$, 263 (28) $[M+H]^+$.

HRMS (ESI): *m*/*z* calcd. for $C_{17}H_{26}O_2Na^+$ $[M+Na]^+$ 285.1825, found 285.1823.

1-(4-(Methylthio)-2-octylphenyl)ethan-1-one (2h)

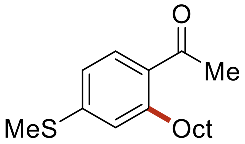

Prepared according to general procedure D using (*E*)-*N*-(4-methoxyphenyl)-1-(4-(methylthio)phenyl)ethan-1-imine (54.3 mg, 0.2 mmol) and 1-octene (95 µL, 0.6 mmol). Purification by column chromatography (hexane/EtOAc 20:1) afforded the title compound as colorless oil (40.1 mg, 72%).

^{1}H NMR (300 MHz, $CDCl_3$): δ 7.61 (d, *J* = 8.6 Hz, 1H), 7.07 (d, *J* = 5.6 Hz, 2H), 2.89 – 2.81 (m, 2H), 2.55 (s, 3H), 2.50 (s, 3H), 1.54 (p, *J* = 7.7 Hz, 2H), 1.41 – 1.22 (m, 10H), 0.87 (t, *J* = 6.6 Hz, 3H) ppm.

^{13}C NMR (75 MHz, $CDCl_3$): δ 200.8 (C_q), 144.5 (C_q), 143.6 (C_q), 133.9 (C_q), 130.4 (CH), 128.1 (CH), 122.4 (CH), 34.5 (CH_2), 32.0 (CH_2), 31.9 (CH_2), 30.0 (CH_2), 29.7 (CH_3), 29.6 (CH_2), 29.4 (CH_2), 22.8 (CH_2), 15.0 (CH_3), 14.3 (CH_3) ppm.

IR (ATR): $\tilde{\nu}$ = 2924, 2854, 1680, 1589, 1546, 1354, 1252, 1116, 955, 810 cm^{-1}.

MS (ESI) *m*/*z* (relative intensity): 301 (100) $[M+Na]^+$, 279 (35) $[M+H]^+$.

HRMS (ESI): *m*/*z* calcd. for $C_{17}H_{26}OSNa^+$ $[M+Na]^+$ 301.1597, found 301.1589.

1-(4-(Dimethylamino)-2-octylphenyl)ethan-1-one (2i)

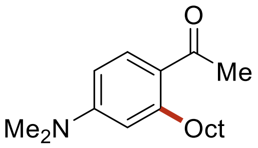

Prepared according to general procedure D using (*E*)-4-(1-((4-methoxyphenyl)imino)ethyl)-*N*,*N*-dimethylaniline (53.7 mg, 0.2 mmol) and 1-octene (95 µL, 0.6 mmol). Purification by column chromatography (hexane/EtOAc 20:1) afforded the title compound as brown oil (41.9 mg, 76%).

^{1}H NMR (300 MHz, $CDCl_3$): δ 7.72 (d, *J* = 8.0 Hz, 1H), 6.51 – 6.47 (m, 2H), 3.03 (s, 6H), 2.97 – 2.91 (m, 2H), 2.52 (s, 3H), 1.59 – 1.54 (m, 2H), 1.35 – 1.28 (m, 10H), 0.88 (t, *J* = 7.3 Hz, 3H) ppm.

^{13}C NMR (75 MHz, $CDCl_3$): δ 198.5 (C_q), 152.4 (C_q), 147.2 (C_q), 133.4 (CH), 124.4 (C_q), 114.0 (CH), 108.2 (CH), 40.0 (CH_3), 35.9 (CH_2), 32.0 (CH_2), 32.0 (CH_2), 30.1 (CH_2), 29.7 (CH_2), 29.5 (CH_2), 29.0 (CH_3), 22.8 (CH_2), 14.2 (CH_3) ppm.

IR (ATR): $\tilde{\nu}$ = 2923, 2853, 1658, 1604, 1548, 1369, 1263, 1207, 1150, 798 cm^{-1}.

MS (ESI) *m*/*z* (relative intensity): 573 (25) $[2M+Na]^+$, 298 (16) $[M+Na]^+$, 276 (100) $[M+H]^+$.

HRMS (ESI): *m*/*z* calcd. for $C_{18}H_{30}NO^+$ $[M+H]^+$ 276.2322, found 276.2324.

1-(4-Octylbenzo[*d*][1,3]dioxol-5-yl)ethan-1-one (2j)

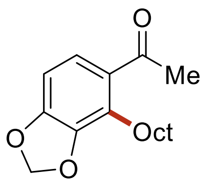

Prepared according to general procedure D using (*E*)-1-(benzo[*d*][1,3]dioxol-5-yl)-*N*-(4-methoxyphenyl)ethan-1-imine (53.9 mg, 0.2 mmol) and 1-octene (95 µL, 0.6 mmol). Purification by column chromatography (hexane/EtOAc 20:1) afforded the title compound as colorless oil (43.9 mg, 79%).

^{1}H NMR (400 MHz, $CDCl_3$): δ 7.34 (d, *J* = 8.2 Hz, 1H), 6.68 (d, *J* = 8.2 Hz, 1H), 6.00 (s, 2H), 2.90 – 2.81 (m, 2H), 2.52 (s, 3H), 1.54 (dtd, *J* = 10.1, 7.8, 7.3, 5.5 Hz, 2H), 1.40 – 1.22 (m, 10H), 0.90 – 0.84 (m, 3H) ppm.

^{13}C NMR (101 MHz, $CDCl_3$): δ 199.6 (C_q), 149.7 (C_q), 147.2 (C_q), 132.0 (C_q), 126.1 (C_q), 125.9 (CH), 105.3 (CH), 101.4 (CH_2), 32.0 (CH_2), 30.2 (CH_2), 30.0 (CH_2), 29.6 (CH_2), 29.5 (CH_3), 29.4 (CH_2), 27.0 (CH_2), 22.8 (CH_2), 14.2 (CH_3) ppm.

IR (ATR): $\tilde{\nu}$ = 2924, 2853, 1673, 1595, 1439, 1257, 1056, 934, 800, 573 cm^{-1}.

MS (ESI) *m*/*z* (relative intensity): 299 (100) $[M+Na]^+$, 277 (35) $[M+H]^+$.

HRMS (ESI): *m*/*z* calcd. for $C_{17}H_{24}O_3Na^+$ $[M+Na]^+$ 299.1618, found 299.1618.

4-Acetyl-3-octylphenyl methanesulfonate (2k)

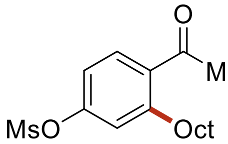

Prepared according to general procedure D using (*E*)-4-(1-((4-methoxyphenyl)imino)ethyl)phenyl methanesulfonate (63.9 mg, 0.2 mmol) and 1-octene (95 µL, 0.6 mmol). Purification by column chromatography (hexane/EtOAc 5:1) afforded the title compound as brown oil (46.4 mg, 71%).

^{1}H NMR (400 MHz, $CDCl_3$): δ 7.68 – 7.65 (m, 1H), 7.19 – 7.16 (m, 2H), 3.17 (s, 3H), 2.80 – 2.87 (m, 2H), 2.56 (s, 3H), 1.58 – 1.53 (m, 2H), 1.30 – 1.26 (m, 10H), 0.87 (t, *J* = 7.4 Hz, 3H) ppm.

^{13}C NMR (101 MHz, $CDCl_3$): δ 201.0 (C_q), 150.8 (C_q), 146.2 (C_q), 137.2 (C_q), 130.9 (CH), 124.2 (CH), 119.0 (CH), 37.9 (CH_3), 34.03 (CH_2), 32.0 (CH_2), 31.6 (CH_2), 30.1 (CH_3), 29.7 (CH_2), 29.5 (CH_2), 29.4 (CH_2), 22.8 (CH_2), 14.2 (CH_3) ppm.

IR (ATR): $\tilde{\nu}$ = 2924, 2855, 1687, 1574, 1373, 1354, 1181, 964, 832, 524 cm^{-1}.

MS (ESI) *m*/*z* (relative intensity): 349 (100) $[M+Na]^+$, 327 (35) $[M+H]^+$.

HRMS (ESI): *m*/*z* calcd for $C_{17}H_{26}O_4SNa^+$ $[M+Na]^+$ 349.1444, found 349.1446.

Methyl 4-acetyl-3-octylbenzoate (2l)

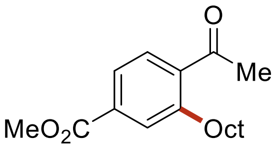

Prepared according to general procedure D using methyl (*E*)-4-(1-((4-methoxyphenyl)imino)ethyl)benzoate (56.7 mg, 0.2 mmol) and 1-octene (95 µL, 0.6 mmol). Purification by column chromatography (hexane/EtOAc 15:1) afforded the title compound as colorless oil (43.0 mg, 74%).

^{1}H NMR (400 MHz, $CDCl_3$): δ 7.91 (d, J = 1.7 Hz, 1H), 7.88 (dd, J = 8.0, 1.7 Hz, 1H), 7.58 (d, J = 8.0 Hz, 1H), 3.92 (s, 3H), 2.85 – 2.78 (m, 2H), 2.57 (s, 3H), 1.62 – 1.50 (m, 2H), 1.37 – 1.20 (m, 10H), 0.90 – 0.83 (m, 3H) ppm.

^{13}C NMR (101 MHz, $CDCl_3$): δ 202.4 (C_q), 166.5 (C_q), 142.6 (C_q), 142.4 (C_q), 132.1 (CH), 128.3 (CH), 126.9 (CH), 52.4 (CH_3), 33.8 (CH_2), 32.0 (CH_2), 31.9 (CH_2), 30.4 (CH_3), 29.8 (CH_2), 29.5 (CH_2), 29.4 (CH_2), 22.8 (CH_2), 14.2 (CH_3) ppm.

IR (ATR): $\tilde{\nu}$ = 2924, 2854, 1723, 1681, 1436, 1272, 1243, 1197, 1115, 769 cm^{-1}.

MS (ESI) *m*/*z* (relative intensity): 313 (85) $[M+Na]^+$, 291 (100) $[M+H]^+$.

HRMS (ESI): *m*/*z* calcd. for $C_{18}H_{27}O_3^+$ $[M+H]^+$ 291.1955, found 291.1953.

1-(4-Fluoro-2-octylphenyl)ethan-1-one (2m)

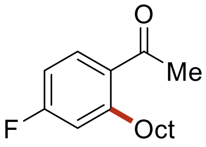

Prepared according to general procedure D using (*E*)-1-(4-fluorophenyl)-*N*-(4-methoxyphenyl)ethan-1-imine (0.2 mmol, 48.7 mg) and 1-octene (95 µL, 0.6 mmol). Purification by column chromatography (hexane/EtOAc 30:1) afforded the title compound as brown oil (43.6 mg, 87%).

^{1}H NMR (300 MHz, $CDCl_3$): δ 7.69 – 7.64 (m, 1H), 6.97 – 6.88 (m, 2H), 2.88 – 2.83 (m, 2H), 2.55 (s, 3H), 1.60 – 1.50 (m, 2H), 1.37 – 1.26 (m, 10H), 0.87 (t, J = 7.4 Hz, 3H) ppm.

^{13}C NMR (75 MHz, $CDCl_3$): δ 200.5 (C_q), 164.2 (d, J_{CF} = 252.5 Hz, C_q), 147.2 (d, J_{CF} = 8.4 Hz, C_q), 134.1 (d, J_{CF} = 2.9 Hz, C_q), 131.9 (d, J_{CF} = 9.4 Hz, CH), 118.0 (d, J_{CF} = 21.1 Hz, CH), 112.6 (d, J_{CF} = 21.4 Hz, CH), 34.3 (d, J_{CF} = 1.5 Hz, CH_2), 32.0 (CH_2), 31.6 (CH_2), 29.8 (CH_3), 29.8 (CH_2), 29.6 (CH_2), 29.4 (CH_2), 22.8 (CH_2), 14.2 (CH_3) ppm.

^{19}F NMR (282 MHz, $CDCl_3$): –108.1 ppm.

IR (ATR): $\tilde{\nu}$ = 2925, 2855, 1687, 1606, 1582, 1354, 1234, 1121, 974, 814 cm^{-1}.

MS (ESI) *m*/*z* (relative intensity): 273 (100) $[M+Na]^+$, 251 $[M+H]^+$ (25).

HRMS (ESI): *m*/*z* calcd. for $C_{16}H_{23}OFNa^+$ $[M+Na]^+$ 273.1625, found 273.1629.

1-(2-Acetyl-5-chlorophenyl)octan-1-one (2n)

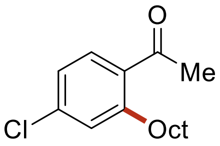

Prepared according to general procedure D using (*E*)-1-(4-chlorophenyl)-*N*-(4-methoxyphenyl)ethan-1-imine (52.0 mg, 0.2 mmol) and 1-octene (95 µL, 0.6 mmol). Purification by column chromatography (hexane/EtOAc 20:1) afforded the title compound as colorless oil (38.5 mg, 72%).

^{1}H NMR (300 MHz, $CDCl_3$): δ 7.56 (d, *J* = 8.1 Hz, 1H), 7.25 – 7.19 (m, 2H), 2.87 – 2.75 (m, 2H), 2.55 (s, 3H), 1.59 – 1.49 (m, 2H), 1.43 – 1.16 (m, 10H), 0.95 – 0.76 (m, 3H) ppm.

^{13}C NMR (75 MHz, $CDCl_3$): δ 201.0 (C_q), 145.4 (C_q), 137.4 (C_q), 136.3 (C_q), 131.2 (CH), 130.6 (CH), 125.9 (CH), 34.0 (CH_2), 32.0 (CH_2), 31.7 (CH_2), 30.0 (CH_3), 29.8 (CH_2), 29.5 (CH_2), 29.4 (CH_2), 22.8 (CH_2), 14.2 (CH_3) ppm.

IR (ATR): $\tilde{\nu}$ = 2958, 2925, 1687, 1591, 1557, 1355, 1244, 1111, 957, 814 cm^{-1}.

MS (ESI) *m*/*z* (relative intensity): 289 (100) $[M+Na]^+$, 267 $[M+H]^+$ (50).

HRMS (ESI): *m*/*z* calcd. for $C_{16}H_{23}ClONa^+$ $[M+Na]^+$ 289.1330, found 289.1325.

1-(2-Octyl-4-(trifluoromethyl)phenyl)ethan-1-one (2o)

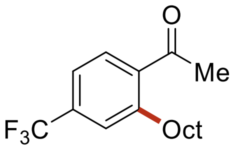

Prepared according to general procedure D using (*E*)-*N*-(4-methoxyphenyl)-1-(4-(trifluoromethyl)phenyl)ethan-1-imine (58.7 mg, 0.2 mmol) and 1-octene (95 µL, 0.6 mmol). Purification by column chromatography (hexane/EtOAc 25:1) afforded the title compound as brown oil (55.3 mg, 92%).

^{1}H NMR (300 MHz, $CDCl_3$): δ 7.65 (dd, *J* = 8.6, 0.9 Hz, 1H), 7.54 – 7.45 (m, 2H), 2.90 – 2.79 (m, 2H), 2.59 (s, 3H), 1.65 – 1.49 (m, 2H), 1.43 – 1.19 (m, 10H), 0.92 – 0.82 (m, 3H).

^{13}C NMR (75 MHz, $CDCl_3$): δ 202.0 (C_q), 143.4 (C_q), 141.6 (C_q), 132.7 (q, J_{CF} = 32.4 Hz, C_q), 128.7 (CH), 127.8 (q, J_{CF} = 3.8 Hz, CH), 123.8 (q, J_{CF} = 272.8 Hz, C_q), 122.7 (q, J_{CF} = 3.8 Hz, CH), 33.9 (CH_2), 32.0 (CH_2), 31.8 (CH_2), 30.7 (CH_3), 29.8 (CH_2), 29.5 (CH_2), 29.4 (CH_2), 22.8 (CH_2), 14.2 (CH_3) ppm.

^{19}F NMR (282 MHz, $CDCl_3$): δ –63.1 ppm.

IR (ATR): $\tilde{\nu}$ = 2956, 2926, 2856, 1696, 1332, 1248, 1168, 1129, 1088, 829 cm^{-1}.

MS (ESI) *m*/*z* (relative intensity): 323 (100) $[M+Na]^+$.

HRMS (ESI): *m*/*z* calcd. for $C_{17}H_{23}F_3ONa^+$ $[M+Na]^+$ 323.1593, found 323.1580.

1-(4-(Methylsulfonyl)-2-octylphenyl)ethan-1-one (2p)

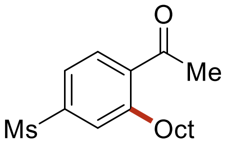

Prepared according to general procedure D using (*E*)-*N*-(4-methoxyphenyl)-1-(4-(methylsulfonyl)phenyl)ethan-1-imine (60.7 mg, 0.2 mmol) and 1-octene (95 µL, 0.6 mmol). Purification by column chromatography (hexane/EtOAc 5:1) afforded the title compound as colorless oil (44.1 mg, 71%).

^{1}H NMR (300 MHz, $CDCl_3$): δ 7.82 – 7.80 (m, 2H), 7.69 – 7.66 (m, 1H), 3.05 (s, 3H), 2.85 – 2.80 (m, 2H), 2.59 (s, 3H), 1.60 – 1.54 (m, 2H), 1.30 – 1.25 (m, 10H), 0.86 (t, *J* = 7.3 Hz, 3H) ppm.

^{13}C NMR (75 MHz, $CDCl_3$): δ 201.9 (C_q), 143.8 (C_q), 143.5 (C_q), 142.2 (C_q), 129.6 (CH), 128.8 (CH), 124.8 (CH), 44.5 (CH_3), 33.7 (CH_2), 31.9 (CH_2), 31.7 (CH_2), 30.5 (CH_3), 29.7 (CH_2), 29.4 (CH_2), 29.3 (CH_2), 22.7 (CH_2), 14.2 (CH_3) ppm.

IR (ATR): $\tilde{\nu}$ = 2926, 2854, 1696, 1358, 1319, 1152, 955, 764, 633, 496 cm^{-1}.

MS (ESI) *m*/*z* (relative intensity): 643 (100) $[2M+Na]^+$, 333 (45) $[M+Na]^+$, 311 (5) $[M+H]^+$.

HRMS (ESI): *m/z* calcd. for $C_{17}H_{26}O_3SNa^+$ $[M+Na]^+$ 333.1495, found 333.1495.

1-(2-Octyl-4-(thiophen-2-yl)phenyl)ethan-1-one (2q)

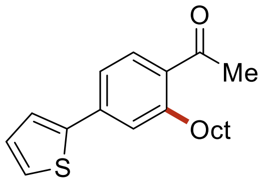

Prepared according to general procedure D using (*E*)-N-(4-methoxyphenyl)-1-(4-(thiophen-2-yl)phenyl)ethan-1-imine (61.5 mg, 0.2 mmol) and 1-octene (95 µL, 0.6 mmol). Purification by column chromatography (hexane/EtOAc 30:1) afforded the title compound as brown oil (40.3 mg, 64%).

^{1}H NMR (300 MHz, $CDCl_3$): δ 7.67 (d, *J* = 8.6 Hz, 1H), 7.53 – 7.46 (m, 2H), 7.39 (d, *J* = 3.6 Hz, 1H), 7.34 (d, *J* = 5.1 Hz, 1H), 7.11 (dd, *J* = 5.1, 3.6 Hz, 1H), 3.09 – 2.73 (m, 2H), 2.59 (s, 3H), 1.72 – 1.51 (m, 2H), 1.44 – 1.22 (m, 10H), 0.89 (t, *J* = 7.4 Hz, 3H) ppm.

^{13}C NMR (75 MHz, $CDCl_3$): δ 201.2 (C_q), 144.4 (C_q), 143.3 (C_q), 137.2 (C_q), 136.4 (C_q), 130.4 (CH), 128.6 (CH), 128.3 (CH), 126.1 (CH), 124.3 (CH), 123.0 (CH), 34.4 (CH_2), 32.0 (CH_2), 32.0 (CH_2), 29.9 (CH_2), 29.9 (CH_3), 29.6 (CH_2), 29.4 (CH_2), 22.8 (CH_2), 14.2 (CH_3) ppm.

IR (ATR): $\tilde{\nu}$ = 2957, 2924, 2853, 1681, 1603, 1431, 1353, 1246, 816, 697 cm^{-1}.

MS (ESI) *m/z* (relative intensity): 651 (100) $[2M+Na]^+$, 337 (75) $[M+Na]^+$, 315 (30) $[M+H]^+$.

HRMS (ESI): *m/z* calcd. for $C_{20}H_{26}OSNa^+$ $[M+Na]^+$ 337.1597, found 337.1596.

1-(2-Octyl-4-(thiophen-3-yl)phenyl)ethan-1-one (2r)

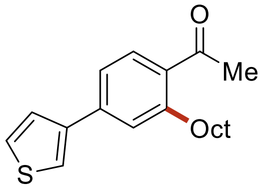

Prepared according to general procedure D using (*E*)-*N*-(4-methoxyphenyl)-1-(4-(thiophen-3-yl)phenyl)ethan-1-imine (61.5 mg, 0.2 mmol) and 1-octene (95 µL, 0.6 mmol). Purification by column chromatography (hexane/EtOAc 20:1) afforded the title compound as brown oil (31.4 mg, 50%).

^{1}H NMR (300 MHz, $CDCl_3$): δ 7.70 (d, *J* = 8.9 Hz, 1H), 7.55 – 7.53 (m, 1H), 7.48 – 7.43 (m, 2H), 7.44 – 7.38 (m, 2H), 3.04 – 2.83 (m, 2H), 2.60 (s, 3H), 1.69 – 1.50 (m, 2H), 1.34 – 1.28 (m, 10H), 0.89 (t, *J* = 7.3 Hz, 3H) ppm.

^{13}C NMR (75 MHz, $CDCl_3$): δ 201.4 (C_q), 144.3 (C_q), 141.4 (C_q), 138.7 (C_q), 136.3 (C_q), 130.3 (CH), 129.3 (CH), 126.7 (CH), 126.4 (CH), 123.6 (CH), 121.7 (CH), 34.5 (CH_2), 32.0 (CH_2), 32.0 (CH_2), 30.0 (CH_2), 29.9 (CH_3), 29.6 (CH_2), 29.4 (CH_2), 22.8 (CH_2), 14.2 (CH_3) ppm.

IR (ATR): $\tilde{\nu}$ = 2957, 2924, 2854, 1680, 1604, 1561, 1422, 1353, 1245, 780 cm^{-1}.

MS (ESI) *m/z* (relative intensity): 651 (100) $[2M+Na]^+$, 337 (83) $[M+Na]^+$, 315 (70) $[M+H]^+$.

HRMS (ESI): *m/z* calcd. for $C_{20}H_{26}OSNa^+$ $[M+Na]^+$ 337.1597, found 337.1597.

1-(4-(Furan-3-yl)-2-octylphenyl)ethan-1-one (2s)

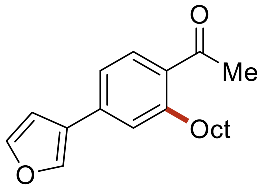

Prepared according to general procedure D using (*E*)-1-(4-(furan-3-yl)phenyl)-*N*-(4-methoxyphenyl)ethan-1-imine (58.3 mg, 0.2 mmol) and 1-octene (95 µL, 0.6 mmol). Purification by column chromatography (hexane/EtOAc 10:1) afforded the title compound as yellow oil (56.7 mg, 95%).

^{1}H NMR (400 MHz, $CDCl_3$) δ 7.79 (t, J = 1.2 Hz, 1H), 7.67 (d, J = 8.4 Hz, 1H), 7.50 (t, J = 1.7 Hz, 1H), 7.36 (dq, J = 3.8, 1.9 Hz, 2H), 6.75 – 6.70 (m, 1H), 2.95 – 2.85 (m, 2H), 2.58 (s, 3H), 1.62 – 1.55 (m, 2H), 1.43 – 1.20 (m, 10H), 0.95 – 0.81 (m, 3H) ppm.

^{13}C NMR (101 MHz, $CDCl_3$) δ 201.3 (C_q), 144.3 (C_q), 144.1 (CH), 139.5 (CH), 136.3 (C_q), 135.6 (C_q), 130.3 (CH), 128.7 (CH), 125.9 (C_q), 123.0 (CH), 108.9, (CH) 34.5 (CH_2), 32.0 (CH_2), 30.0 (CH_2), 29.9 (CH_3), 29.9 (CH_2), 29.6 (CH_2), 29.5 (CH_2), 22.8 (CH_2), 14.2 (CH_3) ppm.

IR (ATR): $\tilde{\nu}$ = 2958, 2923, 2853, 1681, 1611 1353, 1163, 1098, 1068, 947 cm^{-1}.

MS (ESI) *m*/*z* (relative intensity): 619 (85) $[2M+Na]^+$, 321 (100) $[M+Na]^+$, 299 (43) $[M+H]^+$.

HRMS (ESI): *m*/*z* calcd. for $C_{20}H_{26}O_2Na^+$ $[M+Na]^+$ 299.2006, found 299.2004.

1-(5-Methyl-2-octylphenyl)ethan-1-one (2t)

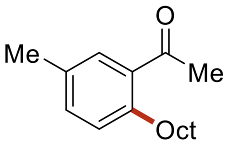

Prepared according to general procedure D using (E)-N-(4-methoxyphenyl)-1-(m-tolyl)ethan-1-imine (47.9 mg, 0.2 mmol) and 1-octene (95 µL, 0.6 mmol). Purification by column chromatography (hexane/EtOAc 25:1) afforded the title compound as colorless oil (29.1 mg, 59%).

^{1}H NMR (300 MHz, $CDCl_3$): δ 7.40 (s, 1H), 7.19 (d, J = 9.0 Hz, 1H), 7.14 (d, J = 9.1 Hz, 1H), 2.81 – 2.78 (m, 2H), 2.56 (s, 3H), 2.36 (s, 3H), 1.60 – 1.48 (m, 2H), 1.34 – 1.26 (m, 10H), 0.87 (t, J = 7.4 Hz, 3H) ppm.

^{13}C NMR (75 MHz, $CDCl_3$): δ 202.7 (C_q), 139.9 (C_q), 138.3 (C_q), 135.2 (C_q), 132.1 (CH), 131.2 (CH), 129.6 (CH), 33.7 (CH_2), 32.1 (CH_2), 32.0 (CH_2), 30.1 (CH_3), 29.9 (CH_2), 29.6 (CH_2), 29.4 (CH_2), 22.8 (CH_2), 21.1 (CH_3), 14.3 (CH_3) ppm.

IR (ATR): $\tilde{\nu}$ = 2924, 2854, 1686, 1468, 1353, 1258, 1187, 956, 826, 618 cm^{-1}.

MS (ESI) *m*/*z* (relative intensity): 515 (80) $[2M+Na]^+$, 493 (45) $[2M+H]^+$, 269 (100) $[M+Na]^+$, 247 (90) $[M+H]^+$.

HRMS (ESI): *m*/*z* calcd. for $C_{17}H_{26}ONa^+$ $[M+Na]^+$ 269.1876, found 269.1878.

1-(2-Octyl-5-(trifluoromethyl)phenyl)ethan-1-one (2u)

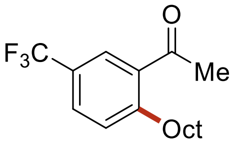

Prepared according to general procedure D using (E)-N-(4-methoxyphenyl)-1-(3-(trifluoromethyl)phenyl)ethan-1-imine (58.7 mg, 0.2 mmol) and 1-octene (95 µL, 0.6 mmol). Purification by column chromatography (hexane/EtOAc 30:1) afforded the title compound as yellow oil (44.5 mg, 74%).

^{1}H NMR (400 MHz, $CDCl_3$): δ 7.82 (s, 1H), 7.62 (d, J = 8.1 Hz, 1H), 7.38 (d, J = 8.1 Hz, 1H), 2.89 – 2.84 (m, 2H), 2.61 (s, 3H), 1.63 – 1.51 (m, 2H), 1.30 – 1.27 (m, 10H), 0.87 (t, J = 7.3 Hz, 3H) ppm.

^{13}C NMR (101 MHz, $CDCl_3$): δ 201.1 (C_q), 147.0 (C_q), 138.7 (C_q), 131.8 (CH), 128.3 (q, J_{CF} = 32.7 Hz, C_q), 127.7 (q, J_{CF} = 3.6 Hz, CH), 125.6 (q, J = 3.6 Hz, CH), 124.0 (q, J = 271.8 Hz, C_q), 34.0 (CH_2), 32.0 (CH_2), 31.8 (CH_2), 30.1 (CH_3), 29.8 (CH_2), 29.5 (CH_2), 29.4 (CH_2), 22.8 (CH_2), 14.2 (CH_3) ppm.

^{19}F NMR (282 MHz, $CDCl_3$) δ –62.5 ppm.

IR (ATR): $\tilde{\nu}$ = 2926, 2856, 1695, 1358, 1335, 1237, 1170, 1126, 1086, 1066 cm^{-1}.

MS (ESI) *m*/*z* (relative intensity): 323 (100) $[M+Na]^+$.

HRMS (ESI): *m*/*z* calcd. for $C_{17}H_{23}OF_3Na^+$ $[M+Na]^+$ 323.1593, found 323.1595.

1-(2-Hexadecylphenyl)ethan-1-one (2v)

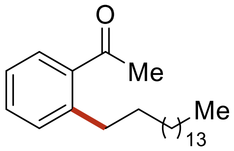

Prepared according to general procedure D using (*E*)-*N*-(4-methoxyphenyl)-1-phenylethan-1-imine (45.1 mg, 0.2 mmol) and hexadec-1-ene (134.7 mg, 0.6 mmol). Purification by column chromatography (hexane/EtOAc 25:1) afforded the title compound as brown oil (57.5 mg, 95%).

^{1}H NMR (300 MHz, $CDCl_3$): δ 7.61 (dd, *J* = 8.1, 1.5 Hz, 1H), 7.43 – 7.38 (m, 1H), 7.29 – 7.24 (m, 2H), 2.89 – 2.84 (m, 2H), 2.60 (s, 3H), 1.62 – 1.57 (m, 2H), 1.35 – 1.29 (m, 26H), 0.92 (t, *J* = 7.3 Hz, 3H) ppm.

^{13}C NMR (75 MHz, $CDCl_3$): δ 202.3 (C_q), 143.0 (C_q), 138.2 (C_q), 131.3 (CH), 131.3 (CH), 129.0 (CH), 125.7 (CH), 34.1 (CH_2), 32.1 (CH_2), 32.0 (CH_2), 30.1 (CH_3), 29.9 (CH_2), 29.9 (CH_2), 29.9 (CH_2), 29.8 (CH_2), 29.8 (CH_2), 29.8 (CH_2), 29.8 (CH_2), 29.8 (CH_2), 29.7 (CH_2), 29.7 (CH_2), 29.5 (CH_2), 22.8 (CH_2), 14.3 (CH_3) ppm.

IR (ATR): $\tilde{\nu}$ = 2961, 2922, 2852, 1688, 1467, 1353, 1248, 954, 757, 609 cm^{-1}.

MS (ESI) *m*/*z* (relative intensity): 711 (85) $[2M+Na]^+$, 367 (100) $[M+Na]^+$, 345 (45) $[M+H]^+$.

HRMS (ESI): *m*/*z* calcd. for $C_{24}H_{40}ONa^+$ $[M+Na]^+$ 367.2971, found 367.2966.

1-(2-(4-Phenylbutyl)phenyl)ethan-1-one (2w)

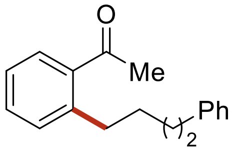

Prepared according to general procedure D using (*E*)-*N*-(4-methoxyphenyl)-1-phenylethan-1-imine (45.1 mg, 0.2 mmol) and but-3-en-1-ylbenzene (79.3 mg, 0.6 mmol). Purification by column chromatography (hexane/EtOAc 20:1) afforded the title compound as colorless oil (49.5 mg, 98%).

^{1}H NMR (300 MHz, $CDCl_3$): δ 7.66 (d, *J* = 8.1 Hz, 1H), 7.45 – 7.39 (m, 1H), 7.34 – 7.25 (m, 4H), 7.23 – 7.18 (m, 3H), 2.96 – 2.91 (m, 2H), 2.69 (t, *J* = 7.5 Hz, 2H), 2.60 (s, 3H), 1.80 – 1.61 (m, 4H) ppm.

^{13}C NMR (75 MHz, $CDCl_3$): δ 202.4 (C_q), 142.8 (C_q), 142.8 (C_q), 138.1 (C_q), 131.5 (CH), 131.3 (CH), 129.2 (CH), 128.6 (CH), 128.4 (CH), 125.8 (CH), 125.7 (CH), 35.9 (CH_2), 34.0 (CH_2), 31.6 (CH_2), 31.6 (CH_2), 30.1 (CH_3) ppm.

IR (ATR): $\tilde{\nu}$ = 2691, 2925, 2849, 1623, 1603, 1501, 1254, 1235, 1031, 834 cm^{-1}.

MS (ESI) *m*/*z* (relative intensity): 527 (4) $[2M+Na]^+$, 275 (100) $[M+Na]^+$, 253 (20) $[M+H]^+$.

HRMS (ESI): *m*/*z* calcd. for $C_{18}H_{20}ONa^+$ $[M+Na]^+$ 253.1587, found 253.1590.

1-(2-(10-Bromodecyl)phenyl)ethan-1-one (2x)

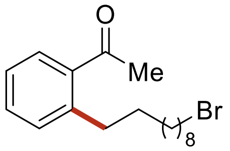

Prepared according to general procedure D using (*E*)-*N*-(4-methoxyphenyl)-1-phenylethan-1-imine (45.1 mg, 0.2 mmol) and 10-bromodec-1-ene (131.5 mg, 0.6 mmol). Purification by column chromatography (hexane/EtOAc 25:1) afforded the title compound as yellow oil (49.5 mg, 73%).

^{1}H NMR (300 MHz, $CDCl_3$): δ 7.66 – 7.62 (m, 1H), 7.43 – 7.40 (m, 1H), 7.38 – 7.25 (m, 2H), 3.44 – 3.40 (t, *J* = 6.9 Hz, 2H), 2.88 – 2.83 (m, 2H), 2.59 (s, 3H), 1.87 (p, *J* = 6.9 Hz, 2H), 1.60 – 1.55 (m, 2H), 1.34 – 1.31 (m, 12H) ppm.

^{13}C NMR (75 MHz, $CDCl_3$): δ 202.4 (C_q), 143.0 (C_q), 138.2 (C_q), 131.4 (CH), 131.2 (CH), 129.1 (CH), 125.7 (CH), 34.2 (CH_2), 34.1 (CH_2), 33.0 (CH_2), 32.0 (CH_2), 30.1 (CH_3), 29.8 (CH_2), 29.6 (CH_2), 29.5 (CH_2), 29.5 (CH_2), 28.9 (CH_2), 28.3 (CH_2) ppm.

IR (ATR): $\tilde{\nu}$ = 2923, 2852, 1686, 1599, 1571, 1466, 1354, 1246, 954, 759 cm^{-1}.

MS (ESI) *m*/*z* (relative intensity): 699 (20) $[2M+Na]^+$, 361 (100) $[M+Na]^+$, 339 (30) $[M+H]^+$.

HRMS (ESI): *m*/*z* calcd. for $C_{18}H_{27}BrONa^+$ $[M+Na]^+$ 361.1137, found 361.1148.

1-(2-(6-Hydroxyhexyl)phenyl)ethan-1-one (2y)

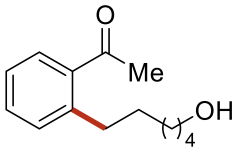

Prepared according to general procedure D using (*E*)-*N*-(4-methoxyphenyl)-1-phenylethan-1-imine (45.1 mg, 0.2 mmol) and hex-5-en-1-ol (60.1 mg, 0.6 mmol). Purification by column chromatography (hexane/EtOAc 4:1) afforded the title compound as yellow oil (29.3 mg, 67%).

^{1}H NMR (400 MHz, $CDCl_3$): δ 7.63 (d, *J* = 7.8 Hz, 1H), 7.39 (t, *J* = 7.5 Hz, 1H), 7.30 – 7.22 (m, 2H), 3.64 (t, *J* = 6.6 Hz, 2H), 2.88 – 2.81 (m, 2H), 2.58 (s, 3H), 1.61 – 1.55 (m, 4H), 1.43 – 1.35 (m, 4H).

^{13}C NMR (101 MHz, $CDCl_3$): δ 202.5 (C_q), 142.9 (C_q), 138.0 (C_q), 131.4 (CH), 131.3 (CH), 129.2 (CH), 125.8 (CH), 63.0 (CH_2), 34.0 (CH_2), 32.8 (CH_2), 31.8 (CH_2), 30.0 (CH_3), 29.5 (CH_2), 25.6 (CH_2).

IR (ATR): $\tilde{\nu}$ = 3394, 2927, 1682, 1599, 1571, 1458, 1335, 1054, 955, 759 cm^{-1}.

MS (ESI) *m*/*z* (relative intensity): 463 (27) $[2M+Na]^+$, 243 (100) $[M+Na]^+$, 203 (5) $[M+H]^+$.

HRMS (ESI): *m*/*z* calcd. for $C_{14}H_{20}O_2Na^+$ $[M+Na]^+$ 221.1536 found 221.1537.

1-(2-(2-(Trimethylsilyl)ethyl)phenyl)ethan-1-one (2z)

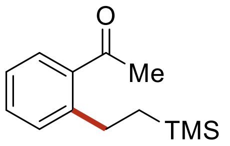

Prepared according to general procedure D using (*E*)-*N*-(4-methoxyphenyl)-1-phenylethan-1-imine (45.1 mg, 0.2 mmol) and trimethyl(vinyl)silane (60.1 mg, 0.6 mmol). Purification by column chromatography (hexane/EtOAc 30:1) afforded the title compound as brown oil (41.9 mg, 95%).

^{1}H NMR (300 MHz, $CDCl_3$): δ 7.65 (d, *J* = 7.7 Hz, 1H), 7.44 – 7.39 (m, 1H), 7.31 – 7.24 (m, 2H), 2.89 – 2.84 (m, 2H), 2.60 (s, 3H), 0.87 – 0.81 (m, 2H), 0.07 (s, 9H) ppm.

^{13}C NMR (75 MHz, $CDCl_3$): δ 202.2 (C_q), 146.0 (C_q), 137.5 (C_q), 131.6 (CH), 130.6 (CH), 129.3 (CH), 125.6 (CH), 30.0 (CH_3), 28.5 (CH_2), 19.6 (CH_2), –1.7 (CH_3) ppm.

IR (ATR): $\tilde{\nu}$ = 2953, 1686, 1599, 1354, 1246, 861, 842, 758, 690, 600 cm^{-1}.

MS (ESI) *m/z* (relative intensity): 463 (15) $[2M+Na]^+$, 243 (100) $[M+Na]^+$, 221 (20) $[M+H]^+$.

HRMS (ESI): *m/z* calcd. for $C_{13}H_{20}OSiNa^+$ $[M+Na]^+$ 243.1176, found 243.1182.

1-(2-(3-(Trimethylsilyl)propyl)phenyl)ethan-1-one (2aa)

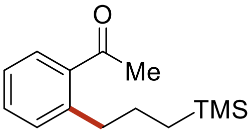

Prepared according to general procedure D using (*E*)-*N*-(4-methoxyphenyl)-1-phenylethan-1-imine (45.1 mg, 0.2 mmol) and allyltrimethylsilane (68.6 mg, 0.6 mmol). Purification by column chromatography (hexane/EtOAc 30:1) afforded the title compound as orange oil (42.7 mg, 91%).

^{1}H NMR (300 MHz, $CDCl_3$): δ 7.64 (d, *J* = 6.9 Hz, 1H), 7.43 – 7.39 (m, 1H), 7.30 – 7.25 (m, 2H), 2.90 – 2.85 (m, 2H), 2.60 (s, 3H), 1.65 – 1.55 (m, 2H), 0.62 – 0.57 (m, 2H), 0.00 (s, 9H) ppm.

^{13}C NMR (75 MHz, $CDCl_3$): δ 202.5 (C_q), 142.7 (C_q), 138.3 (C_q), 131.4 (CH), 131.3 (CH), 129.1 (CH), 125.8 (CH), 38.0 (CH_2), 30.1 (CH_3), 26.5 (CH_2), 17.1 (CH_2), –1.6 (CH_3) ppm.

IR (ATR): $\tilde{\nu}$ = 2952, 1687, 1600, 1572, 1247, 954, 864, 836, 758, 600 cm^{-1}.

MS (ESI) *m/z* (relative intensity): 491 (22) $[2M+Na]^+$, 257 (100) $[M+Na]^+$, 235 (22) $[M+H]^+$.

HRMS (ESI): *m/z* calcd. for $C_{14}H_{22}OSiNa^+$ $[M+Na]^+$ 257.1332, found 257.1338.

1-(2-(2-(Dimethyl(phenyl)silyl)ethyl)phenyl)ethan-1-one (2ab)

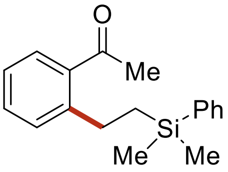

Prepared according to general procedure D using (*E*)-*N*-(4-methoxyphenyl)-1-phenylethan-1-imine (45.1 mg, 0.2 mmol) and dimethyl(phenyl)(vinyl)silane (97.4 mg, 0.6 mmol). Purification by column chromatography (hexane/EtOAc 10:1) afforded the title compound as brown oil (50.8 mg, 90%).

^{1}H NMR (400 MHz, $CDCl_3$): δ 7.67 (d, *J* = 7.7 Hz, 1H), 7.61 (d, *J* = 4.3 Hz, 2H), 7.44 – 7.40 (m, 4H), 7.30 – 7.26 (m, 2H), 2.96 – 2.87 (m, 2H), 2.60 (s, 3H), 1.18 – 1.09 (m, 2H), 0.39 (s, 6H).

^{13}C NMR (101 MHz, $CDCl_3$): δ 202.1 (C_q), 145.7 (C_q), 139.3 (C_q), 137.4 (C_q), 133.7 (CH), 131.6 (CH), 130.7 (CH), 129.3 (CH), 129.0 (CH), 127.9 (CH), 125.7 (CH), 29.9 (CH_3), 28.5 (CH_2), 18.6 (CH_2), –3.0 (CH_3) ppm.

IR (ATR): $\tilde{\nu}$ = 2956, 1685, 1427, 1245, 1114, 841, 811, 758, 700, 602 cm^{-1}.

MS (ESI) *m/z* (relative intensity): 587 (20) $[2M+Na]^+$, 305 (100) $[M+Na]^+$.

HRMS (ESI): *m/z* calcd. for $C_{18}H_{22}OSiNa^+$ $[M+Na]^+$ 305.1332, found 305.1336.

2-(2-Acetylphenethyl)-6-methyl-1,3,6,2-dioxazaborocane-4,8-dione (2ac)

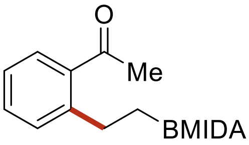

Prepared according to general procedure D using (*E*)-*N*-(4-methoxyphenyl)-1-phenylethan-1-imine (45.1 mg, 0.2 mmol) and 6-methyl-2-vinyl-1,3,6,2-dioxazaborocane-4,8-dione (110 mg, 0.6 mmol). Purification by column chromatography (CH_2Cl_2/MeOH 20:1) afforded the title compound as colorless oil (37.6 mg, 62%).

^{1}H NMR (400 MHz, $CDCl_3$): 7.69 (d, *J* = 7.7 Hz, 1H), 7.42 (t, *J* = 7.5 Hz, 1H), 7.33 – 7.22 (m, 2H), 3.90 (d, *J* = 16.3 Hz, 2H), 3.80 (d, *J* = 16.4 Hz, 2H), 3.12 (s, 3H), 2.88 – 2.78 (m, 2H), 2.57 (s, 3H), 1.06 – 0.97 (m, 2H) ppm.

^{13}C NMR (101 MHz, $CDCl_3$): δ 202.5 (C_q), 167.4 (C_q), 145.5 (C_q), 136.7 (C_q), 132.3 (CH), 131.4 (CH), 129.9 (CH), 125.9 (CH), 62.3 (CH_2), 61.6 (CH_2), 46.0 (CH_3), 29.9 (CH_3), 28.9 (CH_2) ppm.

IR (ATR): $\tilde{\nu}$ = 1769, 1745, 1682, 1339, 1301, 1251, 1109, 1026, 764, 600 cm^{-1}.

MS (ESI) *m*/*z* (relative intensity): 629 (100) $[2M+Na]^+$, 326 (95) $[M+Na]^+$, 304 (10) $[M+H]^+$.

HRMS (ESI): *m*/*z* calcd. for $C_{15}H_{18}NO_5BNa^+$ $[M+Na]^+$ 325.1207, found 325.1215.

6-(2-Acetylphenyl)hexyl 2-(4-isobutylphenyl)propanoate (2ad)

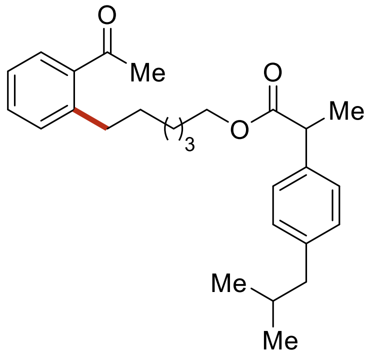

Prepared according to general procedure D using (*E*)-*N*-(4-methoxyphenyl)-1-phenylethan-1-imine (45.1 mg, 0.2 mmol) and hex-5-en-1-yl 2-(4-isobutylphenyl)propanoate (173 mg, 0.6 mmol) with 96 h of reaction time. Purification by column chromatography (hexane/EtOAc 9:1) afforded the title compound as colorless oil (74.5 mg, 92%).

^{1}H NMR (400 MHz, $CDCl_3$): δ 7.63 (dd, *J* = 7.8, 1.5 Hz, 1H), 7.38 (td, *J* = 7.5, 1.5 Hz, 1H), 7.26 (td, *J* = 7.1, 1.4 Hz, 2H), 7.23 – 7.17 (m, 2H), 7.08 (d, *J* = 8.1 Hz, 2H), 4.05 (t, *J* = 6.6 Hz, 2H), 3.68 (q, *J* = 7.1 Hz, 1H), 2.85 – 2.76 (m, 2H), 2.57 (s, 3H), 2.43 (d, *J* = 7.2 Hz, 2H), 1.83 (hept, *J* = 6.8 Hz, 1H), 1.62 – 1.45 (m, 7H), 1.38 – 1.21 (m, 4H), 0.88 (d, *J* = 6.5 Hz, 6H) ppm.

^{13}C NMR (101 MHz, $CDCl_3$): δ 202.3 (C_q), 175.0 (C_q), 142.9 (C_q), 140.5 (C_q), 138.4 (C_q), 131.4 (CH), 131.3 (CH), 129.4 (CH), 129.2 (CH), 127.3 (CH), 125.8 (CH), 64.9 (CH_2), 45.3 (CH), 45.2 (CH_2), 34.0 (CH_2), 31.8 (CH_2), 30.3 (CH_3), 30.1 (CH), 29.4 (CH_2), 28.6 (CH_2), 25.8 (CH_2), 22.5 (CH_3), 18.6 (CH_3) ppm.

IR (ATR): $\tilde{\nu}$ = 2955, 2924, 1735, 1688, 1466, 1377, 1352, 1249, 1092, 954 cm^{-1}.

MS (ESI) *m*/*z* (relative intensity): 839 (50) $[2M+Na]^+$, 431 (100) $[M+Na]^+$.

HRMS (ESI): *m*/*z* calcd. for $C_{27}H_{36}O_3Na^+$ $[M+Na]^+$ 431.2557, found 431.2560.

1-(2-(6-(((3*S*,8*S*,9*S*,10*R*,13*R*,14*S*,17*R*)-10,13-dimethyl-17-((*R*)-6-methylheptan-2-yl)-2,3,4,7,8,9,10,11,12,13,14,15,16,17-tetradecahydro-1*H*-cyclopenta[*a*]phenanthren-3-yl)oxy)hexyl)phenyl)ethan-1-one (2ae)

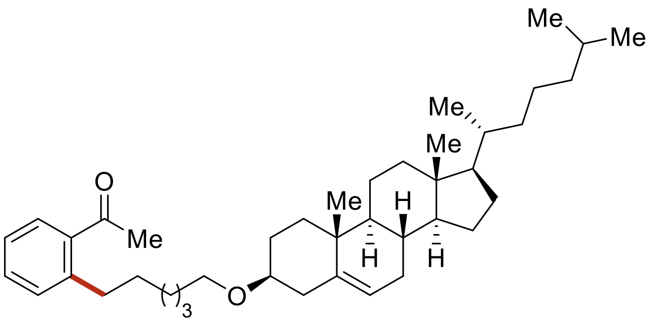

Prepared according to general procedure D using (*E*)-*N*-(4-methoxyphenyl)-1-phenylethan-1-imine (18.2 mg, 80 µmol) and the corresponding cholesterol-derived alkene (112.5 mg, 0.24 mmol) with 96 h of reaction time. Purification by column chromatography (hexane/EtOAc 9:1) afforded the title compound as colorless oil (19.1 mg, 41%).

^{1}H NMR (400 MHz, $CDCl_3$): δ 7.61 (dd, *J* = 8.0, 1.4 Hz, 1H), 7.38 (td, *J* = 7.5, 1.4 Hz, 1H), 7.24 (ddd, *J* = 7.6, 3.8, 1.5 Hz, 2H), 5.37 – 5.30 (m, 1H), 3.44 (td, *J* = 6.8, 1.6 Hz, 2H), 3.12 (tt, *J* = 11.2, 4.4 Hz, 1H), 2.87 – 2.79 (m, 2H), 2.57 (s, 3H), 2.35 (ddd, *J* = 13.2, 4.8, 2.2 Hz, 1H), 2.23 – 2.14 (m, 1H), 1.99 (tt, *J* = 14.9, 3.3 Hz, 2H), 1.91 – 1.79 (m, 3H), 1.61 – 1.51 (m, 9H), 1.51 – 1.41 (m, 4H), 1.40 – 1.34 (m, 6H), 1.27 – 1.24 (m, 4H), 1.17 – 1.03 (m, 6H), 0.99 (s, 3H), 0.91 (d, *J* = 6.5 Hz, 3H), 0.86 (dd, *J* = 6.6, 1.8 Hz, 6H), 0.67 (s, 3H) ppm.

^{13}C NMR (101 MHz, $CDCl_3$): δ 202.5 (C_q), 143.0 (C_q), 141.3 (C_q), 138.2 (C_q), 131.4 (CH), 131.3 (CH), 129.1 (CH), 125.8 (CH), 121.5 (CH), 79.1 (CH), 68.2 (CH_2), 56.9 (CH), 56.3 (CH), 50.4 (CH), 42.5 (C_q), 39.9 (CH_2), 39.7 (CH_2), 39.4 (CH_2), 37.5 (CH_2), 37.1 (C_q), 36.3 (CH_2), 35.9 (CH), 34.1 (CH_2), 32.1 (CH_2), 32.0 (CH_3), 31.9 (CH_2), 30.3 (CH_2), 30.1 (CH), 29.7 (CH_2), 28.6 (CH_2), 28.4 (CH_2), 28.2 (CH), 26.2 (CH_2), 24.4 (CH_2), 24.0 (CH_2), 23.0 (CH_3), 22.7 (CH_3), 21.2 (CH_2), 19.5 (CH_3), 18.9 (CH_3), 12.0 (CH_3) ppm.

IR (ATR): $\tilde{\nu}$ = 2853, 2851, 1728, 1687, 1463, 1467, 1354, 1248, 1107, 757 cm^{-1}.

MS (ESI) *m*/*z* (relative intensity): 627 (25) $[M+K]^+$, 611 (100) $[M+Na]^+$.

HRMS (ESI): *m*/*z* calcd. for $C_{41}H_{64}O_2Na^+$ $[M+Na]^+$ 611.4799, found 611.4795.

(*E*)-1-(5-Methoxy-2-((4-methoxyphenyl)((4-methoxyphenyl)imino)methyl)phenyl)octan-1-one (3a)

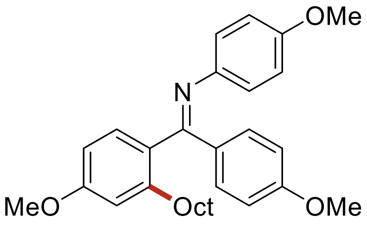

Prepared according to general procedure D using *N*,1,1-tris(4-methoxyphenyl)methanimine (27.9 mg, 0.08 mmol) and 1-octene (20 µL, 0.13 mmol). Purification by column chromatography (hexane/EtOAc 10:1) afforded the title compound as yellow oil (21.7 mg, 59%).

^{1}H NMR (400 MHz, $CDCl_3$) δ 7.72 – 7.61 (m, 2H), 6.96 (d, *J* = 9.1 Hz, 1H), 6.92 – 6.82 (m, 2H), 6.74 – 6.63 (m, 6H), 3.84 (s, 3H), 3.79 (s, 3H), 3.72 (s, 3H), 2.35 – 2.11 (m, 2H), 1.27 – 1.25 (m, 7H), 1.17 – 1.11 (m, 5H), 0.85 (t, *J* = 7.1 Hz, 3H) ppm.

^{13}C NMR (101 MHz, $CDCl_3$) δ 167.1 (C_q), 161.6 (C_q), 159.6 (C_q), 156.0 (C_q), 144.3 (C_q), 142.1 (C_q), 133.5 (C_q), 130.8 (CH), 130.5 (CH), 129.1 (C_q), 123.0 (CH), 114.4 (CH), 113.7 (CH), 113.6 (CH), 110.8 (CH), 55.5 (CH_3), 55.4 (CH_3), 55.2 (CH_3), 33.5 (CH_2), 32.0 (CH_2), 30.1 (CH_2), 29.9 (CH_2), 29.4 (CH_2), 29.3 (CH_2), 22.8 (CH_2), 14.2 (CH_3) ppm.

IR (ATR): $\tilde{\nu}$ = 2955, 2924, 2853, 1604, 1501, 1464, 1440, 1239, 1165, 873 cm^{-1}.

MS (ESI) *m*/*z* (relative intensity): 460 (100) $[M+H]^+$.

HRMS (ESI): *m*/*z* calcd. for $C_{30}H_{38}NO_3^+$ $[M+H]^+$ 460.2846, found 460.2843.

Performing the reaction using the conditions for the thermal hydroarylation employing ruthenium-catalysis reported by Ackermann in 2013,[10] product **3a** was obtained in 38% yield along with 26% of the bis-alkylated product (arene (0.2 mmol), 1-octene (0.32 mmol), $[RuCl_2(p\text{-cymene})]_2$ (5 mol%), toluene (1.0 mL), 100 °C, 24 h).

(*E*)-4-(((4-(*tert*-Butyl)phenyl)imino)(4-(diethylamino)phenyl)methyl)-*N*,*N*-diethyl-3-octylaniline (3b)

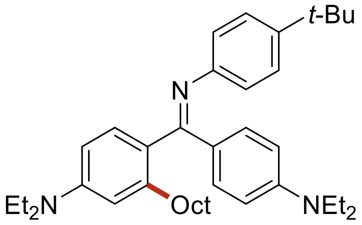

Prepared according to general procedure D using 4,4'-(((4-(*tert*-butyl)phenyl)imino)methylene)bis(*N*,*N*-diethylaniline) (69.4 mg, 0.2 mmol) and 1-octene (50 µL, 0.32 mmol). Purification by column chromatography (hexane/EtOAc 4:1) afforded the title compound as orange oil (64.8 mg, 58%).

^{1}H NMR (400 MHz, $CDCl_3$) δ 7.61 (d, *J* = 8.7 Hz, 2H), 7.15 – 7.07 (m, 2H), 6.89 (d, *J* = 8.4 Hz, 1H), 6.71 (d, *J* = 8.6 Hz, 2H), 6.61 (d, *J* = 8.8 Hz, 2H), 6.50 – 6.39 (m, 2H), 3.39 (q, *J* = 7.3 Hz, 4H), 3.33 (q, *J* = 7.1 Hz, 4H), 2.35 – 2.28 (m, 1H), 2.20 – 2.13 (m, 1H), 1.31 – 1.26 (m, 6H), 1.24 (s, 9H), 1.19 – 1.13 (m, 18H), 0.88 – 0.84 (m, 3H) ppm.

^{13}C NMR (101 MHz, $CDCl_3$) δ 168.1 (C_q), 149.4 (C_q), 149.2 (C_q), 147.7 (C_q), 145.2 (C_q), 141.5 (C_q), 131.1 (CH), 130.7 (CH), 128.5 (C_q), 125.0 (CH), 124.7 (C_q), 121.7 (CH), 112.1 (CH), 110.7 (CH), 109.2 (CH), 44.6 (CH_2), 44.4 (CH_2), 34.3 (CH_2), 34.0 (CH_2), 32.0 (CH_2), 31.6 (CH_3), 30.5 (CH_2), 29.9 (CH_2), 29.6 (CH_2), 29.4 (CH_2), 22.8 (C_q), 14.2 (CH_3), 12.8 (CH_3), 12.7 (CH_3) ppm.

IR (ATR): $\tilde{\nu}$ = 2964, 2925, 1742, 1579, 1375, 1356, 1317, 1266, 1149, 1013 cm^{-1}.

MS (ESI) *m*/*z* (relative intensity): 1139 (20) $[2M+H]^+$, 568 (100) $[M+H]^+$.

HRMS (ESI): *m*/*z* calcd. for $C_{39}H_{58}N_3^+$ $[M+H]^+$ 568.4625, found 568.4625.

Performing the reaction using the conditions for the thermal hydroarylation employing ruthenium-catalysis reported by Ackermann in 2013,[10] product **3b** was obtained in 39% yield along with 27% of the bis-alkylated product (arene (0.2 mmol), 1-octene (0.32 mmol), $[RuCl_2(p\text{-cymene})]_2$ (5 mol%), toluene (1.0 mL), 100 °C, 24 h).

1-(2-Methyl-6-octylphenyl)-3,4-dihydroisoquinoline (3c)

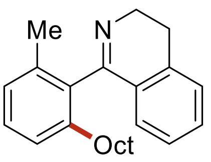

Prepared according to general procedure D using 1-(*o*-tolyl)-3,4-dihydroisoquinoline (44.3 mg, 0.2 mmol) and 1-octene (95 µL, 0.6 mmol). Purification by column chromatography (hexane/EtOAc 6:1) afforded the title compound as brown oil (19.3 mg, 29%).

^{1}H NMR (400 MHz, $CDCl_3$) δ 7.34 (td, *J* = 7.4, 1.3 Hz, 1H), 7.26 – 7.19 (m, 2H), 7.16 – 7.04 (m, 3H), 6.84 (dd, *J* = 7.8, 1.3 Hz, 1H), 3.93 (q, *J* = 7.4 Hz, 2H), 2.87 (t, *J* = 7.5 Hz, 2H), 2.49 – 2.29 (m, 2H), 2.11 (s, 3H), 1.62 – 1.49 (m, 1H), 1.43 – 1.35 (m, 1H), 1.25 – 1.10 (m, 10H), 0.85 (t, *J* = 7.0 Hz, 3H) ppm.

^{13}C NMR (101 MHz, $CDCl_3$) δ 168.0 (C_q), 140.8 (C_q), 138.1 (C_q), 137.4 (C_q), 135.8 (C_q), 130.9 (CH), 129.8 (C_q), 128.0 (CH), 127.6 (CH), 127.5 (CH), 127.2 (CH), 126.8 (CH), 126.7 (CH), 47.5 (CH_2), 33.5 (CH_2), 32.0 (CH_2), 31.3 (CH_2), 29.8 (CH_2), 29.4 (CH_2), 29.3 (CH_2), 26.1 (CH_2), 22.8 (CH_2), 19.6 (CH_3), 14.3 (CH_3) ppm.

IR (ATR): $\tilde{\nu}$ = 2923, 2853, 1743, 1619, 1463, 1239, 781, 759, 741, 703 cm^{-1}.

MS (ESI) *m/z* (relative intensity): 334 (100) $[M+H]^+$.

HRMS (ESI): *m/z* calcd. for $C_{24}H_{32}N^+$ $[M+H]^+$ 334.2529, found 334.2530.

2-(2-Octylphenyl)pyridine (3d)

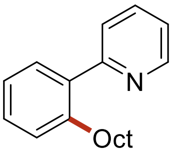

Prepared according to general procedure D using 2-phenylpyridine (31.0 mg, 0.2 mmol) and 1-octene (95 µL, 0.6 mmol). Purification by column chromatography (hexane/EtOAc 10:1) afforded the title compound as yellow oil (38.0 mg, 71%).

^{1}H NMR (300 MHz, $CDCl_3$) δ 8.68 (ddd, *J* = 4.9, 1.9, 0.9 Hz, 1H), 7.74 (td, *J* = 7.7, 1.8 Hz, 1H), 7.40 – 7.27 (m, 5H), 7.26 – 7.22 (m, 1H), 2.75 – 2.61 (m, 2H), 1.44 (t, *J* = 7.8 Hz, 2H), 1.33 – 1.10 (m, 10H), 0.85 (t, *J* = 6.8 Hz, 3H).

^{13}C NMR (101 MHz, $CDCl_3$) δ 160.5 (C_q), 149.3, (CH) 140.9 (C_q), 140.5 (C_q), 136.2 (CH), 129.9 (CH), 129.8 (CH), 128.4 (CH), 125.9 (CH), 124.3 (CH), 121.7 (CH), 33.1 (CH_2), 32.0 (CH_2), 31.4 (CH_2), 29.6 (CH_2), 29.4 (CH_2), 29.3 (CH_2), 22.8 (CH_2), 14.2 (CH_3) ppm.

IR (ATR): $\tilde{\nu}$ = 2926, 2923, 1586, 1562, 1469, 1377, 1149, 1023, 989, 794 cm^{-1}.

MS (ESI) *m/z* (relative intensity): 268 (100) $[M+H]^+$.

HRMS (ESI): *m/z* calcd. for $C_{19}H_{26}N^+$ $[M+H]^+$ 268.2054, found 268.2060.

2-(4-Methoxy-2-octylphenyl)pyrimidine (3e)

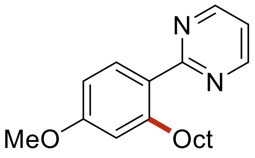

Prepared according to general procedure D using 2-(4-methoxyphenyl)pyrimidine (37.2 mg, 0.2 mmol) and 1-octene (95 µL, 0.6 mmol). Purification by column chromatography (hexane/EtOAc 10:1) afforded the title compound as yellow oil (10.7 mg, 18%).

^{1}H NMR (400 MHz, $CDCl_3$) δ 8.80 (d, *J* = 4.9 Hz, 2H), 7.77 (d, *J* = 9.3 Hz, 1H), 7.16 (t, *J* = 4.9 Hz, 1H), 6.87 – 6.80 (m, 2H), 3.85 (s, 3H), 3.03 – 2.93 (m, 2H), 1.54 – 1.40 (m, 2H), 1.33 – 1.17 (m, 10H), 0.86 (t, *J* = 7.0 Hz, 3H).

^{13}C NMR (101 MHz, $CDCl_3$) δ 167.8 (C_q), 160.6 (C_q), 156.9 (CH), 144.5 (C_q), 132.5 (CH), 130.8 (C_q), 118.2 (CH), 116.1 (CH), 111.2 (CH), 55.4 (CH_3), 34.0 (CH_2), 32.0 (CH_2), 31.7 (CH_2), 29.7 (CH_2), 29.5 (CH_2), 29.4 (CH_2), 22.8 (CH_2), 14.3 (CH_3).

IR (ATR): $\tilde{\nu}$ = 2956, 2922, 1607, 1567, 1551, 1512, 1466, 1410, 1161, 875 cm^{-1}.

MS (ESI) *m*/*z* (relative intensity): 312 (20) $[M+Na]^+$, 299 (10) $[M+H]^+$.

HRMS (ESI): *m*/*z* calcd. for $C_{19}H_{27}N_2O^+$ $[M+H]^+$ 299.2117, found 299.2118.

Performing the reaction using the conditions for the thermal hydroarylation employing ruthenium-catalysis reported by Ackermann in 2013,[10] product **3e** was obtained in 19% yield along with 12% of the bis-alkylated product (arene (0.2 mmol), 1-octene (0.32 mmol), $[RuCl_2(p\text{-cymene})]_2$ (5 mol%), toluene (1.0 mL), 100 °C, 24 h).

2-Octylbenzaldehyde (3f)

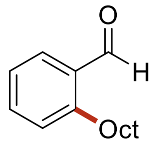

Prepared according to general procedure D using (*E*)-*N*-(4-methoxyphenyl)-1-phenylmethanimine (42.4 mg, 0.2 mmol) and 1-octene (95 µL, 0.6 mmol). Purification by column chromatography (hexane) afforded the title compound as colorless oil (6.6 mg, 15%).

^{1}H NMR (300 MHz, $CDCl_3$) δ 10.30 (s, 1H), 7.83 (dd, *J* = 7.7, 1.5 Hz, 1H), 7.50 (td, *J* = 7.5, 1.6 Hz, 1H), 7.41 – 7.29 (m, 1H), 7.26 (s, 1H), 3.07 – 2.96 (m, 2H), 1.61 (p, *J* = 7.4 Hz, 2H), 1.28 (d, *J* = 8.3 Hz, 10H), 0.94 – 0.82 (m, 3H) ppm.

^{13}C NMR (101 MHz, $CDCl_3$) δ 192.4 (CH), 146.1 (CH_q), 133.9 (CH), 133.8 (CH_q), 131.4 (CH), 131.1 (CH), 126.5 (CH), 32.6 (CH_2), 32.6 (CH_2), 32.0 (CH_2), 29.7 (CH_2), 29.6 (CH_2), 29.4 (CH_2), 22.8 (CH_2), 14.2 (CH_3) ppm.

IR (ATR): $\tilde{\nu}$ = 2956, 2924, 1698, 1600, 1574, 1465, 1454, 1207, 1190, 754 cm^{-1}.

MS (ESI) *m*/*z* (relative intensity): 242 (100) $[M+Na]^+$.

HRMS (ESI): *m*/*z* calcd. for $C_{15}H_{22}ONa^+$ $[M+H]^+$ 241.1563, found 241.1563.

5 Mechanistic Studies

5.1 Detection of Free *p*-Cymene

Reaction monitoring in the presence of alkene

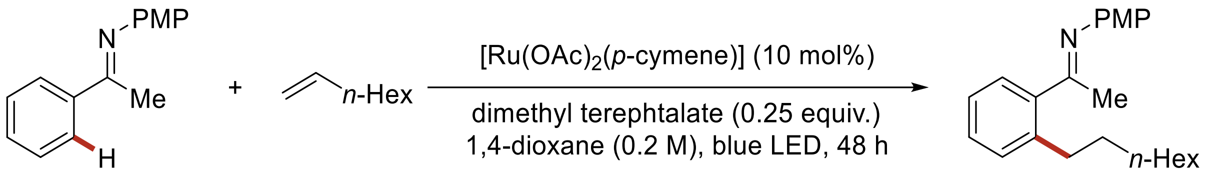

1a **2a'**

Following general procedure D, an oven-dried 10 mL glass vial containing a teflon-coated magnetic stirring bar was filled with ketimine **1a** (90.1 mg, 0.4 mmol, 1.0 equiv.) and [Ru(OAc)$_2$(*p*-cymene)] (14.1 mg, 40 µmol, 10 mol%). The vial was transferred into a N_2-filled glovebox before 1-octene (0.19 mL, 0.12 mmol, 3.0 equiv.), dimethyl terephthalate as internal standard (19.4 mg, 0.1 mmol, 0.25 equiv.) and 1,4-dioxane (2.0 mL) were added. The vial was sealed and transferred out of the glovebox. Subsequently, the reaction mixture was stirred for 48 h under blue light. At t = 1, 2, 3, 5, 7, 9, 22, 32 and 44 h aliquots (0.1 mL) were taken which were diluted with $CDCl_3$ and analyzed by ^{1}H NMR afterwards.

Entry	Time (h)	Free *p*-cymene (%)[a,b]	Yield **2a‘** (%)[a]
1	0	0	0
2	1	12	0
3	2	15	3
4	3	20	5
5	5	40	9
6	7	50	12
7	9	63	21
8	22	83	46
9	32	97	74
10	44	99	89

[a] Determined by ^{1}H NMR with dimethyl terephthalate as the internal standard. [b] Percentage of detected *p*-cymene relative to the used amount of [Ru(OAc)$_2$(*p*-cymene)].

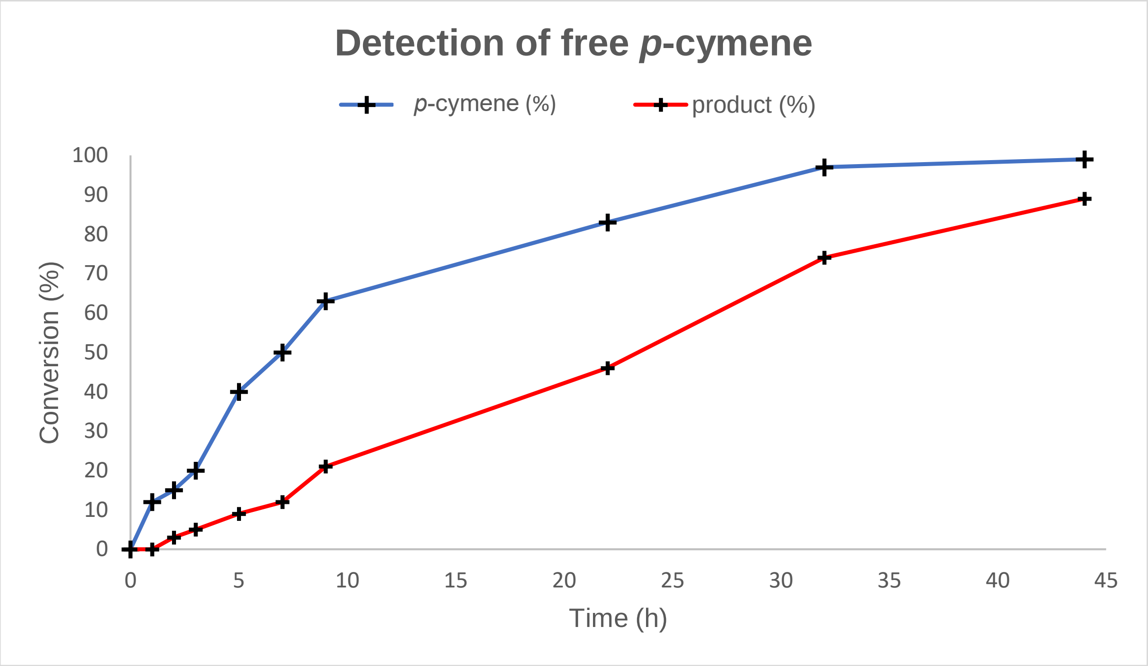

Reaction monitoring in the absence of alkene

The reaction monitoring was performed under the same conditions as described above but in the absence of the terminal alkene. Following this procedure, the amount of free *p*-cymene was detected using ^{1}H NMR spectroscopy with dimethyl terephthalate as internal standard.

Entry	Time (h)	Free *p*-cymene (%)[a]
1	0	0
2	1	10
3	2	15
4	3	20
5	5	29
6	7	40
7	9	47
8	22	77
9	32	85
10	44	99

[a] Percentage of detected *p*-cymene relative to the used amount of [Ru(OAc)$_2$(*p*-cymene)] determined by ^{1}H NMR using dimethyl terephthalate as the internal standard.

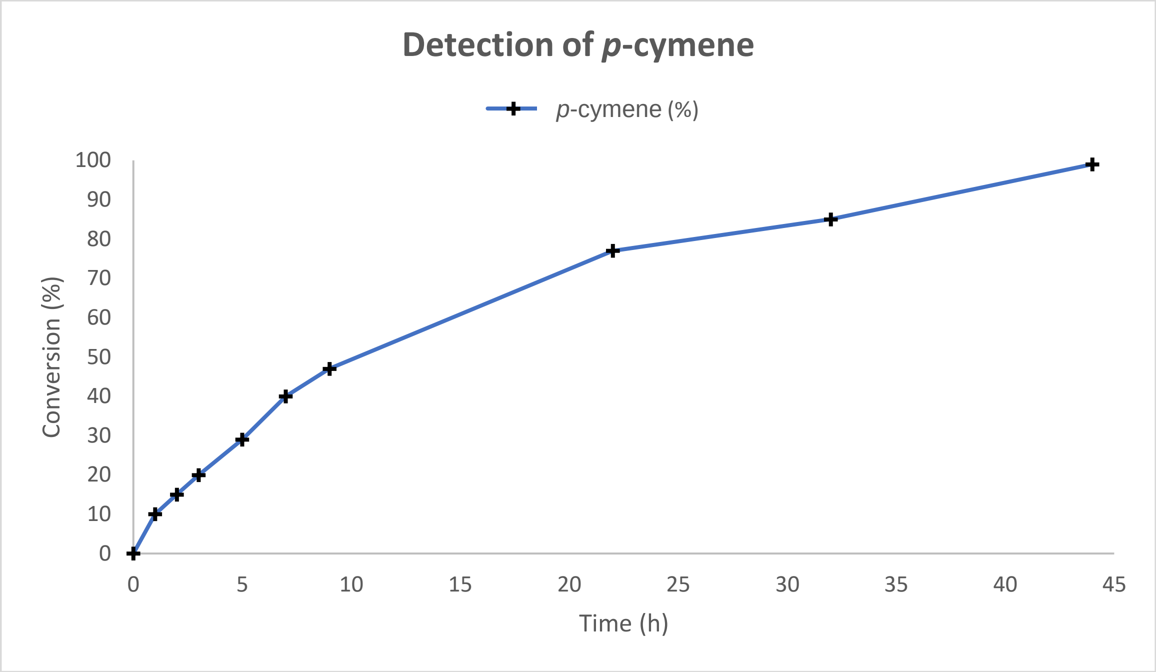

*In situ monitoring using 2-phenyl-pyridine **3d***

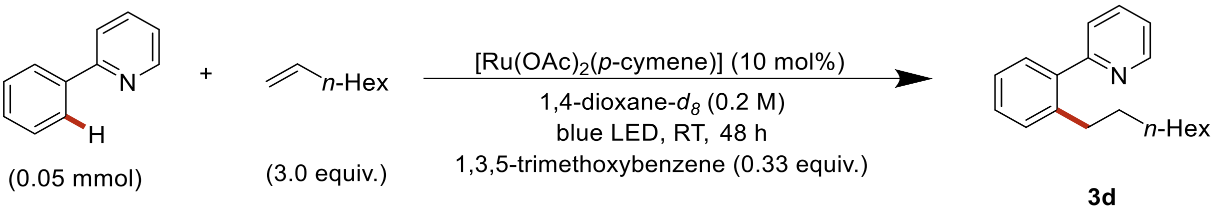

3d

Following general procedure D, an oven-dried 10 mL glass vial containing a teflon-coated magnetic stirring bar was filled with [Ru(OAc)$_2$(*p*-cymene)] (3.53, 10 µmol, 10 mol%). The vial was transferred into a N_2-filled glovebox before 2-phenyl-pyridine (0.014 mL, 0.1 mmol, 1.0 equiv.), 1-octene (0.047 mL, 0.3 mmol, 3.0 equiv.), 1,3,5-trimethoxybenzene as internal standard (5.55 mg, 0.033 mmol, 0.33 equiv.) and 1,4-dioxane-d_8 (0.5 mL) were added. The reaction was stirred for 5min, transferred into a J-Young NMR Tube and the reaction mixture was stirred for 48 h under blue light. At t = 2, 4, 6, 8, 10, 12, 24, 30, 36 and 48 h, a ^{1}H NMR analysis was performed.

Entry	Time (h)	Free *p*-cymene (%)[a,b]	Yield **4d** (%)[a]
1	0	0	0
2	2	15	0
3	4	35	1
4	6	45	1
5	8	55	2
6	10	65	2
7	12	70	3
8	24	100	32
9	30	100	45
10	36	100	57
11	48	100	70

[a] Determined by ^{1}H NMR with 1,3,5-trimethoxybenzene as the internal standard. [b] Percentage of detected *p*-cymene relative to the used amount of [Ru(OAc)$_2$(*p*-cymene)].

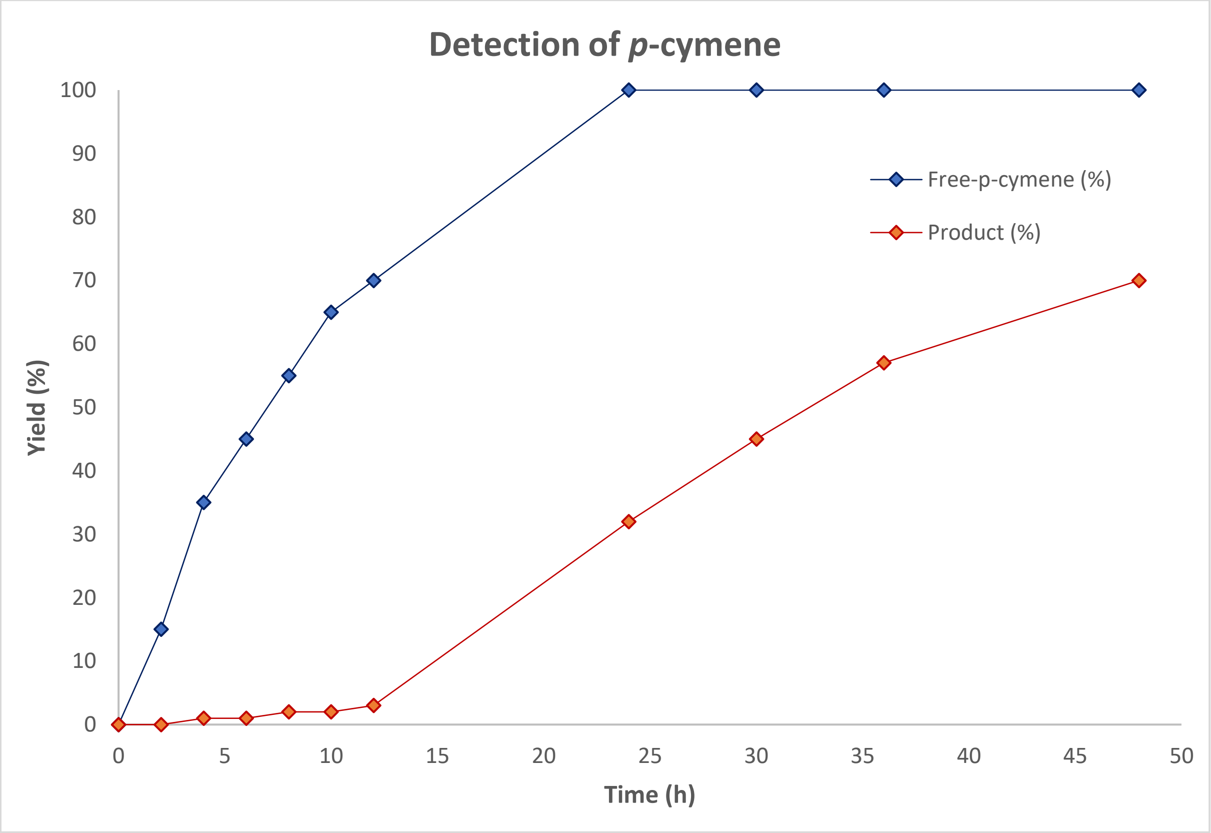

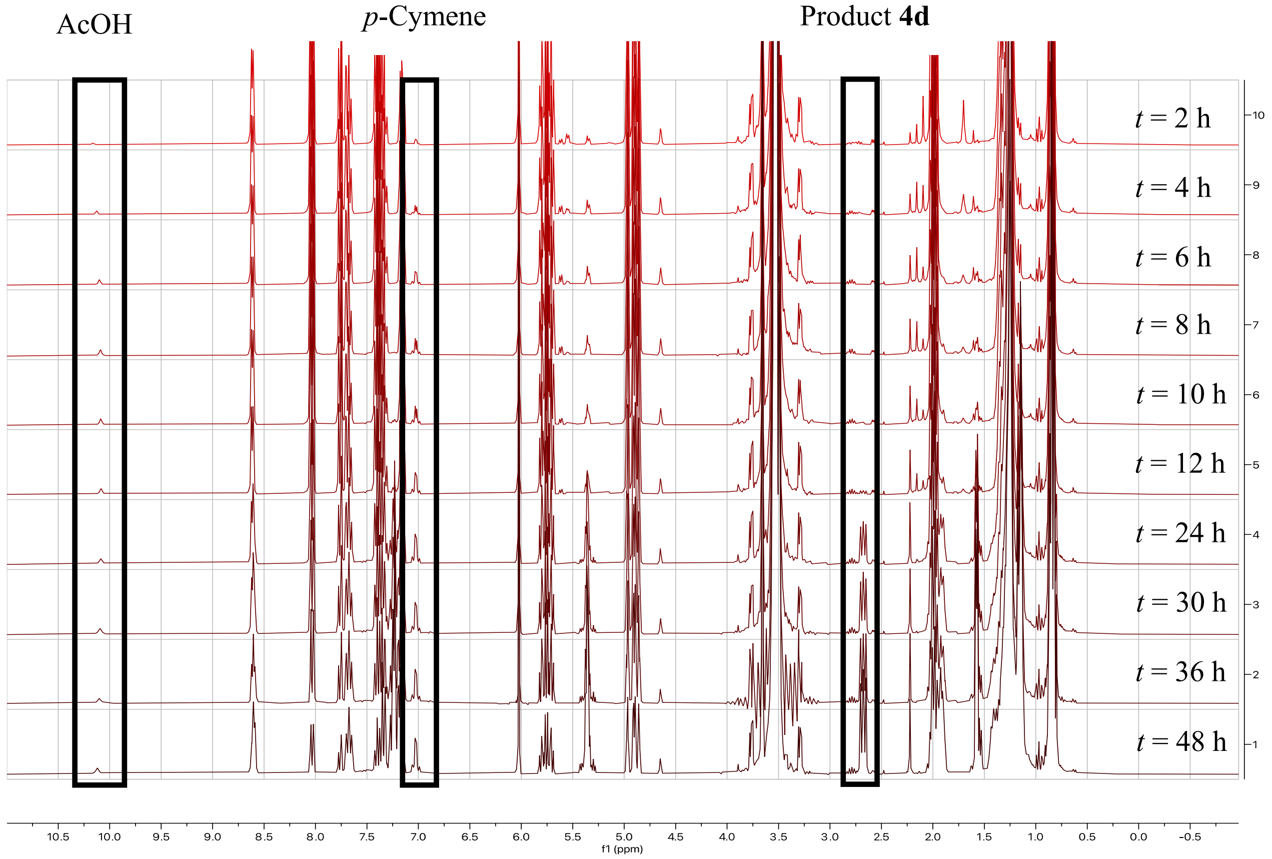

Irradiation of [Ru(OAc)₂(p-cymene)] in 1,4-dioxane

An oven-dried 10 mL glass vial containing a teflon-coated magnetic stirring bar was filled with [Ru(OAc)$_2$(*p*-cymene)] (14.1 mg, 40 µmol, 1.0 equiv.) and 1,3,5-trimethoxybenzene (2.2 mg, 0.33 equiv.). The vial was transferred into a N_2-filled glovebox and 1,4-dioxane (2 mL) was added. The resulting mixture was stirred for 5 min, transferred into a J-Young NMR Tube containing few drops of benzene-d_8 and the reaction mixture was stirred for 24 h under blue light. At t = 2, 4, 6, 8, 10, 12, and 24 h, an ^{1}H NMR analysis was performed.

Entry	Time (h)	Free *p*-cymene (%)[a]
1	0	0
2	2	15
3	4	35
4	6	45
5	8	55
6	10	65
7	12	70
8	24	100

[a] Percentage of detected *p*-cymene relative to the used amount of [Ru(OAc)$_2$(*p*-cymene)] determined by ^{1}H NMR using 1,3,5-trimethoxybenzene as the internal standard.

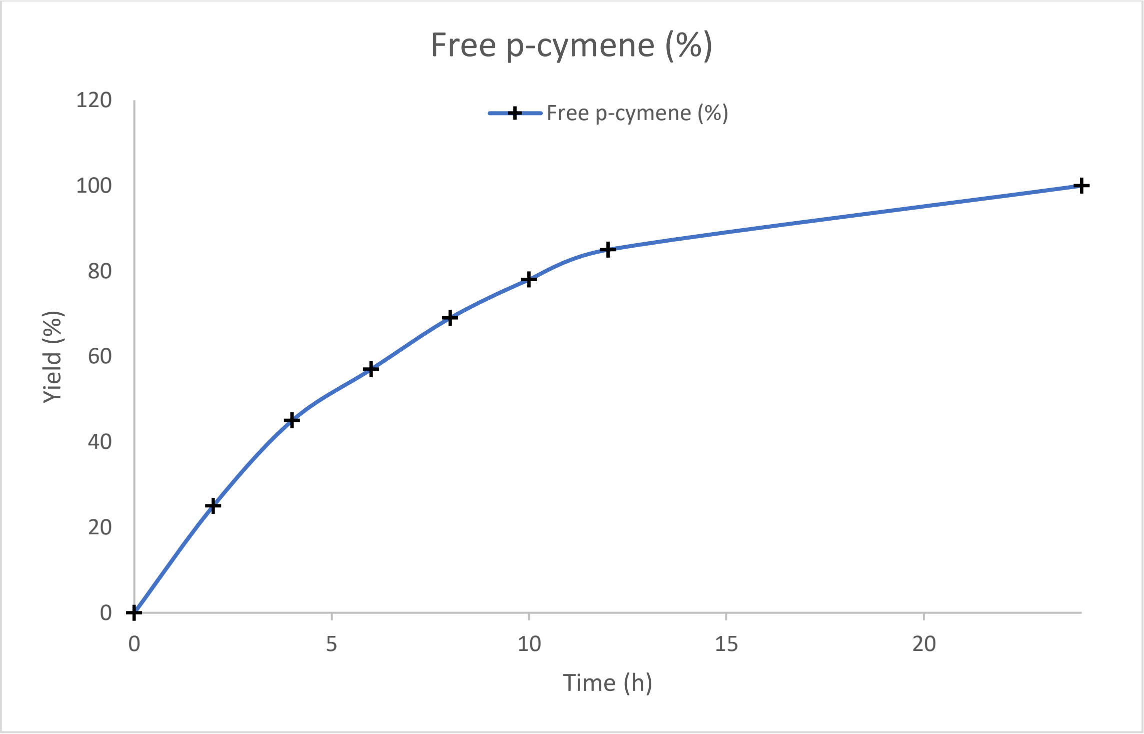

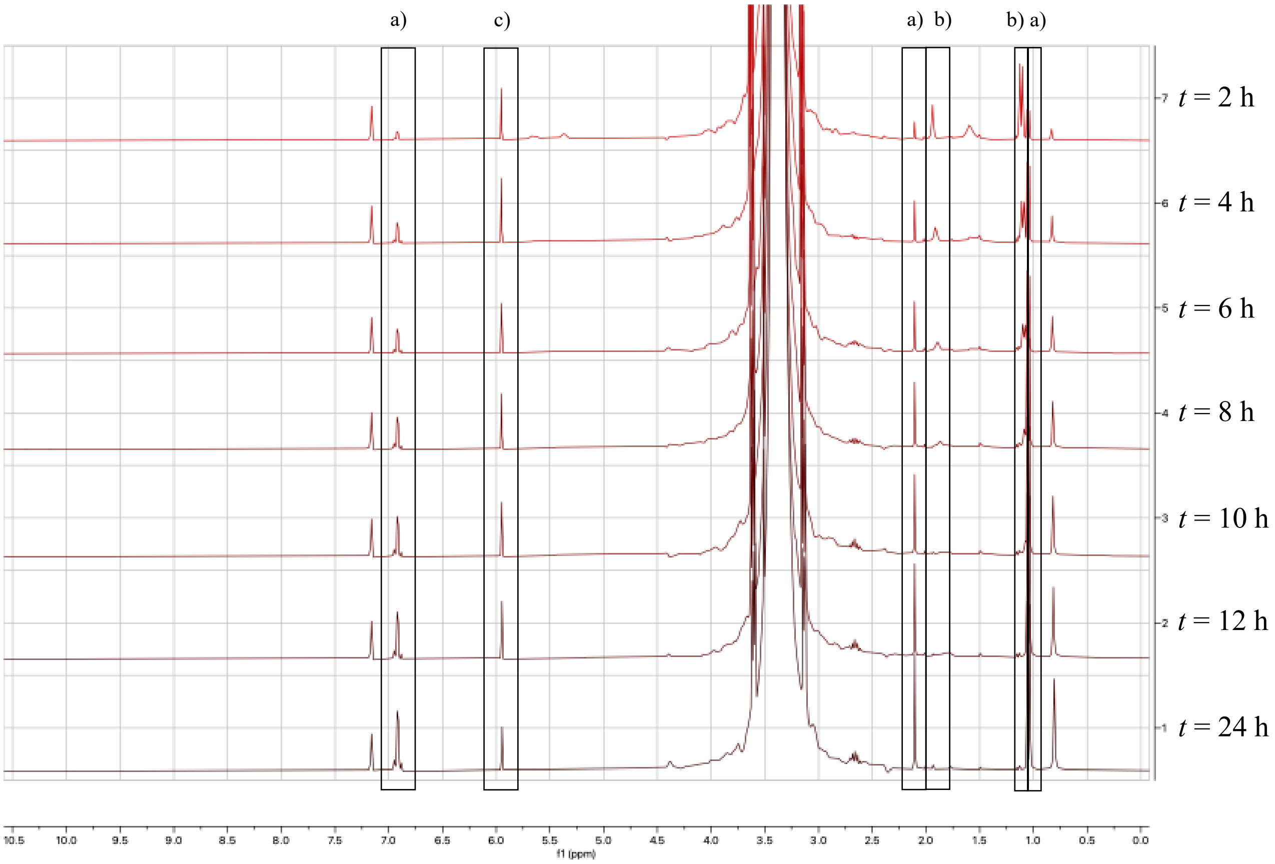

a) *p*-cymene (released), b) *p*-cymene (coordinated), c) 1,3,5-trimethoxybenzene.

5.2 Inhibition Experiment with *p*-Cymene

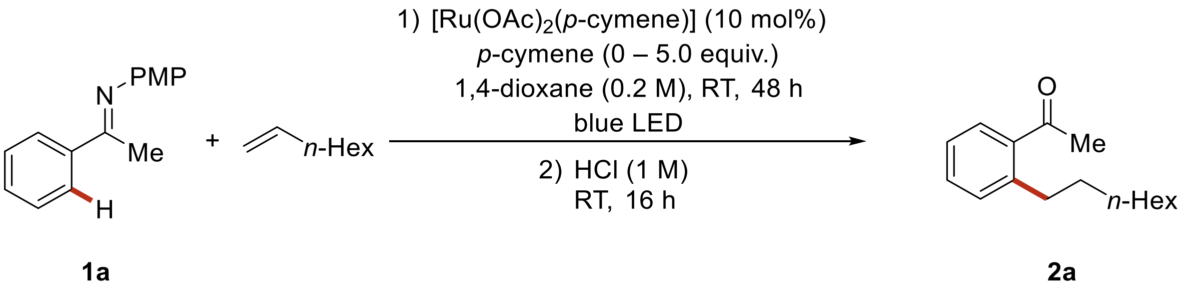

	Yield of 2a:
Without additional *p*-cymene:	**85%**
With 0.5 equiv. *p*-cymene:	**0%**
With 5.0 equiv. *p*-cymene:	**0%**

The reaction was carried out according to general procedure D with additional *p*-cymene (15.6 µL, 0.1 mmol or 156 µL, 1.0 mmol, respectively). After 48 h under blue light irradiation, the reactions were quenched by the addition of HCl (1 M, 3 mL). After extraction with CH_2Cl_2 (3 x 2 mL), 1,3,5-

trimethoxybenze was added as internal standard. After drying over Na_2SO_4, filtration and removal of all volatiles, the obtained residue was used to determine the yield of **2a** via ^{1}H NMR spectroscopy.

5.3 Sequential Experiments

Irradiation of the Ru complex, then addition of reagents

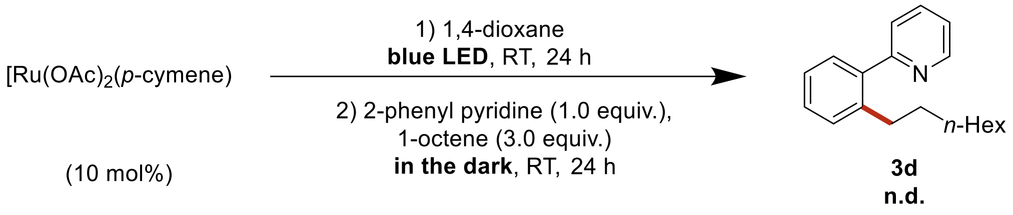

An oven-dried 10 mL glass vial containing a teflon-coated magnetic stirring bar was filled with [Ru(OAc)$_2$(*p*-cymene)] (7.1 mg, 20 µmol, 10 mol%). The vial was transferred into a N_2-filled glovebox before the 1,4-dioxane (1.0 mL) was added. The vial was transferred out of the glovebox and the obtained solution was stirred for 24 h under blue light irradiation. Then, the vial was transferred into the glovebox before 2-phenyl-pyridine (0.028 mL, 0.2 mmol, 1.0 equiv.) and 1-octene (0.097 mL, 0.6 mmol, 3.0 equiv.) were added. The vial was transferred out of the glovebox and the reaction mixture was stirred in the dark for 48 h. Analysis of the crude reaction mixture showed that no desired product has been formed.

Stirring of arene and the Ru complex in the dark, then addition of alkene and irradiation

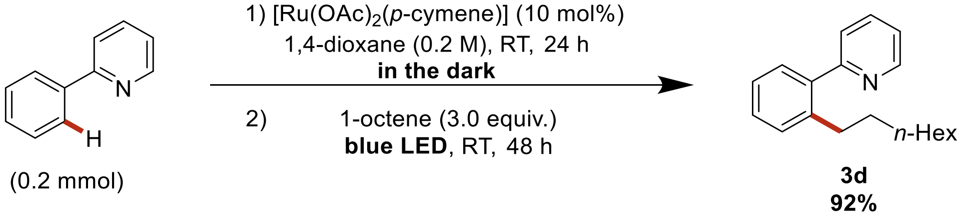

An oven-dried 10 mL glass vial containing a teflon-coated magnetic stirring bar was filled with [Ru(OAc)$_2$(*p*-cymene)] (7.1 mg, 20 µmol, 10 mol%). The vial was transferred into a N_2-filled glovebox before the addition of of 2-phenyl-pyridine (0.028 mL, 0.2 mmol, 1.0 equiv.) and 1,4-dioxane (1.0 mL). Then, the vial was transferred into the glovebox and the solution was stirred in the dark for 24 h. The vial was transferred into the glovebox before 1-octene (0.097 mL, 0.6 mmol, 3.0 equiv.) was added. The vial was transferred out of the glovebox and stirred under blue light irradiation for 48 h. The solvent was

evaporated under reduced pressure. Purification of the residue by column chromatography on silica gel (hexane/EtOAc) afforded the corresponding product **3d** in 92% yield.

Irradiation of arene and the Ru complex without alkene

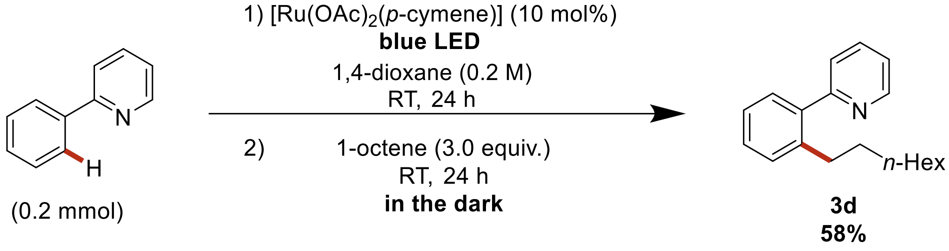

To an oven-dried 10 mL glass vial containing a teflon-coated magnetic stirring bar, [Ru(OAc)$_2$(*p*-cymene)] (7.1 mg, 20 µmol, 10 mol%) was added. The vial was transferred into a N_2-filled glovebox and 2-phenyl-pyridine (0.2 mmol, 1.0 equiv.) and 1,4-dioxane (1.0 mL) were added. The vial was closed, sealed with parafilm, and transferred out of the glovebox. Subsequently, the reaction mixture was stirred for 24 h under blue light irradiation (two Kessil PR160L lamps, 456 nm). Afterwards, the vial was transferred into a N_2-filled glovebox and 1-Octene (0.6 mmol, 3.0 equiv.) was added. The vial was closed, sealed with parafilm, transferred out of the glovebox and stirred in the dark for 24 h. At the end of the reaction, the volatiles were removed under reduced pressure. Purification of the residue by column chromatography on silica gel (hexane/EtOAc) afforded the corresponding product **3d** in 58% yield.

Reduced irradiation time

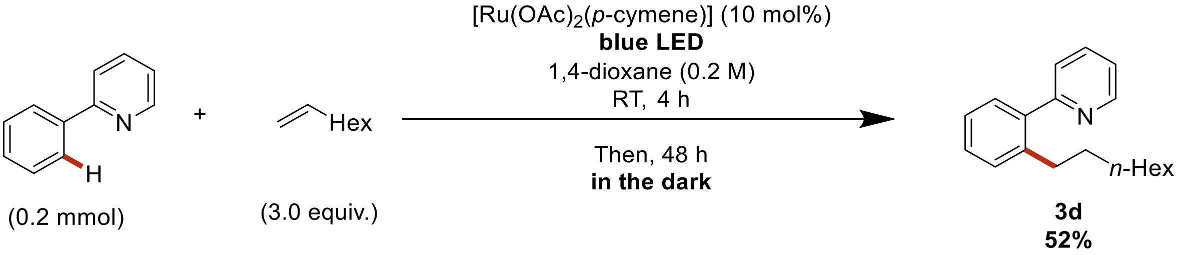

To an oven-dried 10 mL glass vial containing a teflon-coated magnetic stirring bar [Ru(OAc)$_2$(*p*-cymene)] (7.1 mg, 20 µmol, 10 mol%) was added. The vial was transferred into a N_2-filled glovebox before 2-phenyl-pyridine (0.2 mmol, 1.0 equiv.), the terminal alkene (0.6 mmol, 3.0 equiv.) and 1,4-dioxane (1.0 mL) were added. The vial was closed, sealed with parafilm, and transferred out of the glovebox. Subsequently, the reaction mixture was stirred for 4 h under blue light irradiation (two Kessil

PR160L lamps, 456 nm). Afterwards, the reaction was stirred in the dark for 48 h. At the end of the reaction, the volatiles were removed under reduced pressure. Purification of the residue by column chromatography on silica gel (hexane/EtOAc) afforded the corresponding product **3d** in 52% yield.

5.4 On-Off Experiment

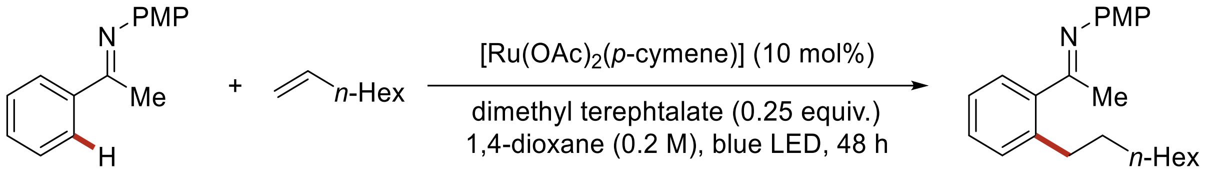

1a **2a'**

An oven-dried 10 mL glass vial equipped with a teflon-coated magnetic stirring bar was charged with ketimine **1a** (90.1 mg, 0.4 mmol, 1.0 equiv.) and [Ru(OAc)$_2$(*p*-cymene)] (14.1 mg, 10 mol%). The vial was transferred into a N_2-filled glovebox before 1-octene (0.12 mmol, 3.0 equiv.), dimethyl terephthalate (19.4 mg, 0.1 mmol, 0.25 equiv.) and 1,4-dioxane (2.0 mL) were added. The vial was sealed and transferred out of the glovebox. The reaction was then stirred using the typical setup for photochemical reactions. The light was turn on or off as specified in the table below and aliquots (0.1 mL) were taken after the time indicated (6, 12, 24, 36, 48, 60 h). The solvent was removed, and the residue was dissolved in $CDCl_3$ (0.6 mL) before the resulting solution was analyzed via ^{1}H NMR spectroscopy.

Entry	Time (h)	Light (On/Off)	Yield **2a'** (%)[a]
1	0	---	0
2	6	Off	0
3	12	On	18
4	24	Off	27
5	36	On	47
6	48	Off	52
7	60	On	67

[a] ^{1}H NMR yield determined using dimethyl terephthalate as the internal standard.

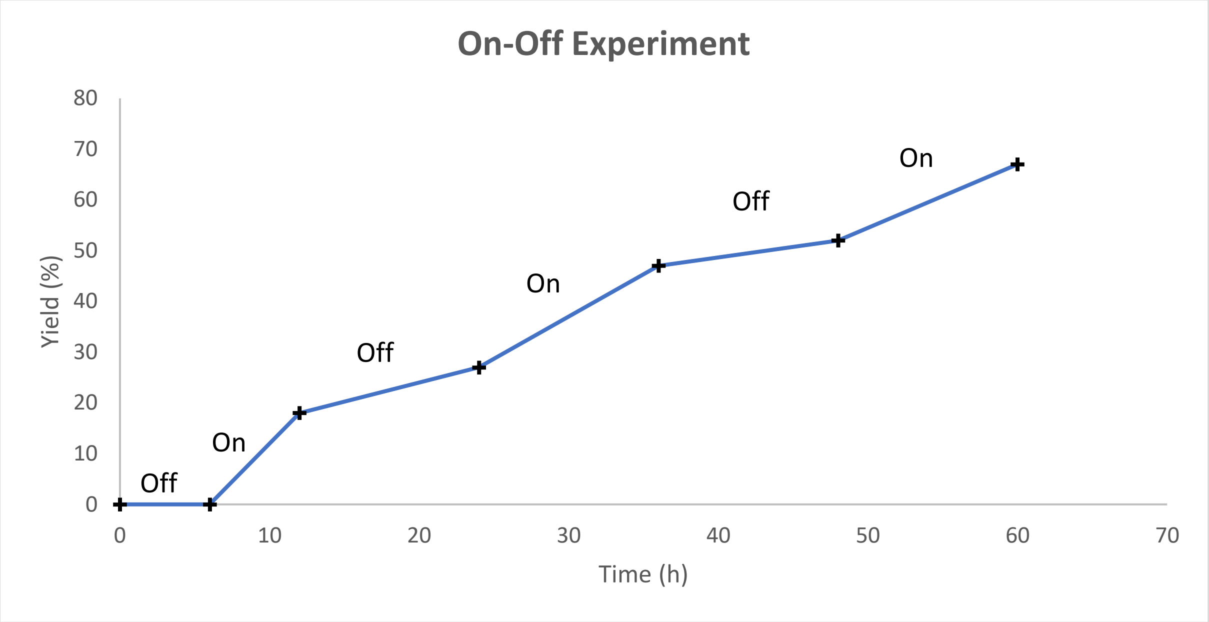

5.5 UV-Vis Absorption Measurements

The following solutions were prepared in a N_2 filed glovebox:

1. Ketimine **1a** (0.2 mmol) in 10 mL of 1,4-dioxane
2. Catalyst [Ru(OAc)$_2$(*p*-cymene)] (0.02 mmol) in 1 mL of 1,4-dioxane
3. Catalyst [Ru(OAc)$_2$(*p*-cymene)] (0.02 mmol) in 1 mL of 1,4-dioxane
4. Ruthenacycle **Ru-1** (0.02 mmol) + KOAc (0.04 mmol) in 1 mL of 1,4-dioxane
5. Ruthenacycle **Ru-1** (0.02 mmol) + KOAc (0.04 mmol) in 1 mL of 1,4-dioxane
6. Ketimine **1a** (0.2 mmol), catalyst [Ru(OAc)$_2$(*p*-cymene)] (0.02 mmol), 1-octene (0.6 mmol) in 10 mL of 1,4-dioxane
7. Ketimine **1a** (0.2 mmol), catalyst [Ru(OAc)$_2$(*p*-cymene)] (0.02 mmol), 1-octene (0.6 mmol) in 10 mL of 1,4-dioxane

Solutions **2**, **4** and **6** were removed from the glovebox and irradiated with 450 nm LEDs overnight. The solutions were again introduced into the glovebox and 50 µL of each solution was transferred to a 10 mL volumetric flask and 1,4-dioxane was added to complete the final volume of 10 mL. Then 3 mL of those solutions were transferred from the volumetric flask to the cuvette. The cuvettes were removed from the glovebox, and the samples were subjected to UV/Vis absorption measurements.

Solution **1** was immediately subjected to measurements by preparing samples in the same way as described above.

Solutions **3**, **5**, and **7** were left overnight at room temperature in the glovebox, and measurements were performed in the same way as described above.

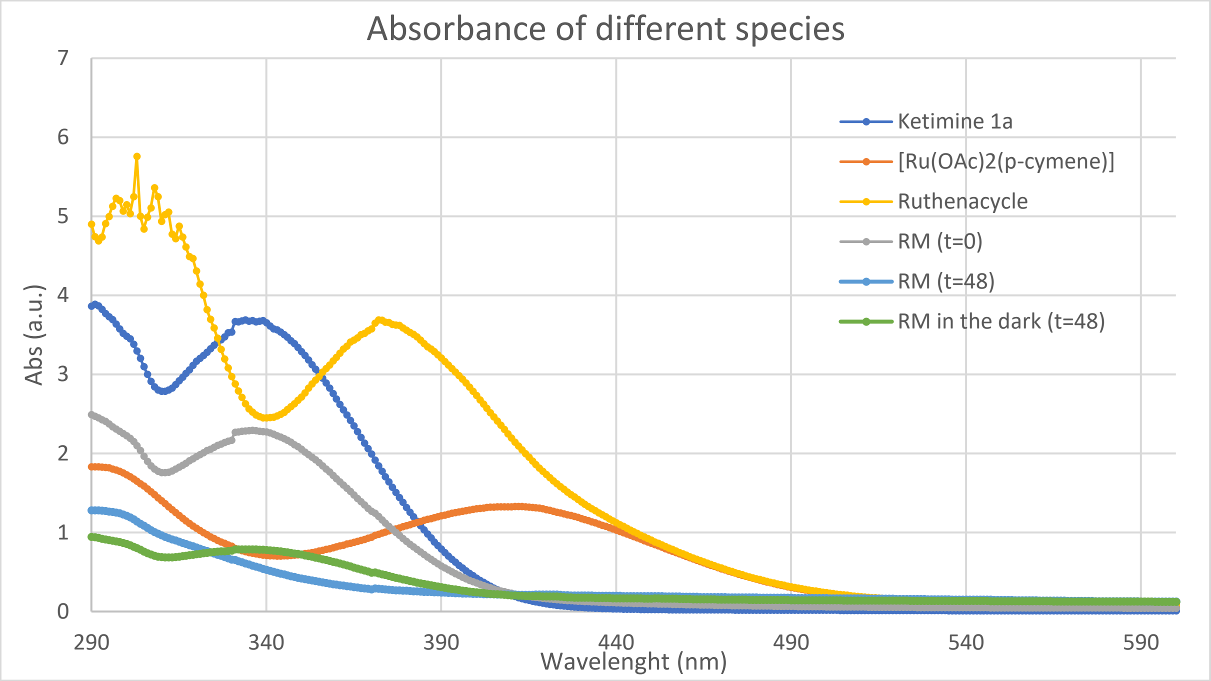

RM = reaction mixture

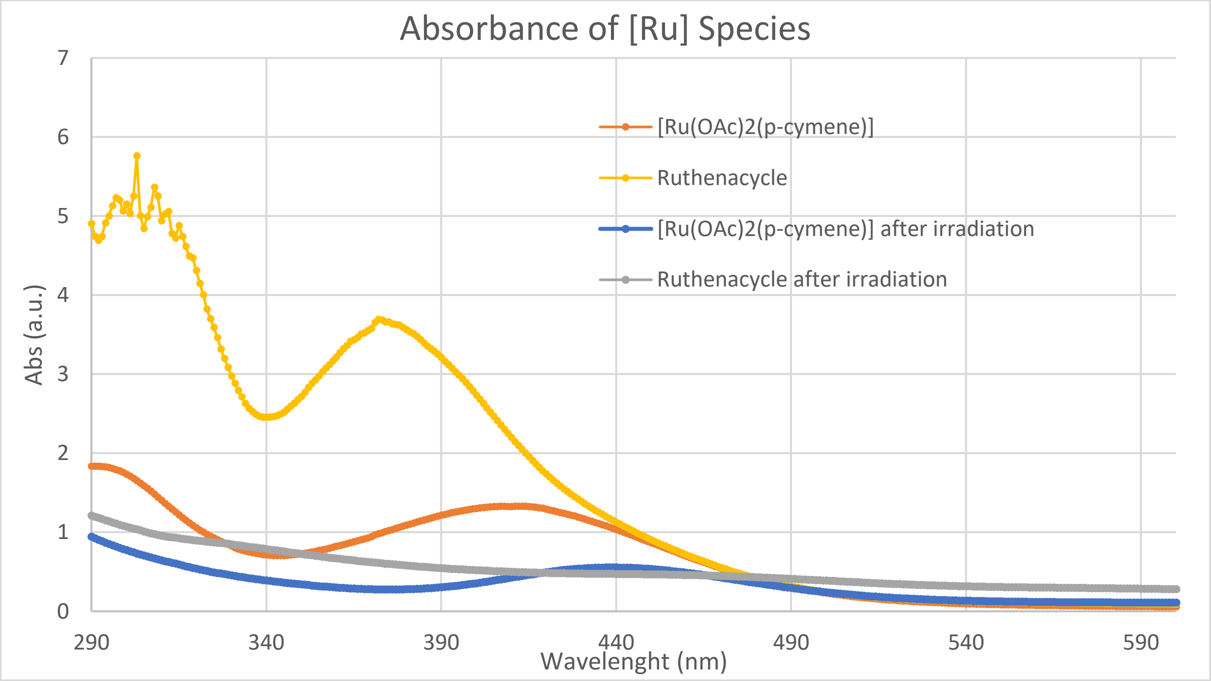

5.6 Intermolecular Competition Experiment

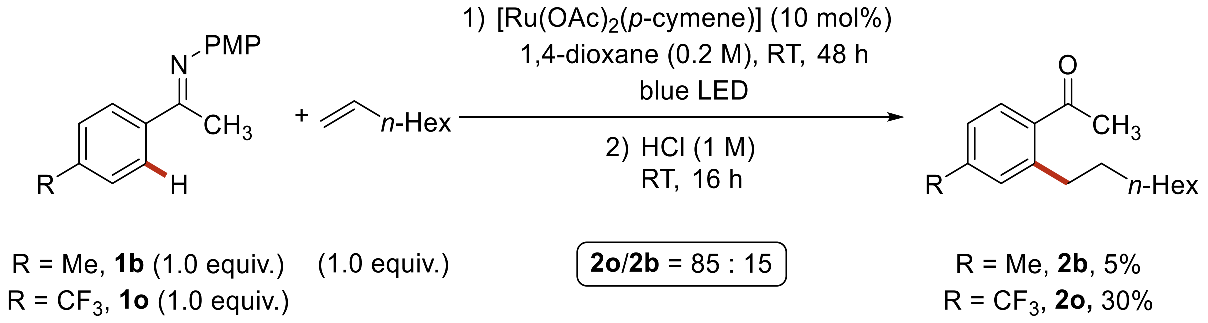

To an oven-dried 10 mL glass vial containing a teflon-coated magnetic stirring bar the methyl-substituted ketimine **1b** (47.9 mg, 0.2 mmol, 1.0 equiv.), trifluoromethyl-substituted ketimine **1o** (58.7 mg, 0.2 mmol, 1.0 equiv.) and [Ru(OAc)$_2$(*p*-cymene)] (7.1 mg, 20 µmol, 10 mol%) were added. The vial was transferred into a N_2-filled glovebox before 1-octene (31 µL, 0.2 mmol, 1.0 equiv.) and 1,4-dioxane (2.0 mL) were added. The vial was closed, sealed with parafilm and transferred out of the glovebox. Subsequently, the reaction mixture was stirred for 48 h under blue light irradiation. Afterwards, the reaction was quenched by the addition of HCl (1 M, 3 mL). The resulting mixture was stirred vigorously for 16 h at ambient temperature. After extraction with CH_2Cl_2 (3 x 2 mL), drying over Na_2SO_4 and filtration, the solvent was removed under reduced pressure. The obtained residue was analyzed by ^{1}H NMR spectroscopy using dibromomethane as internal standard to determine the product ratio.

5.7 Ruthenacycle Ru-1 as Catalyst

Ruthenacycle **Ru-1** was prepared following a procedure reported by Dixneuf. Analytical data match with those in the literature.[7]

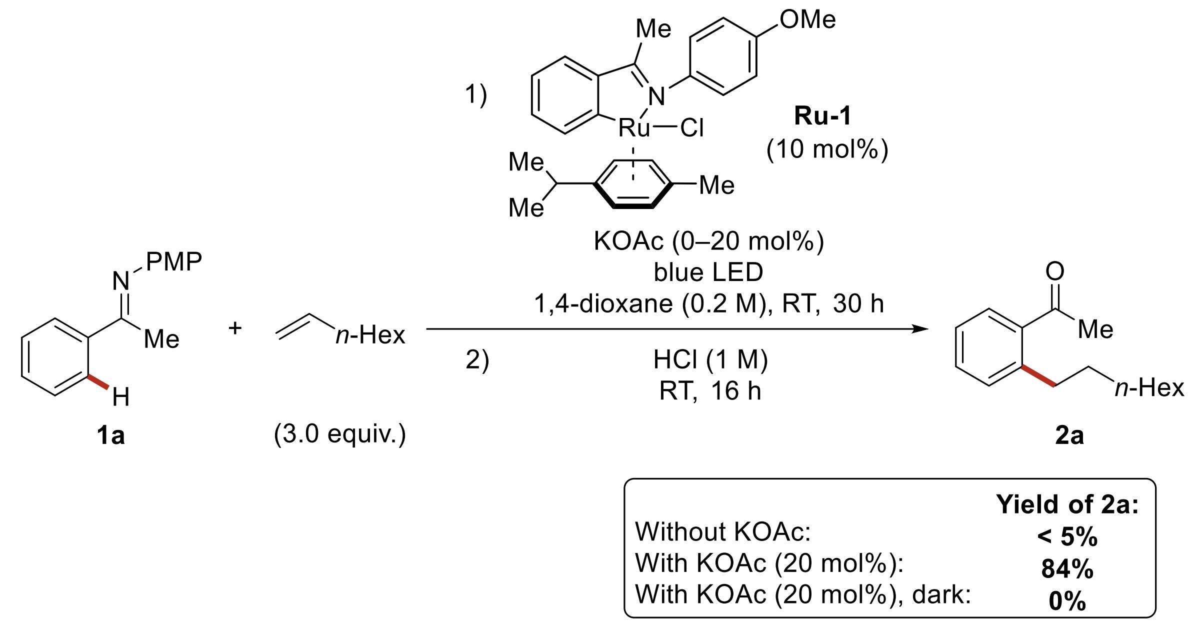

To an oven-dried 10 mL glass vial containing a teflon-coated magnetic stirring bar ketimine **1a** (45.1 mg, 0.2 mmol, 1.0 equiv.) was added. The vial was transferred into a N_2 filled glovebox and ruthenacycle **5** (9.9 mg, 20 µmol, 10 mol%), 1-octene (95 µL, 0.6 mmol, 3.0 equiv.), potassium acetate (0 mol% or 20 mol%), and 1,4-dioxane (1 mL) were added. The vial was closed and transferred out of the glovebox. Subsequently, the reaction mixture was stirred for 48 h under blue light irradiation. Afterwards, the reaction was quenched by the addition of HCl (1 M, 3 mL). The resulting mixture was stirred vigorously for 16 h at ambient temperature. After extraction with CH_2Cl_2 (3 x 2 mL), drying over Na_2SO_4 and filtration, the solvent was removed under reduced pressure. Purification of the residue by column chromatography on silica gel (hexane/EtOAc 20:1) afforded the corresponding product **2a** in the presence of potassium acetate (39.0 mg, 84%). In contrast, no product formation was observed in the absence of potassium acetate.

5.8 Deuterium-Labelling Experiment

Deuterium exchange experiment in the absence of alkene

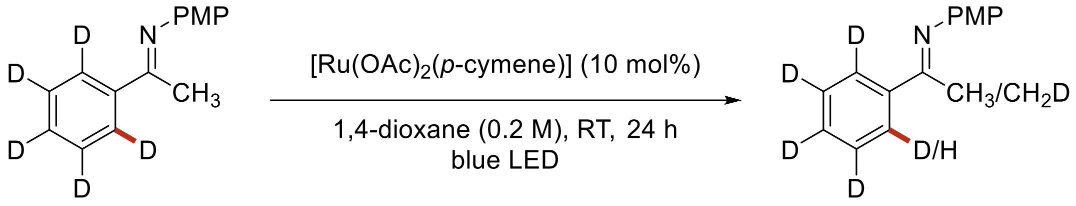

1a-d_5 **1a'-d_5**

To an oven-dried 10 mL glass vial containing a teflon-coated magnetic stirring bar imine [D_5]-**1a** (11.5 mg, 50 µmol, 1.0 equiv.) and [Ru(OAc)$_2$(*p*-cymene)] (1.8 mg, 5 µmol, 10 mol%) were added. The vial was transferred into a N_2-filled glovebox before 1,4-dioxane (0.25 mL) was added. The vial was closed, sealed with parafilm and transferred out of the glovebox. Subsequently, the reaction mixture was stirred for 24 h under blue light irradiation. Afterwards, the solvent was removed under reduced pressure and the crude product was analyzed by ^{1}H NMR spectroscopy.

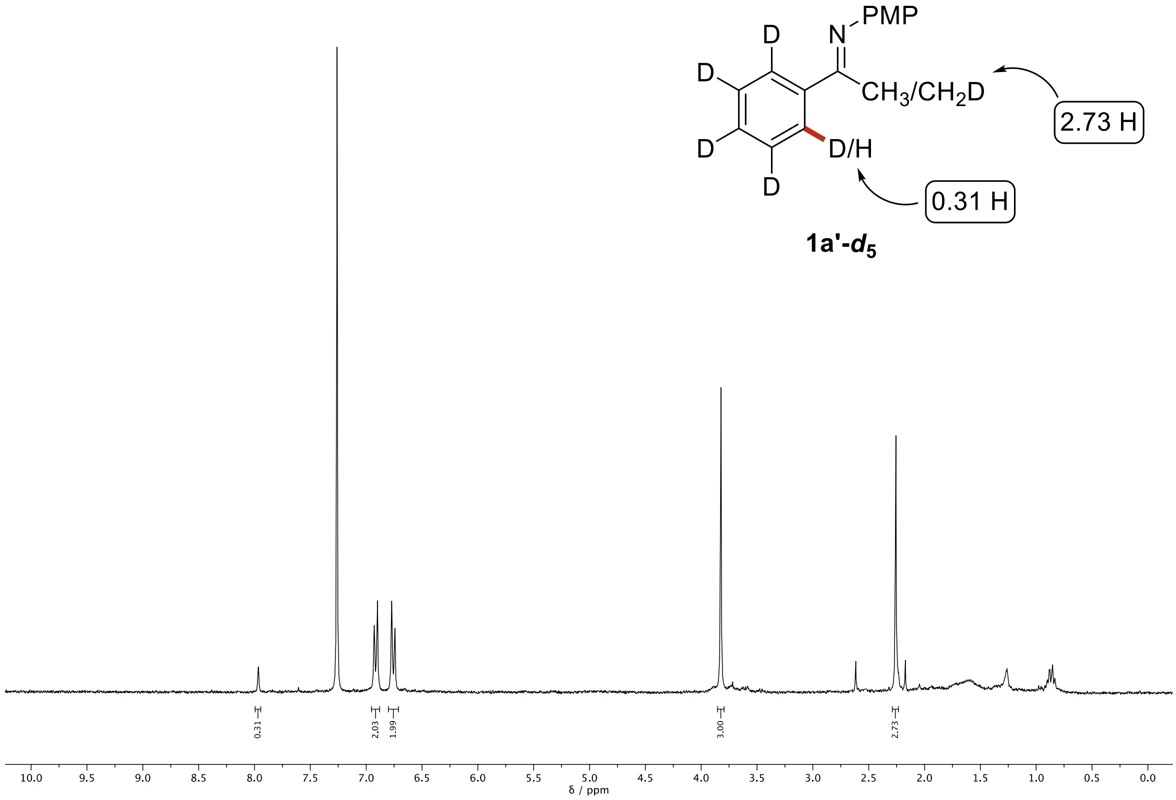

Deuterium-labelling experiment in the presence of an alkene

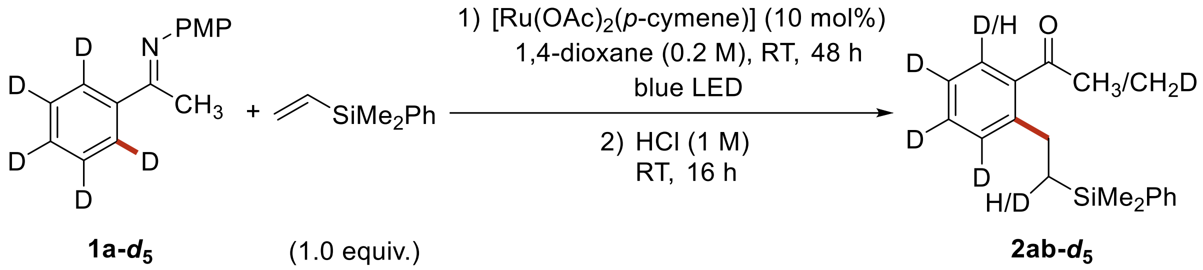

A oven-dried 10 mL glass vial containing a teflon-coated magnetic stirring bar was charged with imine **1a-*d*$_5$** (46.1 mg, 0.2 mmol, 1.0 equiv.) and [Ru(OAc)$_2$(*p*-cymene)] (7.1 mg, 20 µmol 10 mol%) before the vial was transferred into a N_2-filled glovebox. Dimethyl(phenyl)(vinyl)silane (37 µL, 0.2 mmol, 1.0 equiv.) and 1,4-dioxane (1.0 mL) were added afterwards. The vial was closed, sealed with parafilm and transferred out of the glovebox. Subsequently, the reaction mixture was stirred for 48 h under blue light irradiation. Afterwards, the reaction was quenched by the addition of HCl (1 M, 3 mL), and the resulting mixture was stirred vigorously for 16 h at ambient temperature. After extraction with CH_2Cl_2 (3 x 2 mL), drying over Na_2SO_4 and filtration, the solvent was removed under reduced pressure. Purification of the residue by column chromatography on silica gel (hexane/EtOAc 10:1) afforded product **2ac-*d*$_5$** as yellow oil (47.1 mg, 82%).

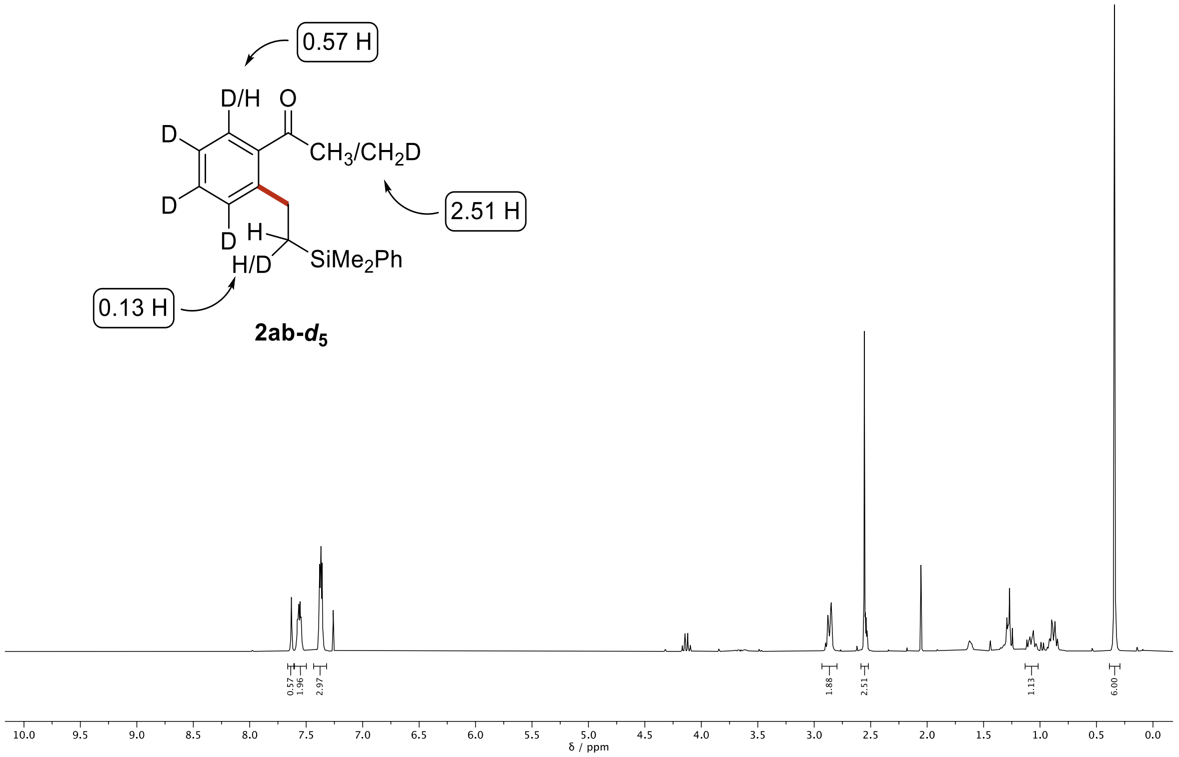

5.9 Determination of Quantum Yield

The quantum yield was determined in slight variation to procedure recently employed by Ackermann.[8] All preparations were carried out in the dark.

Preparation of potassium ferrioxalate solution:

In a volumetric flask, solid potassium ferrioxalate (295 mg) and conc. H_2SO_4 (140 µL) were diluted with H_2O to a finale volume of 50 mL.

Preparation of buffer solution:

In a volumetric flask, NaOAc (4.95 g) and conc. H_2SO_4 (1 mL) were diluted with H_2O to a finale volume of 100 mL.

Using the same setup as for catalytic reactions 2 mL of the potassium ferrioxalate solution were irradiated for 10 s. The sample solution was added to of 4 mL of the buffer solution containing 1,10-phenanthroline (2 mg). The solution was diluted with H_2O to a finale volume of 10 mL. The resulting solution was stored in the dark for 1 h. Subsequently the absorbance of this solution was determined at 510 nm ($\epsilon = 11100\ \mathrm{M^{-1}cm^{-1}}$).[9] The same procedure was followed for a nonirradiated sample.

Calculation Number of Photons:

Abs of Fe^{2+} (at 510 nm) = 2.7450 (after irradiation of 10 s)

Abs of Fe^{2+} (at 510 nm) = 0.1316 (no irradiation)

Abs of Fe^{2+} (at 510 nm) = 2.7450-0.1315 = 2.6134

$$[\mathrm{Fe^{2+}}] = \frac{\text{Abs of Fe}^{2+}\text{ (at 510 nm)}}{\epsilon_{510\,nm} \cdot l} = \frac{2.6134}{11100\ \mathrm{M^{-1}cm^{-1}} \cdot 1\ \mathrm{cm}} = 2.3758 \cdot 10^{-4}\ \mathrm{M}$$

$$n_{(\mathrm{Fe^{2+}})} = 2.3758 \cdot 10^{-4} \cdot 0.010\ \mathrm{L} = 2.3758 \cdot 10^{-6}\ \mathrm{mol}$$

with quantum yield of 0.9 for the absorption of Fe^{3+}:

$$n_{(\mathrm{photons})} = \frac{n_{(\mathrm{Fe^{2+}})}}{\Phi} = \frac{2.3758 \cdot 10^{-6}}{0.9} = 2.6398 \cdot 10^{-6}\ \mathrm{mol}$$

$$n_{(\mathrm{photons/s})} = 2.6398 \cdot 10^{-7}\ \mathrm{mol/s}$$

The initial rate of the alkylation was determined to be $3.08381 \cdot 10^{-9}$ mol/s.

$$\text{Quantum Yield} = \frac{n_{(\mathrm{product/s})}}{n_{(\mathrm{photons/s})}} = \frac{3.08381\ x\ 10^{-9}\ \mathrm{mol/s}}{2.6398\ x\ 10^{-7}\ \mathrm{mol/s}} = 0.01168 = \mathbf{1.2\%}$$

Determination Initial Rate:

Product formation was monitored by ^{1}H NMR spectroscopy using dibromomethane as internal standard.

Time (s)	1800	3600	5400	7200	9000	10800
Yield (%)	1.27	2.16	3.49	4.61	6.91	7.91
$n_{(product)} \cdot 10^{-5}$ (mol)	0.508	0.864	1.396	1.844	2.764	3.164

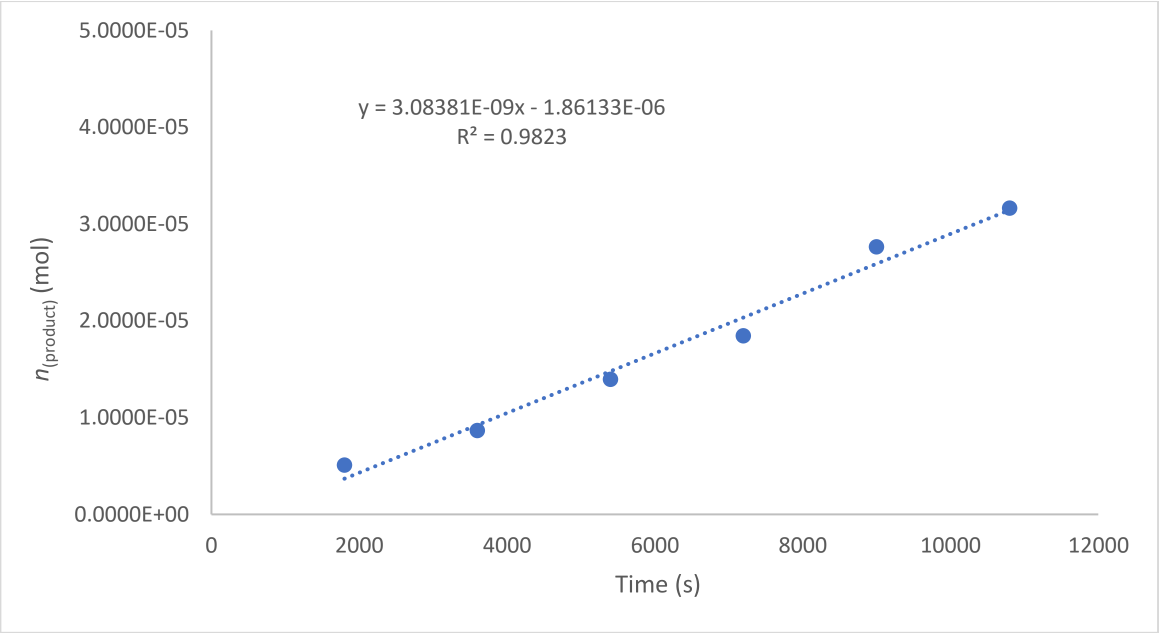

6 References

[1] M. Chang, W. Li, X. Zhang, *Angew. Chem. Int. Ed.* **2011**, *50*, 10679–10681.

[2] F.-M. Gautier, S. Jones, S. J. Martin, *Org. Biomol. Chem.* **2009**, *7*, 229–231.

[3] J. Wu, J. H. Barnard, Y. Zhang, D. Talwar, C. M. Robertson, J. Xiao, *Chem. Commun.* **2013**, *49*, 7052–7054.

[4] B. A. Suslick, T. D. Tilley, *Org. Lett.* **2021**, *23*, 1495–1499.

[5] B. J. Fallon, E. Derat, M. Amatore, C. Aubert, F. Chemla, F. Ferreira, A. Perez-Luna, M. Petit, *J. Am. Chem. Soc.* **2015**, *137*, 2448–2451.

[6] W. Guo, L. Cheng, G. Ma, W. Tong, F. Wu, *Org. Lett.* **2022**, *24*, 1796–1801.

[7] B. Li, C. Darcel, T. Roisnel, P. H. Dixneuf, *J. Organomet. Chem.* **2015**, *793*, 200–209.

[8] I. Choi, V. Müller, G. Lole, R. Köhler, V. Karius, W. Viöl, C. Jooss, L. Ackermann, *Chem. Eur. J.* **2020**, *26*, 3509–3514.

[9] H. J. Kuhn, S. E. Braslavsky, R. Schmidt, *Pure Appl. Chem.* **2004**, *76*, 2105–2146.

[10] M. Schinkel, I. Marek, L. Ackermann, *Angew. Chem. Int. Ed.* **2013**, *52*, 3977–3980.

7 NMR Spectra

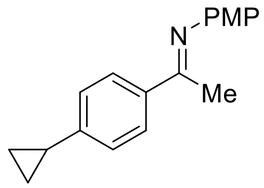

1c

^{1}H NMR (300 MHz, $CDCl_3$)

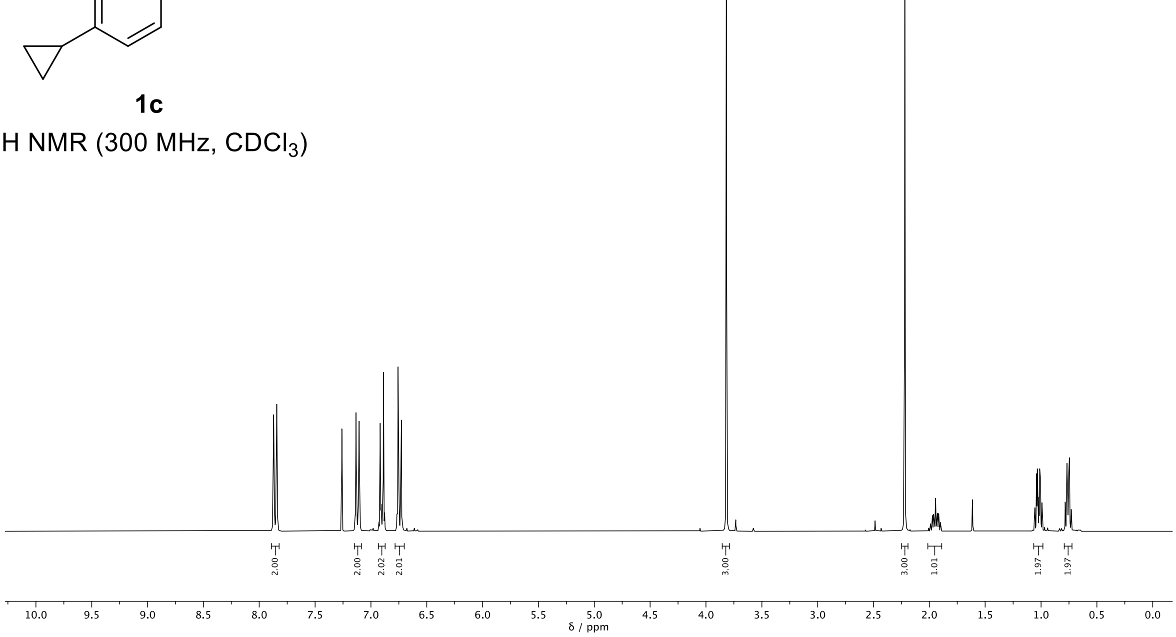

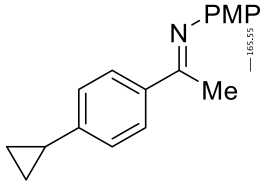

1c

^{13}C NMR (75 MHz, $CDCl_3$)

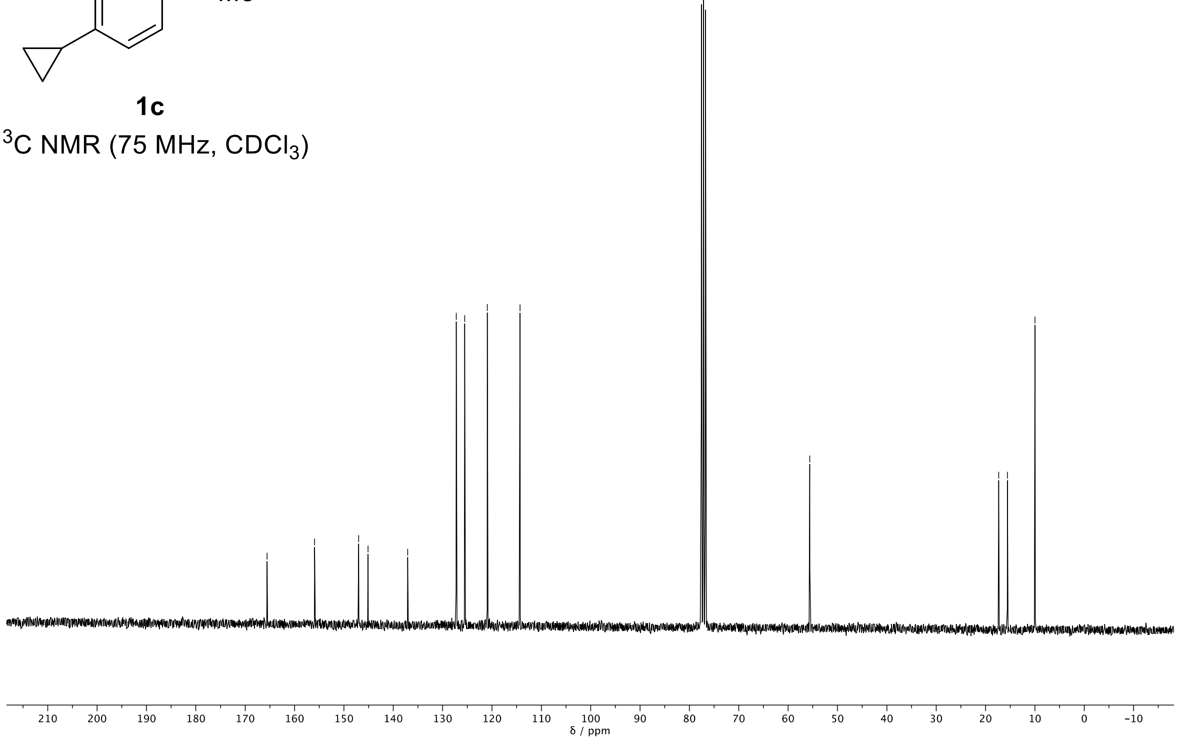

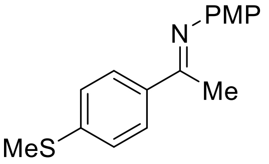

1h

^{1}H NMR (400 MHz, $CDCl_3$)

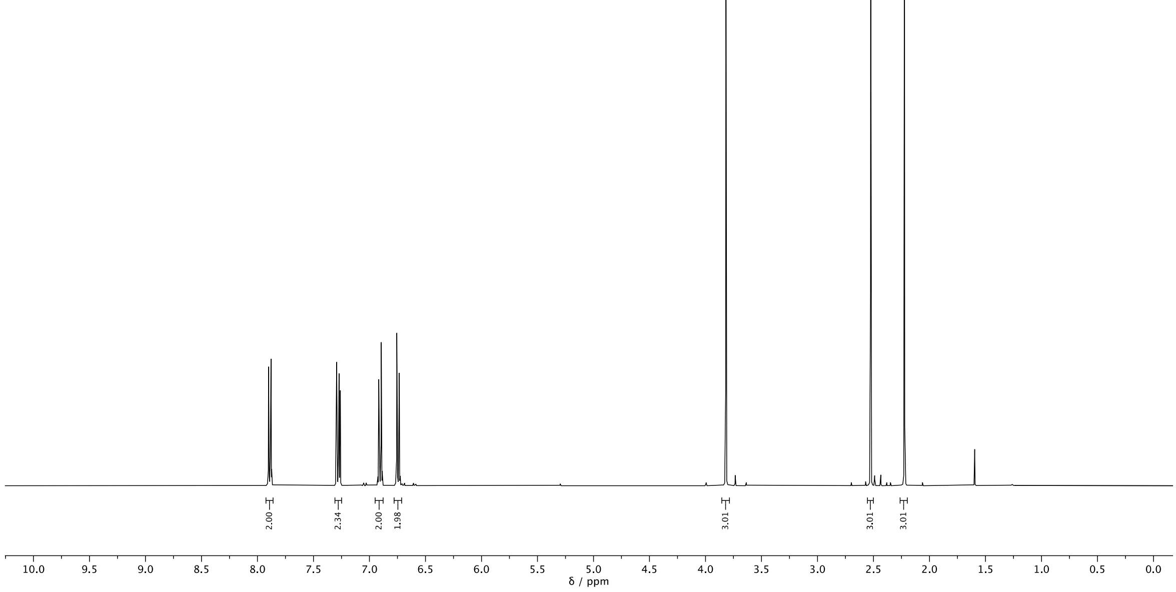

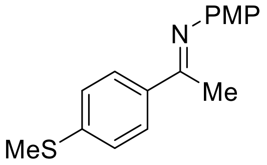

1h

^{13}C NMR (101 MHz, $CDCl_3$)

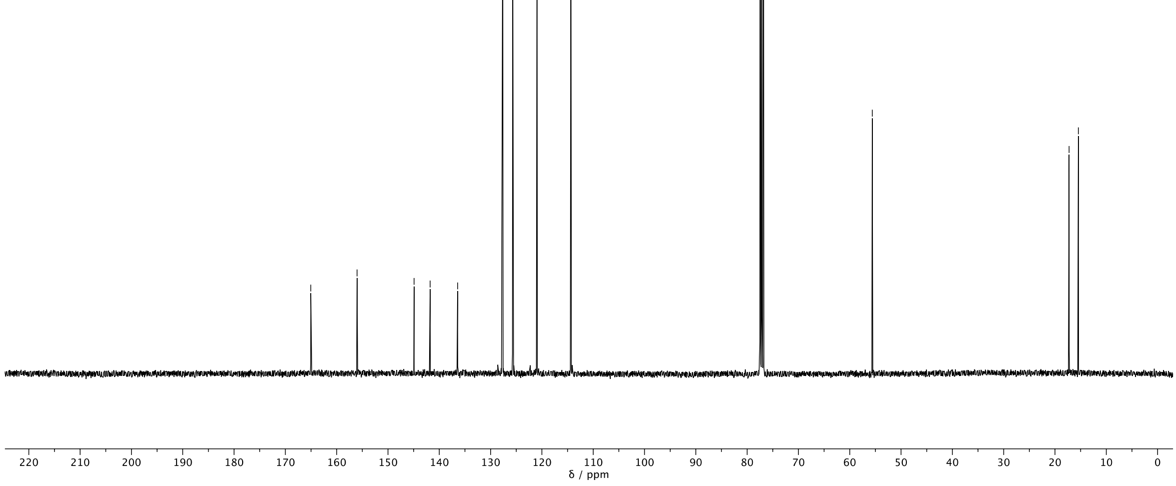

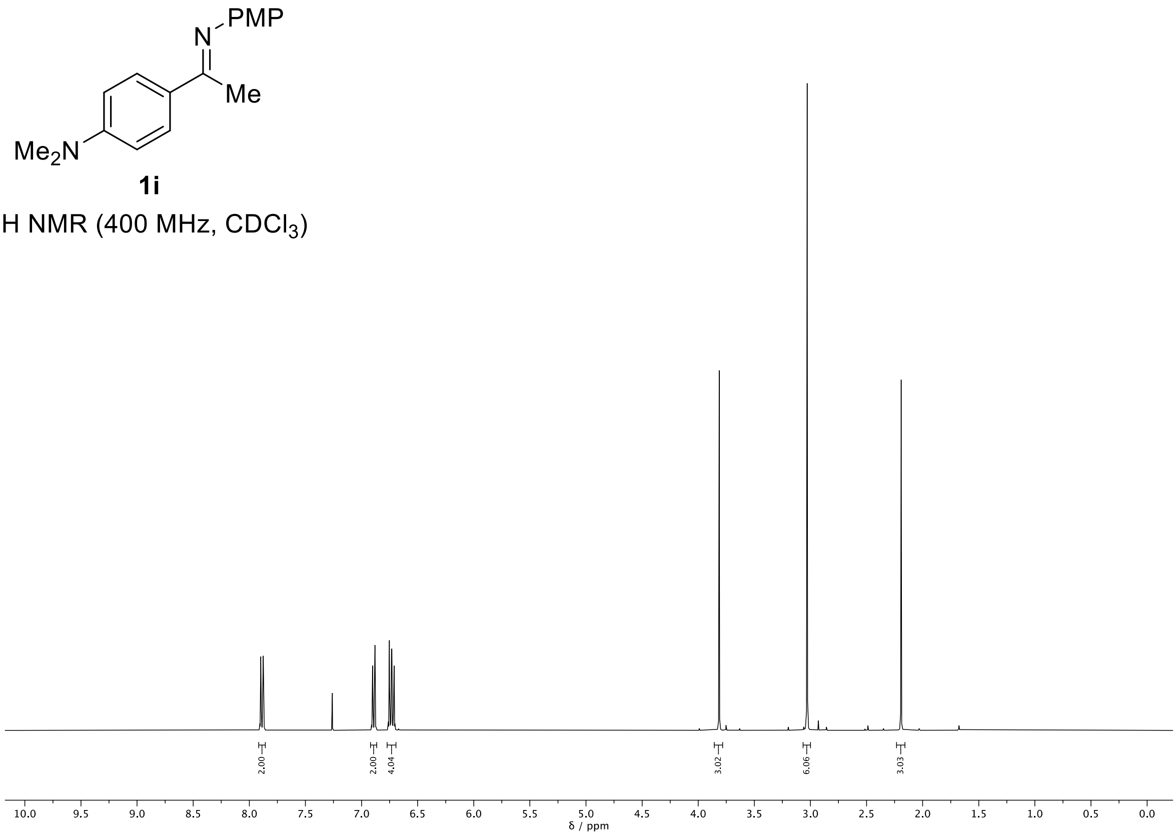

1i

^{1}H NMR (400 MHz, $CDCl_3$)

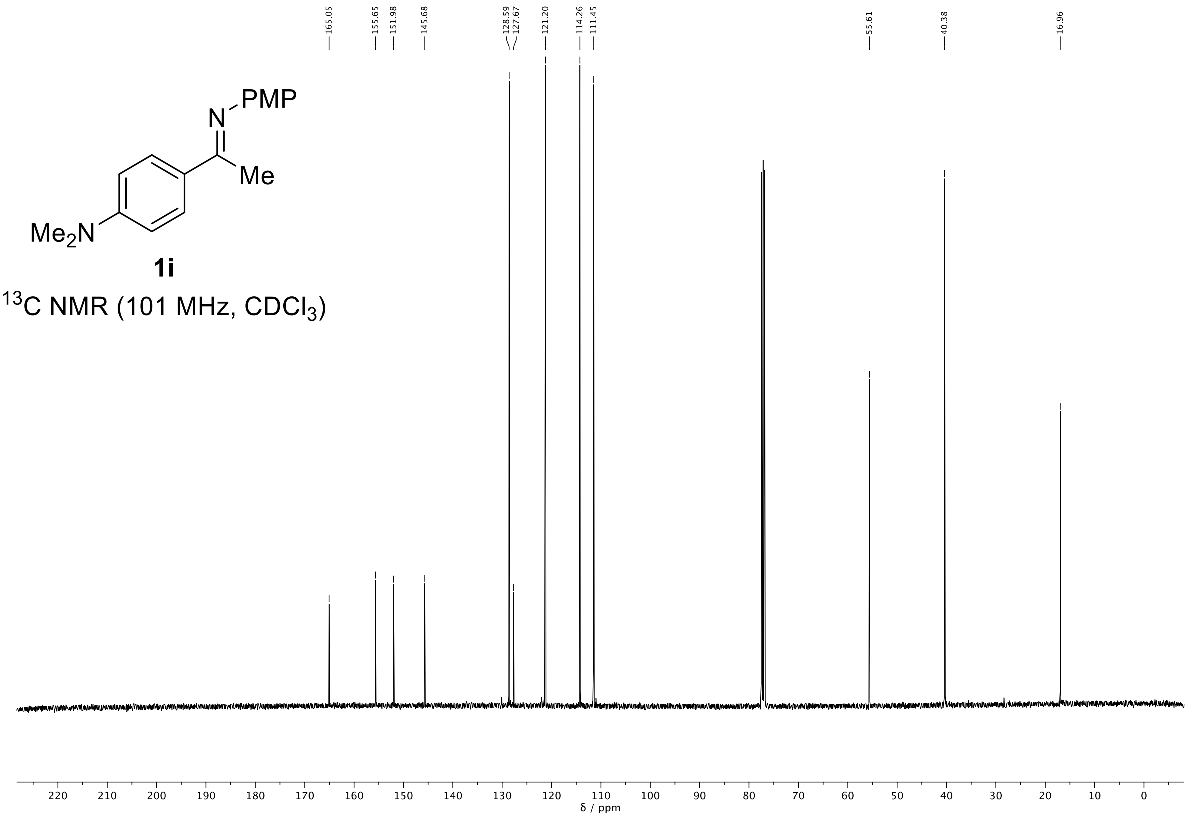

1i

^{13}C NMR (101 MHz, $CDCl_3$)

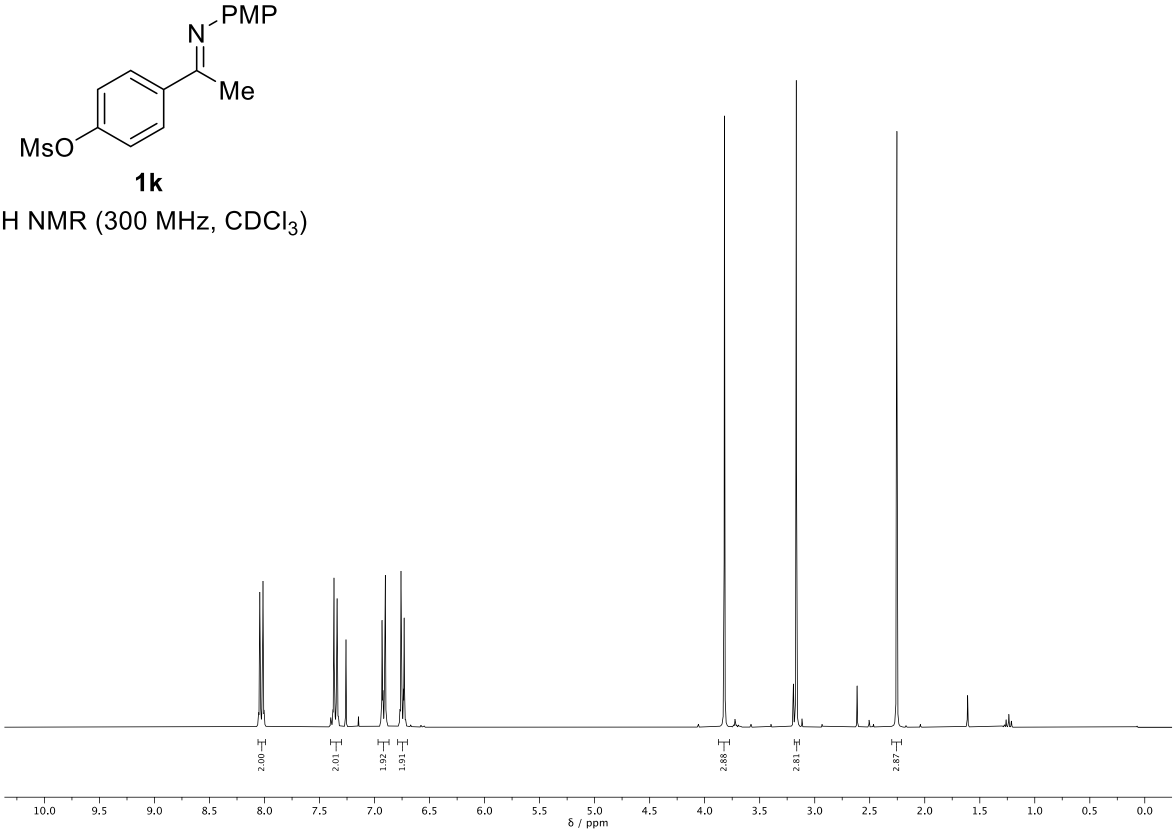

1k

^{1}H NMR (300 MHz, $CDCl_3$)

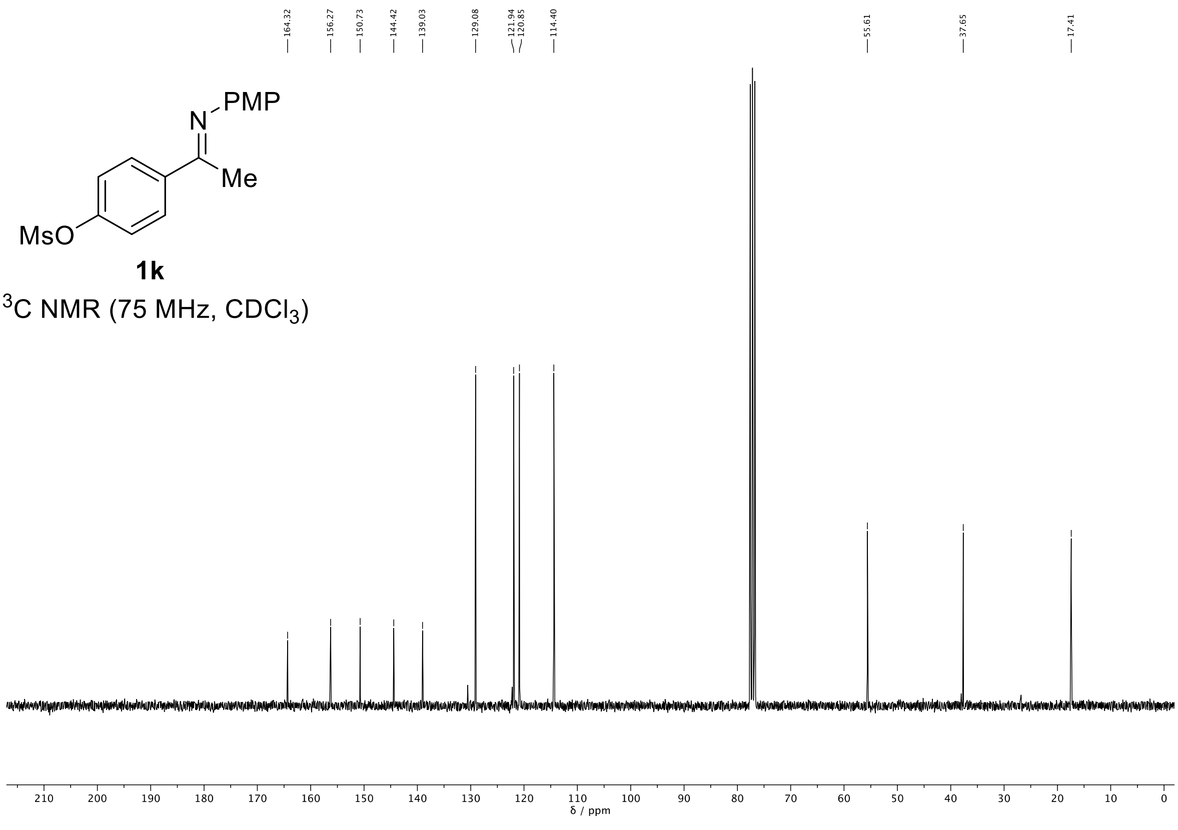

1k

^{13}C NMR (75 MHz, $CDCl_3$)

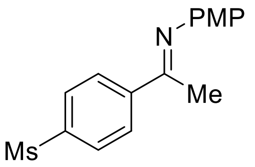

1p

^{1}H NMR (300 MHz, $CDCl_3$)

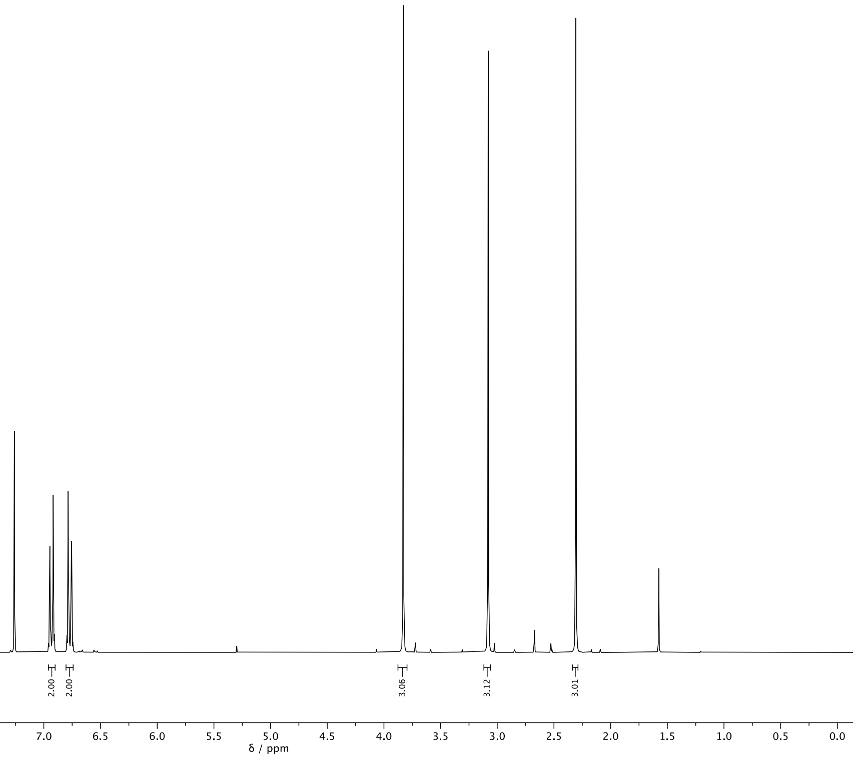

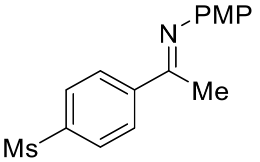

1p

^{13}C NMR (75 MHz, $CDCl_3$)

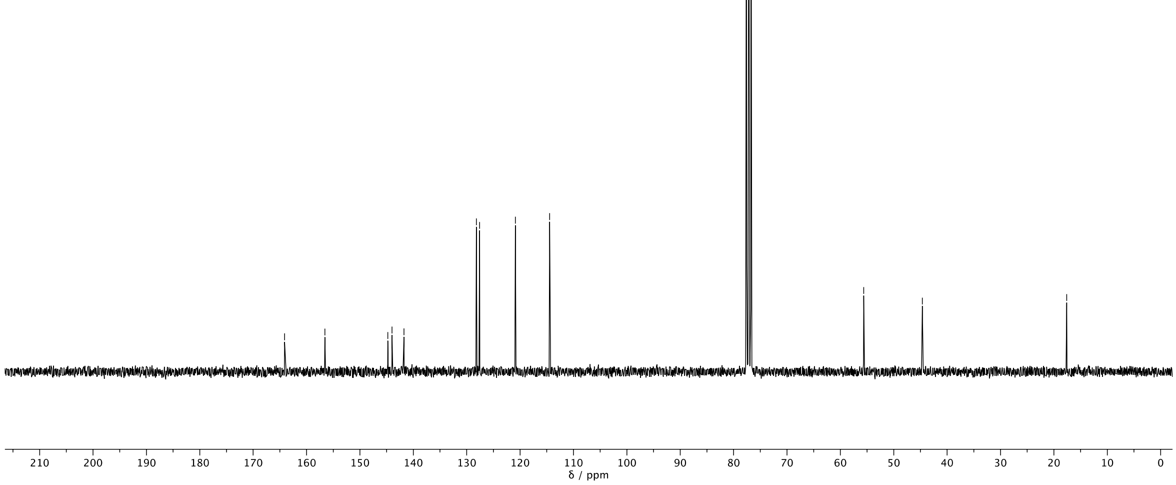

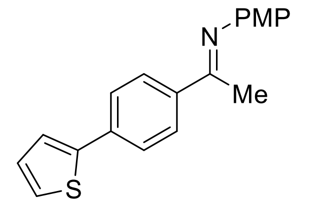

1q

^{1}H NMR (300 MHz, $CDCl_3$)

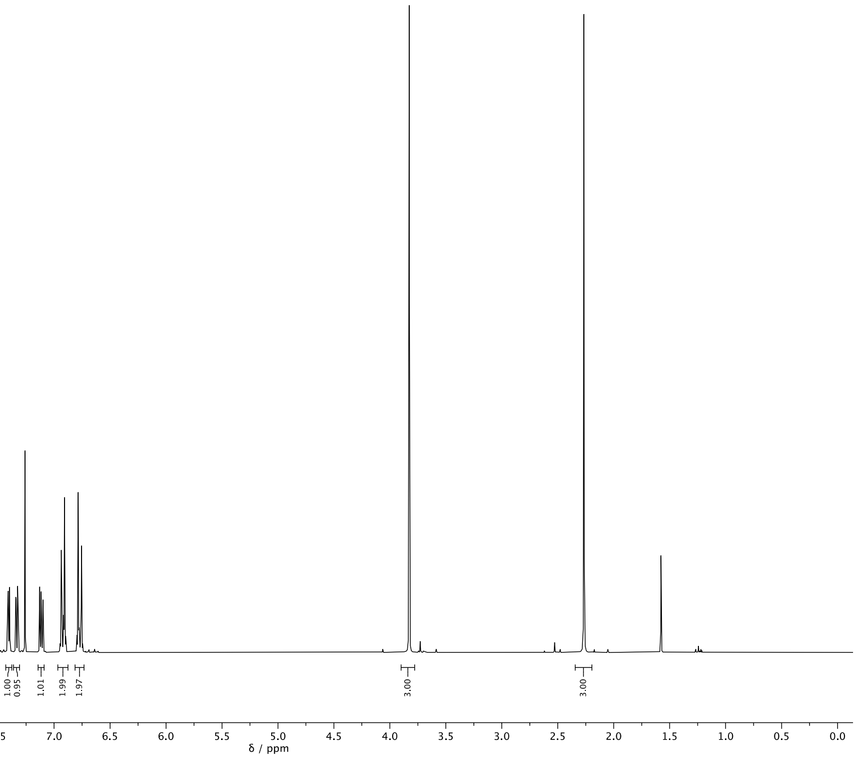

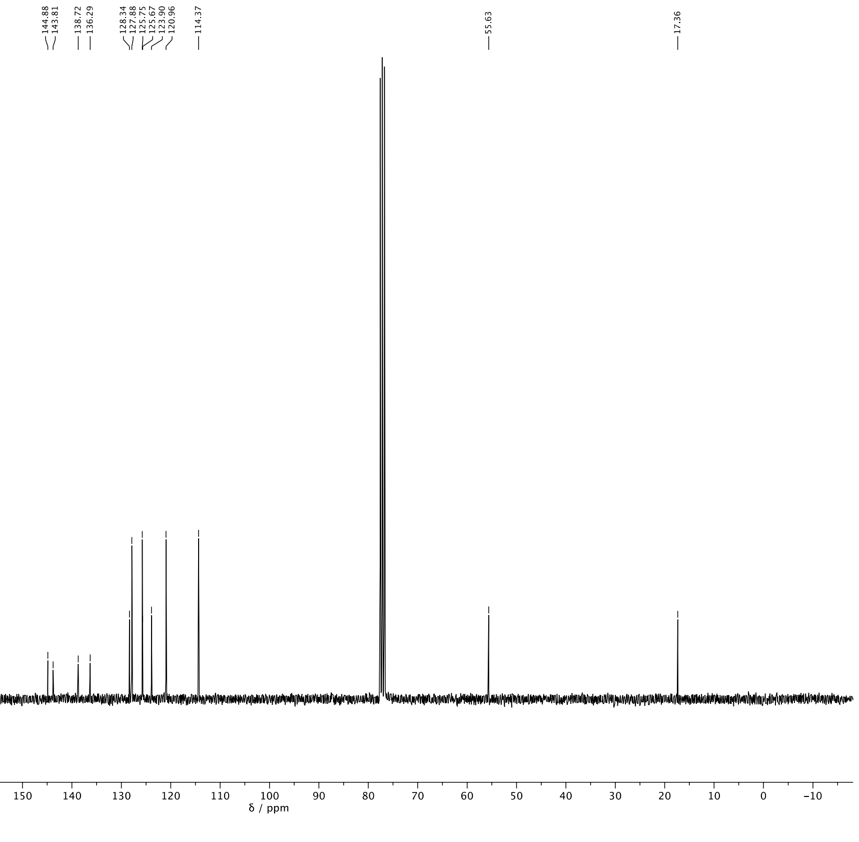

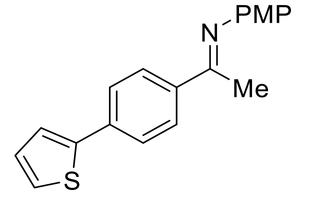

1q

^{13}C NMR (75 MHz, $CDCl_3$)

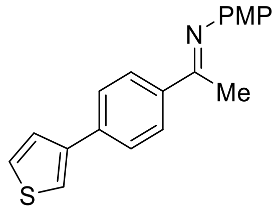

1r

^{1}H NMR (300 MHz, $CDCl_3$)

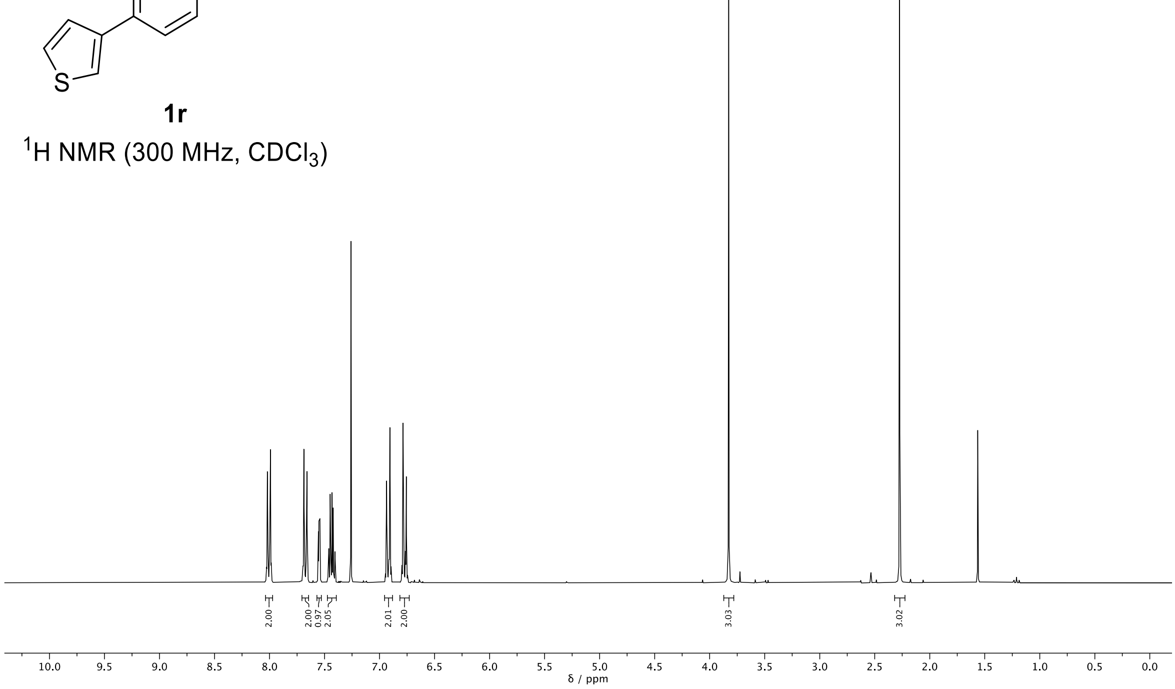

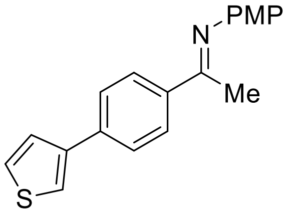

1r

^{13}C NMR (75 MHz, $CDCl_3$)

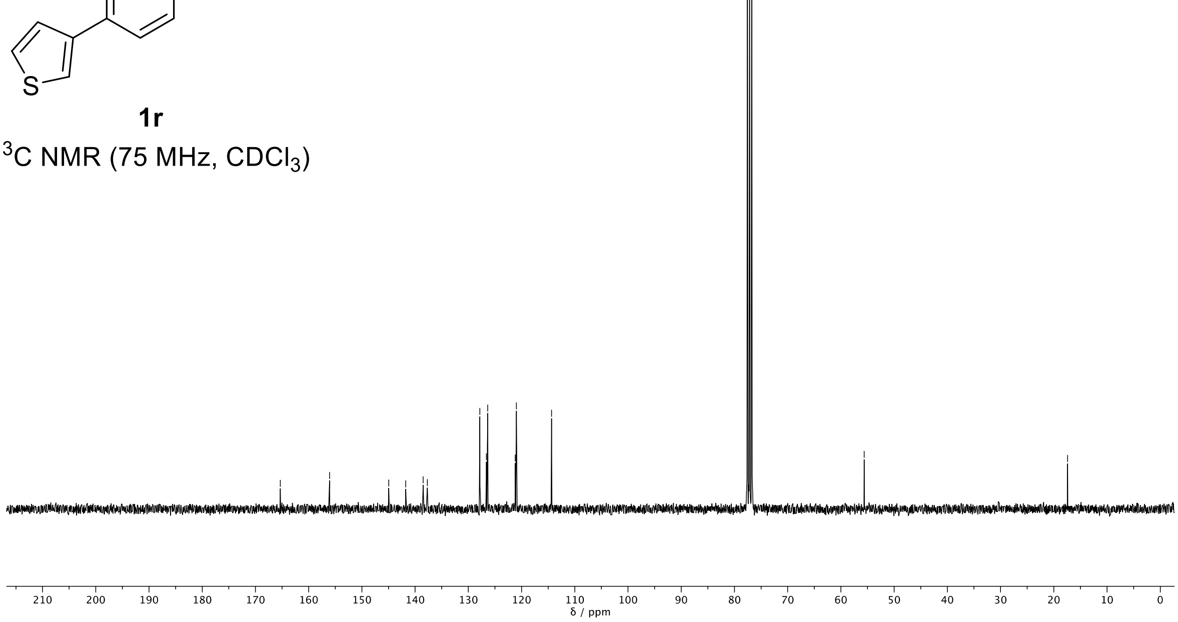

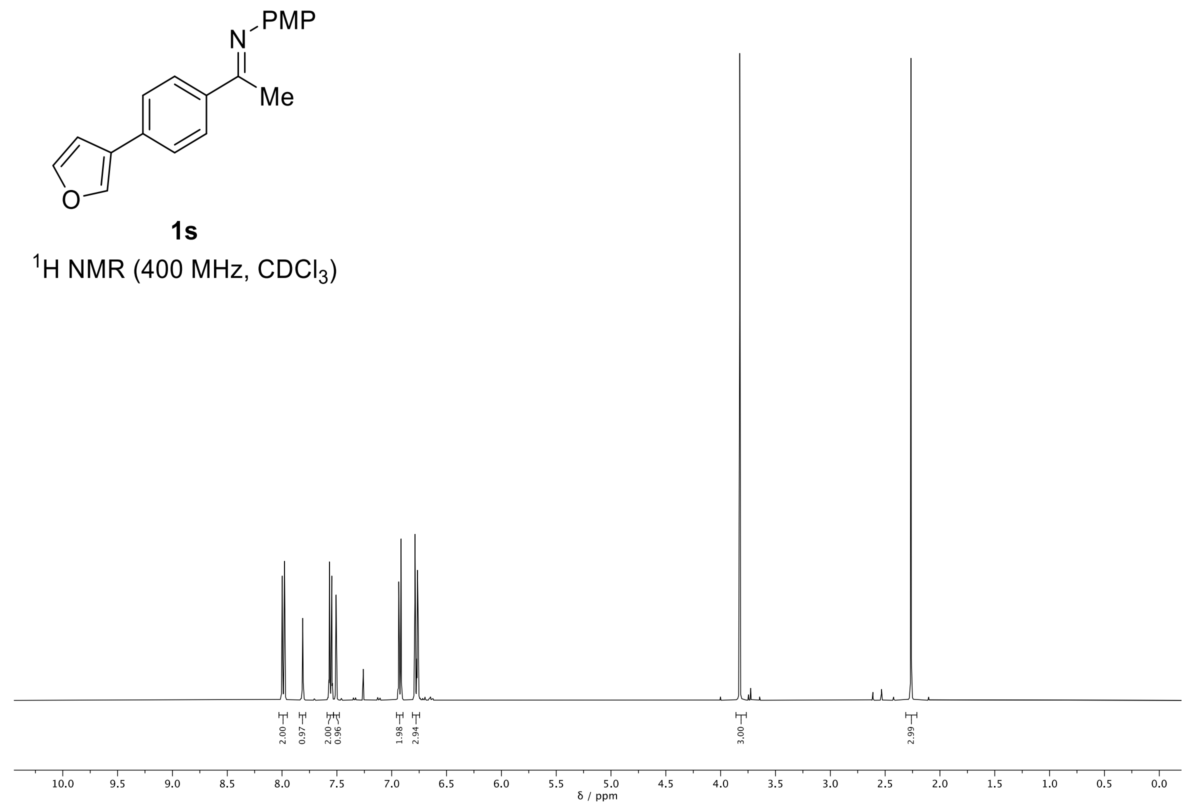
N-PMP
Me
O
1s
^{1}H NMR (400 MHz, CDCl$_3$)
2.00
0.97
2.00
0.96
1.98
2.94
3.00
2.99
10.0 9.5 9.0 8.5 8.0 7.5 7.0 6.5 6.0 5.5 5.0 4.5 4.0 3.5 3.0 2.5 2.0 1.5 1.0 0.5 0.0
δ / ppm

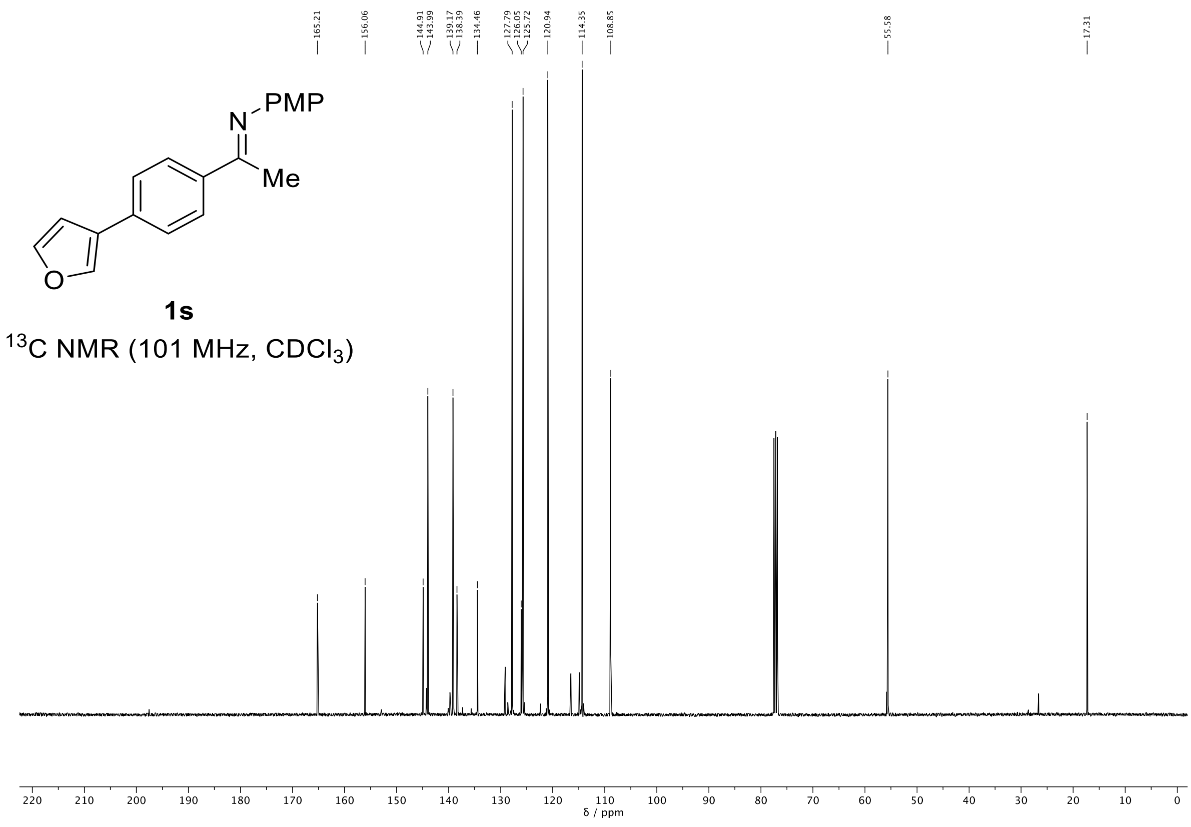
165.21
156.06
144.91
143.99
139.17
138.39
134.46
127.79
126.05
125.72
120.94
114.35
108.85
55.58
17.31
N-PMP
Me
O
1s
^{13}C NMR (101 MHz, CDCl$_3$)
220 210 200 190 180 170 160 150 140 130 120 110 100 90 80 70 60 50 40 30 20 10 0
δ / ppm

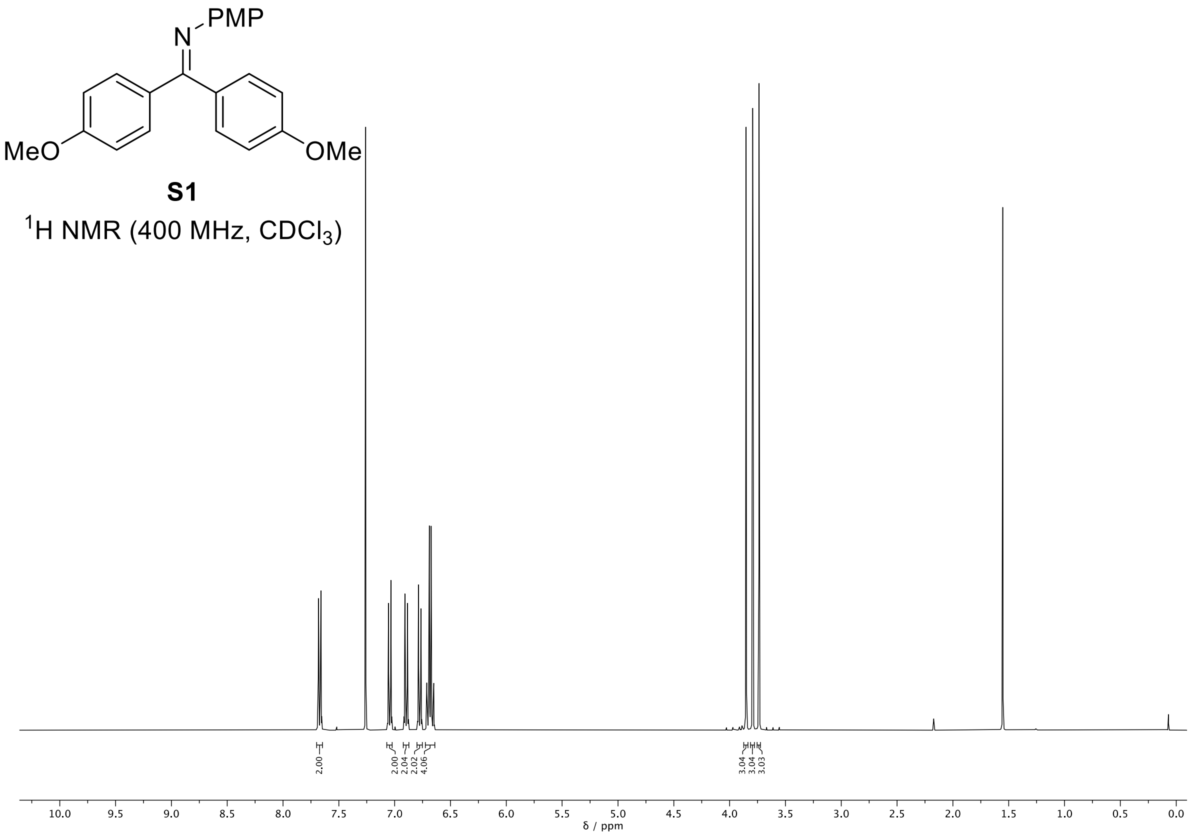
N
PMP
MeO
OMe
S1
¹H NMR (400 MHz, CDCl₃)
δ / ppm

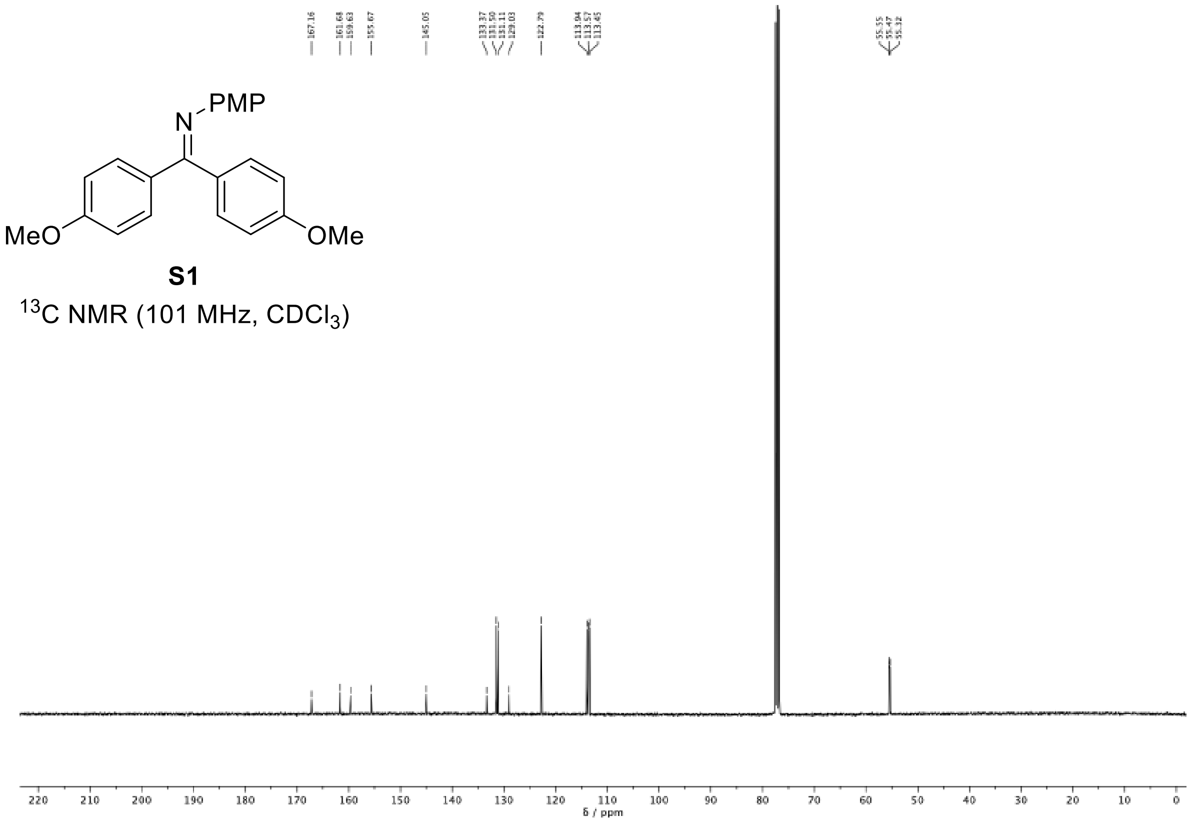
N
PMP
MeO
OMe
S1
¹³C NMR (101 MHz, CDCl₃)
δ / ppm

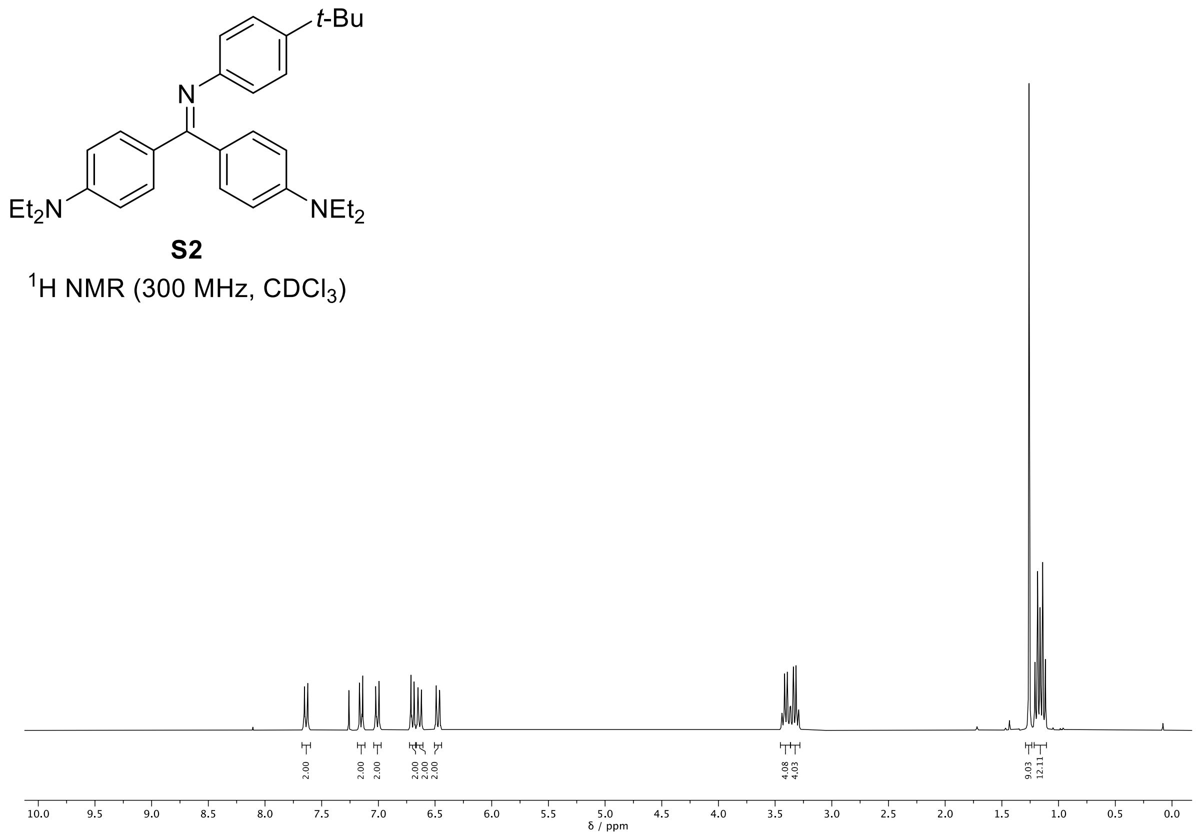

S2

^{1}H NMR (300 MHz, $CDCl_3$)

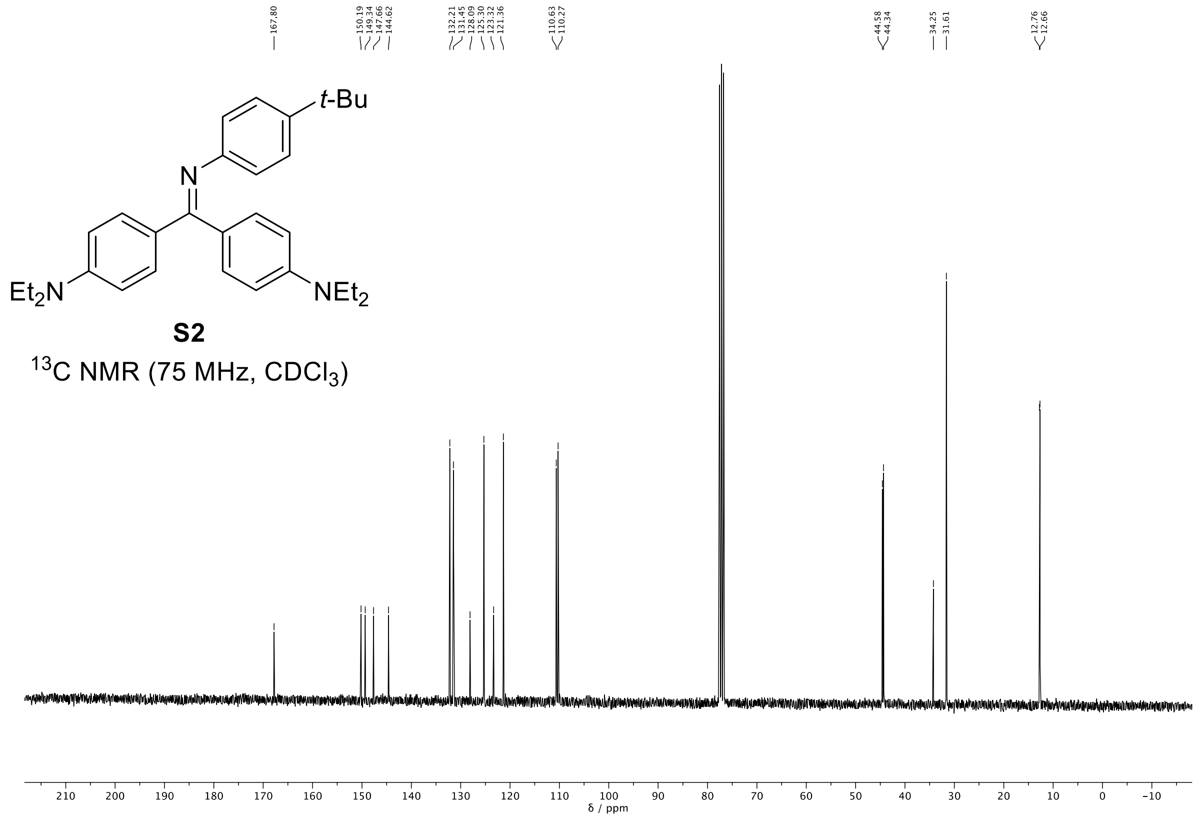

S2

^{13}C NMR (75 MHz, $CDCl_3$)

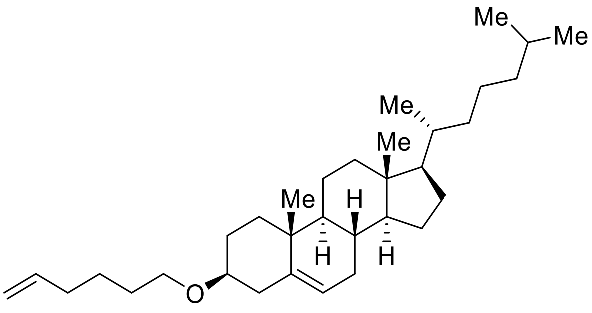

S5

^{1}H NMR (400 MHz, $CDCl_3$)

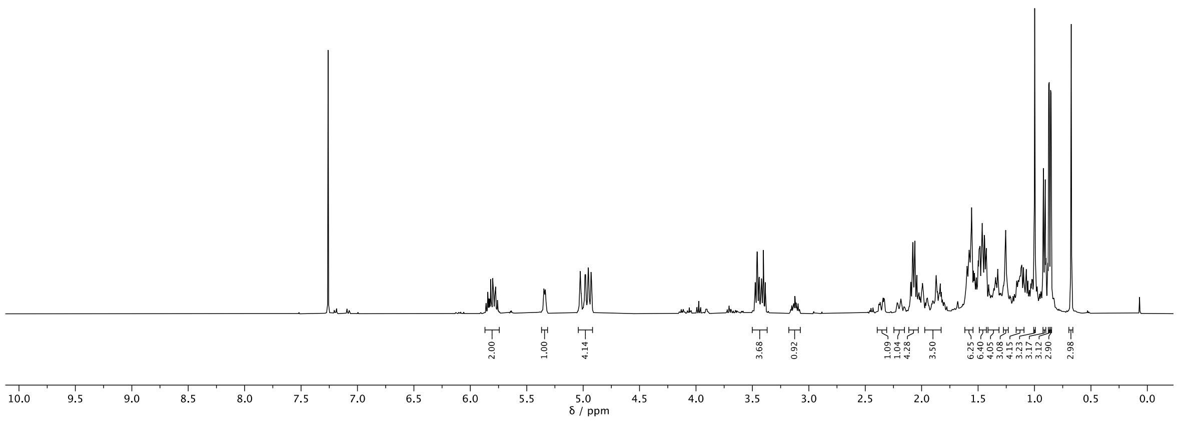

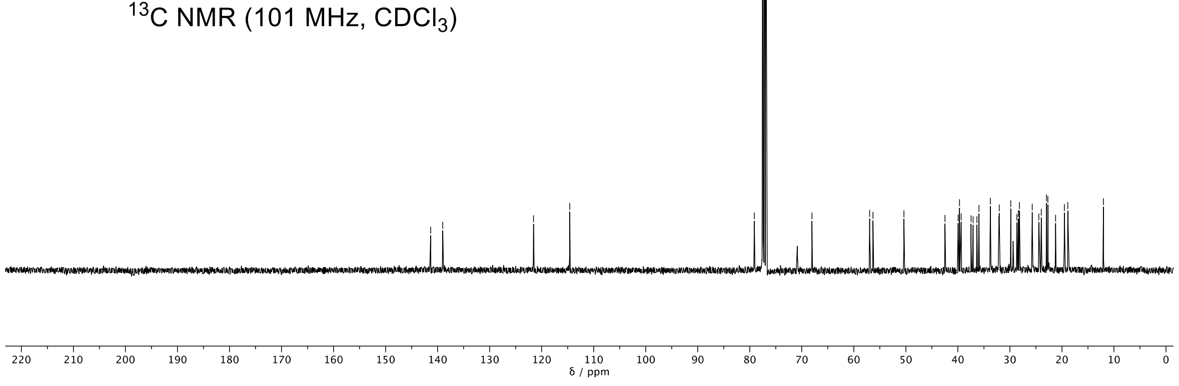

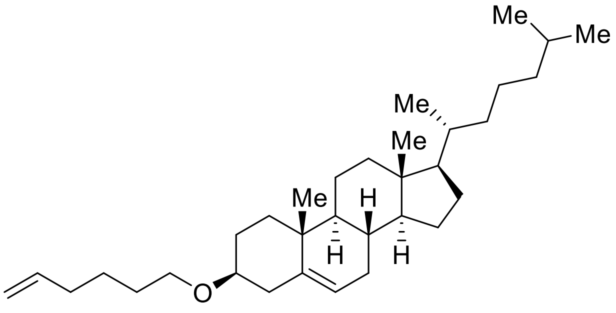

S5

^{13}C NMR (101 MHz, $CDCl_3$)

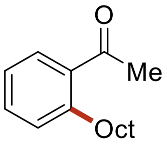

2a

^{1}H NMR (400 MHz, $CDCl_3$)

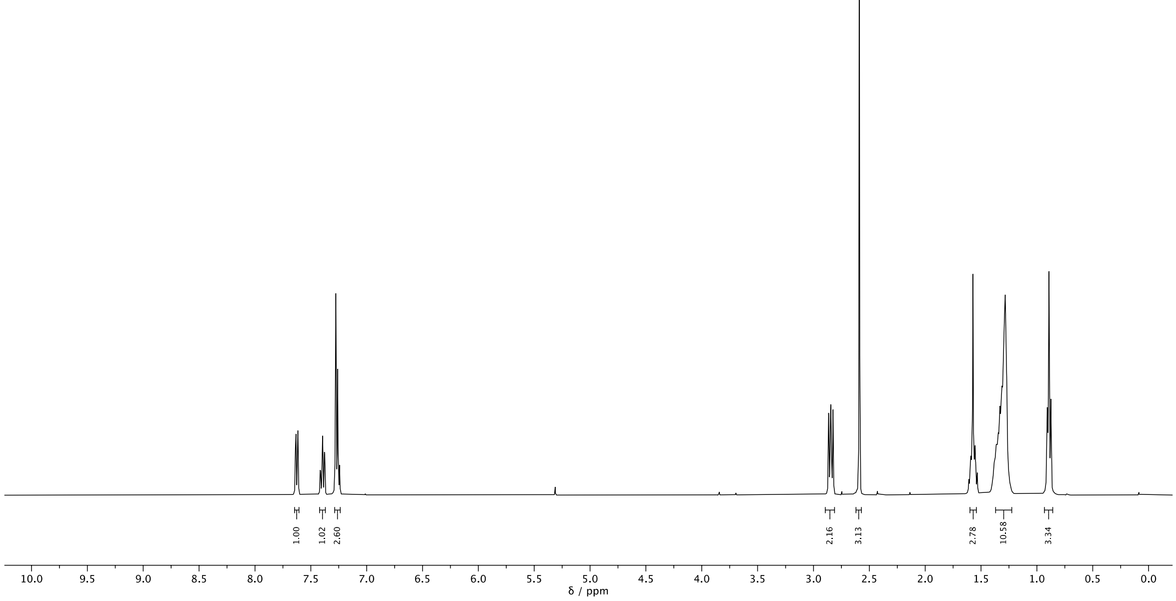

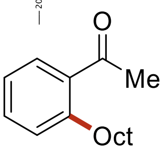

2a

^{13}C NMR (101 MHz, $CDCl_3$)

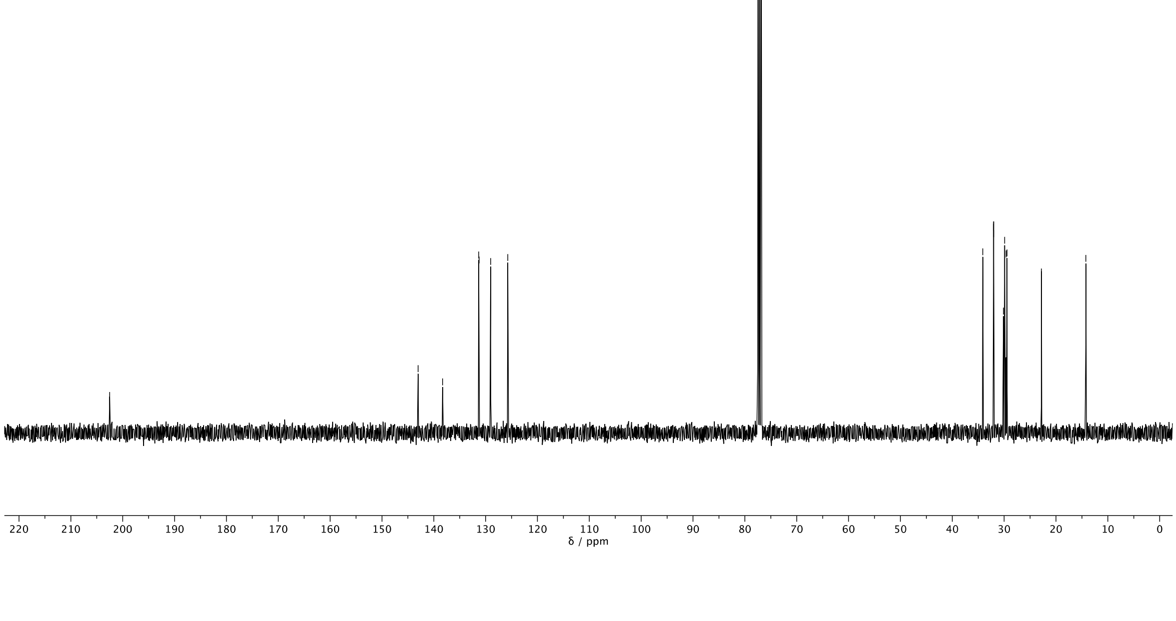

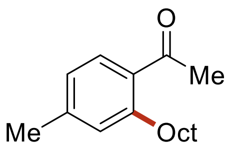

2b

^{1}H NMR (300 MHz, $CDCl_3$)

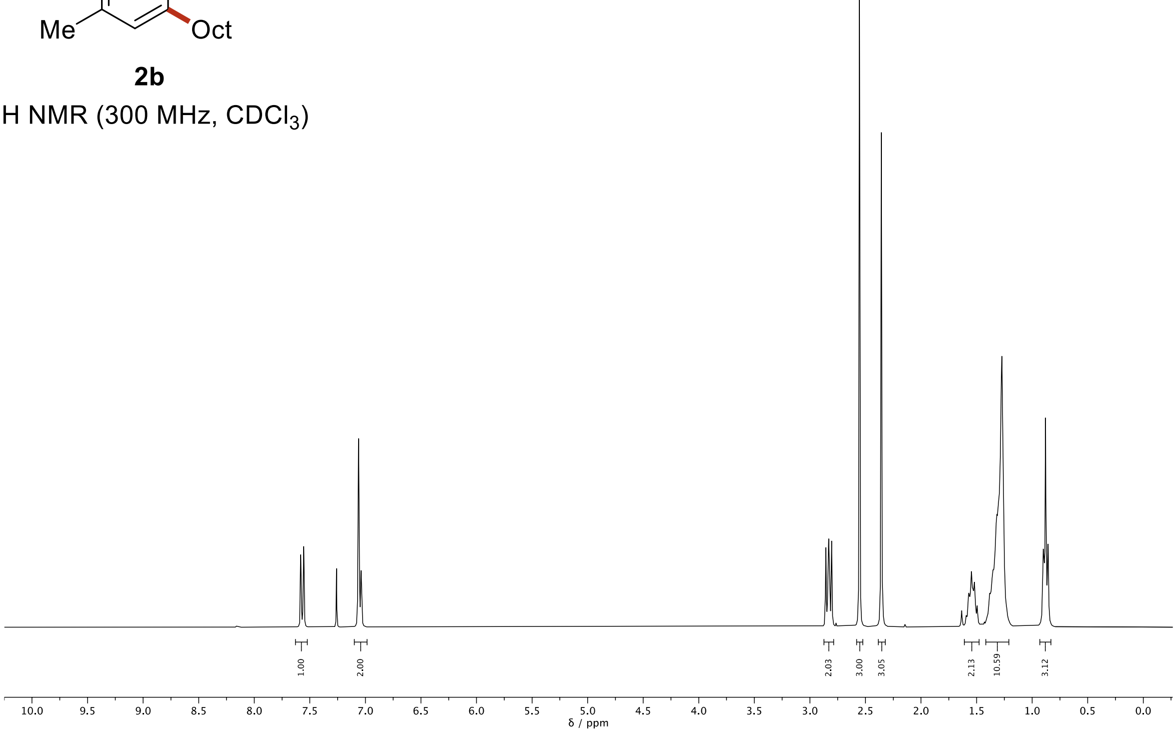

2b

^{13}C NMR (75 MHz, $CDCl_3$)

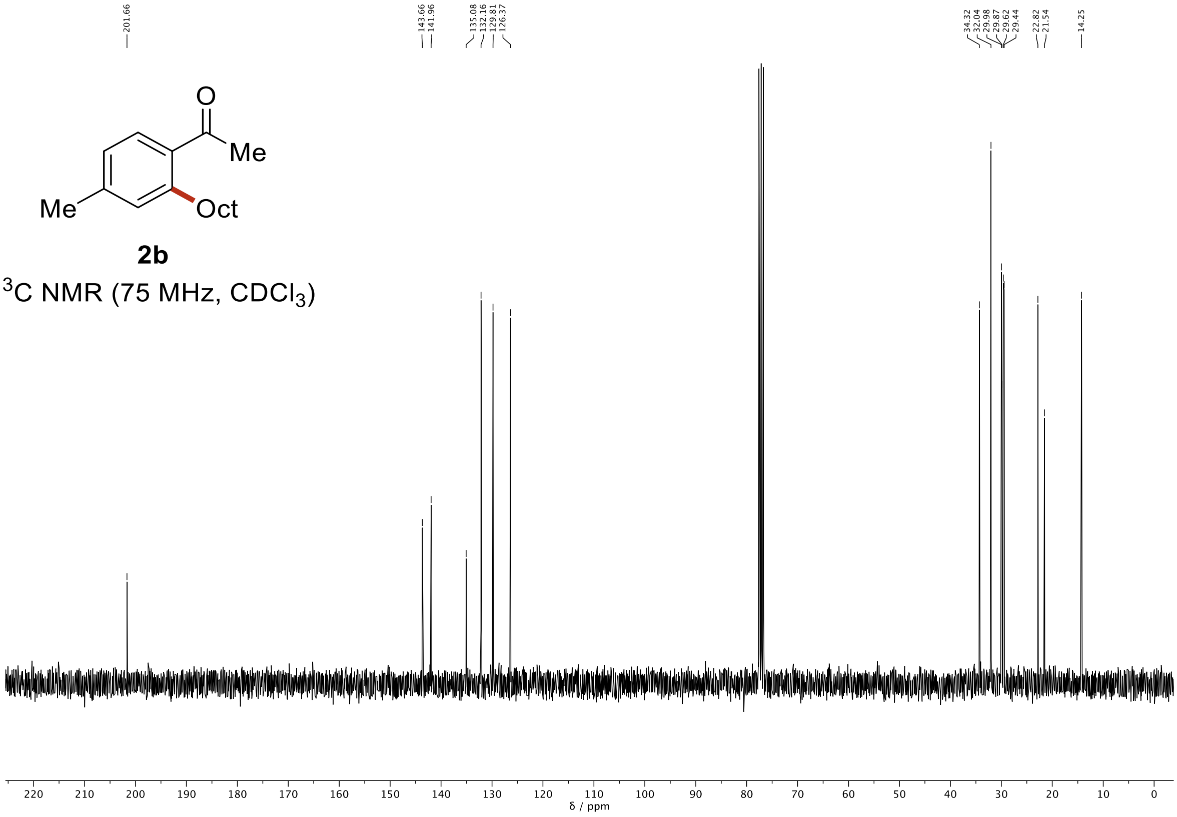

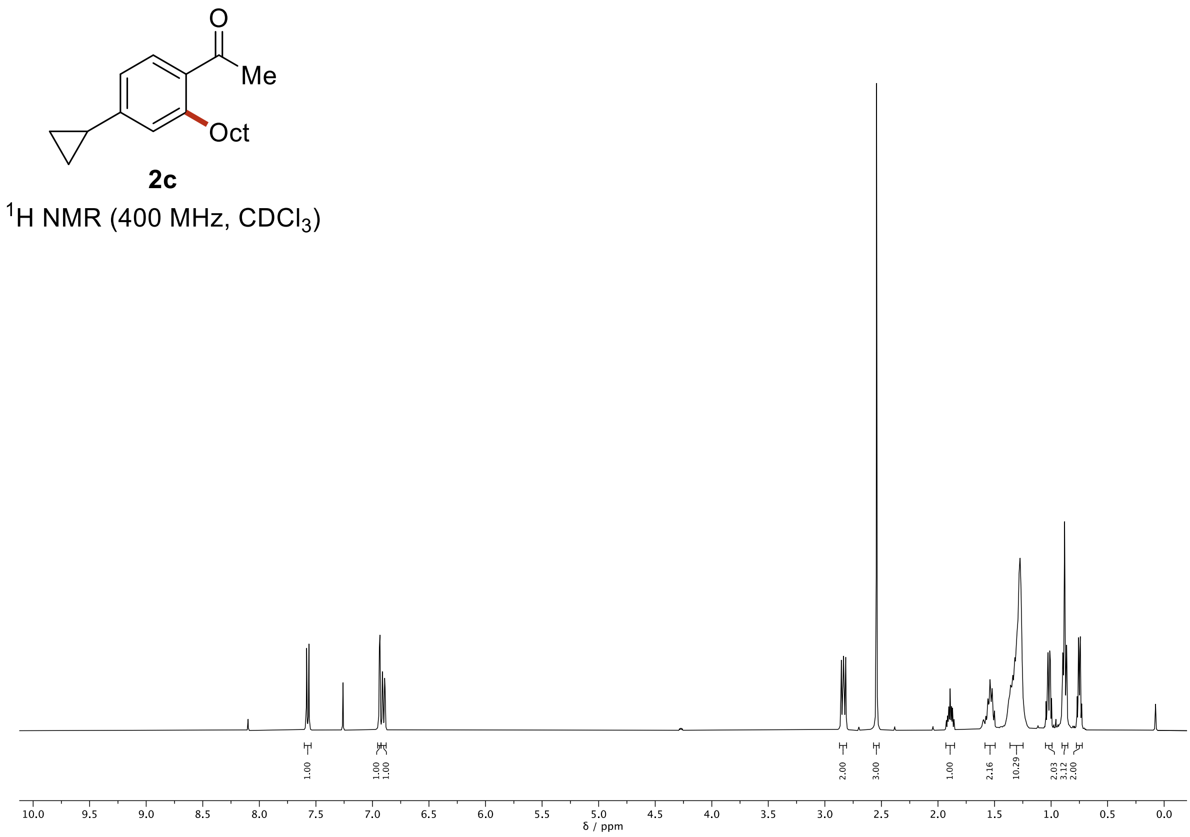

2c

^{1}H NMR (400 MHz, $CDCl_3$)

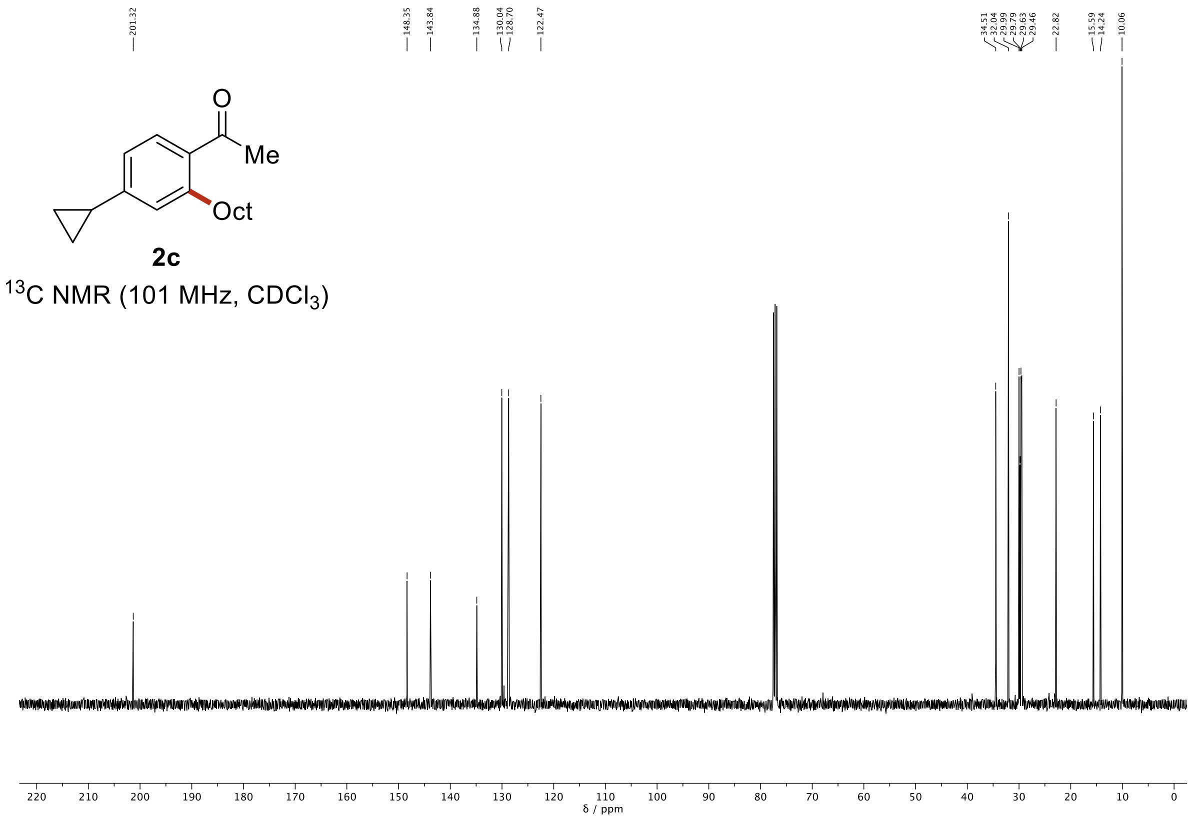

2c

^{13}C NMR (101 MHz, $CDCl_3$)

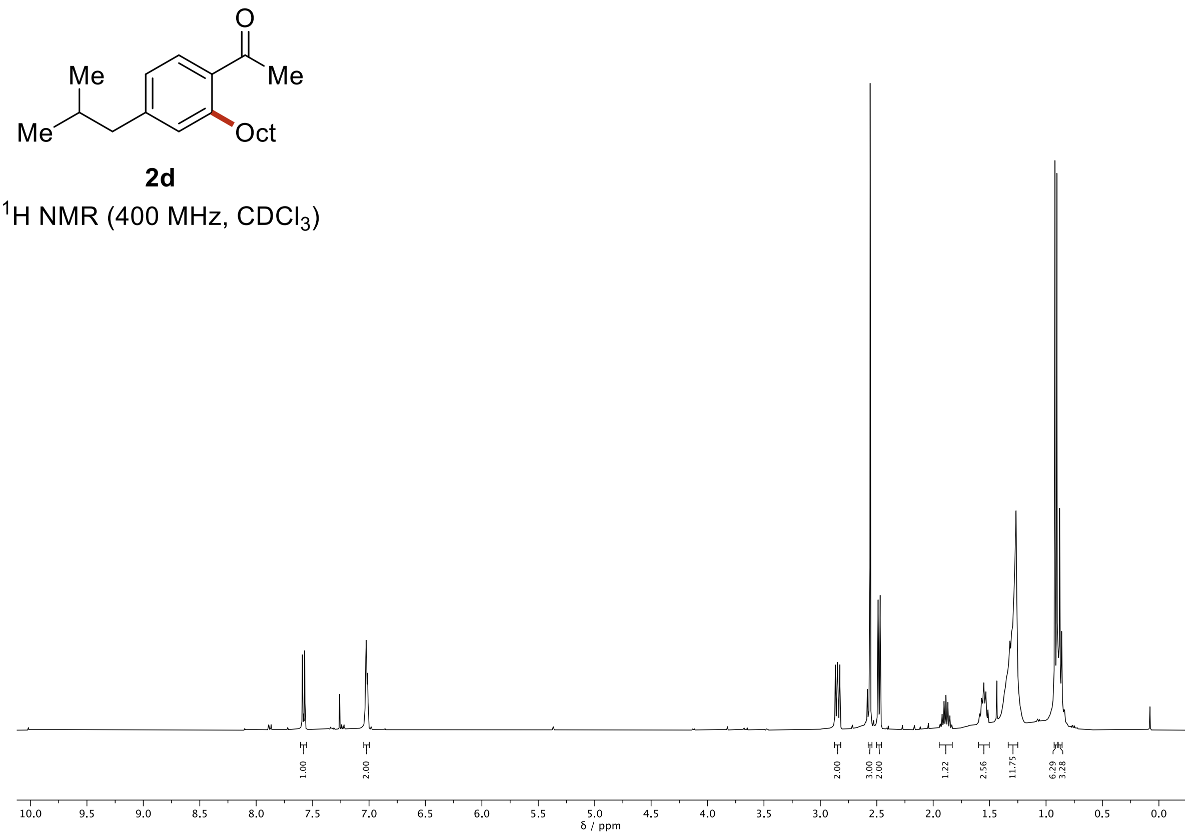

2d

^{1}H NMR (400 MHz, $CDCl_3$)

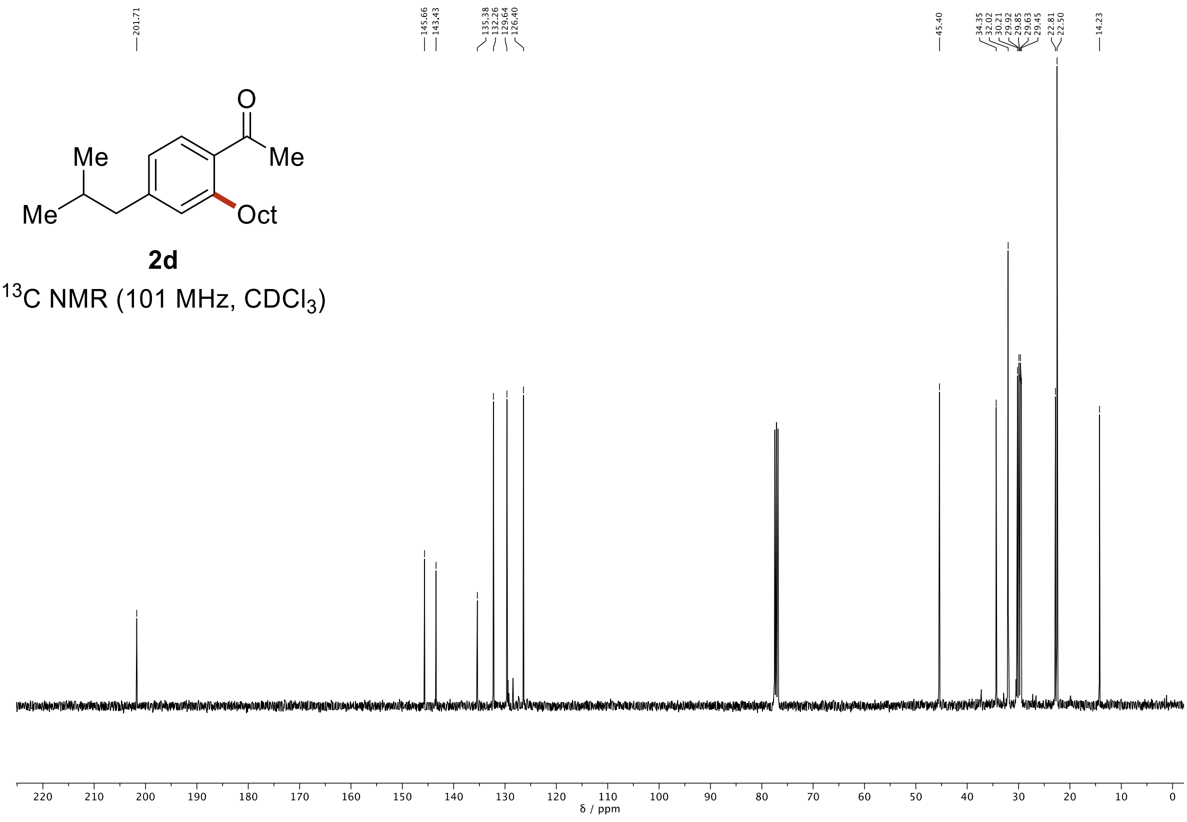

2d

^{13}C NMR (101 MHz, $CDCl_3$)

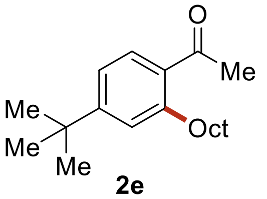

2e

^{1}H NMR (300 MHz, $CDCl_3$)

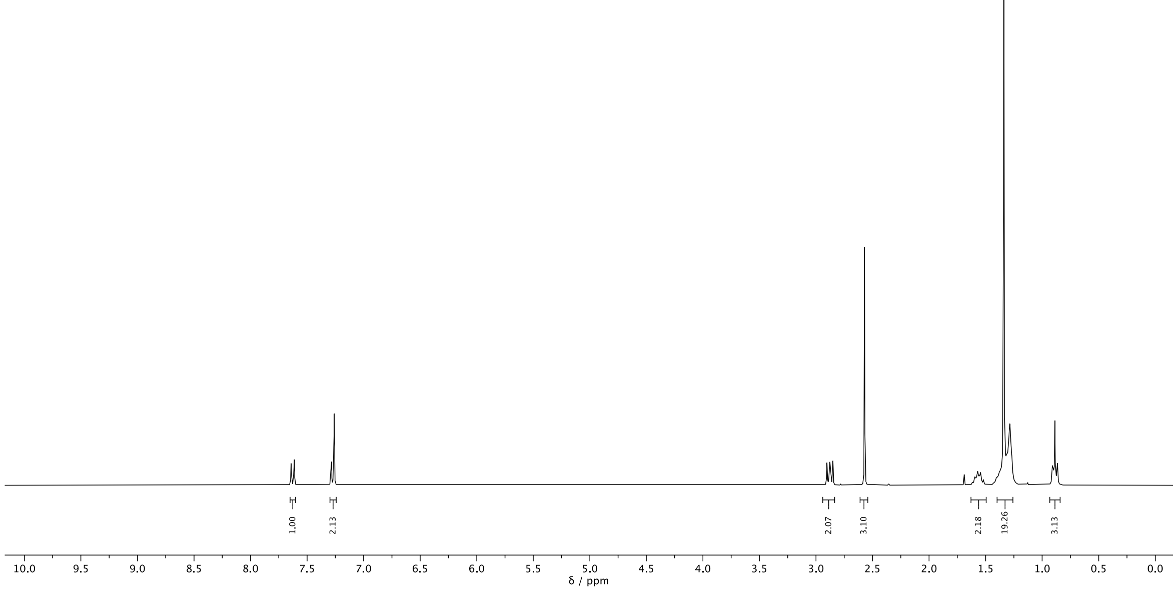

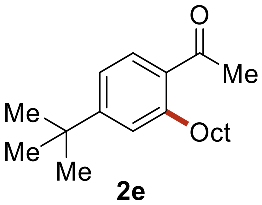

2e

^{13}C NMR (75 MHz, $CDCl_3$)

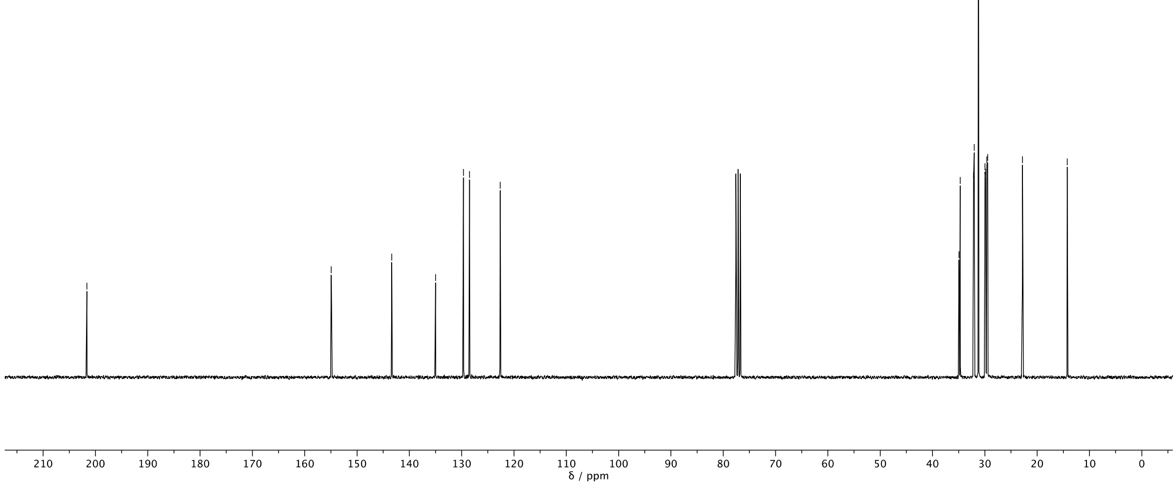

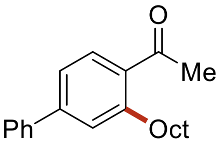

2f

^{1}H NMR (400 MHz, $CDCl_3$)

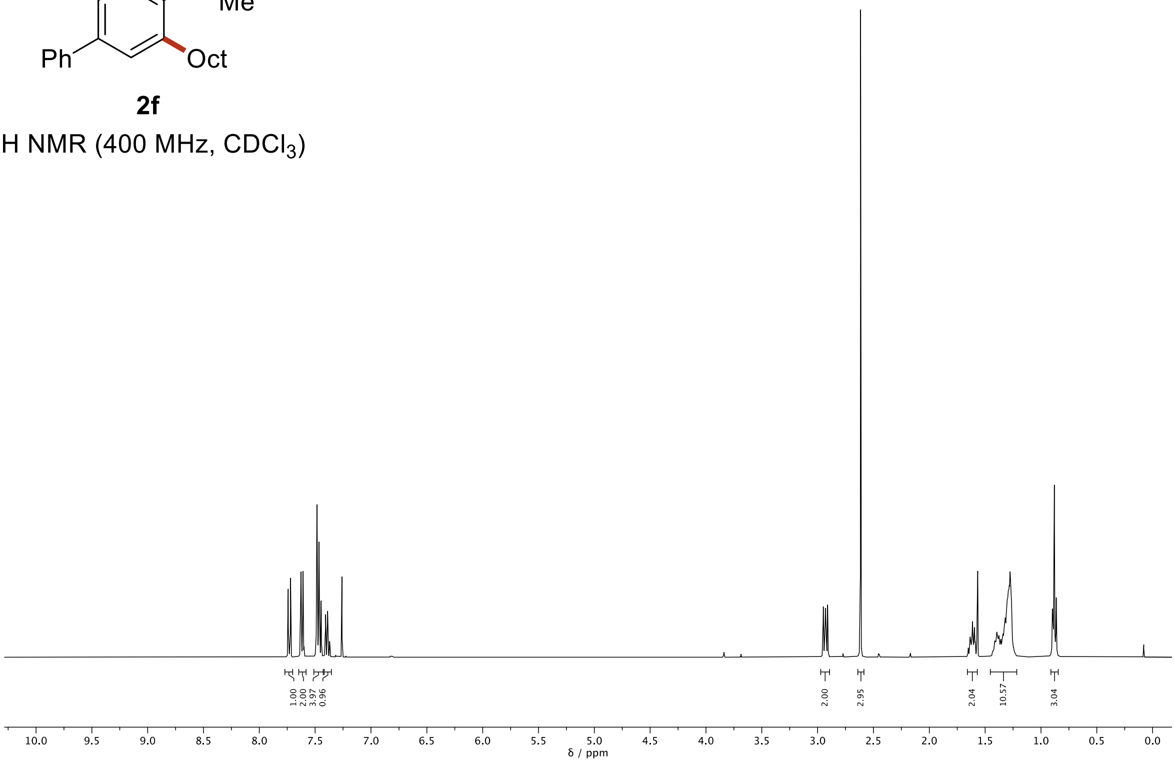

2f

^{13}C NMR (101 MHz, $CDCl_3$)

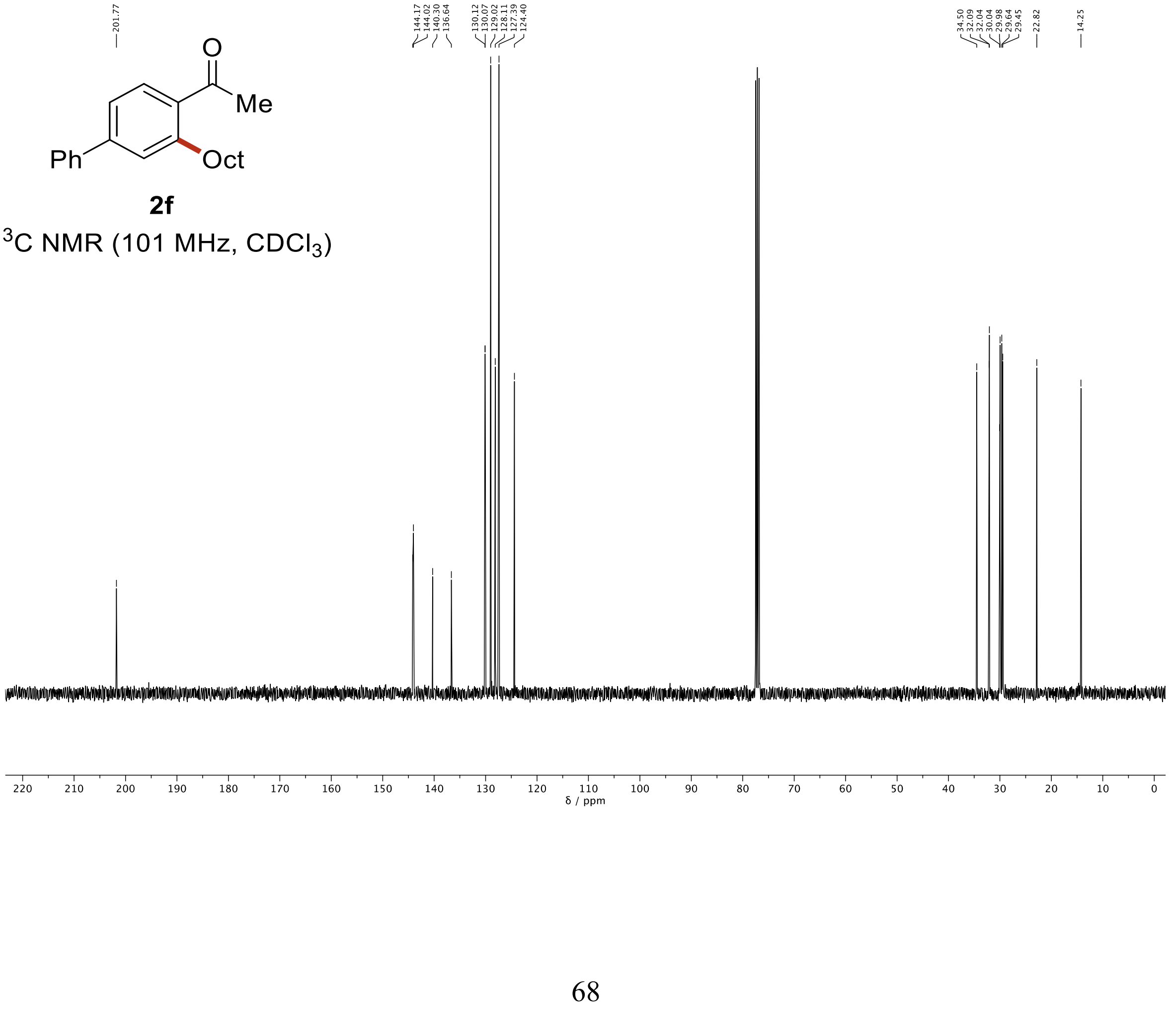

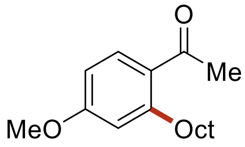

2g

^{1}H NMR (300 MHz, $CDCl_3$)

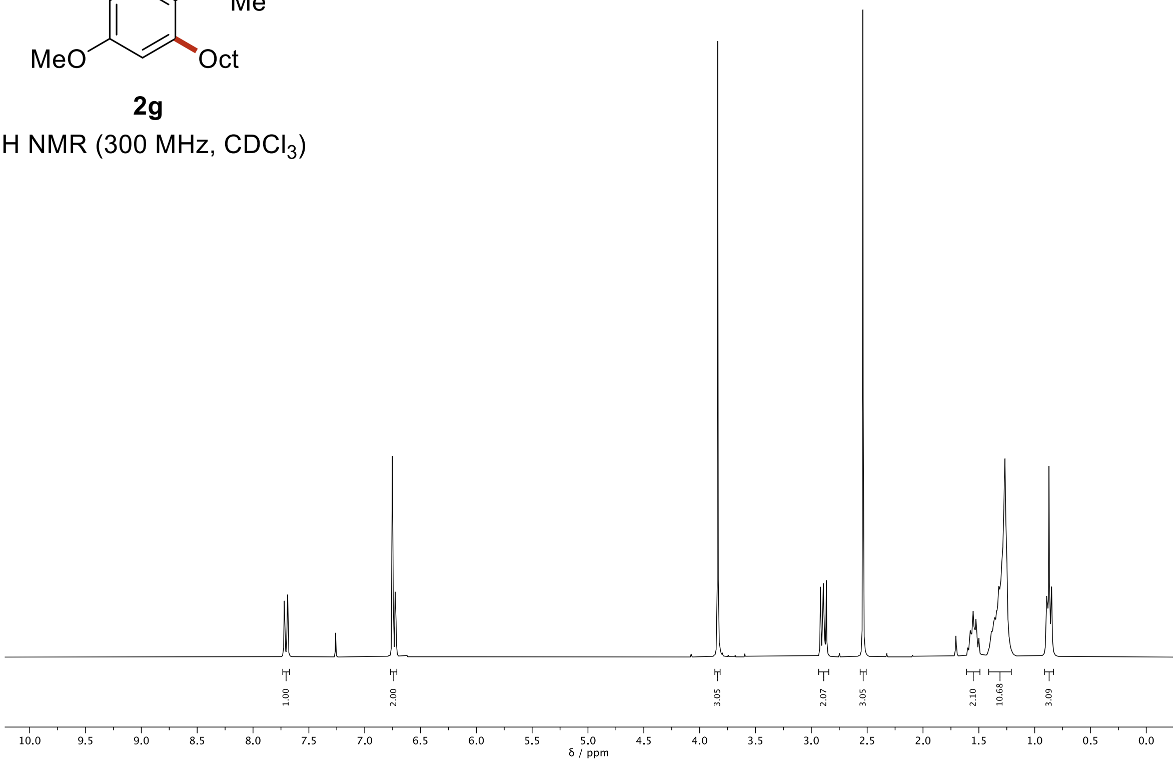

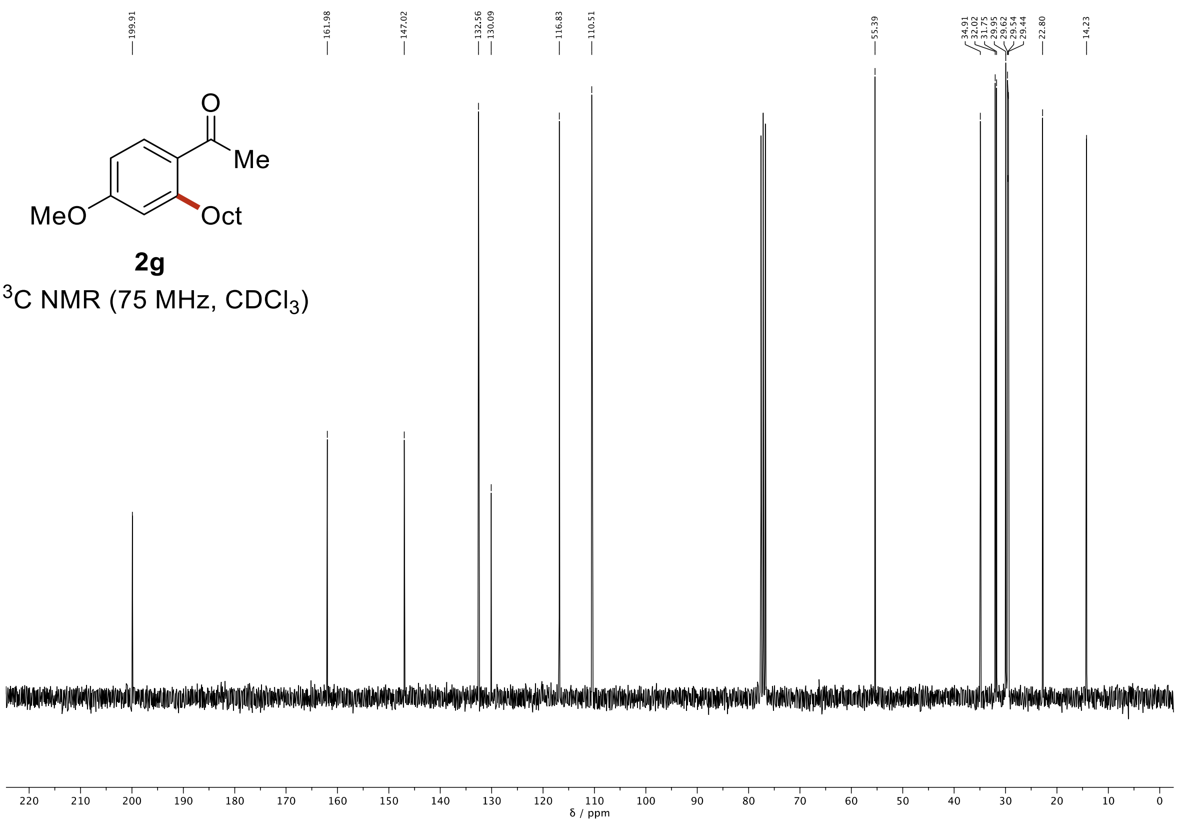

2g

^{13}C NMR (75 MHz, $CDCl_3$)

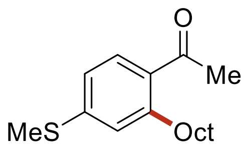

2h

^{1}H NMR (300 MHz, $CDCl_3$)

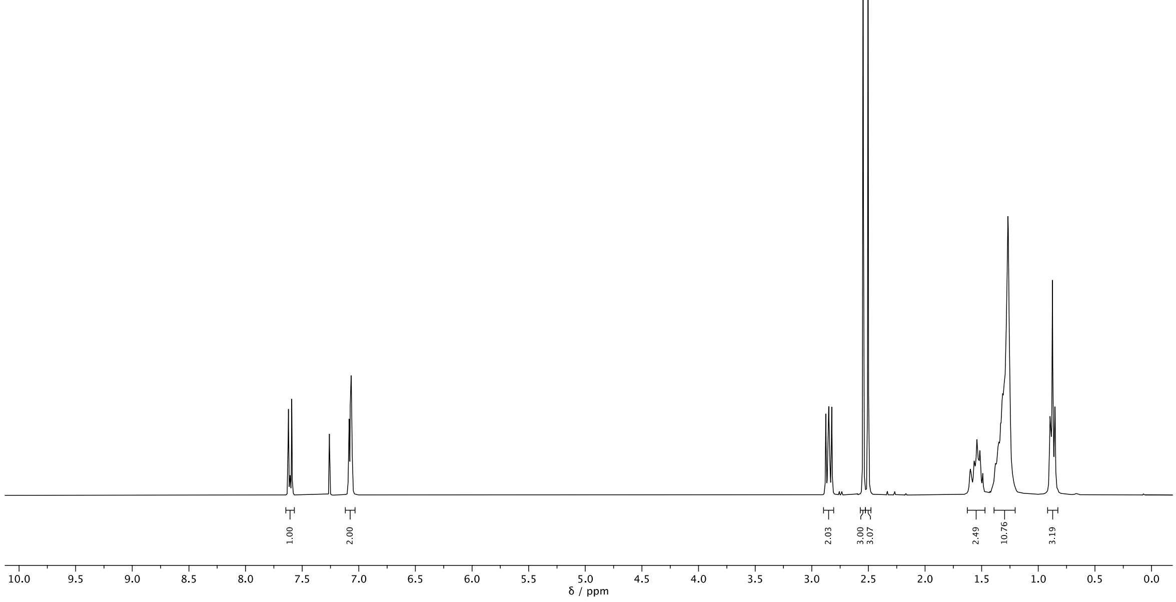

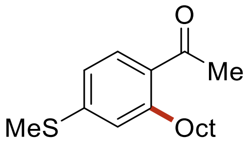

2h

^{13}C NMR (75 MHz, $CDCl_3$)

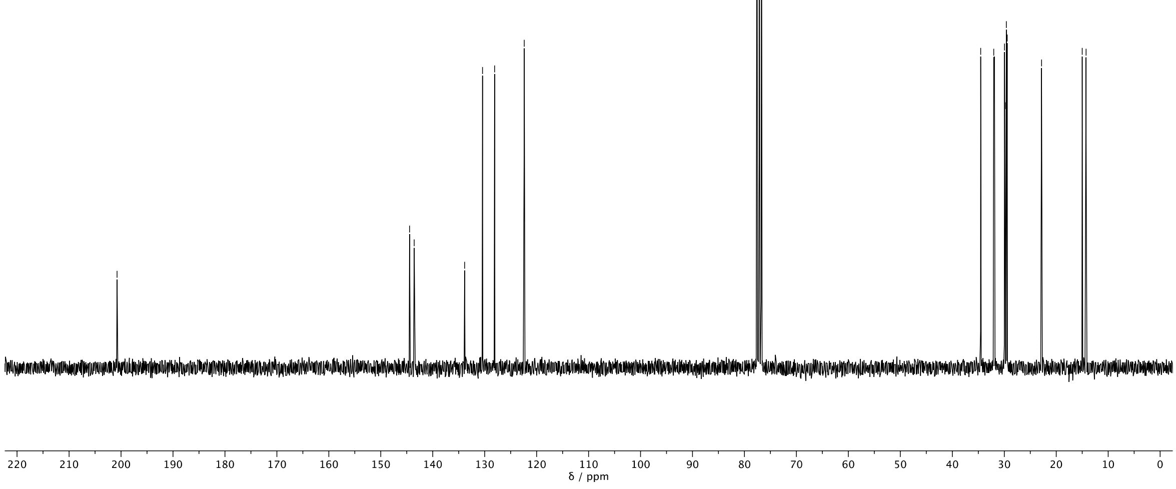

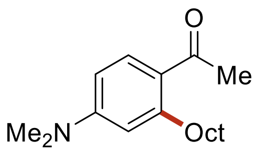

2i

^{1}H NMR (300 MHz, $CDCl_3$)

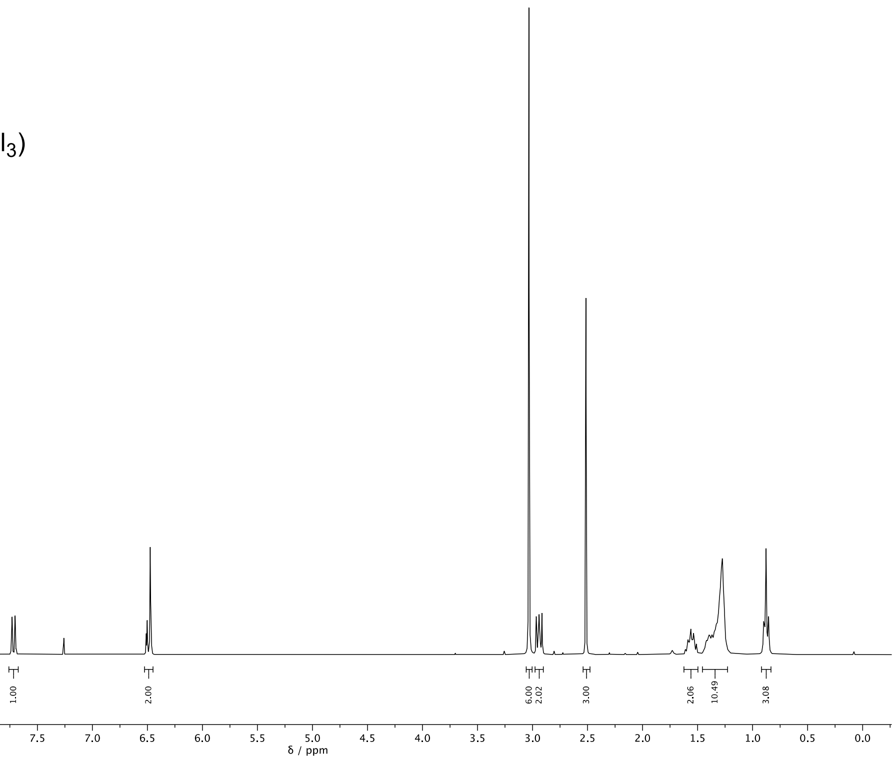

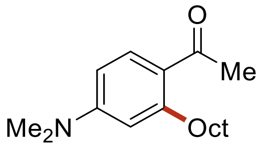

2i

^{13}C NMR (75 MHz, $CDCl_3$)

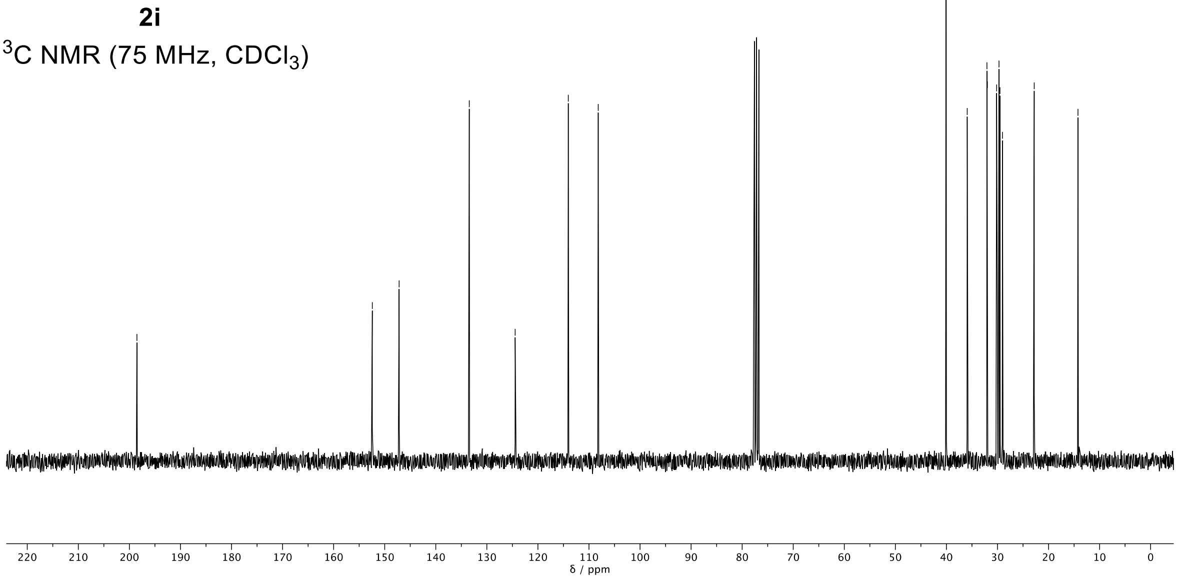

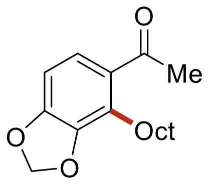

2j

^{1}H NMR (400 MHz, $CDCl_3$)

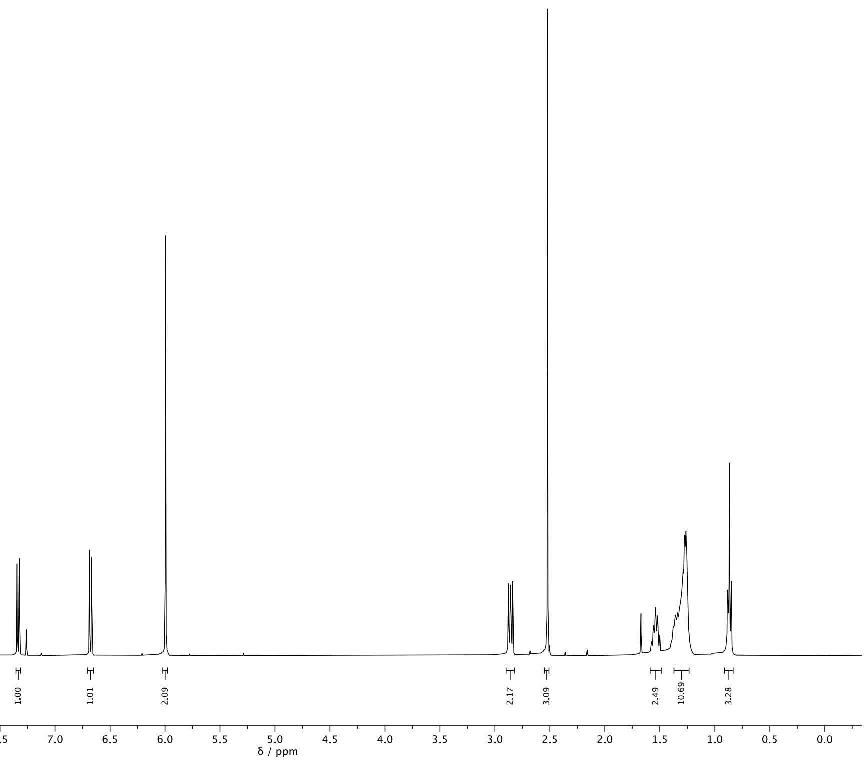

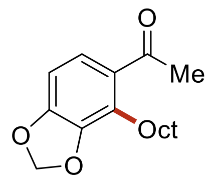

2j

^{13}C NMR (101 MHz, $CDCl_3$)

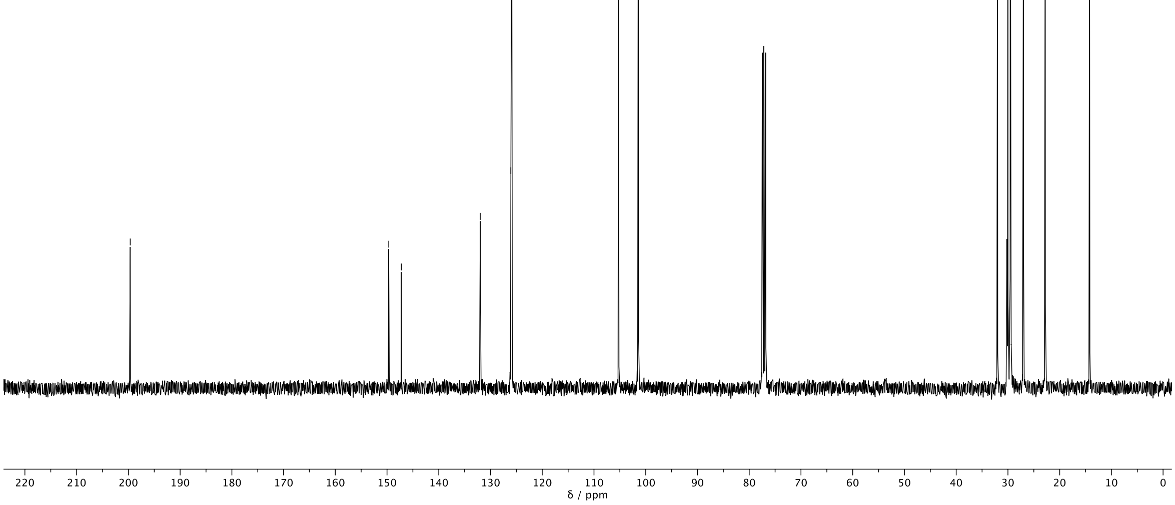

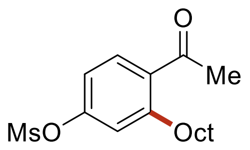

2k

^{1}H NMR (400 MHz, $CDCl_3$)

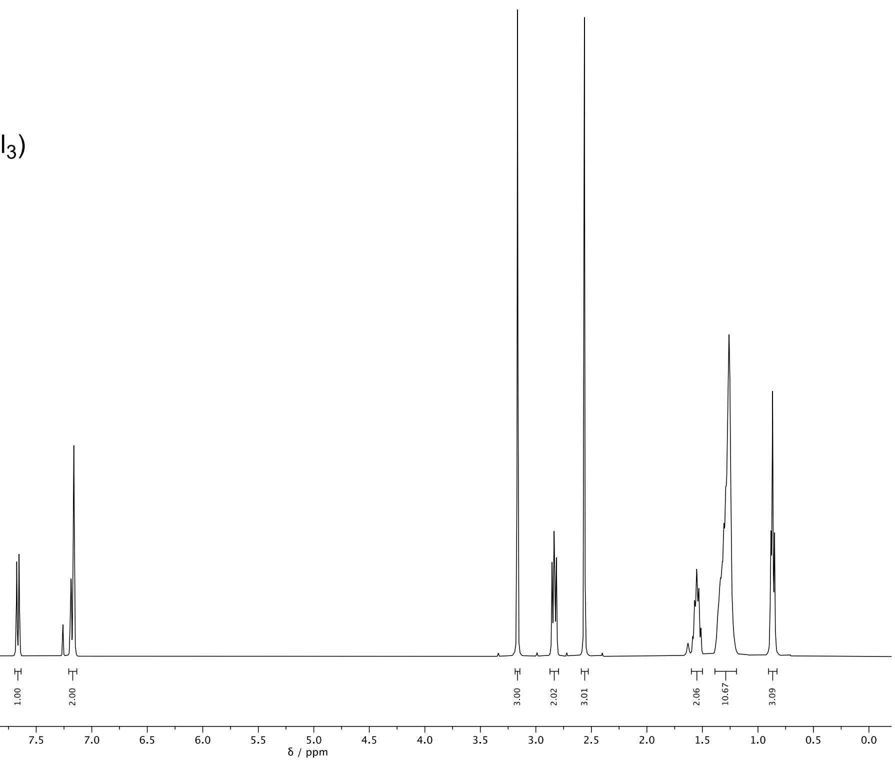

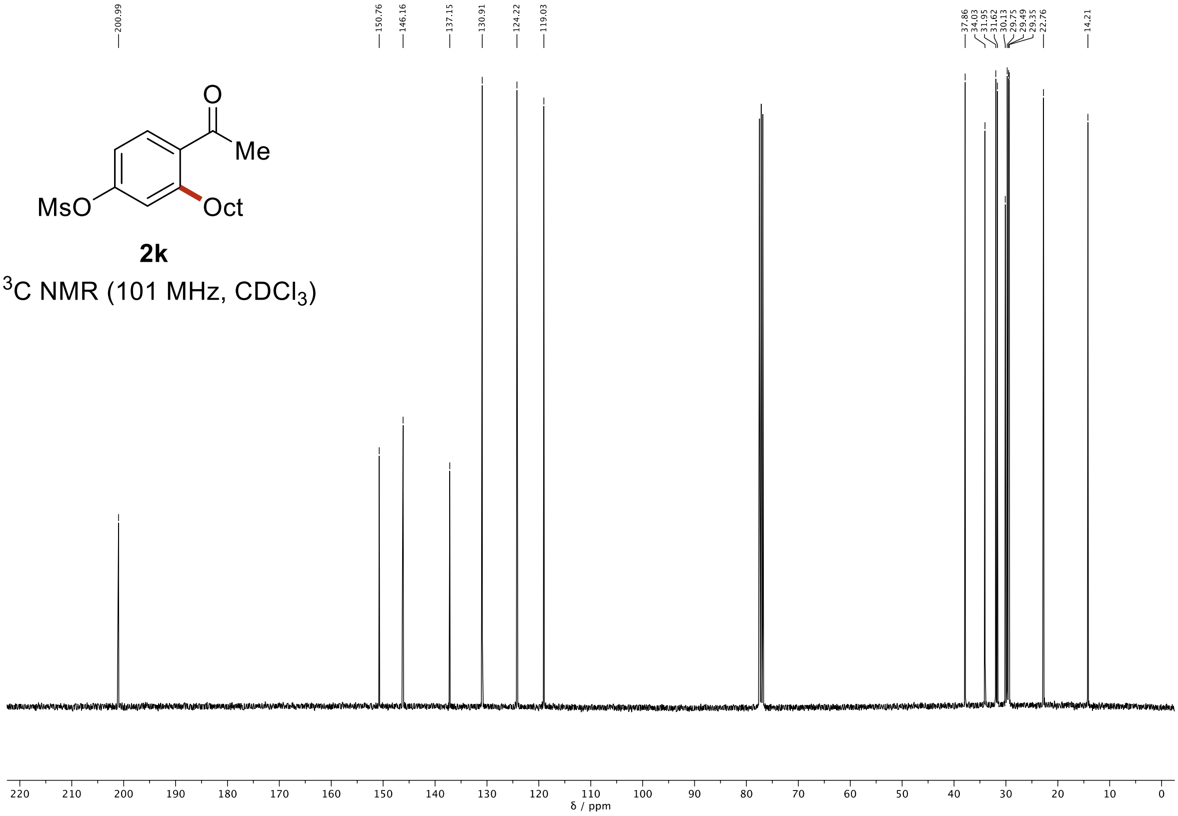

2k

^{13}C NMR (101 MHz, $CDCl_3$)

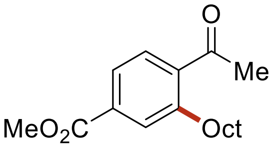

2l

^{1}H NMR (400 MHz, $CDCl_3$)

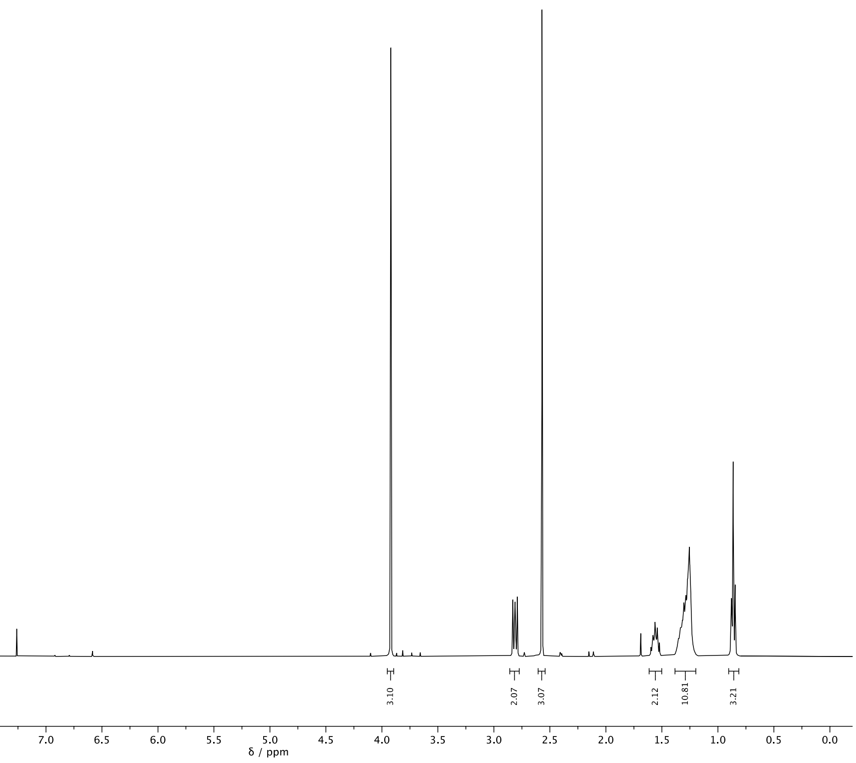

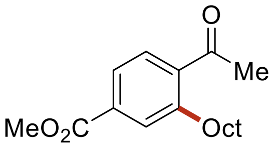

2l

^{13}C NMR (101 MHz, $CDCl_3$)

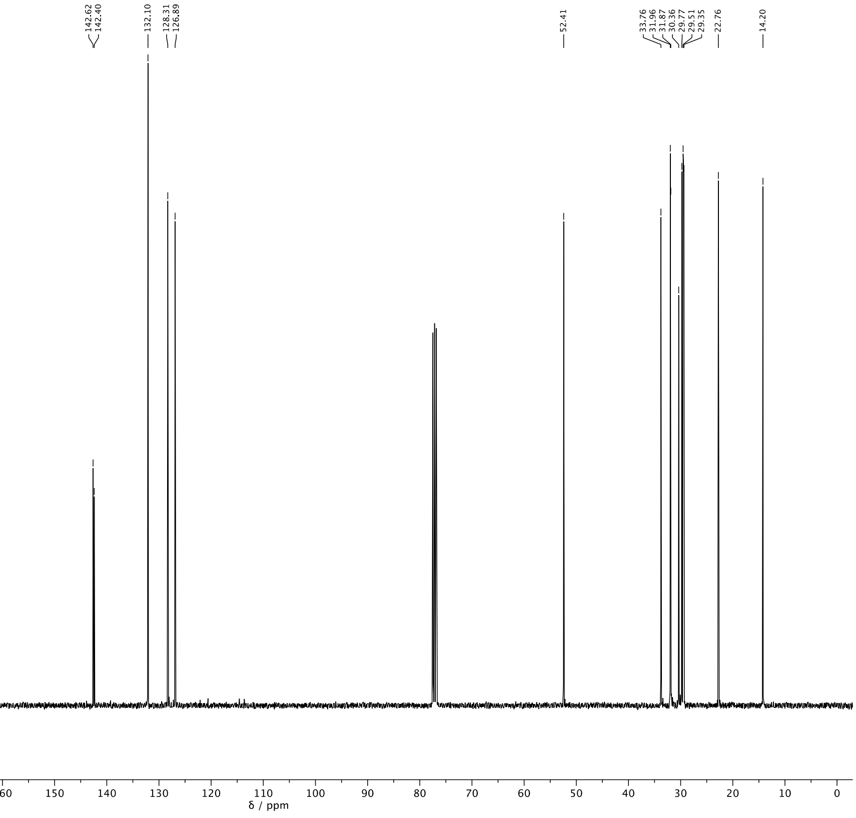

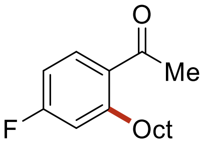

2m

^{1}H NMR (300 MHz, $CDCl_3$)

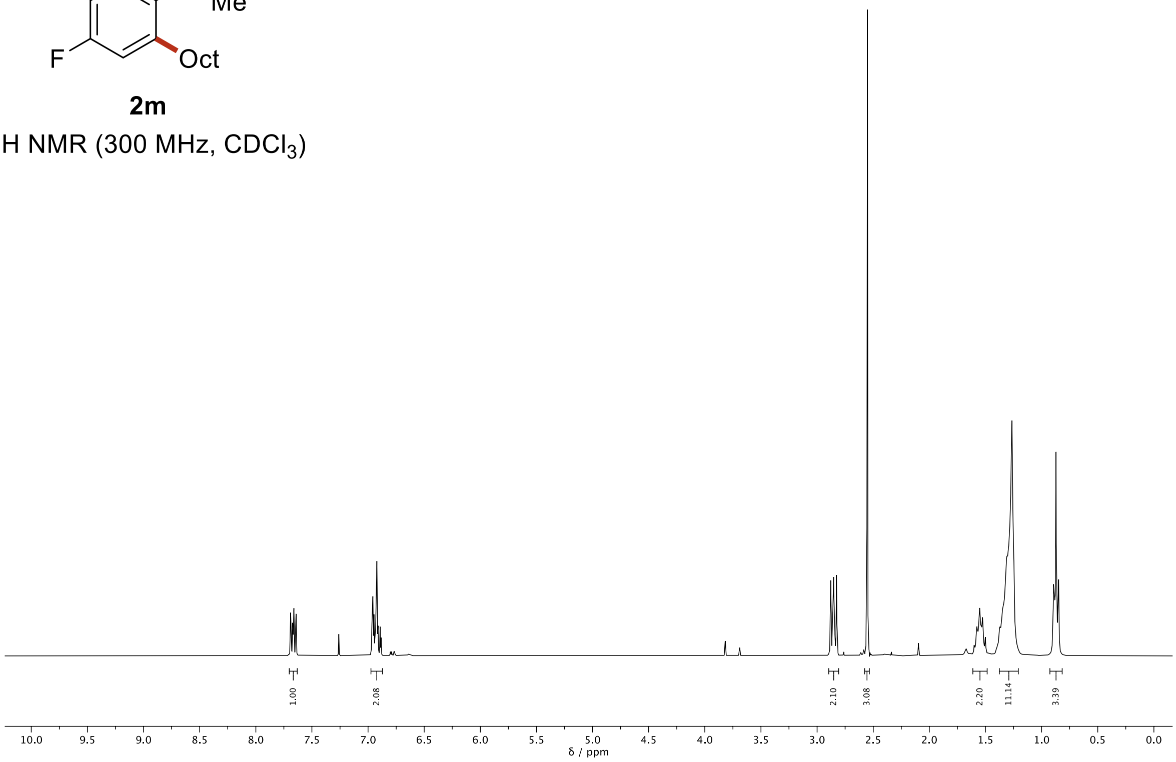

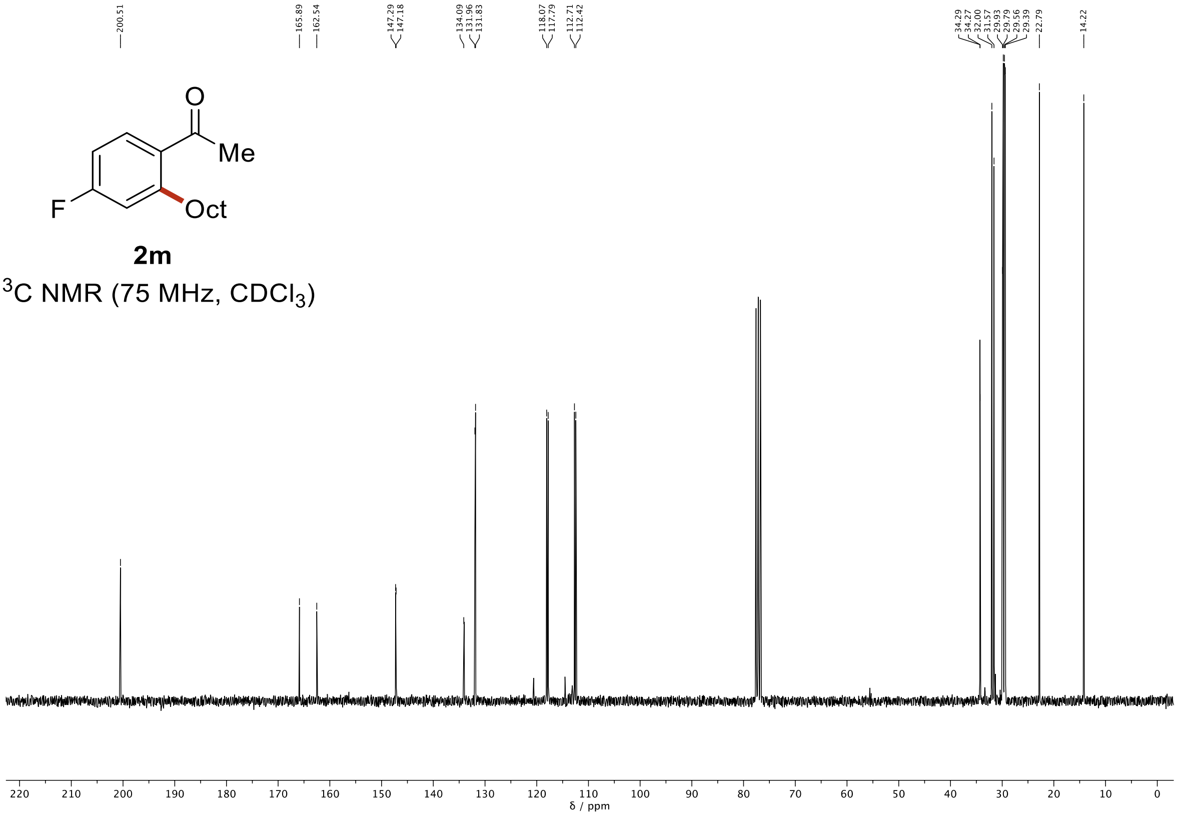

2m

^{13}C NMR (75 MHz, $CDCl_3$)

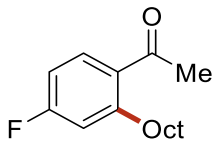

2m

^{19}F NMR (282 MHz, CDCl$_3$)

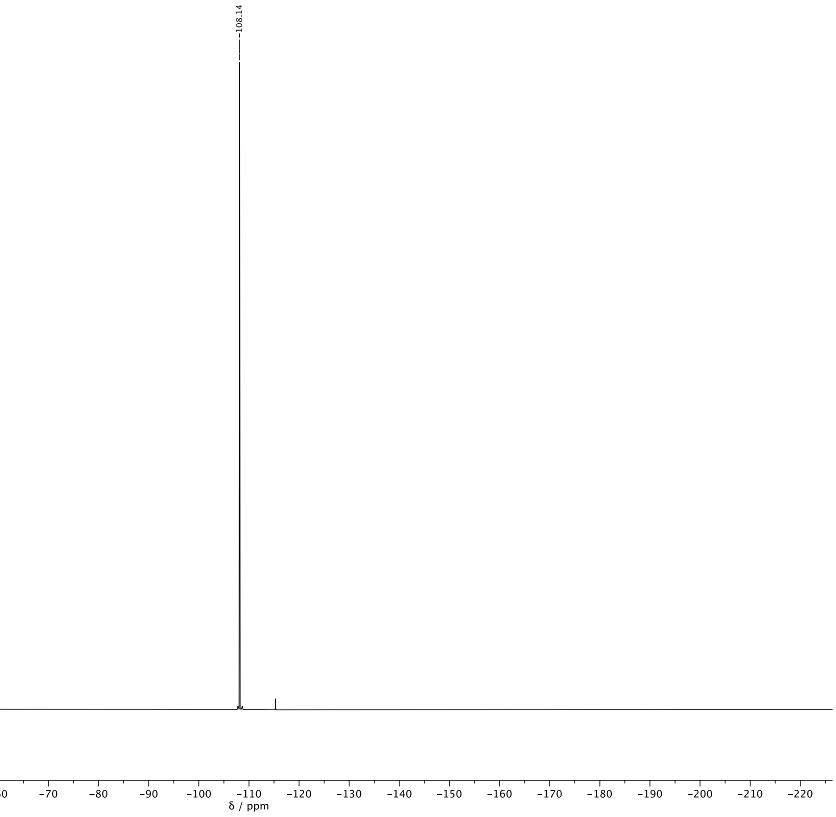

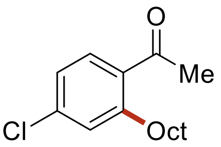

2n

^{1}H NMR (300 MHz, $CDCl_3$)

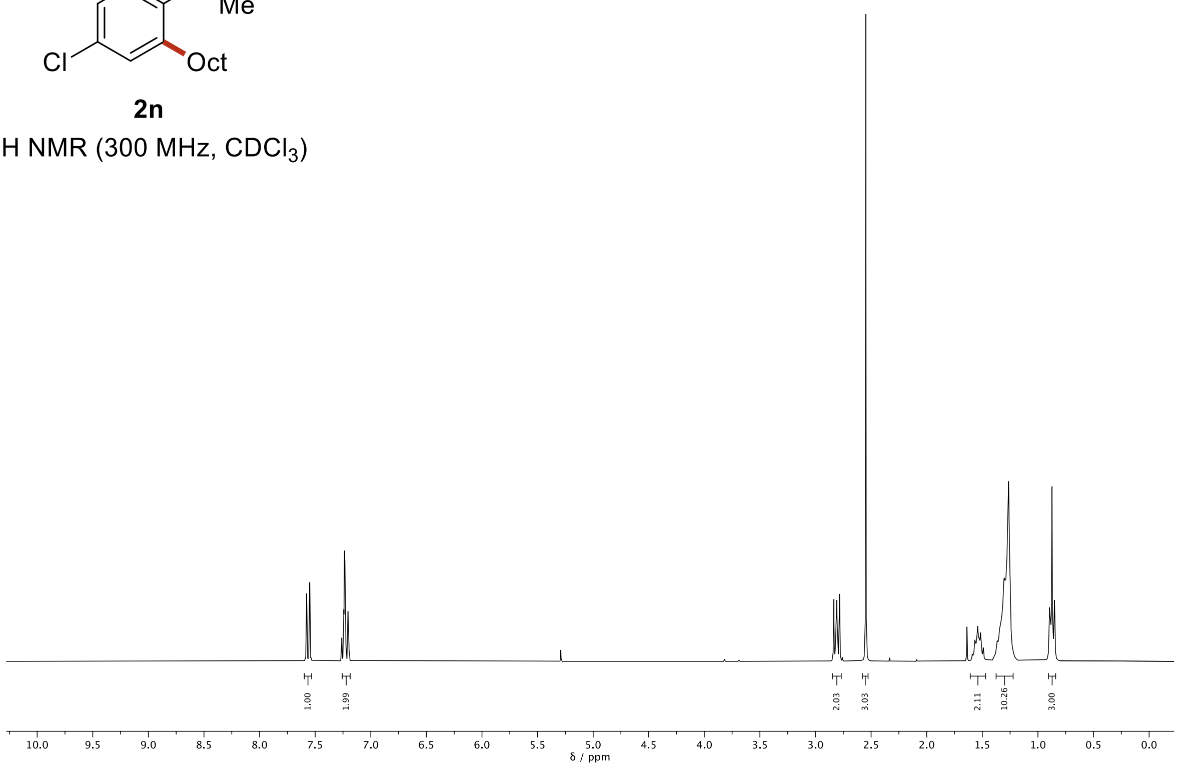

2n

^{13}C NMR (75 MHz, $CDCl_3$)

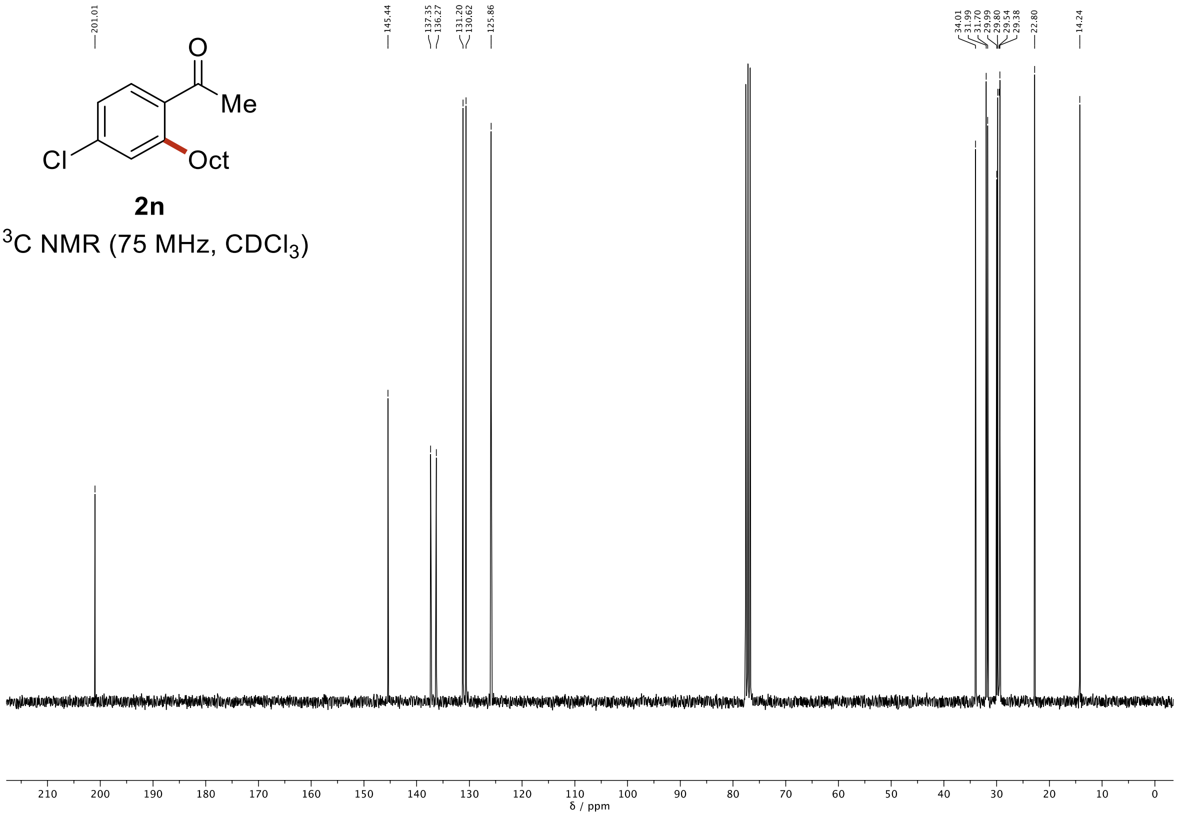

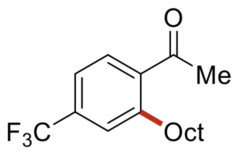

2o

^{1}H NMR (300 MHz, $CDCl_3$)

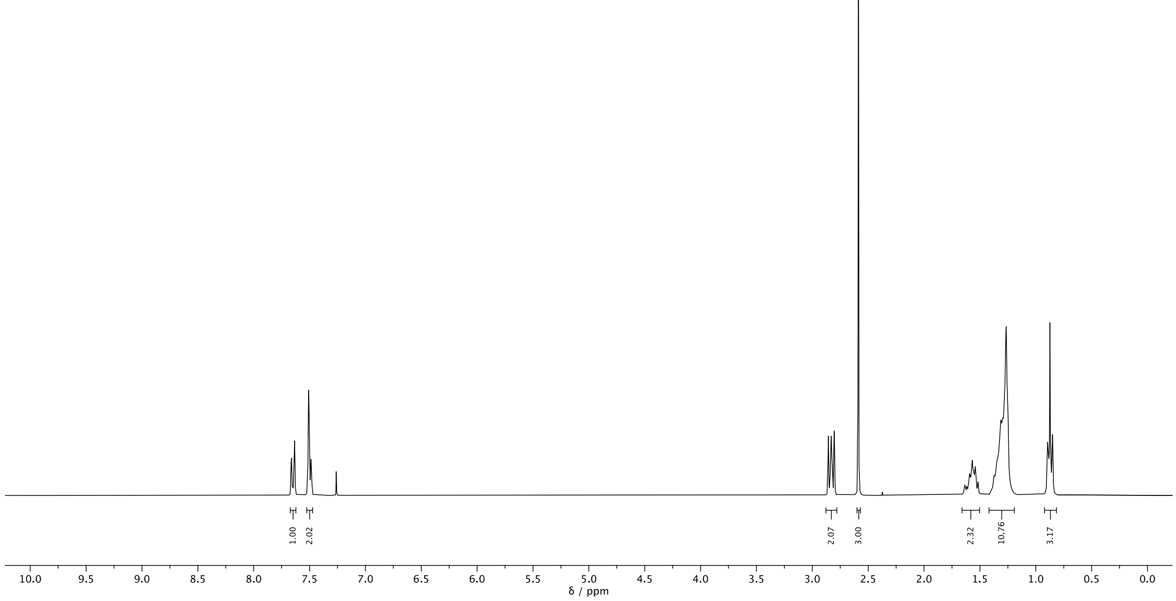

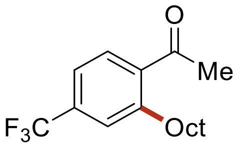

2o

^{13}C NMR (75 MHz, $CDCl_3$)

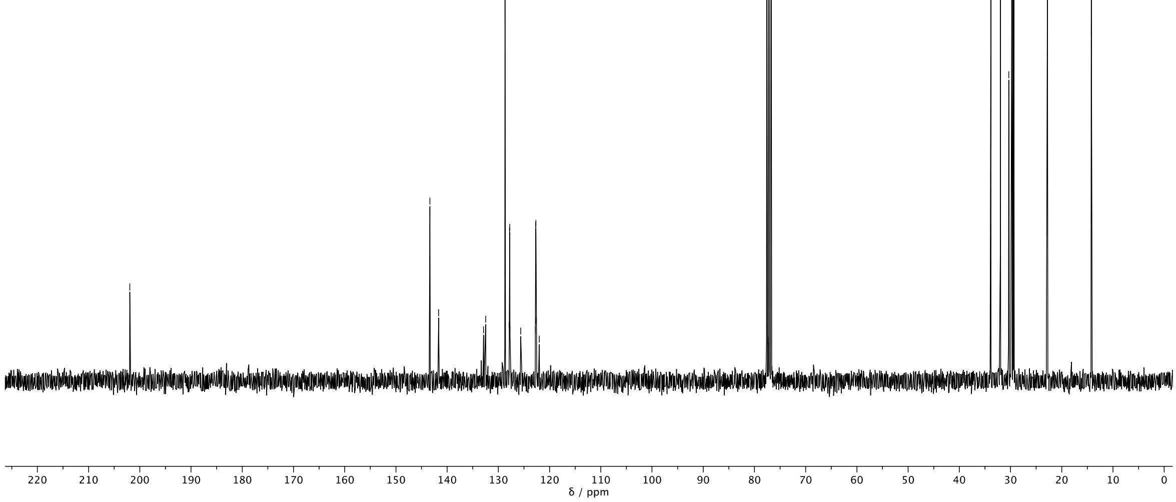

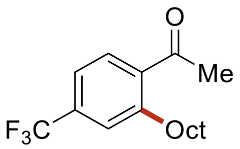

2o

^{19}F NMR (282 MHz, $CDCl_3$)

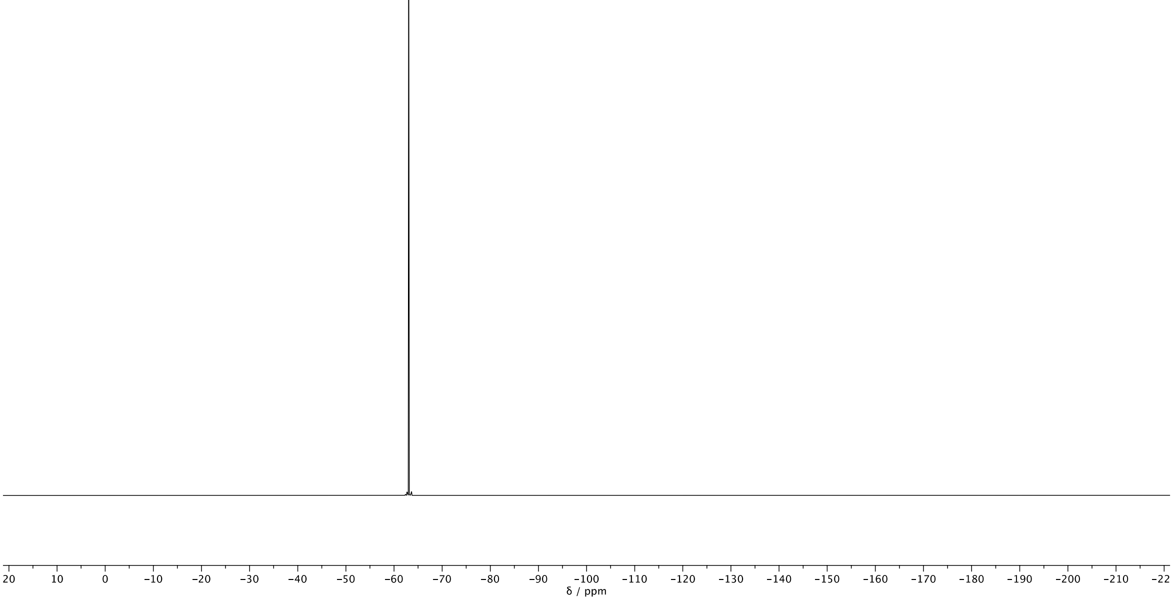

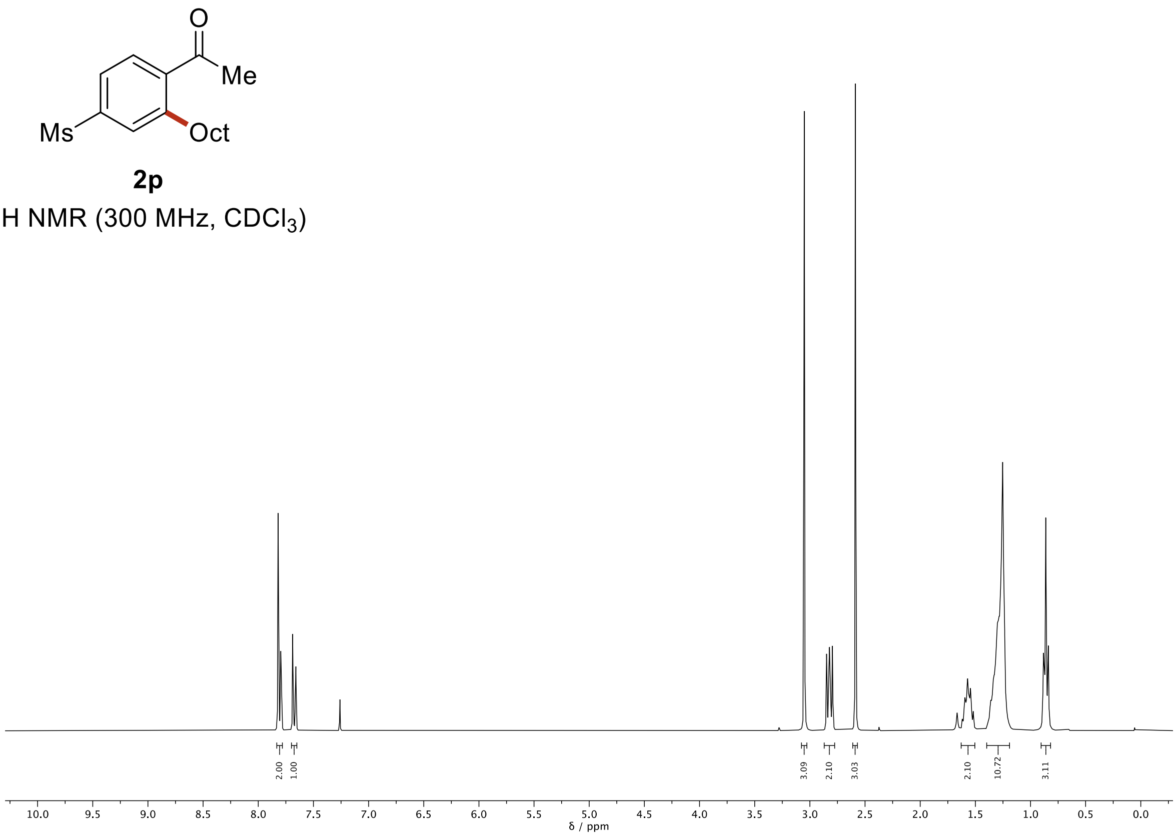

2p

^{1}H NMR (300 MHz, $CDCl_3$)

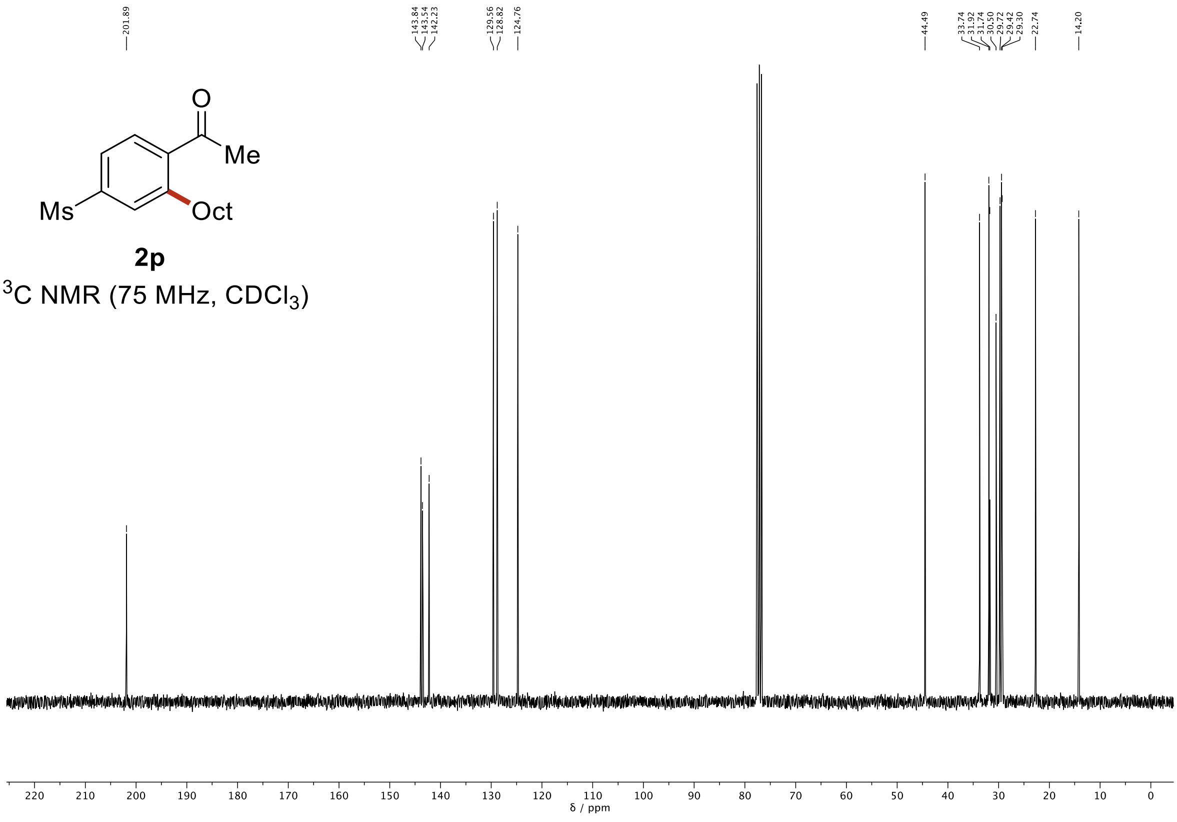

2p

^{13}C NMR (75 MHz, $CDCl_3$)

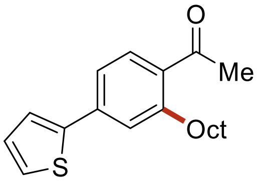

2q

^{1}H NMR (300 MHz, $CDCl_3$)

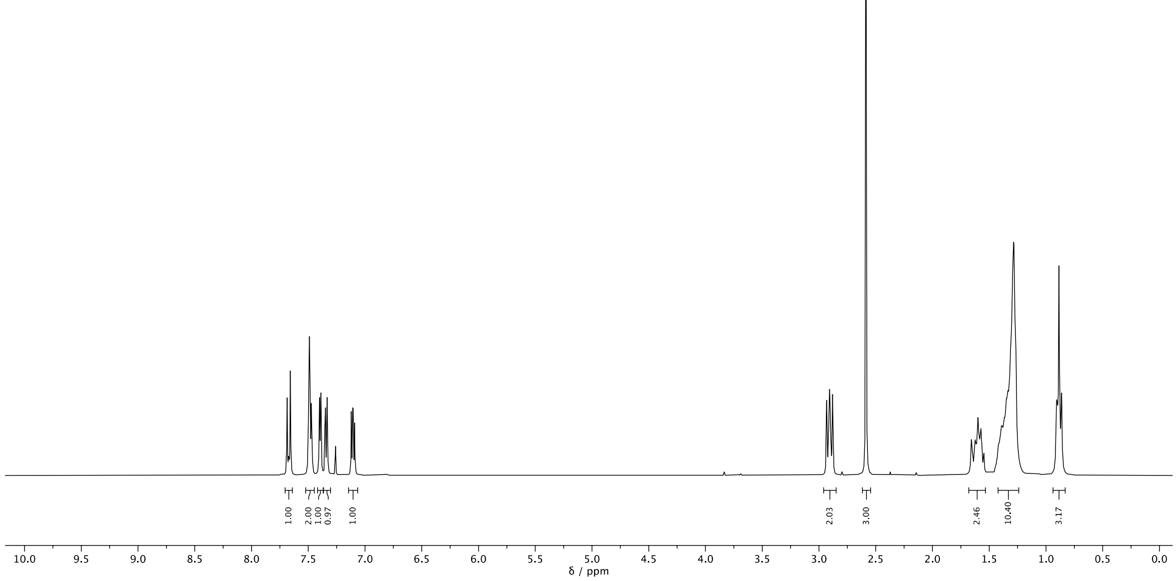

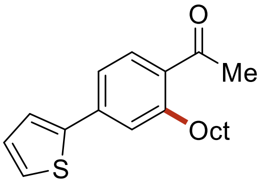

2q

^{13}C NMR (75 MHz, $CDCl_3$)

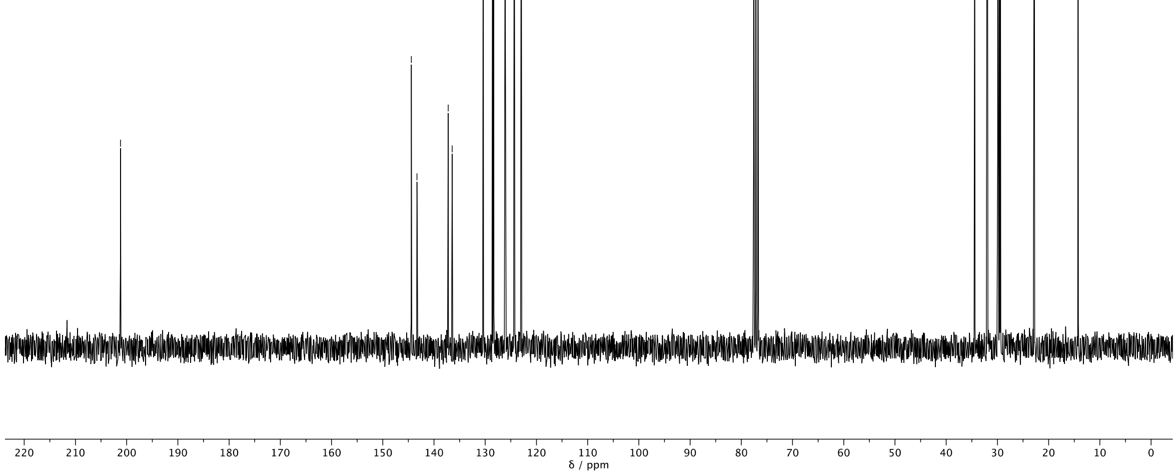

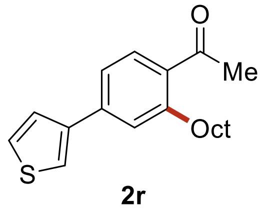

2r

^{1}H NMR (300 MHz, CDCl$_3$)

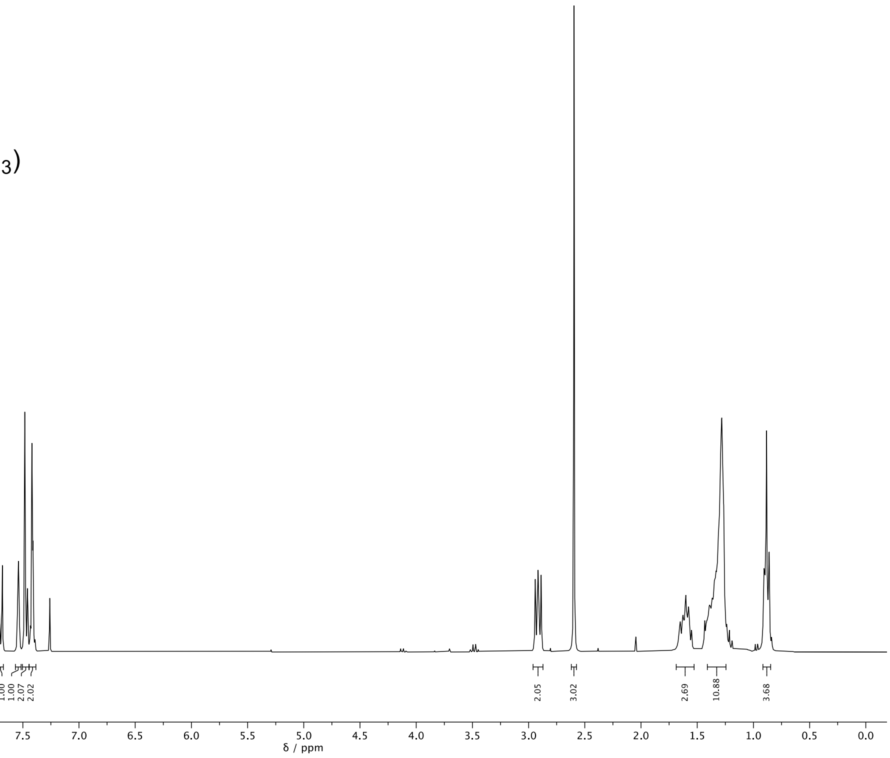

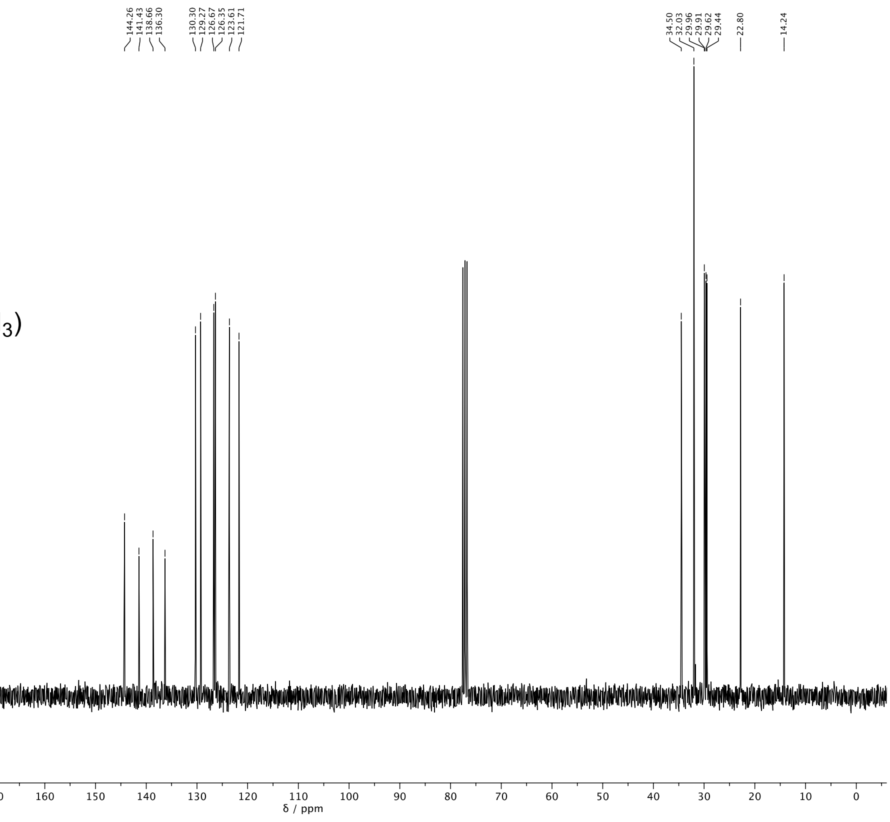

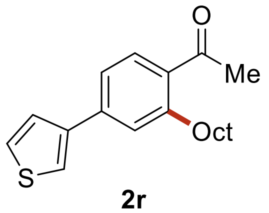

2r

^{13}C NMR (75 MHz, CDCl$_3$)

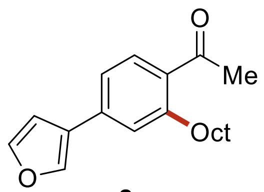

2s

^{1}H NMR (400 MHz, $CDCl_3$)

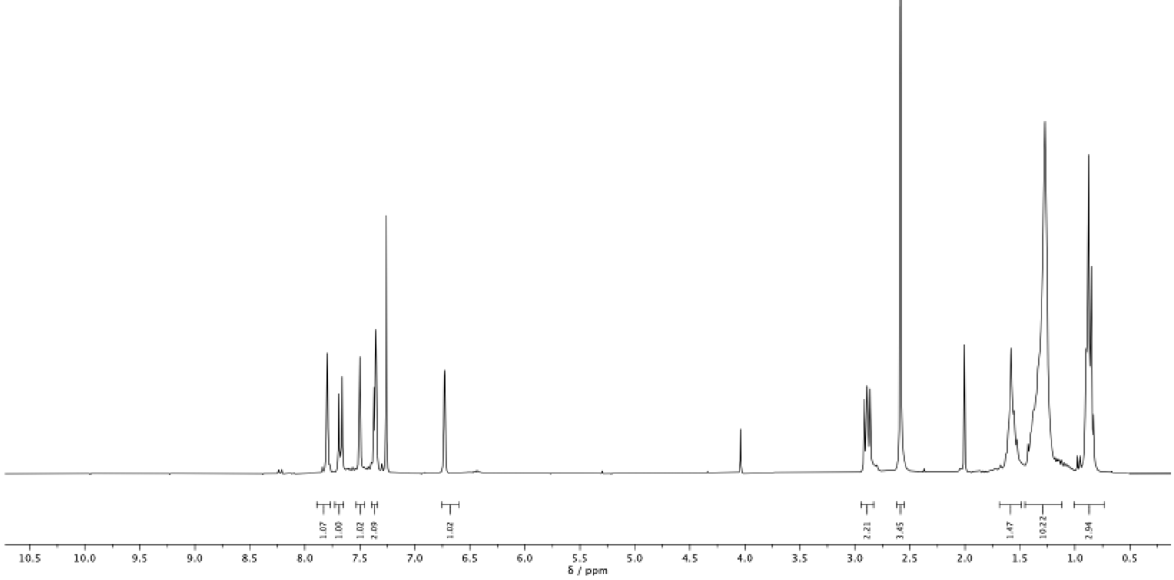

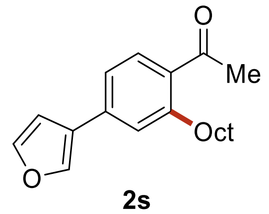

2s

^{13}C NMR (101 MHz, $CDCl_3$)

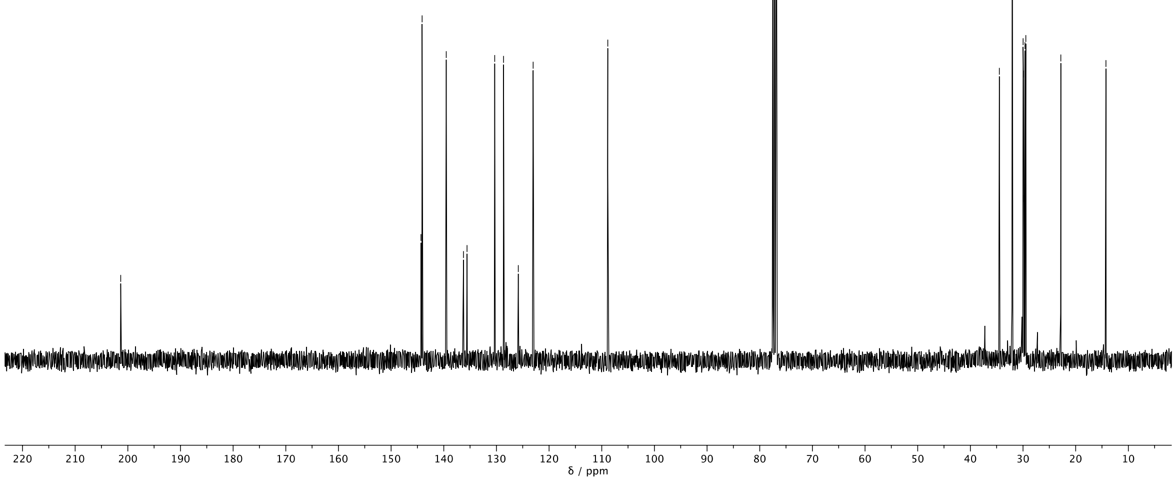

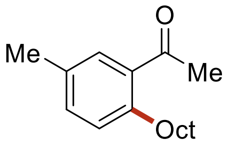

2t

^{1}H NMR (300 MHz, $CDCl_3$)

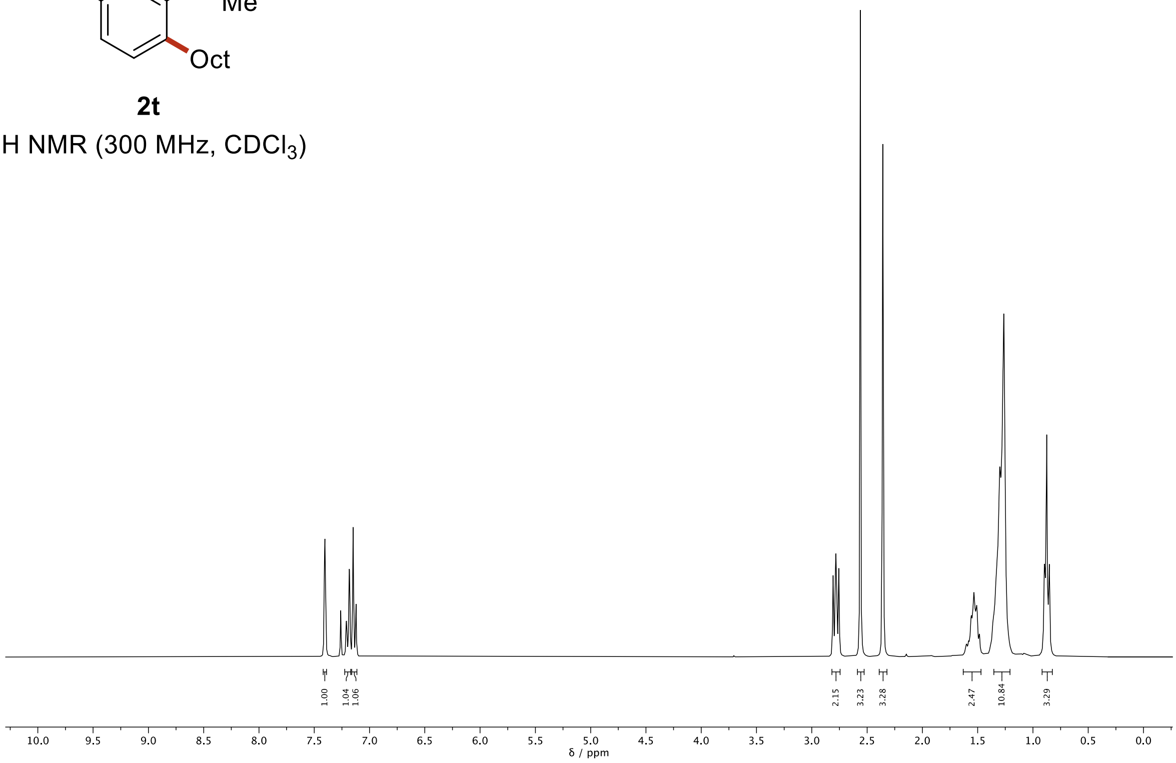

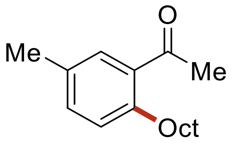

2t

^{13}C NMR (75 MHz, $CDCl_3$)

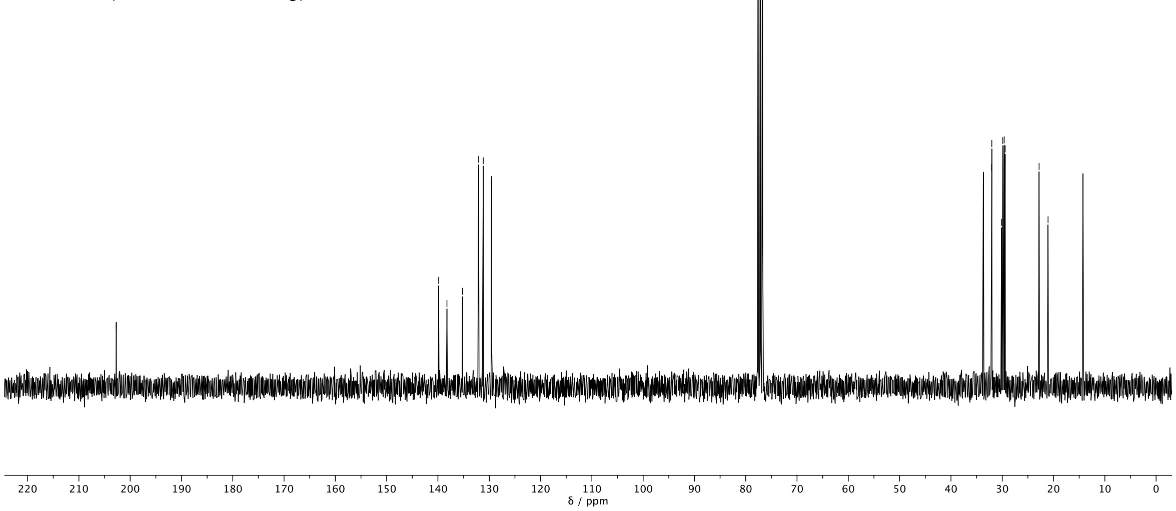

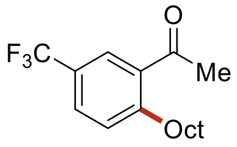

2u

^{1}H NMR (400 MHz, CDCl$_3$)

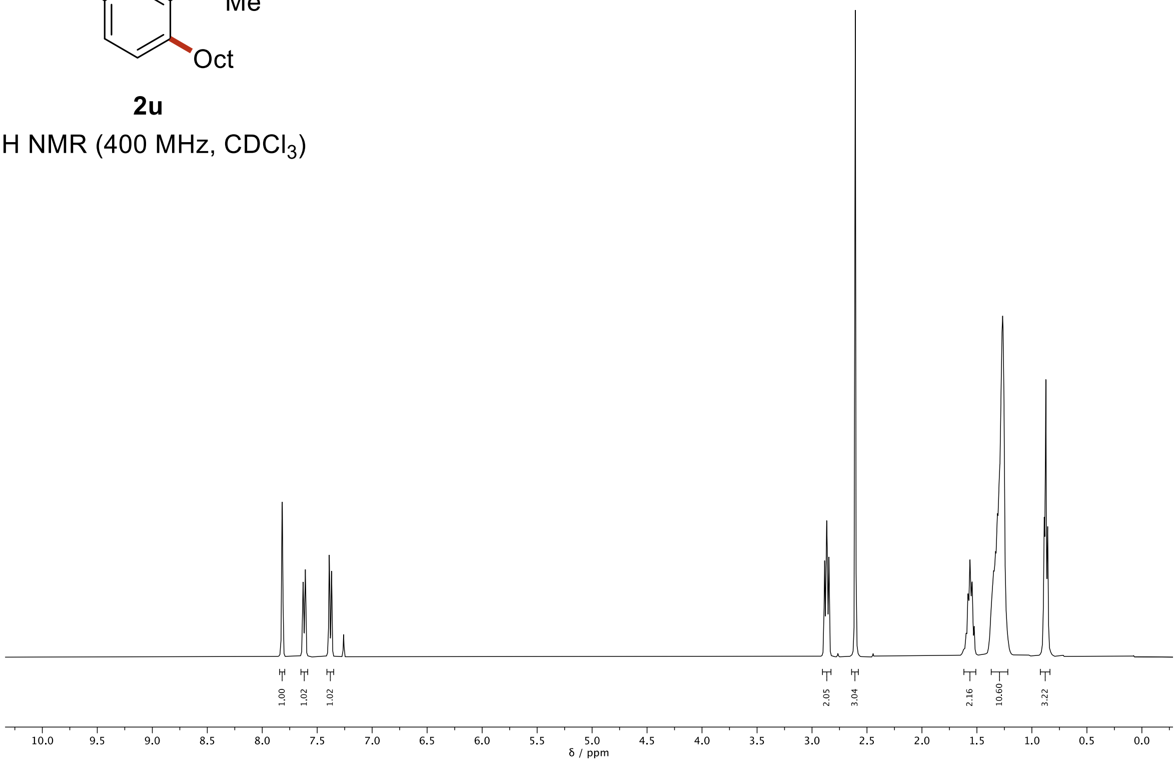

2u

^{13}C NMR (101 MHz, CDCl$_3$)

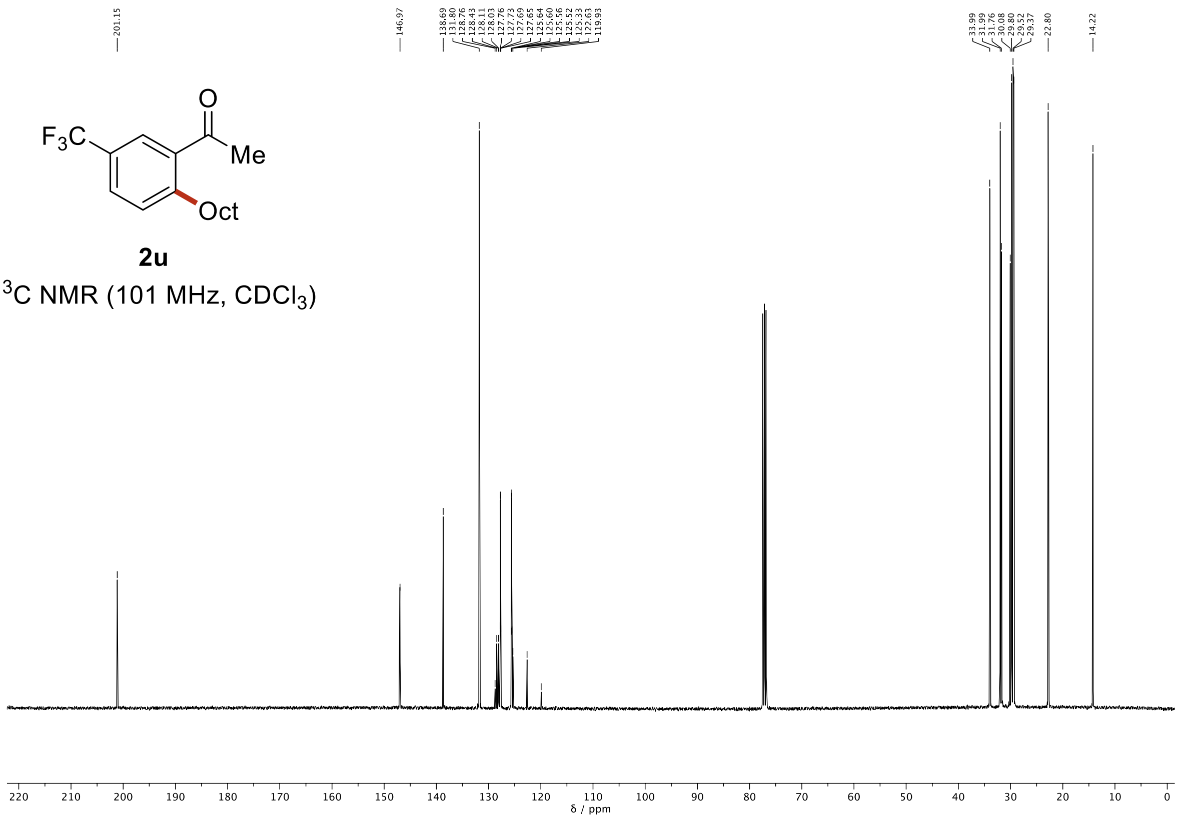

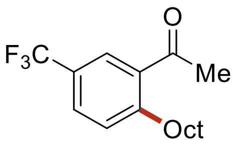

2u

^{19}F NMR (282 MHz, $CDCl_3$)

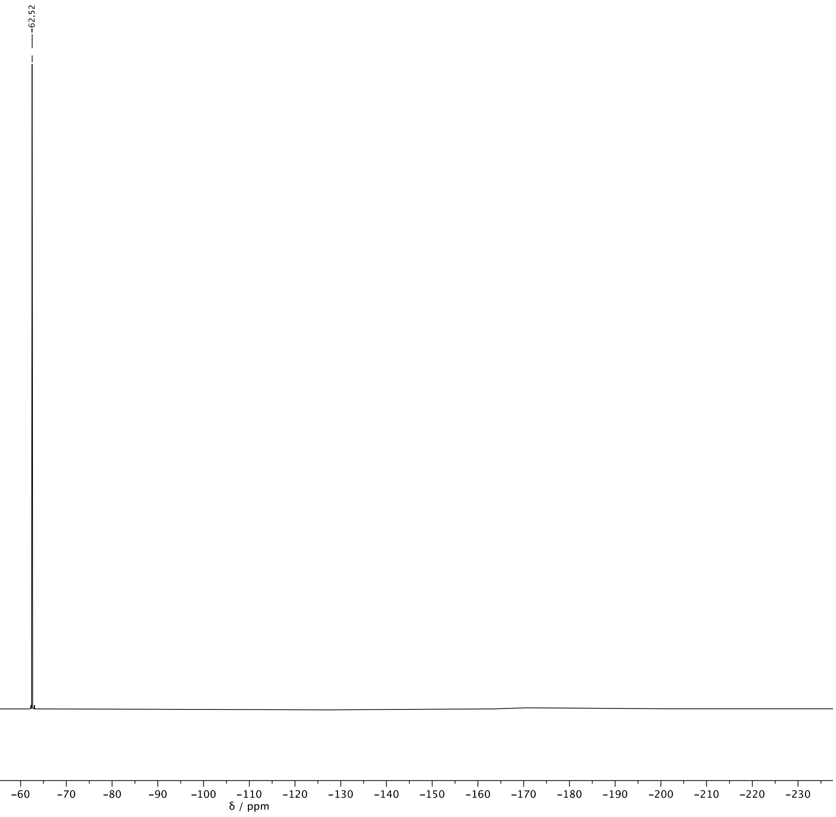

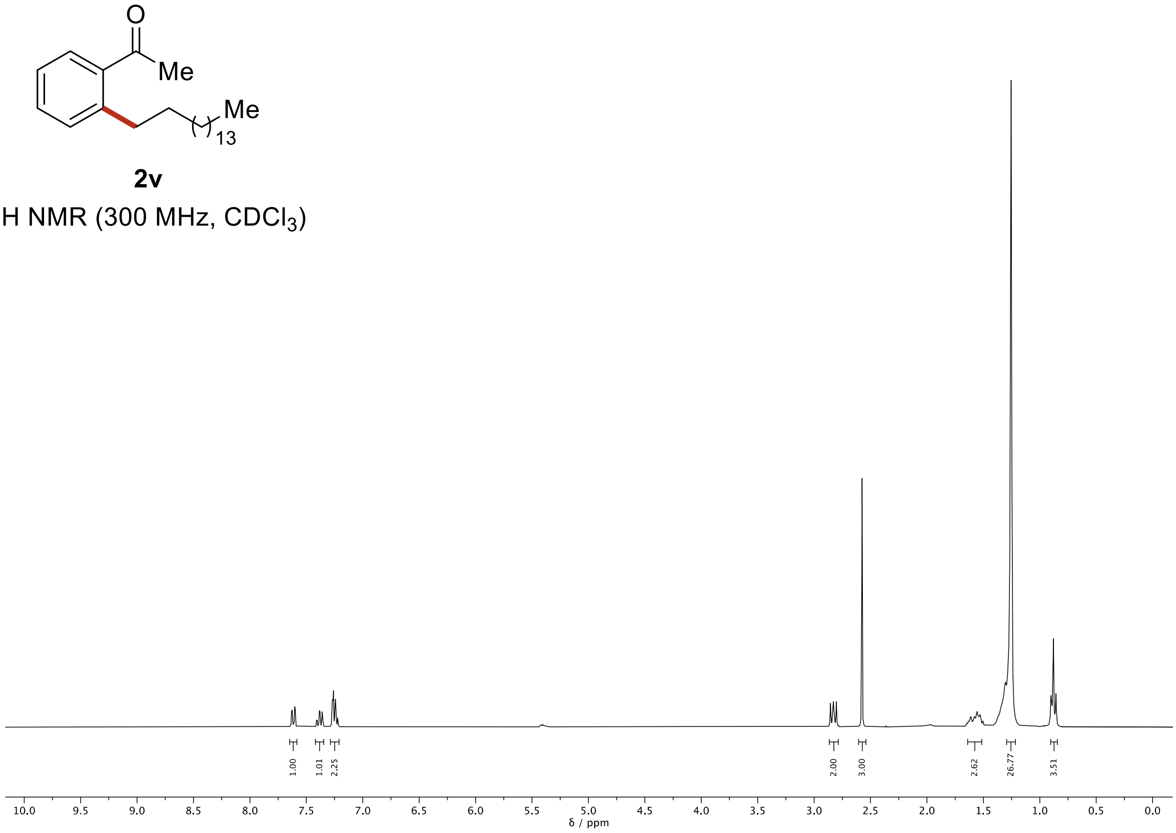

2v

^{1}H NMR (300 MHz, $CDCl_3$)

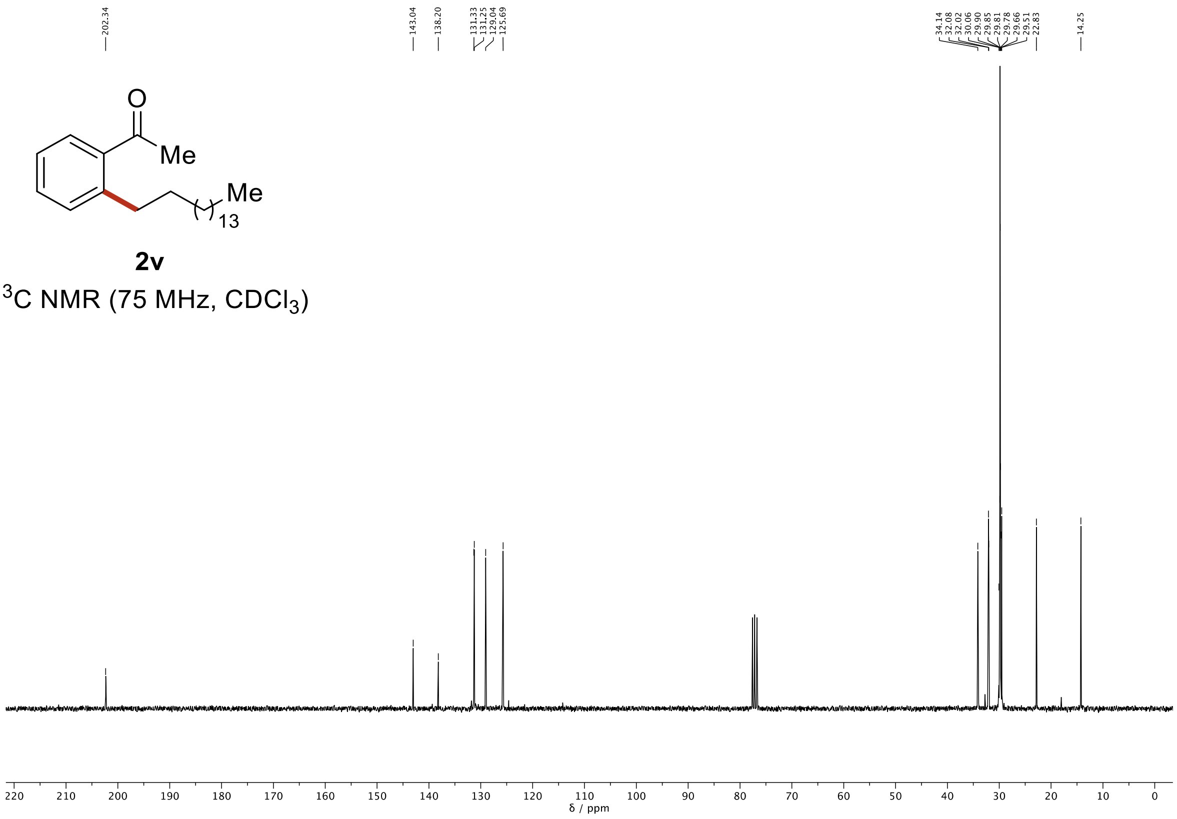

2v

^{13}C NMR (75 MHz, $CDCl_3$)

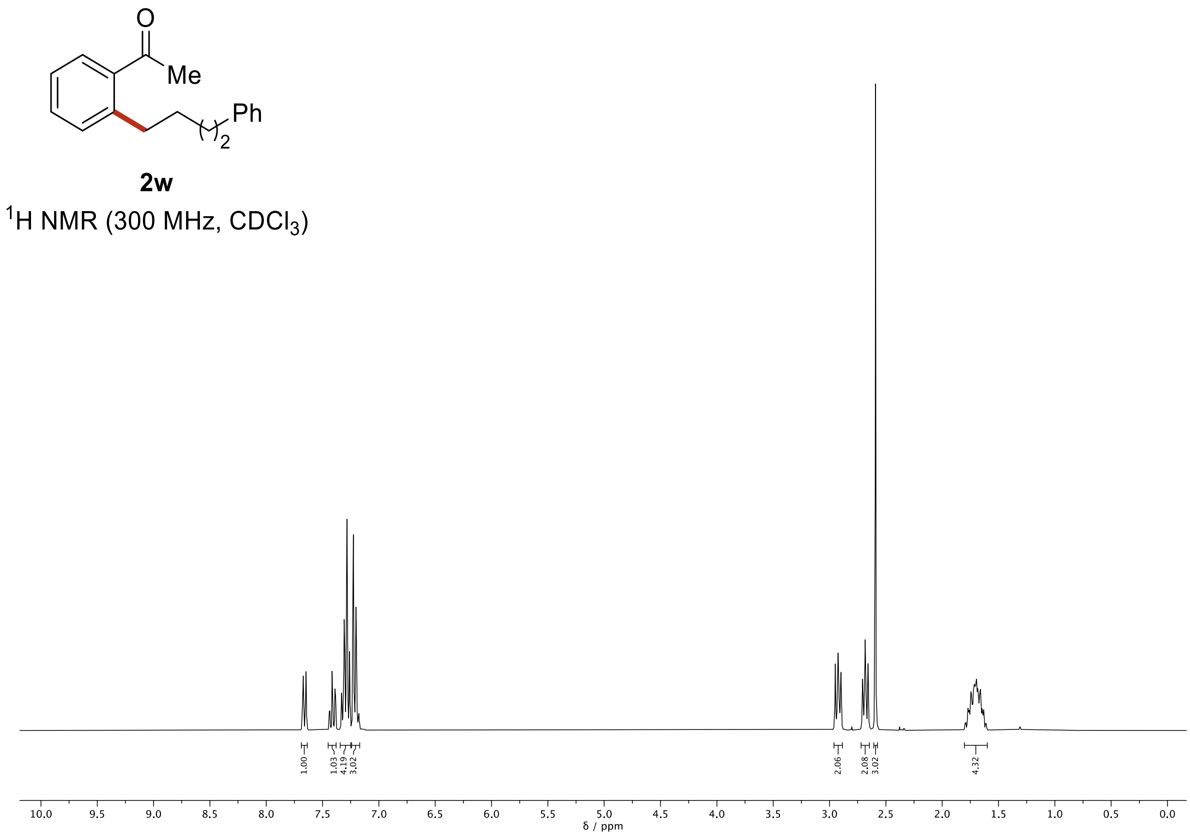
O
Me
Ph
2
2w
^{1}H NMR (300 MHz, $CDCl_3$)
1.00
1.03
4.19
3.02
2.06
2.08
3.02
4.32
10.0 9.5 9.0 8.5 8.0 7.5 7.0 6.5 6.0 5.5 5.0 4.5 4.0 3.5 3.0 2.5 2.0 1.5 1.0 0.5 0.0
δ / ppm

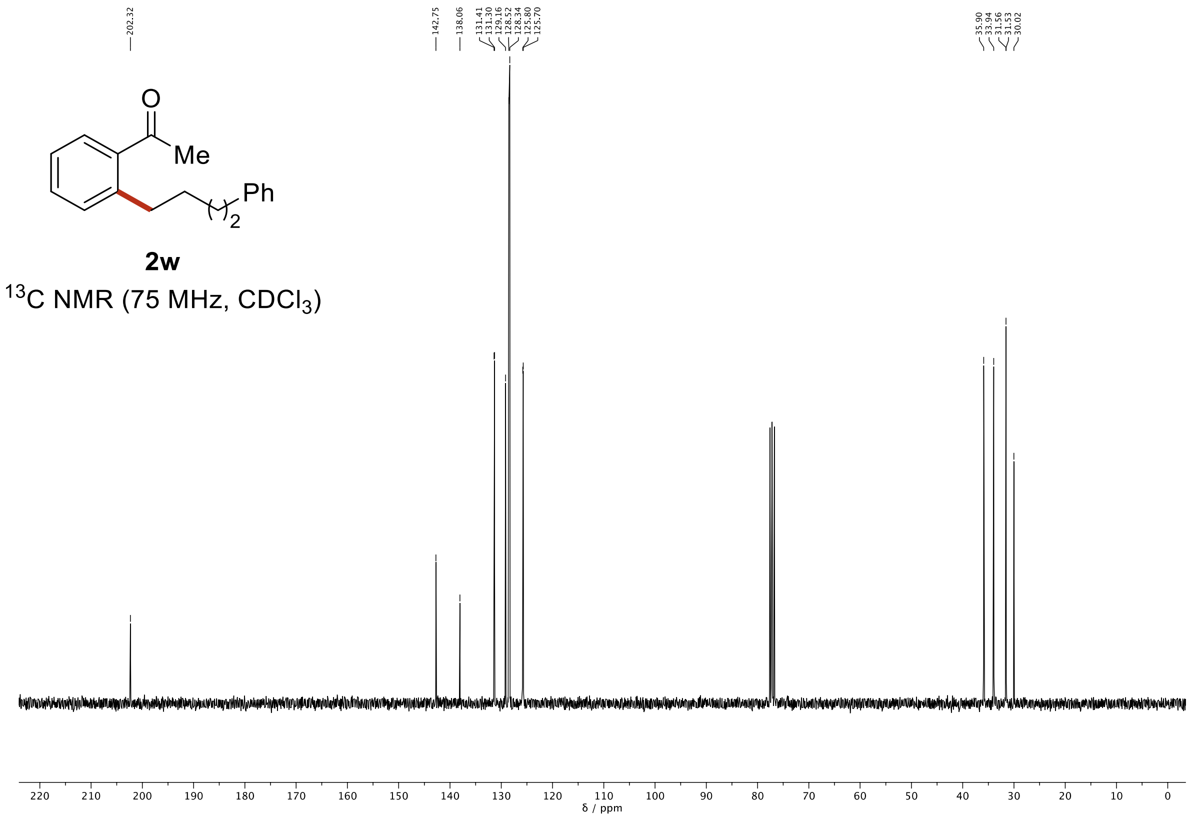
202.32
142.75
138.06
131.41
131.30
129.16
128.52
128.34
125.80
125.70
35.90
33.94
31.56
31.53
30.02
O
Me
Ph
2
2w
^{13}C NMR (75 MHz, $CDCl_3$)
220 210 200 190 180 170 160 150 140 130 120 110 100 90 80 70 60 50 40 30 20 10 0
δ / ppm

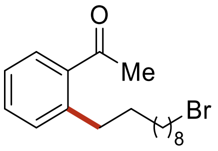

2x

^{1}H NMR (300 MHz, $CDCl_3$)

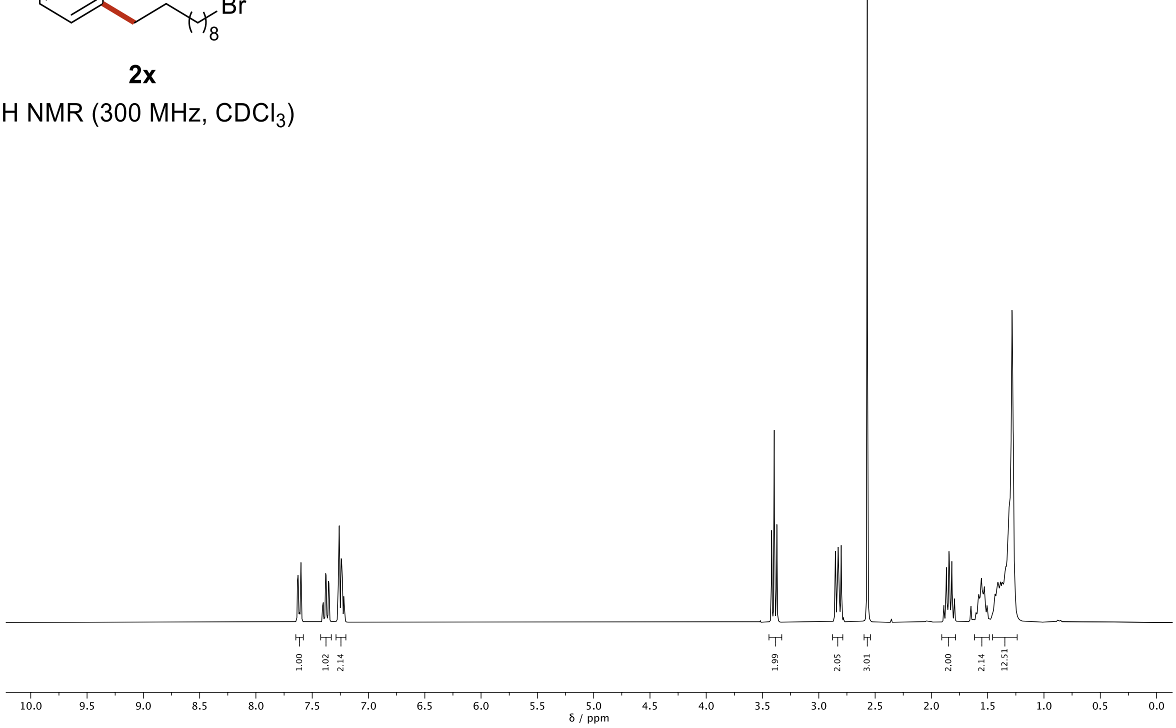

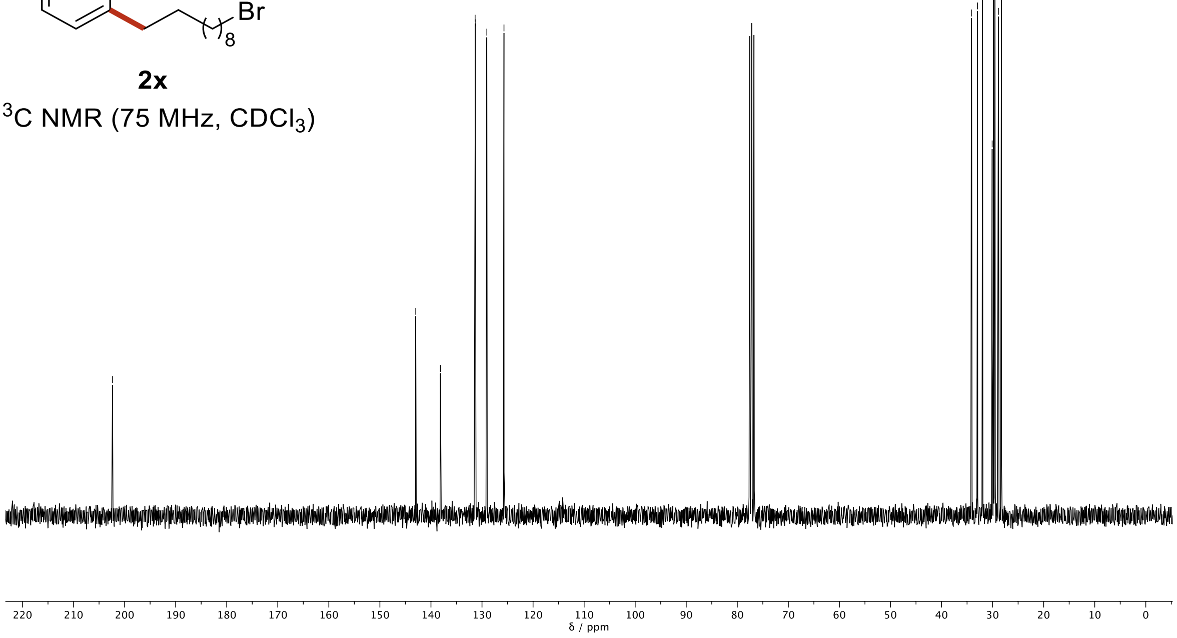

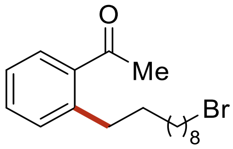

2x

^{13}C NMR (75 MHz, $CDCl_3$)

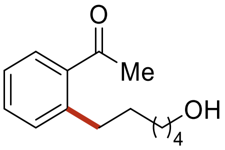

2y

^{1}H NMR (400 MHz, $CDCl_3$)

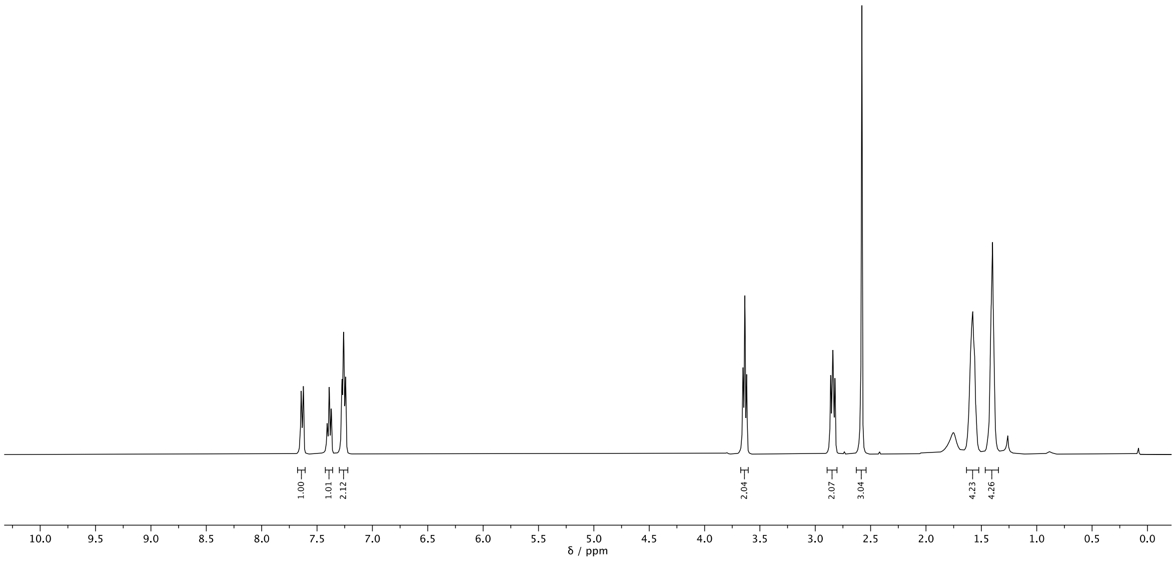

2y

^{13}C NMR (101 MHz, $CDCl_3$)

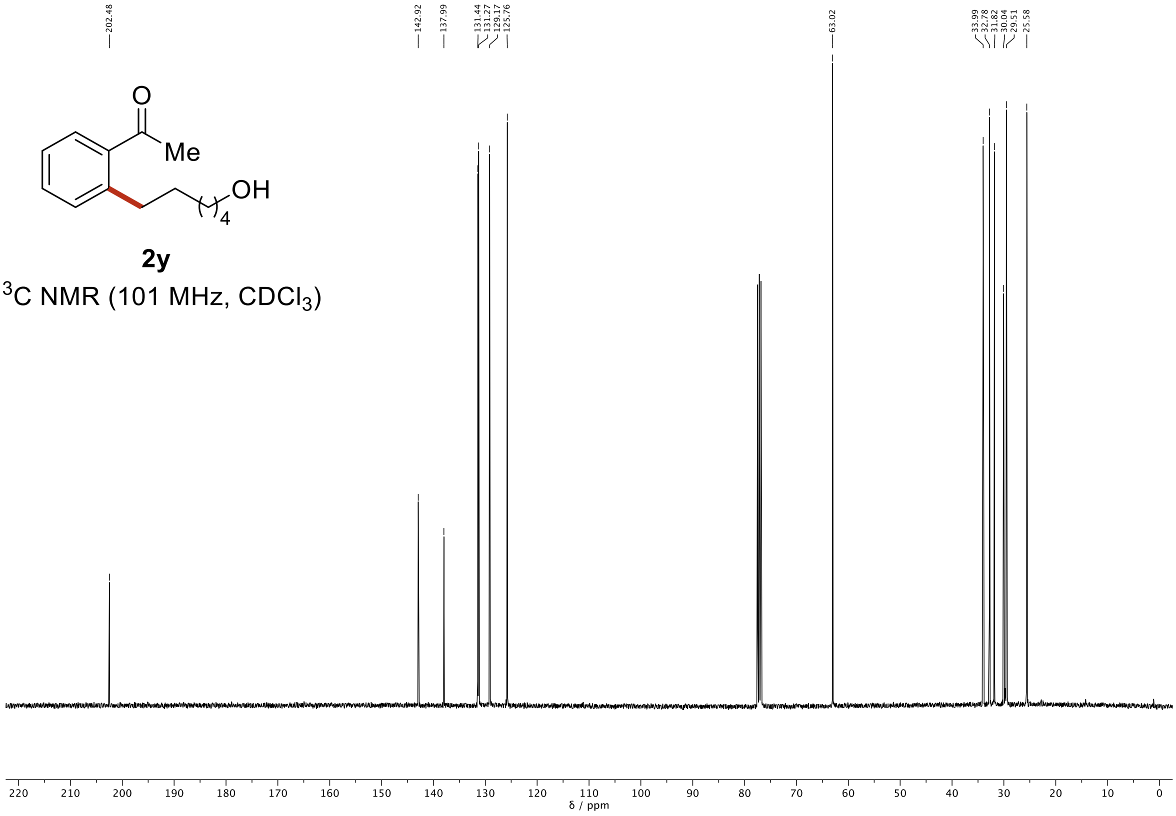

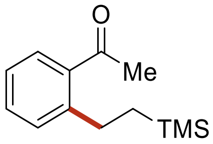

2z

^{1}H NMR (300 MHz, $CDCl_3$)

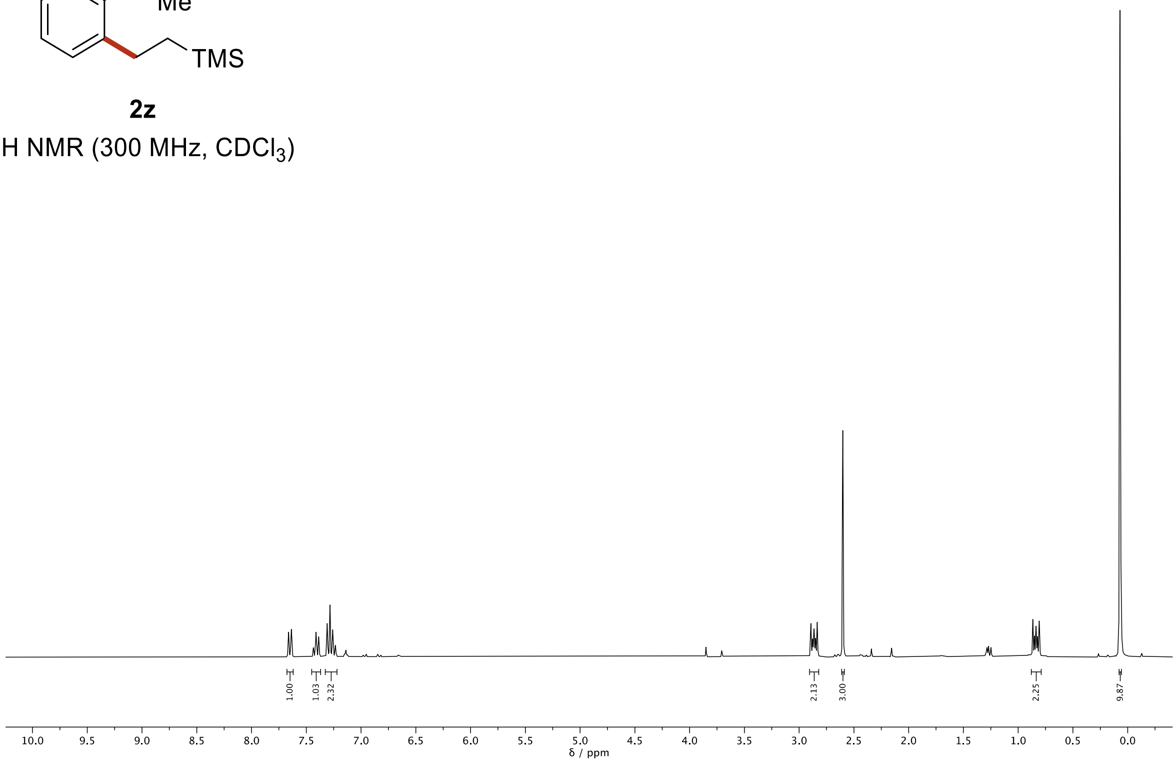

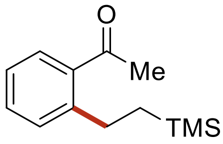

2z

^{13}C NMR (75 MHz, $CDCl_3$)

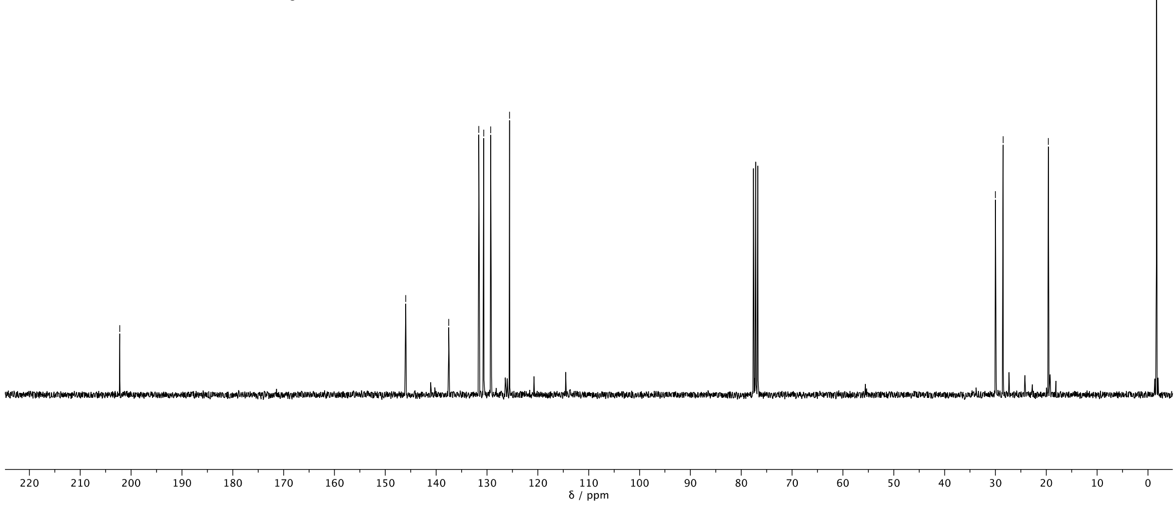

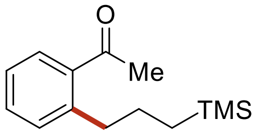

2aa

^{1}H NMR (300 MHz, $CDCl_3$)

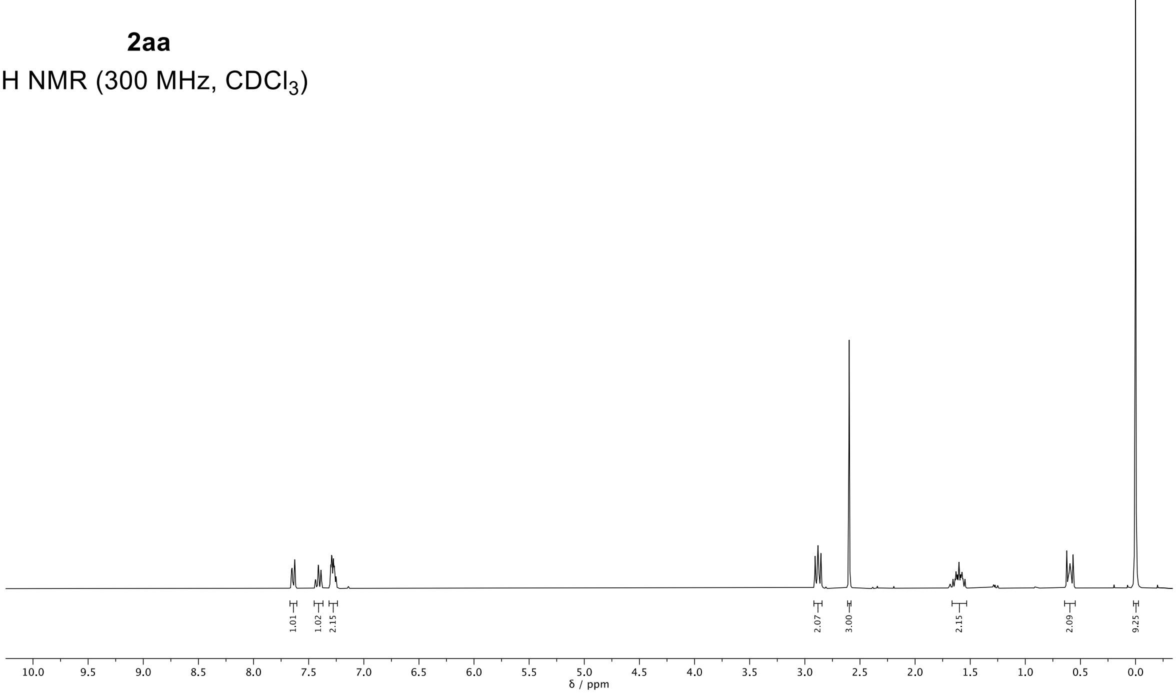

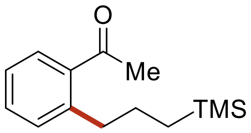

2aa

^{13}C NMR (75 MHz, $CDCl_3$)

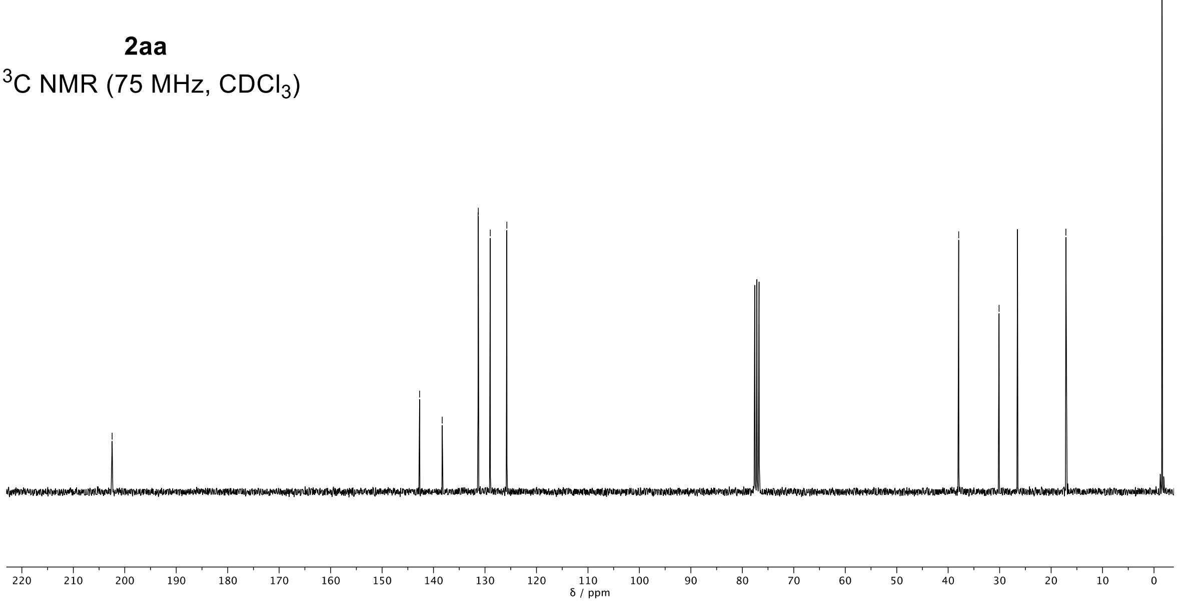

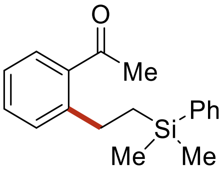

2ab

^{1}H NMR (400 MHz, $CDCl_3$)

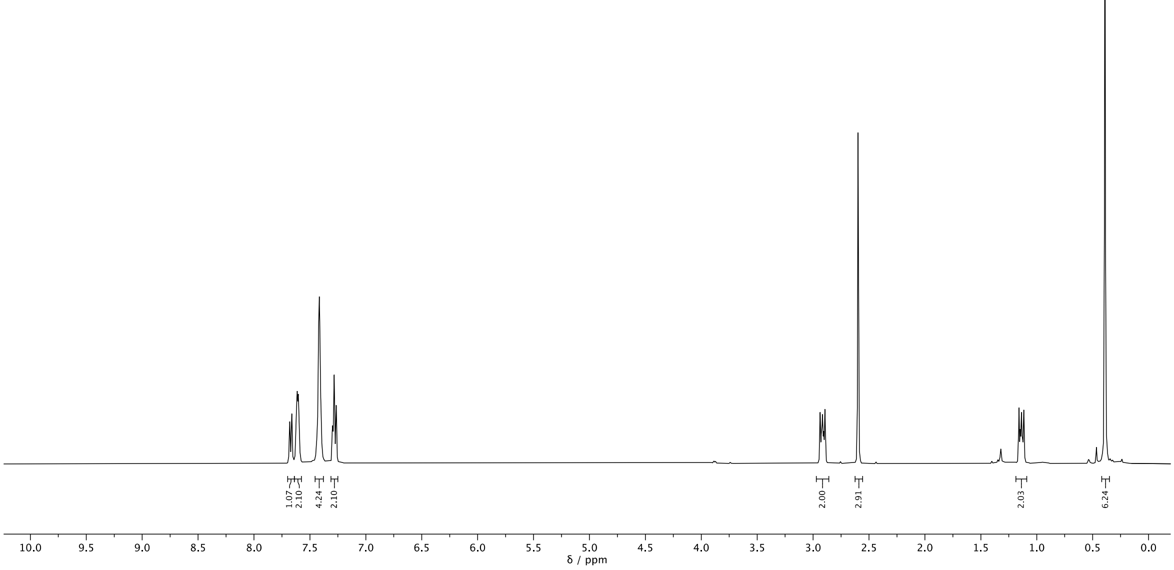

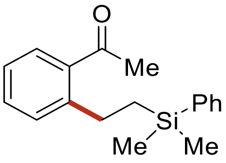

2ab

^{13}C NMR (101 MHz, $CDCl_3$)

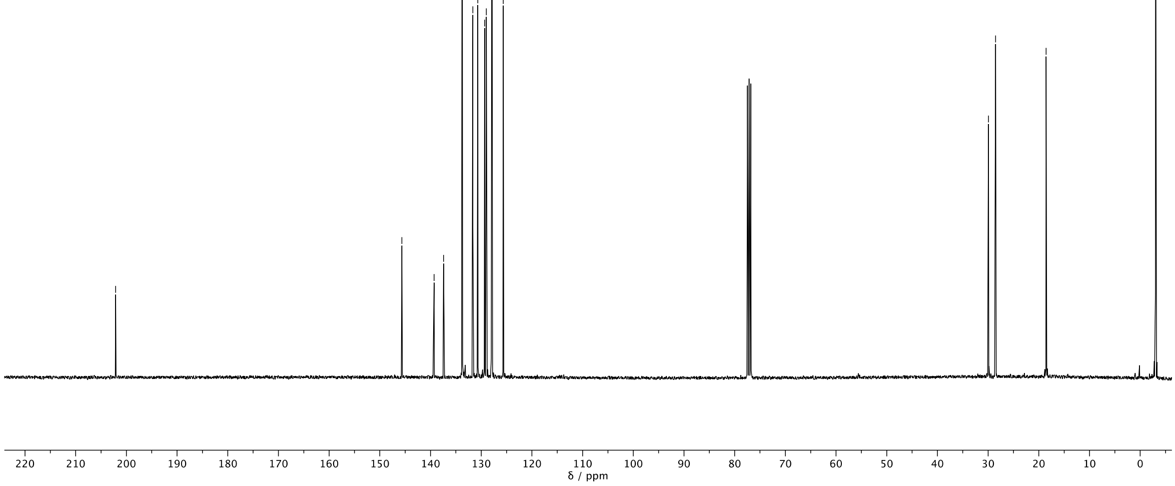

2ac

^{1}H NMR (400 MHz, $CDCl_3$)

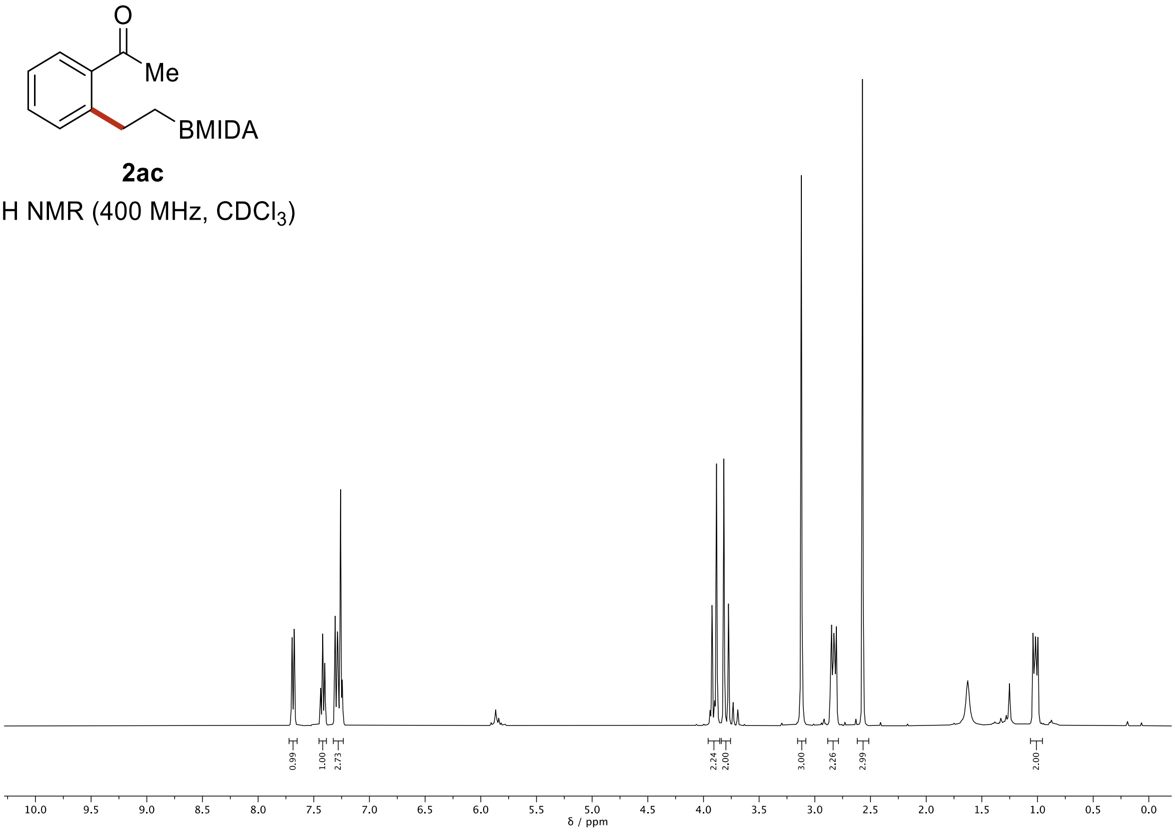

2ac

^{13}C NMR (101 MHz, $CDCl_3$)

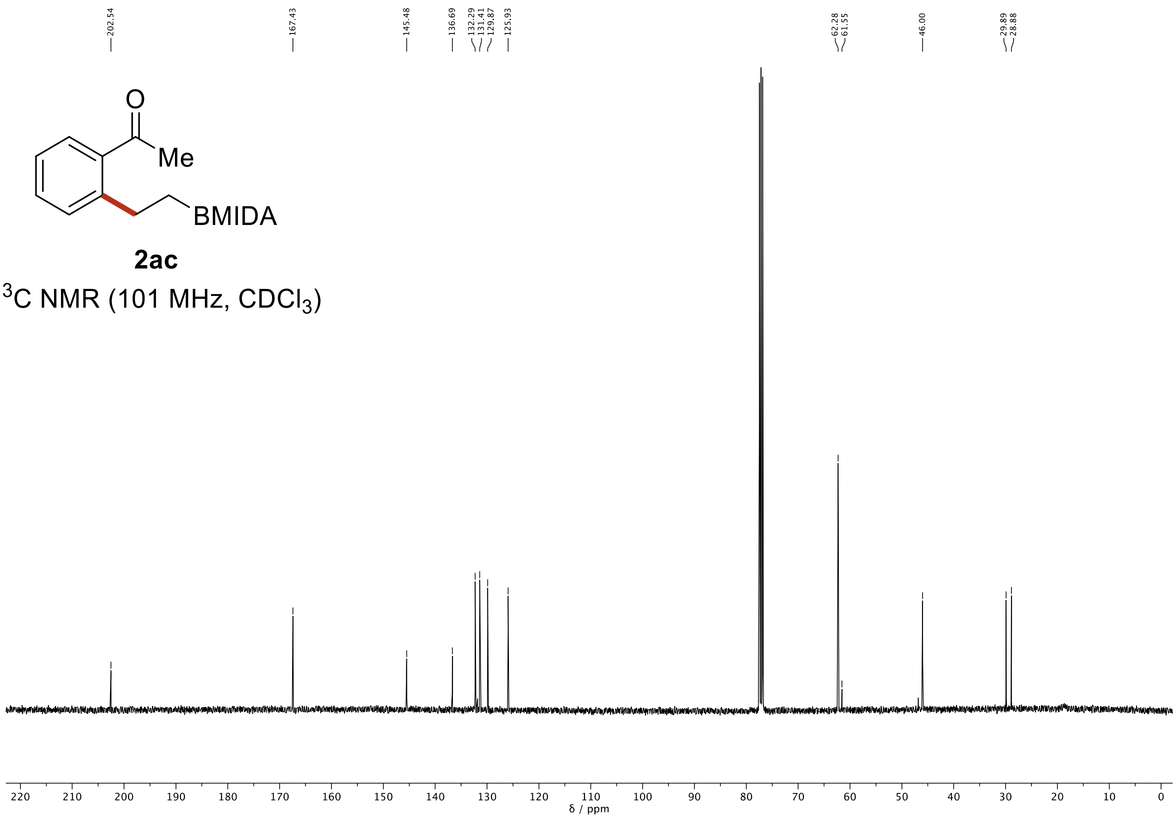

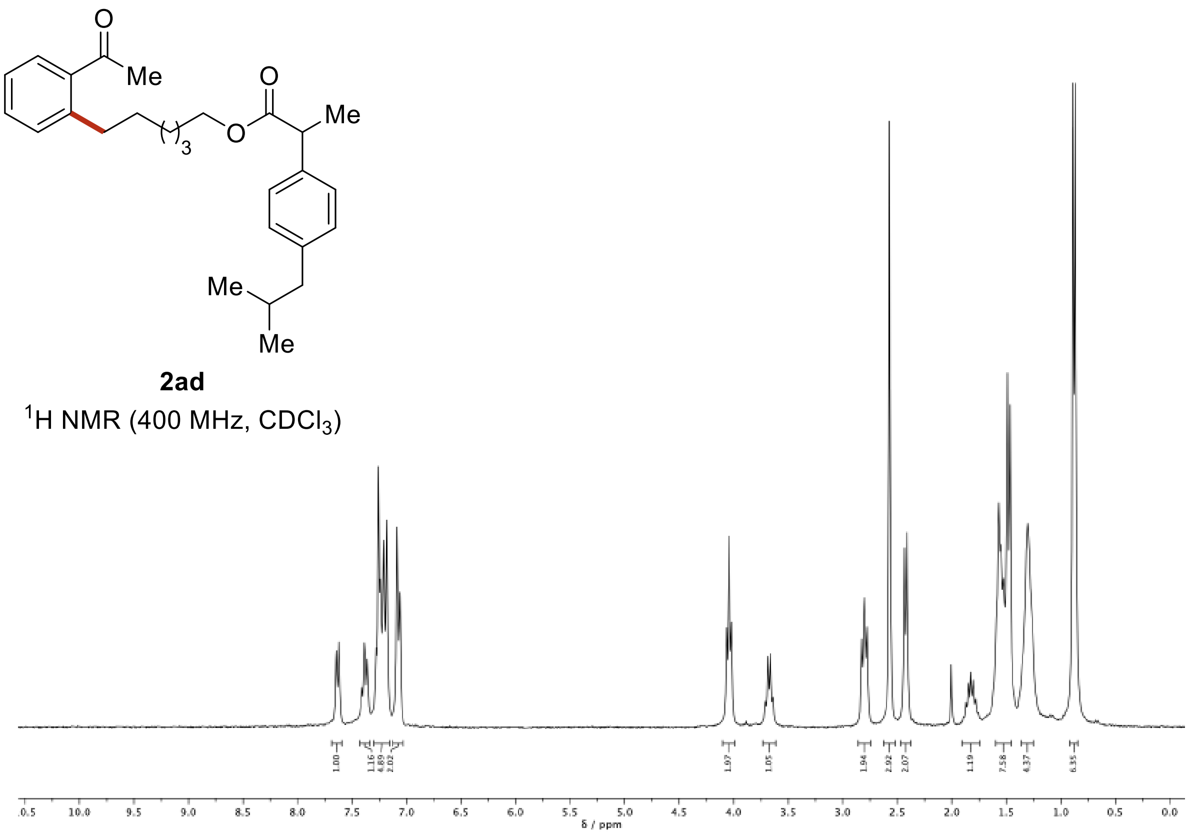

2ad

^{1}H NMR (400 MHz, $CDCl_3$)

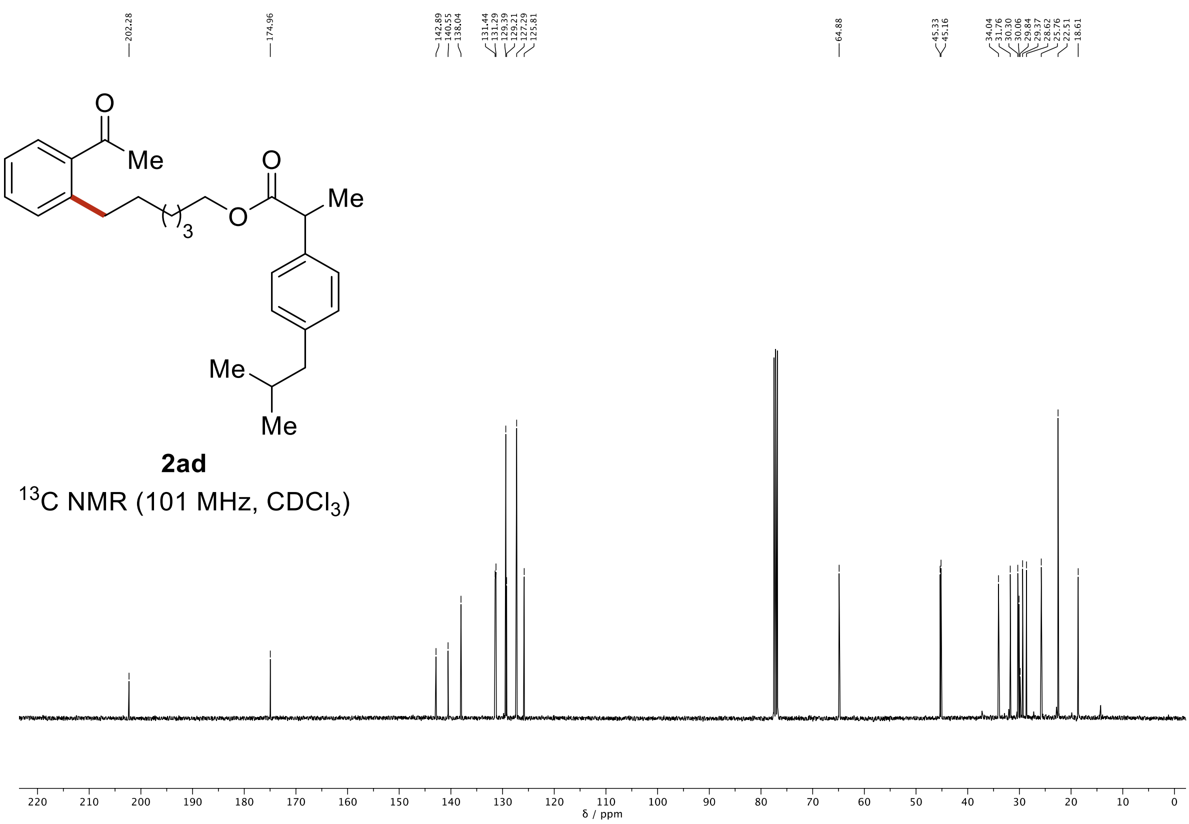

2ad

^{13}C NMR (101 MHz, $CDCl_3$)

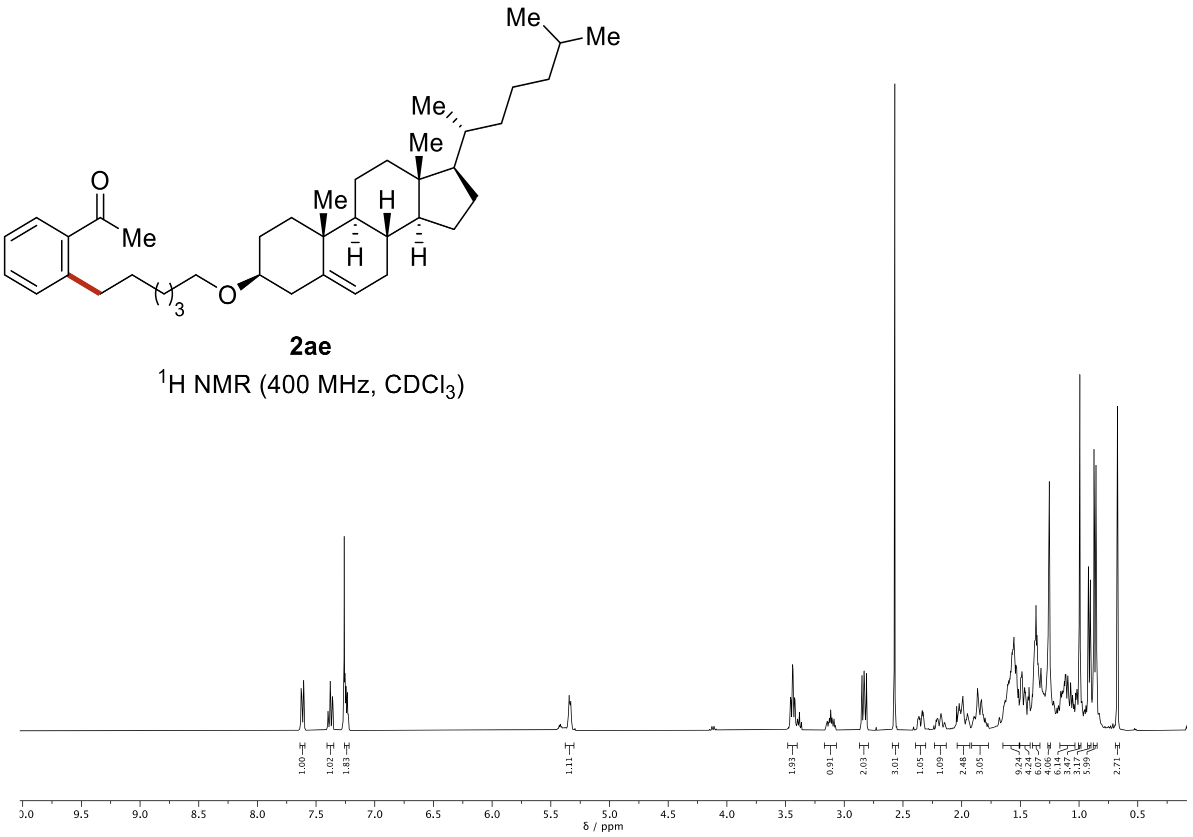
O
Me
Me
Me
Me
Me
H
H
H
O
3
2ae
1H NMR (400 MHz, CDCl3)
δ / ppm

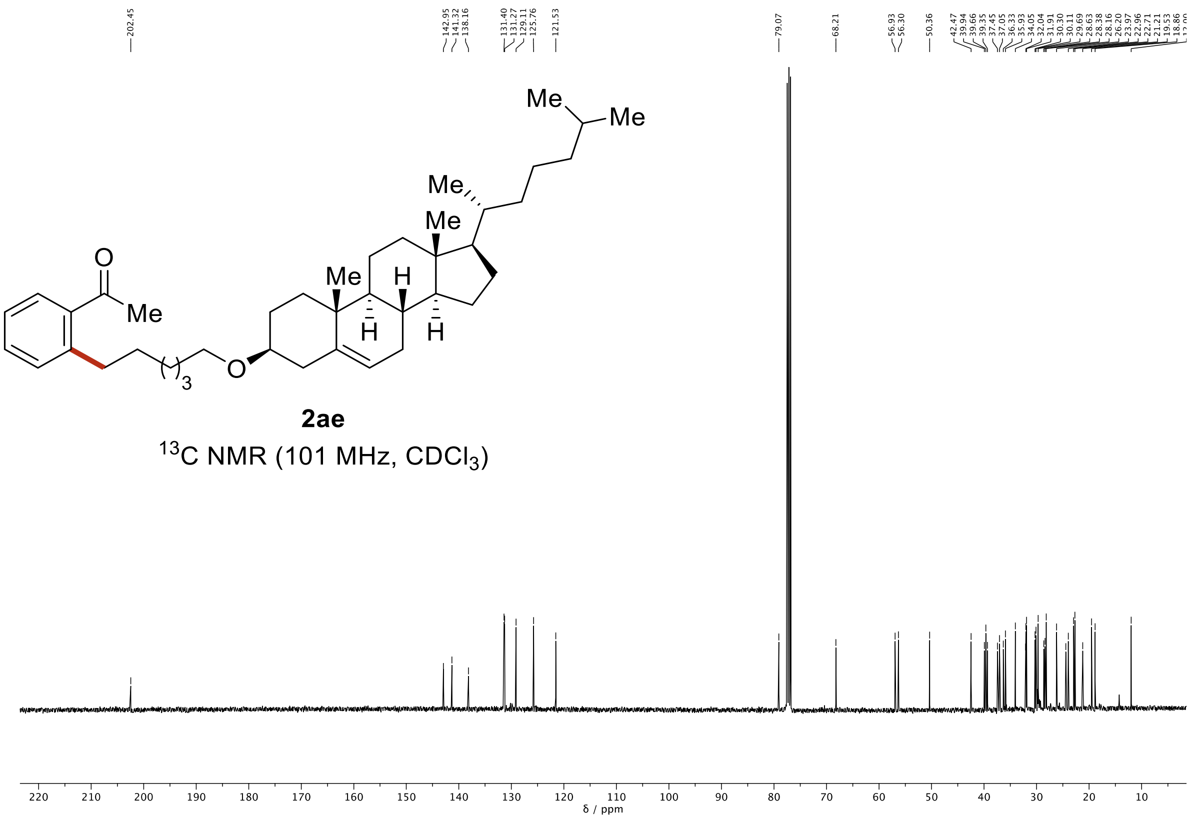
202.45
142.95
141.32
138.16
131.40
131.27
129.11
125.76
121.53
79.07
68.21
56.93
56.30
50.36
O
Me
Me
Me
Me
Me
H
H
H
O
3
2ae
13C NMR (101 MHz, CDCl3)
δ / ppm

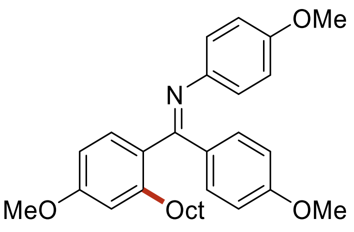

3a

^{1}H NMR (400 MHz, $CDCl_3$)

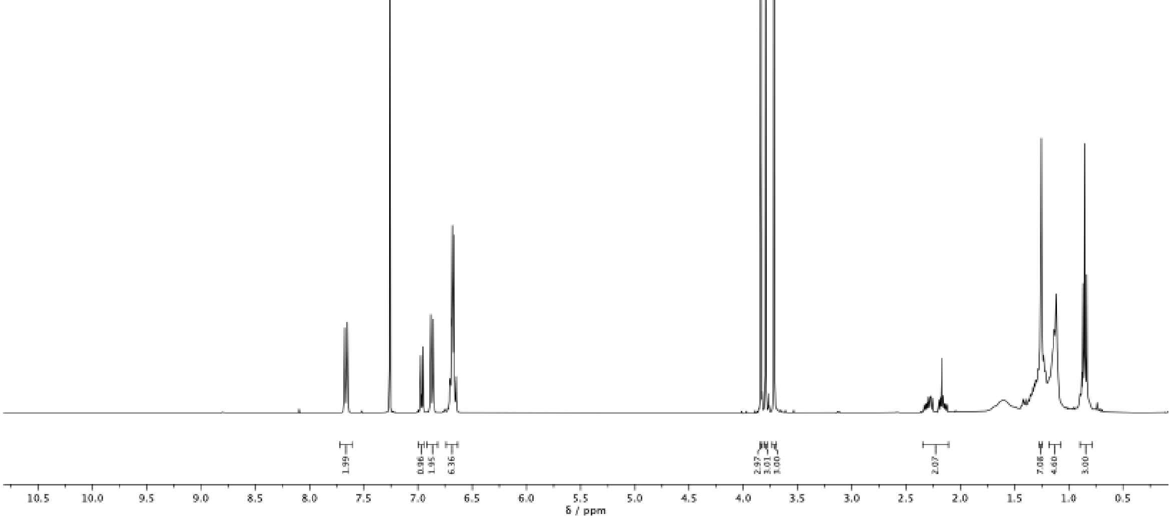

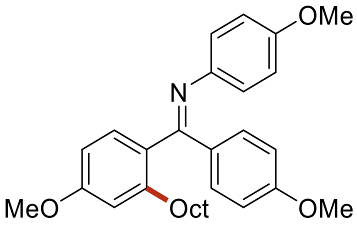

3a

^{13}C NMR (101 MHz, $CDCl_3$)

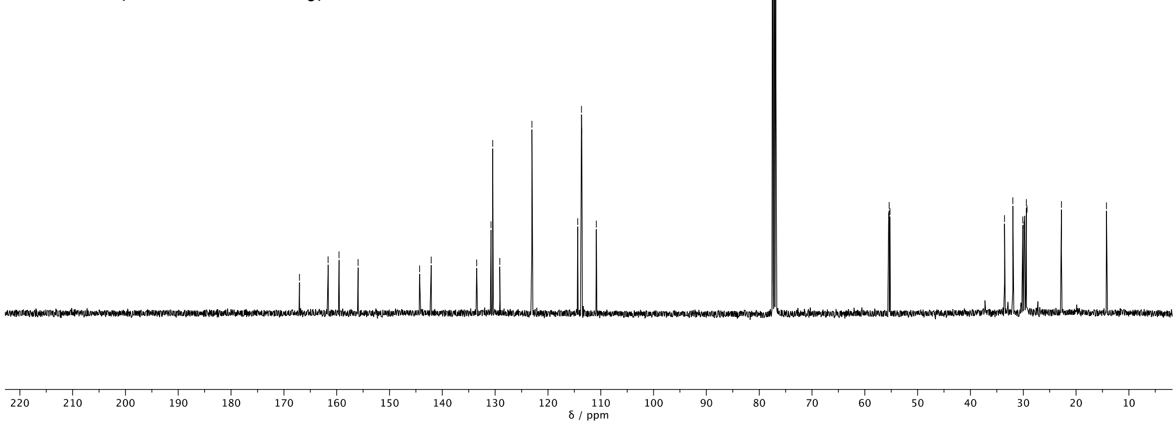

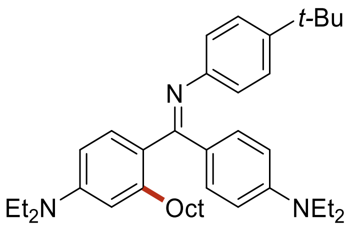

3b

^{1}H NMR (400 MHz, $CDCl_3$)

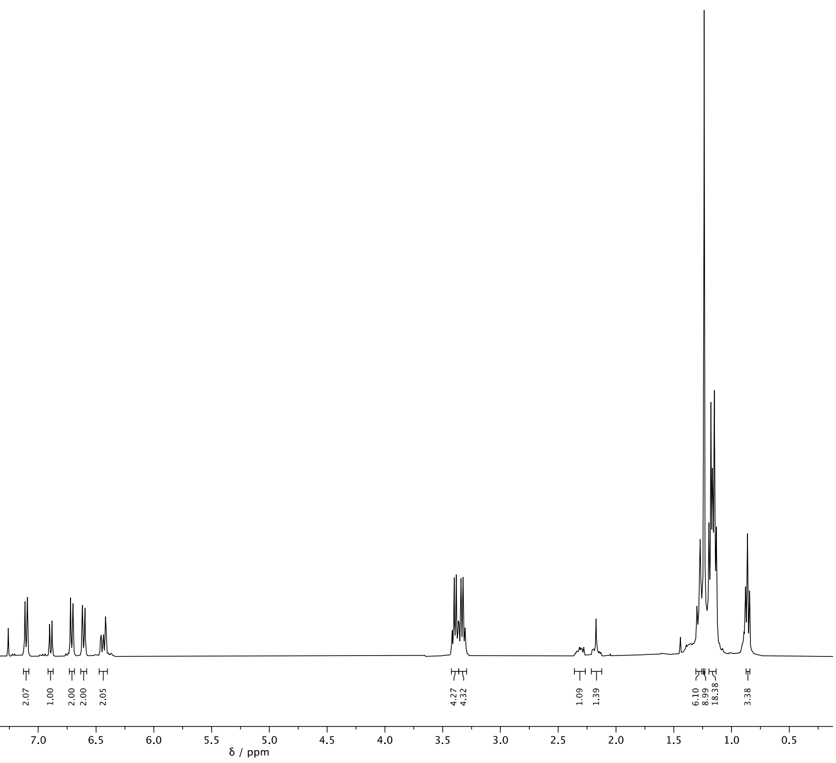

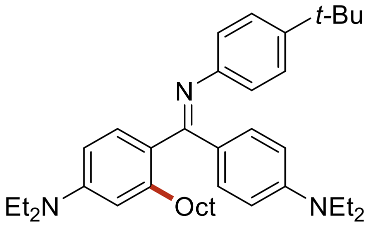

3b

^{13}C NMR (101 MHz, $CDCl_3$)

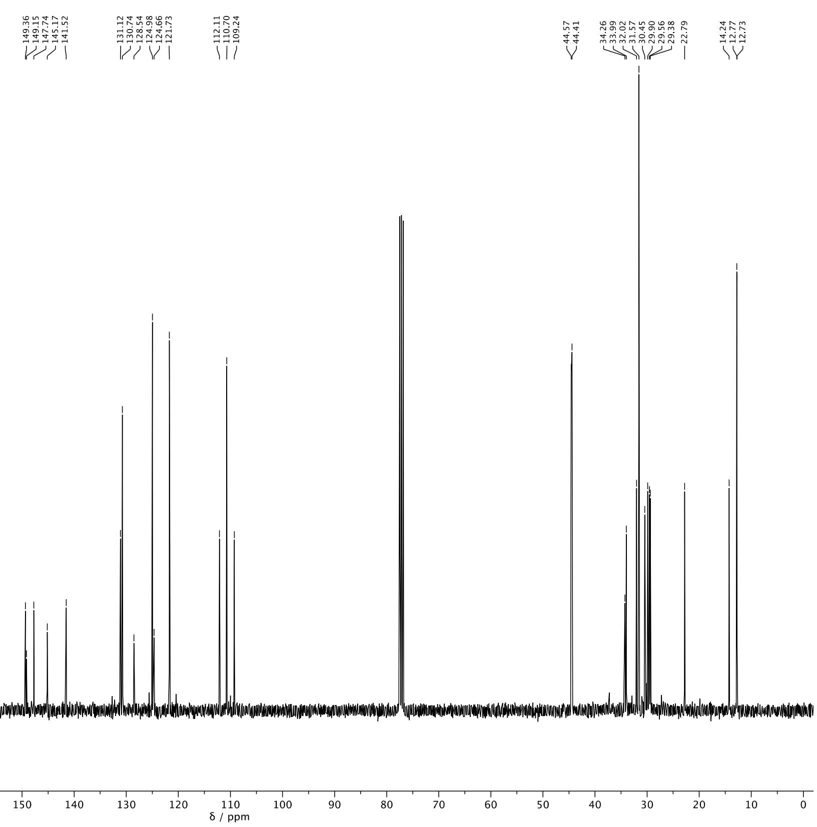

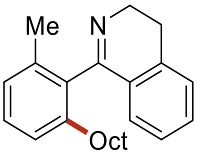

3c

^{1}H NMR (400 MHz, $CDCl_3$)

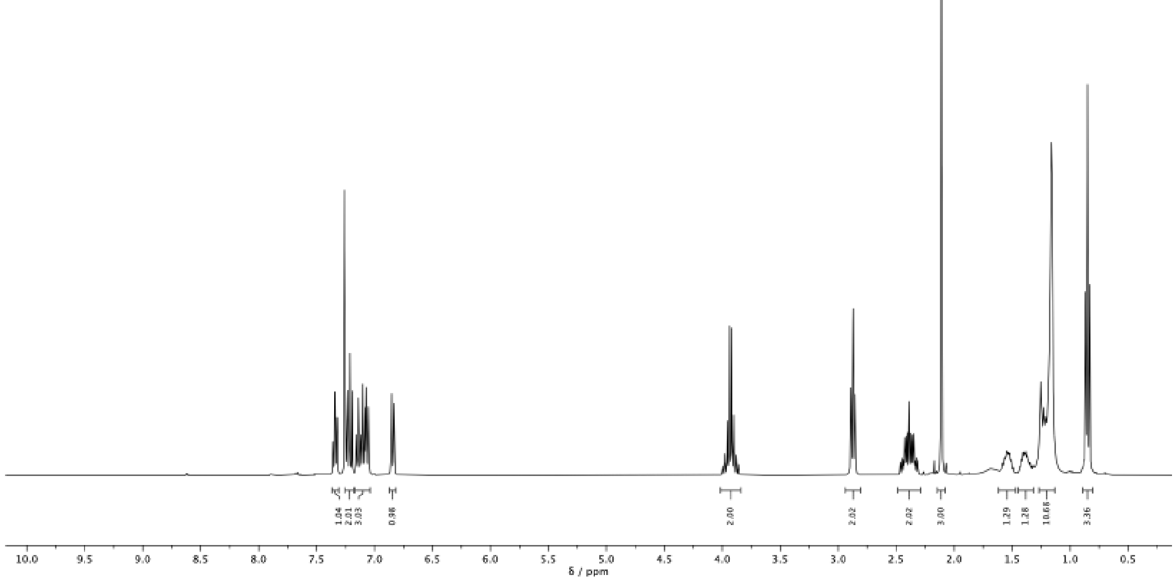

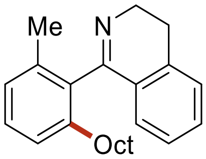

3c

^{13}C NMR (101 MHz, $CDCl_3$)

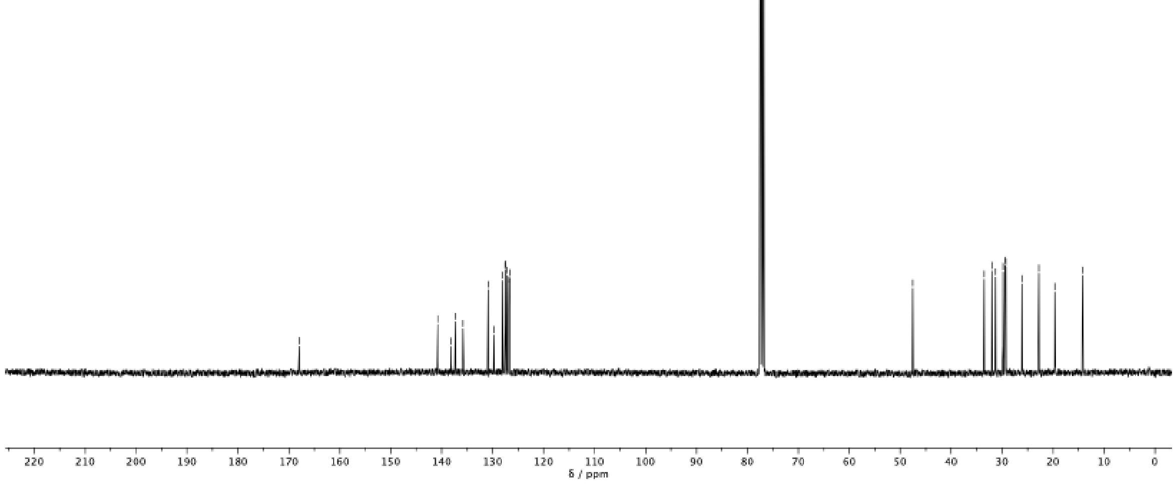

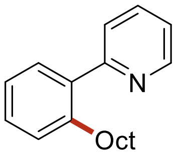

3d

^{1}H NMR (300 MHz, $CDCl_3$)

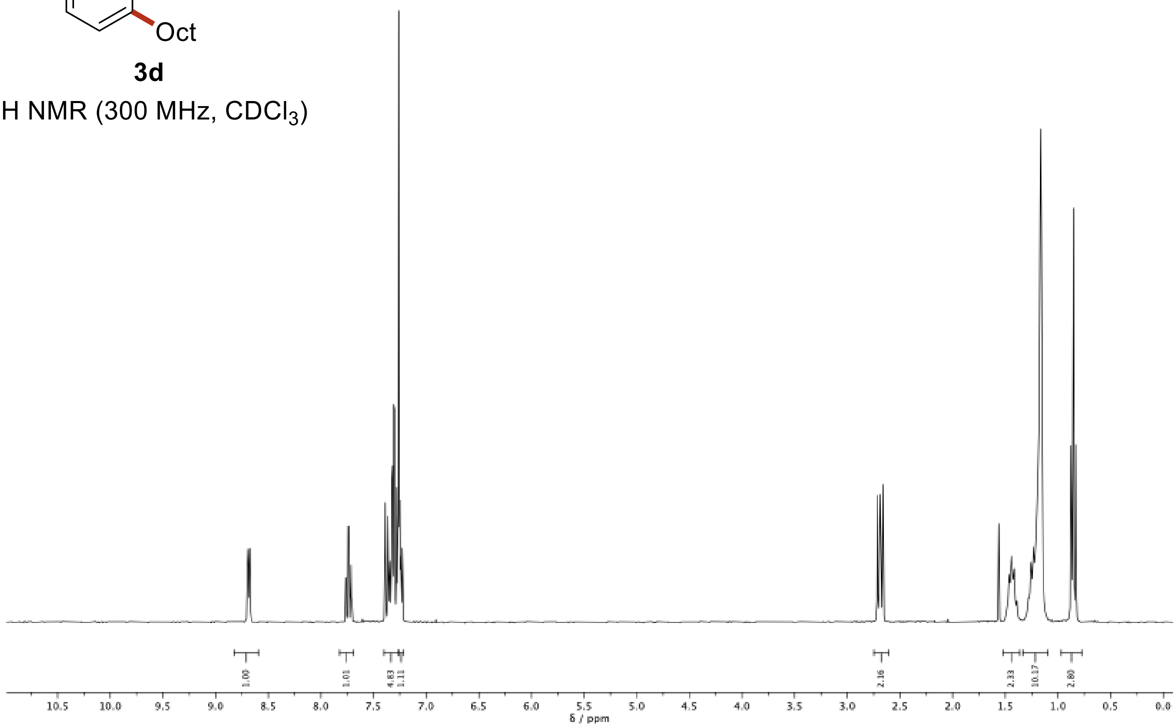

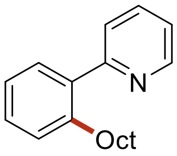

3d

^{13}C NMR (101 MHz, $CDCl_3$)

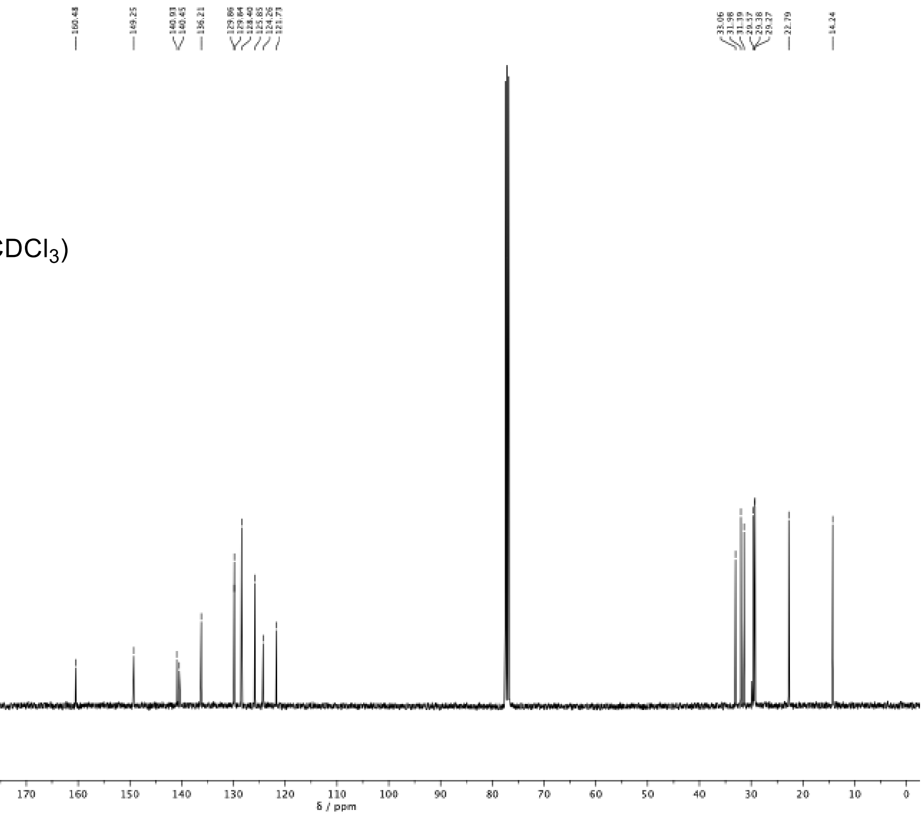

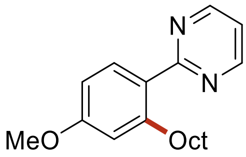

3e

^{1}H NMR (400 MHz, $CDCl_3$)

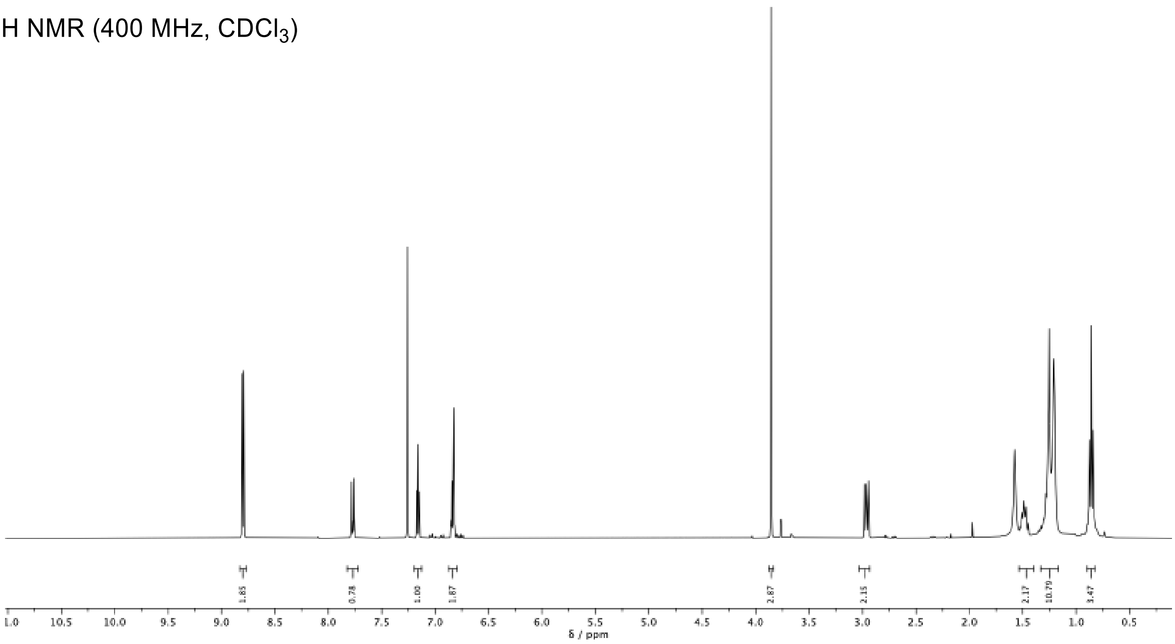

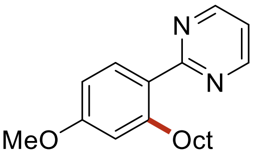

3e

^{13}C NMR (101 MHz, $CDCl_3$)

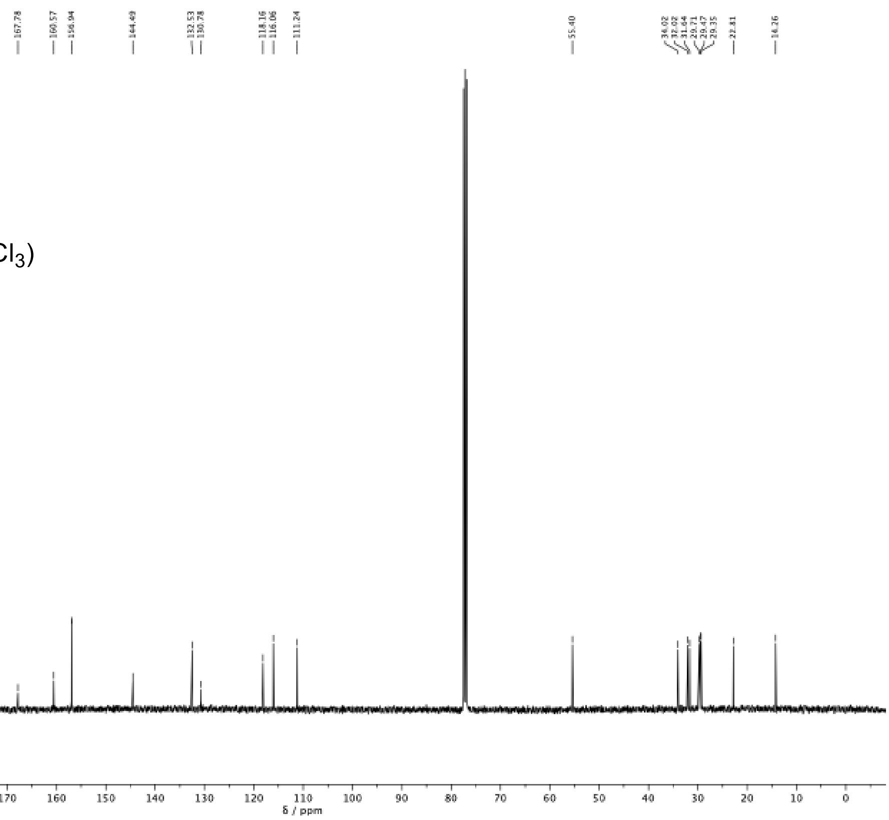

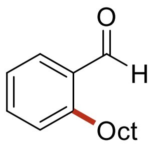

3f

^{1}H NMR (300 MHz, $CDCl_3$)

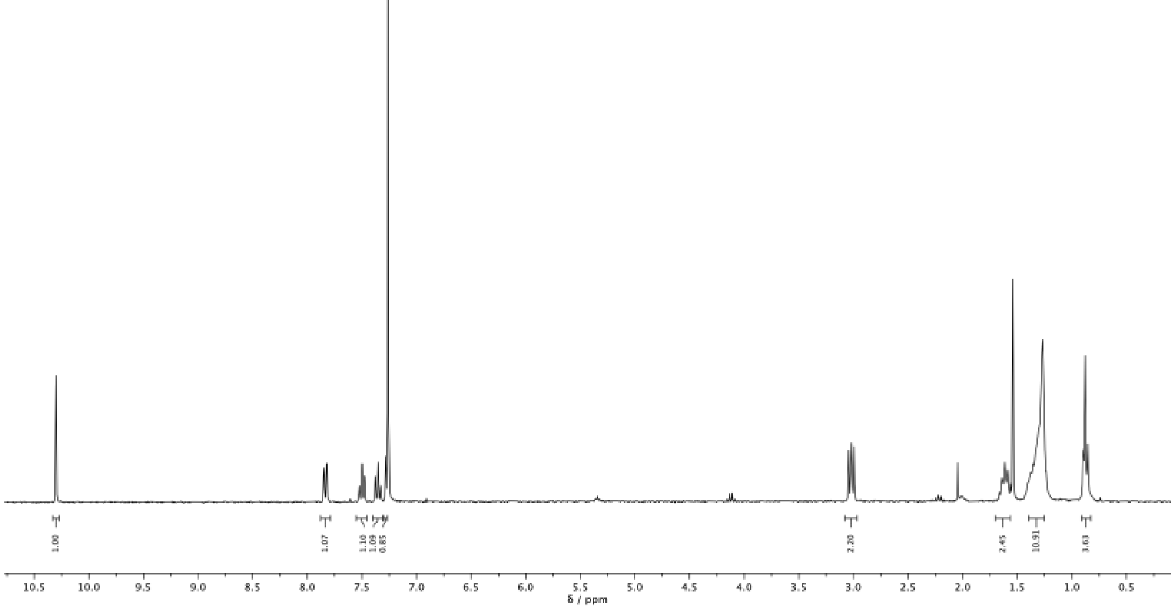

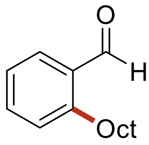

3f

^{13}C NMR (101 MHz, $CDCl_3$)

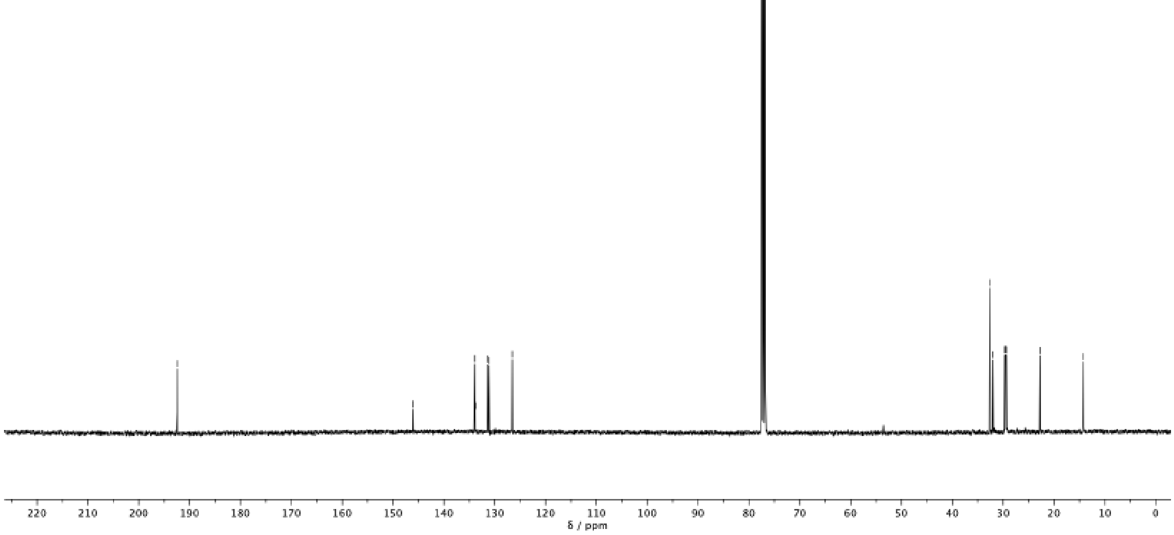

Supplement: SC-015-D4SC06005A-s001 [file SC-015-D4SC06005A-s001.pdf]
